# Stereoselective Direct *N*‐Trifluoropropenylation of Heterocycles with a Hypervalent Iodonium Reagent

**DOI:** 10.1002/chem.202102840

**Published:** 2021-10-06

**Authors:** János T. Csenki, Ádám Mészáros, Zsombor Gonda, Zoltán Novák

**Affiliations:** ^1^ ELTE “Lendület” Catalysis and Organic Synthesis Research Group Institute of Chemistry Eötvös Loránd University Pázmány Péter stny. 1/A 1117 Budapest Hungary

**Keywords:** enamines, iodonium salt, Michael addition, nitrogen heterocycles, trifluoromethyl

## Abstract

The availability and synthesis of fluorinated enamine derivatives such as *N*‐(3,3,3‐trifluoropropenyl)heterocycles are challenging, especially through direct functionalization of the heterocyclic scaffold. Herein, a stereoselective *N*‐trifluoropropenylation method based on the use of a bench‐stable trifluoropropenyl iodonium salt is described. This reagent enables the straightforward trifluoropropenylation of various N‐heterocycles under mild reaction conditions, providing trifluoromethyl enamine type moieties with high stereoselectivity and efficiency.

## Graphical abstract

*E*‐selective trifluoropropenylation of versatile N‐heterocycles was developed with the utilization of trifluoropropenyliodonium salts. The procedure enables the straightforward direct introduction of the trifluoroalkenyl moiety into heterocyclic scaffolds under simple and mild reaction conditions.

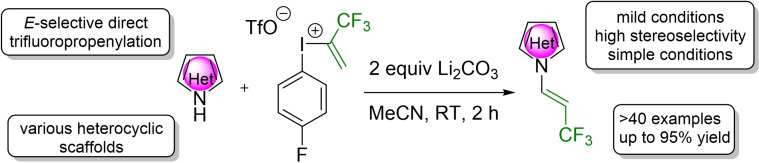

Nitrogen heterocycles are one of the most common and important molecular motifs in nature,^[1]^ and a significant portion of biologically active molecules are based on this framework.^[2]^ Among the subclasses of nitrogen heterocycles, *N*‐alkenylated molecules are an important class from a synthetic aspect due to the transformability of the double bond^[3]^ and several derivatives are used in the field of medicinal chemistry.^[4]^ *N*‐alkenylations can be achieved with hydroamination of alkynes^[5]^ or the functionalization of vinylic species equipped with leaving groups via metal‐catalyzed^[6]^ or metal‐free reactions.^[7]^ The key aspect of these direct functionalizations is the stereoselectivity, thus the development of *E‐* or *Z*‐selective methods are desirable, however this is challenging.

Fluorine‐containing molecules are more and more desired compounds in pharmaceutical, agrochemical and material sciences.^[8]^ The strategic incorporation of fluorine atoms in organic compounds is a valuable tool for researchers to satisfy the developing needs and expectations for new API's.^[9]^ Therefore, versatile synthetic methodologies were developed for the installation of different fluorous functional groups into organic scaffolds.^[10]^ Among these fluorous motifs, the 3,3,3‐trifluoropropen‐1‐yl group is relatively rare and methods which enable its incorporation into heterocycles through the formation of new C−N bond are hardly available and mostly limited to some specific substrates, such as cyclic amides.^[11]^ The *N*‐trifluoropropenylation of enamides such as pyrrolidin‐2‐one can be achieved through the palladium‐^[11a]^ or copper‐catalyzed^[11b]^ trifluoromethylation of *N*‐vinylpyrrolidin‐2‐one with CF_3_I or under photocatalytic conditions (Figure 1a),[^11c^, ^11d^, ^11e^] while the same product can be obtained with trifluoropropionaldehyde (Figure 1b).^[11f]^ The use of 3,3,3‐trifluoropropyne gas as a C_2_‐CF_3_ surrogate requires the handling of gaseous reagent, but it was successfully applied as Michael acceptor in its reaction with 2’‐deoxyiodouridine used for iodinated DNA bases (Figure 1c).^[11g]^ The NH functionalities in uracil and thymine can be trifluoropropenylated with 2‐bromo‐3,3,3‐trifluoropropene with moderate stereoselectivity (Figure 1c).^[11h]^

**Figure 1:**
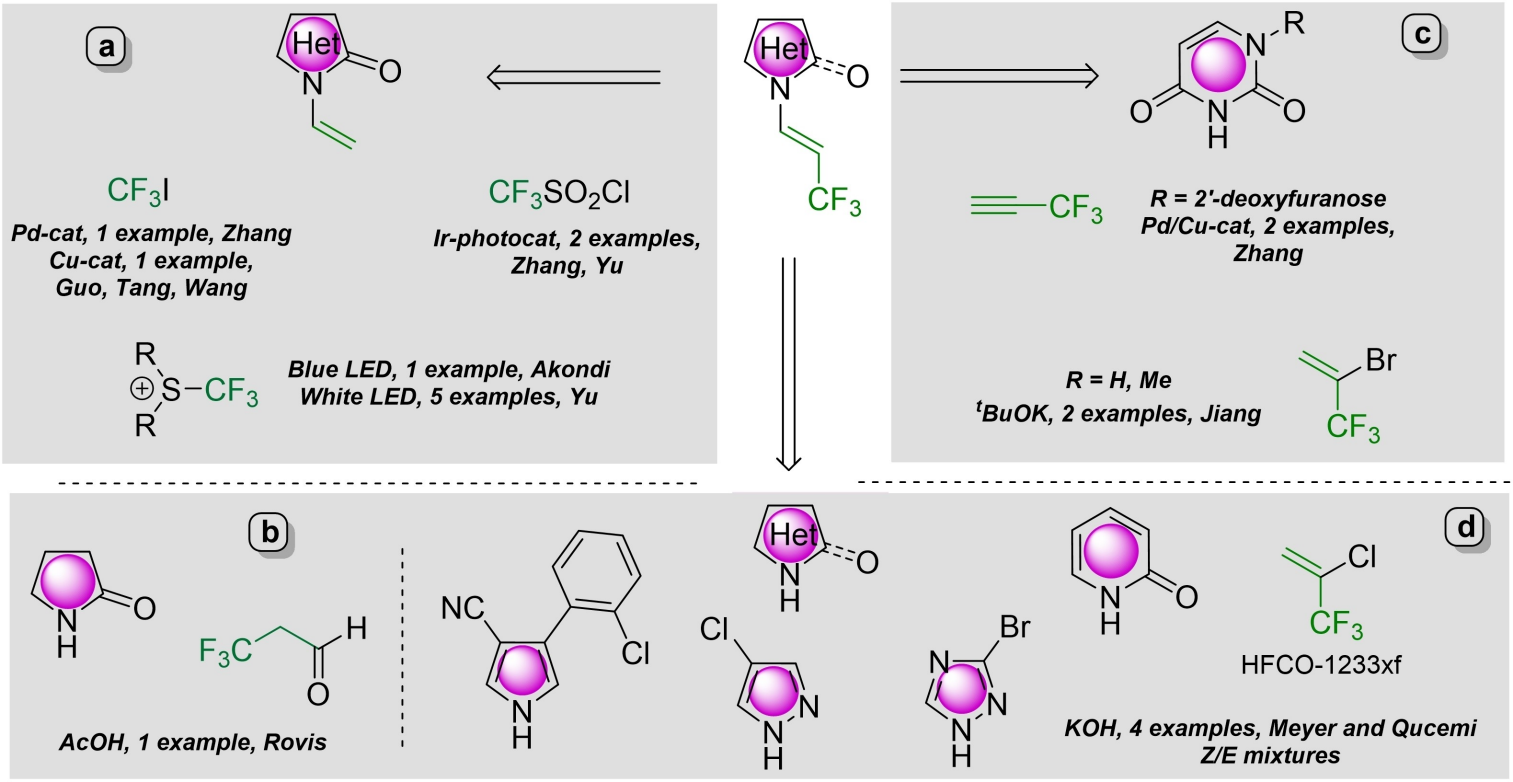
Full spectrum of synthetic approaches to *N*‐trifluoropropenyl heterocycles

However, the applicability of the previous methods was demonstrated with only a few trifluoromethylated examples.

Recently, Meyer and Qacemi demonstrated the *N*‐trifluoropropenylation of heterocycles such as pyrrole, pyrazole, triazole, and pyridone with the utilization of 2‐chloro‐3,3,3‐trifluoroprop‐1‐ene (HCFO‐1233xf) in a base‐promoted transformation under mild reaction condition (Figure 1d).^[12]^ The reaction favors the formation of the *Z*‐isomer, but the *Z*/*E* isomeric ratio of trifluoropropenyl heterocycles was between 6 : 4–7 : 3.

Taking into consideration the importance of the compound class and the limitations of their versatile and selective synthesis, we aimed to develop a novel procedure which enables the direct introduction of trifluoropropenyl functional groups into heterocycles through the formation of new C−N bond in a selective and efficient manner under mild reaction conditions enabled by hypervalent iodonium species.^[13]^

In our laboratory, we recently designed and synthesized a bench stable trifluoroisopropenyl iodonium salt (**1**) and studied its reactivity toward nitrogen nucleophiles. Primary amines provided trifluoromethylaziridines,^[14]^ while the utilization of secondary amines ensured the synthesis of trifluoroalkyl amines and diamines through aziridinium intermediate.^[15]^ To complete the spectrum of applicable nitrogen nucleophiles, we studied the reaction of nitrogen heterocycles with the trifluoropropenyl‐iodonium salt to discover new synthetic possibilities and develop a stereoselective, versatile, and efficient methodology to the access of *N*‐trifluoropropenyl heterocyclic species through a one‐step direct functionalization (Figure 2).

**Figure 2:**
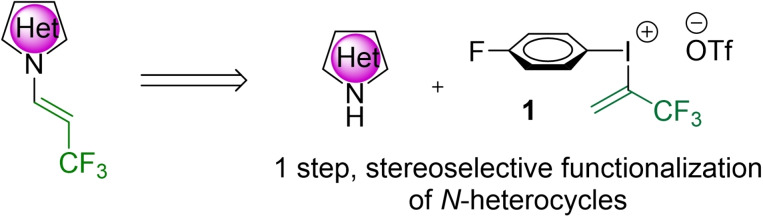
Research goal: new method for direct trifluoropropenylation of N‐heterocycles with iodonium reagent.

We choose benzotriazole (**2**) as model substrate for the reactivity and optimization studies, focusing on the effect of base and solvent. To our delight, in dichloromethane with Li_2_CO_3_ base the desired trifluoropropenylation of benzotriazole with 1.2 equivalent of iodonium reagent **1** the trifluoropropenylation took place on *N*‐1 resulting **3** in 83 % conversion in 2 h at 25 °C (Table 1, Entry 1). However, formation of constitutional isomers was also observed as minor products^[16]^ In further optimization, we aimed to improve both the selectivity and the efficiency of the trifluoropropenylation. In this regard, the use of other carbonates such as sodium and potassium resulted lower conversions (80 % and 60 % respectively, Table 1, Entry 2 and 3). In the presence of NaH, product **3** formed in 75 % conversion, while collidine could also be used effectively as simple organic base with complete reaction, providing the major product **3** in 85 % conversion. Next, we studied some solvent‐base pairs including EtOAc, THF, and MeCN as solvents and Li_2_CO_3_, Na_2_CO_3_, and collidine as bases to find the best combination for the selective trifluoropropenylation (Table 1, entries 6–11). We found that the combination of MeCN and Li_2_CO_3_ provided the best conditions for the reaction, which took place in full regio‐ and stereoselectivity and the *E*‐trifluoropropenylated benzotriazole **3** was isolated in 95 % yield after workup.

**Table 1:** . Trifluoropropenylation of benzotriazole.^[a]^

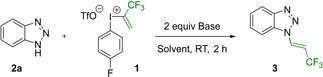			
Entry	Solvent^[b]^	Base	Conversion^[c]^
1	DCM	Li_2_CO_3_	83
2	DCM	Na_2_CO_3_	80
3	DCM	K_2_CO_3_	60
4	DCM	NaH^[d]^	75
5	DCM	Collidine	85
6	EtOAc	Li_2_CO_3_	80
7	THF	Li_2_CO_3_	85 (76)
8	THF	Na_2_CO_3_	80 (73)
9	MeCN	Collidine	66
10	MeCN	Na_2_CO_3_	83 (76)
11	MeCN	Li_2_CO_3_	96 (95)

[a] Reaction conditions: 0.20 mmol benzotriazole, 1.2 equiv. iodonium salt, 2.0 mL solvent, 2.0 equiv. base, room temp. 2 h [b] dry solvents [c] conversion to the main product determined by GC (isolated yield of **3**) [d] 1.1 equiv. NaH

After finding the optimal solvent‐base pair for the transformation (we performed the same solvent‐base optimization with indazole, and obtained the same result),^[16]^ we were also able to lower the iodonium salt loading to 1.1 equivalents without any diminuation of isolated yield. Increasing the temperature had no effect on reaction time or yield.^[16]^

With the optimized conditions in hand, we aimed to explore the scope of the reaction with the *E*‐selective *N*‐trifluoropropenylation of various heterocycles bearing NH functionality. In this regard, reactions of pyrazoles provided the desired products under the optimized reaction conditions. Although the parent compound 1*H*‐pyrazole could be trifuoropropenylated with full conversion (not shown),^[16]^ the isolation of this product was problematic due to its volatility. The presence of Br substituent on the pyrazole ring enabled the isolation of **4** in 80 % yield after the trifluoropropenylation.

Next, we examined a series of 3,5‐symmetrically disubstituted pyrazoles such as 3,5 dimethyl‐pyrazole and its 4‐iodinated derivative and we were able to isolate the corresponding products **5** and **6** in 63 and 80 % yield, respectively. Symmetric 3,5‐diaryl pyrazoles were trifluoropropenylated without difficulties and the products **7**–**13** were isolated in 53–90 % yield range, similarly to the 4‐iodo derivative **14** which was isolated in 50 % yield.

In case of non‐symmetrically substituted pyrazoles, the 3‐phenyl and 3‐trifluoromethyl‐4‐carbetoxy substituted pyrazoles gave exclusively one constitutional isomer **15** and **16** in 86 % and 63 % yield, indicating the steric and electronic influence on the functionalization. Other non‐symmetrically substituted 3,5‐diaryl pyrazoles also reacted smoothly, however, only inseparable mixtures of constitutional isomers of **17**, **18**, and **19** could be isolated. In contrast, when 3‐phenyl‐5 trifluormethylpyrazole was transformed, isomers **20** and **21** were isolable in 31 and 36 % yield, respectively, similarly to products **22** and **23** which were separated and isolated with similar efficiency (48 and 39 % yield, respectively).

In the 1*H*‐imidazole series, 4‐bromo, 2‐ethyl, 4,5‐diphenyl and 4‐ethylcarboxylate derivatives underwent selective trifluoropropenylation and the corresponding enamines **24**, **25**, **26**, and **27** were isolated in 61 %, 50 %, 89 % and 95 % yield, respectively.

Among the five membered nitrogen heterocycles 5‐phenyl‐1*H*‐tetrazole gave one contitutional isomeric product (**28**) which was isolated in 76 % yield (Scheme 1).

**Scheme 1:**
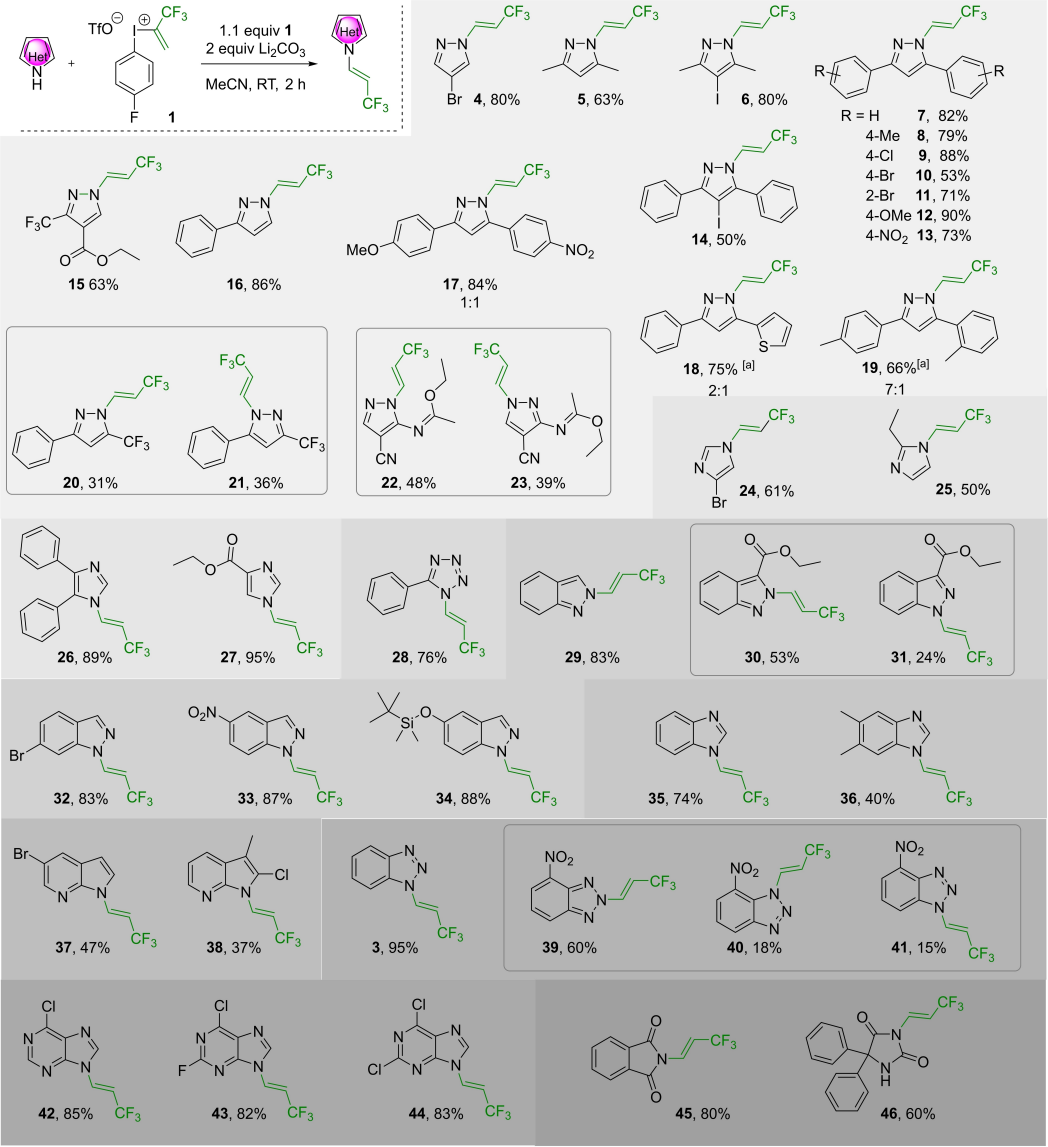
Substrate scope. N−H heterocycles (0.30‐1.00 mmol, 1.0 equiv), Li_2_CO_3_ (0.60‐2.00 mmol, 2.0 equiv), trifluoroisopropenyl iodonium salt **1** (0.33–1.10 mmol, 1.1 equiv) and 3–10 mL of MeCN, room temperature, 2 h. [a] the major regioisomer is depicted on the scheme

Next, we studied the reactivity of the benzene‐fused nitrogen heterocycles having a ring NH functionality. Indazoles were also successfully trifluoropropenylated with iodonium reagent under the optimized conditions.

The reaction of unsubstituted indazole framework provided *N*‐2‐trifluoropropenylated product **29** selectively in 83 % yield.

Presence of ethylcarboxylate group in the pyrazole ring caused the formation of two isomers **30** and **31** which were isolated after separation in 53 % and 24 %. Substituents such as Br, NO_2_, and TBDMSO on the benzene ring of indazole selectively form *N*‐1‐trifluoropropenylated products **32**, **33**, and **34** with similar high efficiency (83 %, 87 % and 88 % respectively). Beside the indazole derivatives, benzimidazoles were also successfully transformed and the desired products (**35**, **36**) were formed selectively and isolated in 74 % and 40 % yield.

In this series, we aimed for the transformation of indole, but we observed the formation of complex reaction mixture without the detection of the desired product.^[17]^ However, azaindoles were trifluoropropenylated successfully and enamine products **37** and **38** were isolated in 47 % and 37 % yields, respectively.

Although our model substrate benzotriazole used for the optimization studies, gave exclusively one product (**3**) under the optimized conditions in 95 % isolated yield, the reaction with its 4‐nitro‐substituted derivative gave three regioisomers **39**, **40** and **41**, which were isolated in 60 %, 18 %, and 15 % respectively after chromatographic purification.

Using the iodonium based functionalization protocol *N*‐trifluoropropenylated purine derivatives **42**, **43**, and **44** were also isolated in high yields, giving the products in 85 %, 82 %, and 83 %, respectively.

To our delight, not just aromatic heterocycles, but also heterocyclic imides were transformed efficiently using the developed methodology. Phthalimide reacted smoothly and the corresponding trifluoropropenyl‐phthalimide **45** was isolated in 80 % yield. Phenytoin was also a suitable substrate for the transformation, and we were able to isolate the *N*‐trifluoropropenylated product **46** in 60 % yield.

On the basis of our previous studies, we propose a mechanism for the trifluoropropenylation (Figure 3).

**Figure 3:**
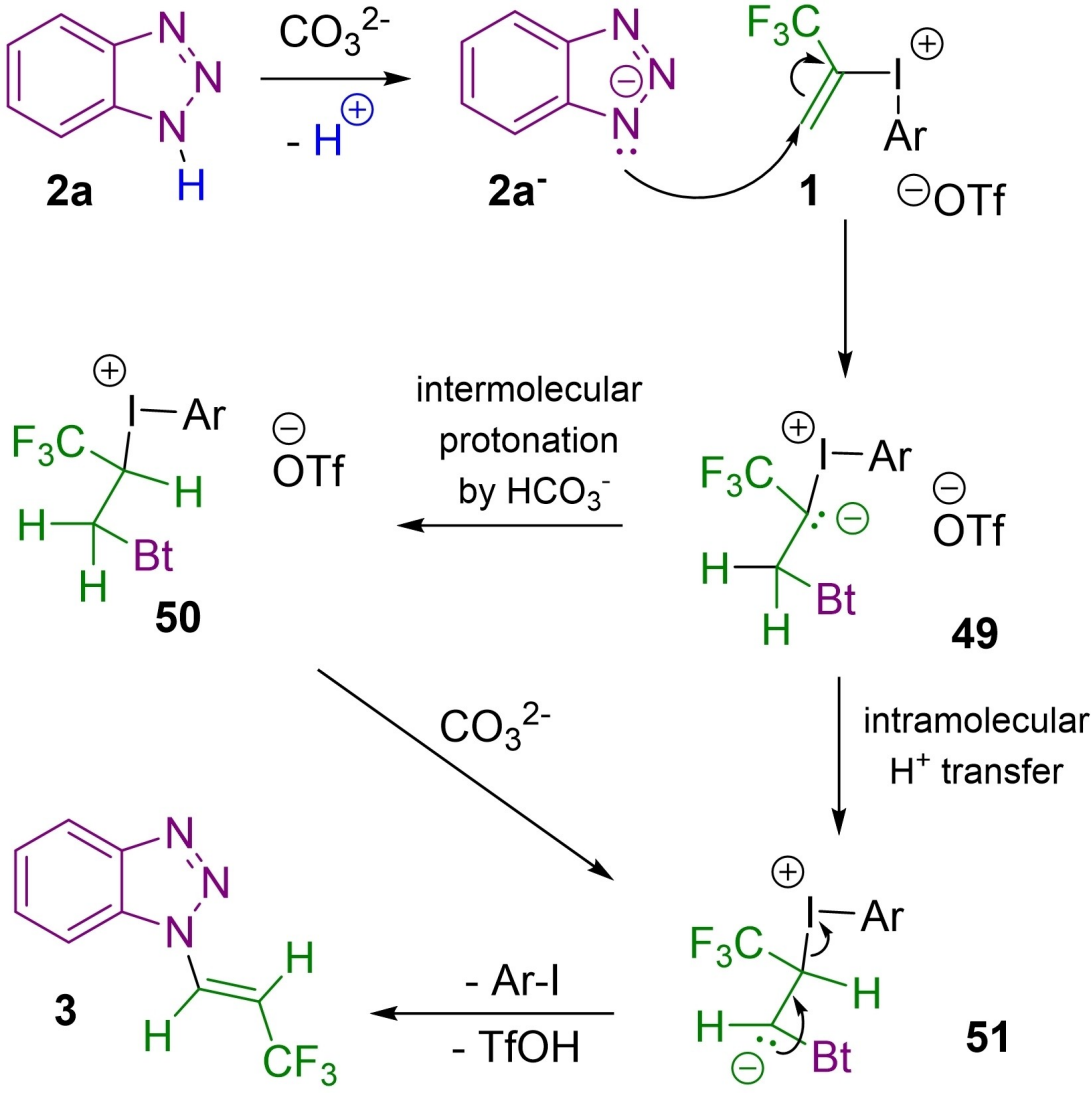
Proposed reaction mechanism.

After the deprotonation step, the heterocyclic anion attacks to the terminal sp^2^ carbon of the trifluoropropenyl moiety in a Michael addition type reaction, then the formed benzotriazolyl iodonium ylide (**49**) undergoes intramolecular proton transfer resulting anion **51**. Alternatively, the stabilized carbanion (**49**) can be protonated by the HCO_3_^−^ ion in an intermolecular fashion forming intermediate **50** which can be deprotonated by the base, with the resulting anion **51** undergoing *E*‐selective elimination step to provide the final product **3**.

To support the mechanistic hypothesis, especially the relevance of intramolecular and intermolecular proton transfers, we performed the reactions with both [1*H*] and [1*D*]‐benzotriazole (**2 a** and **[D]2a**) in MeCN and d_3_‐MeCN, and measured the deuterium incorporation in the product (Scheme 2). The [1*H*] substrate **2 a** gave product **3** with 0 % deuterium incorporation both in MeCN and d_3_‐MeCN (isolated yields 95 % and 87 %). Trifluoropropenylation of **[D]2a** in MeCN resulted 19 % deuterium incorporation, while in d_3_‐MeCN the same reaction provided the product with 24 % deuterium incorporation. In the presence of 1 equivalent of D_2_O, the deuterium incorporation increased about 15–20 % independently from the substrate and the applied solvent, showing the possibility of intermolecular protonation. These results support that both H‐atoms of trifluoropropenyl group of the product are dominantly derived from the reagent **1** through intramolecular proton transfer, but intermolecular base assisted proton transfer could also operate, beside some minor solvent effect on the proton transfer.

**Scheme 2:**
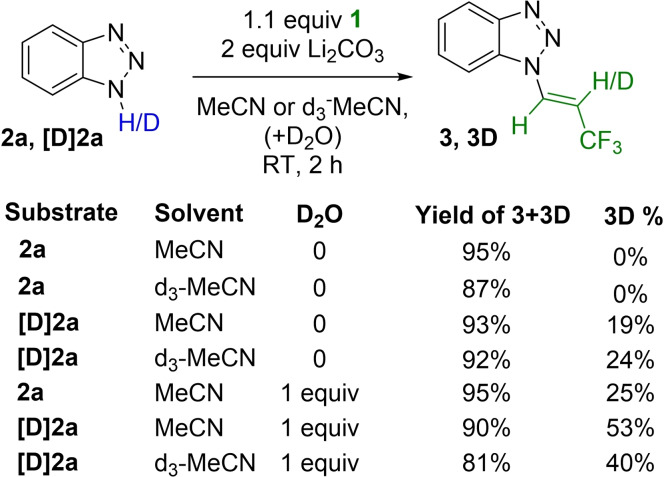
Study of deuterium transfer.

In summary, we developed a novel methodology for the direct *N*‐trifluoropropenylation of heterocyclic molecules with the use of a trifluoropropenyl iodonium salt. The reaction enables the stereoselective synthesis of trifluoromethyl enamines having the potential in further transformations and adds to the synthetic applicability of trifluoropropenyl iodonium species toward versatile nitrogen nucleophiles.

## Conflict of interest

The authors declare no conflict of interest.

## Supporting information

As a service to our authors and readers, this journal provides supporting information supplied by the authors. Such materials are peer reviewed and may be re‐organized for online delivery, but are not copy‐edited or typeset. Technical support issues arising from supporting information (other than missing files) should be addressed to the authors.

Supporting Information
