# Supplementary material for: Stereoselective Direct N‐Trifluoropropenylation of Heterocycles with a Hypervalent Iodonium Reagent
Source: Chemistry. 2021 Oct 6;27(63):15638–43. doi: 10.1002/chem.202102840 (PMC9293340; doi:10.1002/chem.202102840)
Supplement: Supplementary file 1 — Supporting Information [file CHEM-27-15638-s001.pdf]

Chemistry–A European Journal

Supporting Information

Stereoselective Direct *N*-Trifluoropropenylation of Heterocycles with a Hypervalent Iodonium Reagent

János T. Csenki, Ádám Mészáros, Zsombor Gonda,* and Zoltán Novák*

Content

1. Materials and Methods 4

2. Optimization of reaction conditions 5

2.1. Optimalization of base 5

2.2. Optimalization of solvent 5

2.3. Optimization of required amount of iodonium salt 6

3. Reaction mechanism investigation 7

4. Synthesis of starting materials 13

(4-Fluorophenyl)(3,3,3-trifluoroprop-1-en-2-yl)iodonium trifluoromethanesulfonate (1) 13

N-Heterocycles (2) 14

5-Phenyl-3-(trifluoromethyl)-1*H*-pyrazole (2r) 14

Ethyl (*E*)-*N*-(4-cyano-1*H*-pyrazol-3-yl)acetimidate (2s) 15

5-Phenyl-1*H*-tetrazole (2x) 15

Ethyl 1*H*-indazole-3-carboxylate (2z) 16

5-((*tert*-Butyldimethylsilyl)oxy)-1*H*-indazole (2ac) 17

N-Deutero-1*H*-benzotriazole ([D]2a 17

5. General procedure 18

(*E*)-1-(3,3,3-Trifluoroprop-1-en-1-yl)-1*H*-benzo[d][1,2,3]triazole (3) 18

(*E*)-4-Bromo-1-(3,3,3-trifluoroprop-1-en-1-yl)-1*H*-pyrazole (4) 19

(*E*)-3,5-Dimethyl-1-(3,3,3-trifluoroprop-1-en-1-yl)-1*H*-pyrazole (5) 19

(*E*)-4-Iodo-3,5-dimethyl-1-(3,3,3-trifluoroprop-1-en-1-yl)-1*H*-pyrazole (6) 20

(*E*)-3,5-Diphenyl-1-(3,3,3-trifluoroprop-1-en-1-yl)-1*H*-pyrazole (7) 20

(*E*)-3,5-Di-p-tolyl-1-(3,3,3-trifluoroprop-1-en-1-yl)-1*H*-pyrazole (8) 21

(*E*)-3,5-Bis(4-chlorophenyl)-1-(3,3,3-trifluoroprop-1-en-1-yl)-1*H*-pyrazole (9) 22

(*E*)-3,5-Bis(4-bromophenyl)-1-(3,3,3-trifluoroprop-1-en-1-yl)-1*H*-pyrazole (10) 22

(*E*)-3,5-Bis(2-bromophenyl)-1-(3,3,3-trifluoroprop-1-en-1-yl)-1*H*-pyrazole (11) 23

(*E*)-3,5-Bis(4-methoxyphenyl)-1-(3,3,3-trifluoroprop-1-en-1-yl)-1*H*-pyrazole (12) 24

(*E*)-3,5-Bis(4-nitrophenyl)-1-(3,3,3-trifluoroprop-1-en-1-yl)-1*H*-pyrazole (13) 24

(*E*)-4-Iodo-3,5-diphenyl-1-(3,3,3-trifluoroprop-1-en-1-yl)-1*H*-pyrazole (14) 25

Ethyl (*E*)-3-(trifluoromethyl)-1-(3,3,3-trifluoroprop-1-en-1-yl)-1*H*-pyrazole-4-carboxylate (15) 26

(*E*)-3-Phenyl-1-(3,3,3-trifluoroprop-1-en-1-yl)-1*H*-pyrazole (16) 26

(*E*)-3-(4-Methoxyphenyl)-5-(4-nitrophenyl)-1/2-(3,3,3-trifluoroprop-1-en-1-yl)-1*H*-pyrazole (17, mixture of regioisomers) 27

(*E*)-3-Phenyl-5-(thiophen-2-yl)-1/2-(3,3,3-trifluoroprop-1-en-1-yl)-1*H*-pyrazole (18, mixture of regioisomers 18a, 18b) 28

(*E*)-5-(2-Tolyl)-3-(4-tolyl)-1-(3,3,3-trifluoroprop-1-en-1-yl)-1*H*-pyrazole (19, mixture of regioisomers 19a, 19b) 29

(*E*)-3-Phenyl-5-(trifluoromethyl)-1-(3,3,3-trifluoroprop-1-en-1-yl)-1*H*-pyrazole (20) & (*E*)-5-phenyl-3-(trifluoromethyl)-1-(3,3,3-trifluoroprop-1-en-1-yl)-1*H*-pyrazole (21) (regioisomers) 30

Ethyl (*Z*)-*N*-(4-cyano-1-((*E*)-3,3,3-trifluoroprop-1-en-1-yl)-1*H*-pyrazol-5-yl)acetimidate (22) & ethyl (*E*)-*N*-(4-cyano-1-((*E*)-3,3,3-trifluoroprop-1-en-1-yl)-1*H*-pyrazol-3-yl)acetimidate (23) (regioisomers) 32

(*E*)-4-Bromo-1-(3,3,3-trifluoroprop-1-en-1-yl)-1*H*-imidazole (24) 33

(*E*)-2-Ethyl-1-(3,3,3-trifluoroprop-1-en-1-yl)-1*H*-imidazole (25) 34

(*E*)-4,5-Diphenyl-1-(3,3,3-trifluoroprop-1-en-1-yl)-1*H*-imidazole (26) 34

Ethyl (*E*)-1-(3,3,3-trifluoroprop-1-en-1-yl)-1*H*-imidazole-4-carboxylate (27) 35

(*E*)-5-Phenyl-1-(3,3,3-trifluoroprop-1-en-1-yl)-1*H*-tetrazole (28) 36

(*E*)-2-(3,3,3-Trifluoroprop-1-en-1-yl)-2*H*-indazole (29) 36

Ethyl (*E*)-2-(3,3,3-trifluoroprop-1-en-1-yl)-2*H*-indazole-3-carboxylate (30) & ethyl (*E*)-1-(3,3,3-trifluoroprop-1-en-1-yl)-1*H*-indazole-3-carboxylate (31) 37

(*E*)-5-Bromo-1-(3,3,3-trifluoroprop-1-en-1-yl)-1*H*-pyrrolo[2,3-b]pyridine (32) 38

(*E*)-5-Nitro-1-(3,3,3-trifluoroprop-1-en-1-yl)-1*H*-indazole (33) 39

(*E*)-5-((tert-Butyldimethylsilyl)oxy)-1-(3,3,3-trifluoroprop-1-en-1-yl)-1*H*-indazole (34) 39

(*E*)-1-(3,3,3-Trifluoroprop-1-en-1-yl)-1*H*-benzo[d]imidazole (35) 40

(*E*)-5,6-Dimethyl-1-(3,3,3-trifluoroprop-1-en-1-yl)-1*H*-benzo[d]imidazole (36) 41

(*E*)-6-Bromo-1-(3,3,3-trifluoroprop-1-en-1-yl)-1*H*-indazole (37) 41

(*E*)-2-Chloro-3-methyl-1-(3,3,3-trifluoroprop-1-en-1-yl)-1*H*-pyrrolo[2,3-b]pyridine (38) 42

(*E*)-4-Nitro-2-(3,3,3-trifluoroprop-1-en-1-yl)-2*H*-benzo[*d*][1,2,3]triazole (39) & (*E*)-7-nitro-1-(3,3,3-trifluoroprop-1-en-1-yl)-1*H*-benzo[*d*][1,2,3]triazole (40) & (*E*)-4-nitro-1-(3,3,3-trifluoroprop-1-en-1-yl)-1*H*-benzo[d][1,2,3]triazole (41) 43

(*E*)-6-Chloro-9-(3,3,3-trifluoroprop-1-en-1-yl)-9*H*-purine (42) 45

(*E*)-6-Chloro-2-fluoro-9-(3,3,3-trifluoroprop-1-en-1-yl)-9*H*-purine (43) 45

(*E*)-2,6-Dichloro-9-(3,3,3-trifluoroprop-1-en-1-yl)-9*H*-purine (44) 46

(*E*)-2-(3,3,3-Trifluoroprop-1-en-1-yl)isoindoline-1,3-dione (45) 47

(*E*)-5,5-Diphenyl-3-(3,3,3-trifluoroprop-1-en-1-yl)imidazolidine-2,4-dione (46) 47

5-Phenyl-3-(trifluoromethyl)-1*H*-pyrazole (2r) 49

Ethyl (*E*)-*N*-(4-cyano-1*H*-pyrazol-3-yl)acetimidate (2s) 52

5-Phenyl-1*H*-tetrazole (2x) 54

Ethyl 1*H*-indazole-3-carboxylate (2z) 56

5-((tert-Butyldimethylsilyl)oxy)-1*H*-indazole (2ac) 58

N-deutero-1*H*-benzotriazole ([D]2a) 60

(*E*)-1-(3,3,3-Trifluoroprop-1-en-1-yl)-1*H*-benzo[d][1,2,3]triazole (3) 62

(*E*)-4-Bromo-1-(3,3,3-trifluoroprop-1-en-1-yl)-1*H*-pyrazole (4) 65

(*E*)-3,5-Dimethyl-1-(3,3,3-trifluoroprop-1-en-1-yl)-1*H*-pyrazole (5) 68

(*E*)-4-Iodo-3,5-dimethyl-1-(3,3,3-trifluoroprop-1-en-1-yl)-1*H*-pyrazole (6) 71

(*E*)-3,5-Diphenyl-1-(3,3,3-trifluoroprop-1-en-1-yl)-1*H*-pyrazole (7) 74

(*E*)-3,5-Di-4-tolyl-1-(3,3,3-trifluoroprop-1-en-1-yl)-1*H*-pyrazole (8) 77

(*E*)-3,5-Bis(4-chlorophenyl)-1-(3,3,3-trifluoroprop-1-en-1-yl)-1*H*-pyrazole (9) 80

(*E*)-3,5-Bis(4-bromophenyl)-1-(3,3,3-trifluoroprop-1-en-1-yl)-1*H*-pyrazole (10) 83

(*E*)-3,5-Bis(2-bromophenyl)-1-(3,3,3-trifluoroprop-1-en-1-yl)-1*H*-pyrazole (11) 86

(*E*)-3,5-Bis(4-methoxyphenyl)-1-(3,3,3-trifluoroprop-1-en-1-yl)-1*H*-pyrazole (12) 89

(*E*)-3,5-Bis(4-nitrophenyl)-1-(3,3,3-trifluoroprop-1-en-1-yl)-1*H*-pyrazole (13) 92

(*E*)-3,5-(E)-4-Iodo-3,5-diphenyl-1-(3,3,3-trifluoroprop-1-en-1-yl)-1*H*-pyrazole (14) 95

Ethyl (*E*)-3-(trifluoromethyl)-1-(3,3,3-trifluoroprop-1-en-1-yl)-1*H*-pyrazole-4-carboxylate (15) 98

(*E*)-3-phenyl-1-(3,3,3-trifluoroprop-1-en-1-yl)-1*H*-pyrazole (16) 101

(*E*)-3-(4-Methoxyphenyl)-5-(4-nitrophenyl)-1/2-(3,3,3-trifluoroprop-1-en-1-yl)-1*H*-pyrazole (17) mixture of regioisomers 104

(*E*)-3-Phenyl-5-(thiophen-2-yl)-1/2-(3,3,3-trifluoroprop-1-en-1-yl)-1*H*-pyrazole (18) mixture of regioisomers 107

(*E*)-3-(2-Tolyl)-5-(4-tolyl)-1-(3,3,3-trifluoroprop-1-en-1-yl)-1*H*-pyrazole (19) 110

(*E*)-3-Phenyl-5-(trifluoromethyl)-1-(3,3,3-trifluoroprop-1-en-1-yl)-1*H*-pyrazole (20) isomer1 113

(*E*)-5-Phenyl-3-(trifluoromethyl)-1-(3,3,3-trifluoroprop-1-en-1-yl)-1*H*-pyrazole (21) isomer2 116

Ethyl (*Z*)-*N*-(4-cyano-1-((*E*)-3,3,3-trifluoroprop-1-en-1-yl)-1*H*-pyrazol-5-yl)acetimidate (22) isomer1, ...119

Ethyl (*E*)-*N*-(4-cyano-1-((*E*)-3,3,3-trifluoroprop-1-en-1-yl)-1*H*-pyrazol-3-yl)acetimidate (23) isomer2 122

(*E*)-4-Bromo-1-(3,3,3-trifluoroprop-1-en-1-yl)-1*H*-imidazole (24) 125

(*E*)-2-Ethyl-1-(3,3,3-trifluoroprop-1-en-1-yl)-1*H*-imidazole (25) 128

(*E*)-4,5-Diphenyl-1-(3,3,3-trifluoroprop-1-en-1-yl)-1*H*-imidazole (26) 131

Ethyl (*E*)-1-(3,3,3-trifluoroprop-1-en-1-yl)-1*H*-imidazole-4-carboxylate (27) 134

(*E*)-5-Phenyl-1-(3,3,3-trifluoroprop-1-en-1-yl)-1*H*-tetrazole (28) 137
(*E*)-2-(3,3,3-Trifluoroprop-1-en-1-yl)-2*H*-indazole (29) 140
Ethyl (*E*)-2-(3,3,3-trifluoroprop-1-en-1-yl)-2*H*-indazole-3-carboxylate (30) izomer1 143
Ethyl (*E*)-1-(3,3,3-trifluoroprop-1-en-1-yl)-1*H*-indazole-3-carboxylate (31) izomer2 146
(*E*)-6-Bromo-1-(3,3,3-trifluoroprop-1-en-1-yl)-1*H*-indazole (37) 149
(*E*)-5-Nitro-1-(3,3,3-trifluoroprop-1-en-1-yl)-1*H*-indazole (33) 152
(*E*)-5-((*tert*-Butyldimethylsilyl)oxy)-1-(3,3,3-trifluoroprop-1-en-1-yl)-1*H*-indazole (34) 155
(*E*)-1-(3,3,3-Trifluoroprop-1-en-1-yl)-1*H*-benzo[d]imidazole (35) 158
(*E*)-5,6-Dimethyl-1-(3,3,3-trifluoroprop-1-en-1-yl)-1*H*-benzo[d]imidazole (36) 161
(*E*)-5-Bromo-1-(3,3,3-trifluoroprop-1-en-1-yl)-1*H*-pyrrolo[2,3-b]pyridine (37) 164
(*E*)-2-Chloro-3-methyl-1-(3,3,3-trifluoroprop-1-en-1-yl)-1*H*-pyrrolo[2,3-b]pyridine (38) 167
(*E*)-4-Nitro-1-(3,3,3-trifluoroprop-1-en-1-yl)-1*H*-benzo[d][1,2,3]triazole (39) 170
(*E*)-4-Nitro-1-(3,3,3-trifluoroprop-1-en-1-yl)-1*H*-benzo[d][1,2,3]triazole (40) 173
(*E*)-4-Nitro-1-(3,3,3-trifluoroprop-1-en-1-yl)-1*H*-benzo[d][1,2,3]triazole (41) 176
(*E*)-6-Chloro-9-(3,3,3-trifluoroprop-1-en-1-yl)-9*H*-purine (42) 179
(*E*)-6-Chloro-2-fluoro-9-(3,3,3-trifluoroprop-1-en-1-yl)-9*H*-purine (43) 182
(*E*)-2,6-Dichloro-9-(3,3,3-trifluoroprop-1-en-1-yl)-9*H*-purine (44) 185
(3,3,3-Trifluoroprop-1-en-1-yl)isoindoline-1,3-dione (45) 188
(*E*)-5,5-Diphenyl-3-(3,3,3-trifluoroprop-1-en-1-yl)imidazolidine-2,4-dione (46) 191

Materials and Methods

Analytical thin-layer chromatography (TLC) was performed on Merck DC pre-coated TLC plates with 0.25 mm Kieselgel 60 F254. Visualization was performed with a 254 nm UV lamp and $KMnO_4$ stain. The ^{1}H, ^{13}C and ^{19}F NMR spectra were recorded on a Bruker Avance-250, a Varian Inova 300, Bruker Avance-500 and Nanalysis 60 in $CDCl_3$ or DMSO-d_6. We used proton decoupled measurements for recording ^{19}F-NMR spectra. Chemical shifts are expressed in parts per million (δ) using residual solvent protons as internal standards (δ 7.26 in $CDCl_3$, 2.50 in DMSO-d_6 and 1.94 in CD_3CN for ^{1}H, δ 77.16 in $CDCl_3$, 39.52 in DMSO-d_6 and 1.32 in CD_3CN for ^{13}C). Coupling constants (J) are reported in Hertz (Hz). Splitting patterns are designated as s (singlet), bs (broad singlet), d (doublet), t (triplet), q (quartet), quin (quintet), sextet (sex), septet (sept) m (multiplet). Conversions determined by gas chromatography. Low resolution mass spectrometry was obtained on an Agilent 6890N Gas Chromatograph (30 m × 0.25mm column with 0.25 µm HP-5MS coating, He carrier gas) and Agilent 5973 Mass Spectrometer (Ion source: EI+, 70eV, 230 °C interface 300 °C). IR spectra were obtained from solids or as a thin film on a Mettler Toledo ReactIR™ 15, AgX DiComp probe, 6 mm x 1.5 m Fiber (Silver Halide), MCT detector. All melting points were measured on Büchi 501 apparatus and values are uncorrected. High-resolution mass spectra were acquired on an Agilent 6230 time-of-flight mass spectrometer equipped with a Jet Stream electrospray ion source in positive ion mode. Injections of 0.5µl were directed to the mass spectrometer at a flow rate 1.5 ml/min (5mM ammonium-formate in water and acetonitrile gradient program), using an Agilent 1290 Infinity HPLC system. Jet Stream parameters: drying gas (N_2) flow and temperature: 8.0 l/min and 325 °C, respectively; nebulizer gas (N_2) pressure: 30 psi; capillary voltage: 3000 V; sheath gas flow and temperature: 325 °C and 10.0 l/min; TOFMS parameters: fragmentor voltage: 100 V; skimmer potential: 60 V; OCT 1 RF Vpp:750 V. Full-scan mass spectra were acquired over the m/z range 105-1700 at an acquisition rate of 995.6 ms/spectrum and processed by Agilent MassHunter B.04.00 software.

1. Optimization of reaction conditions

2.1. Optimalization of base

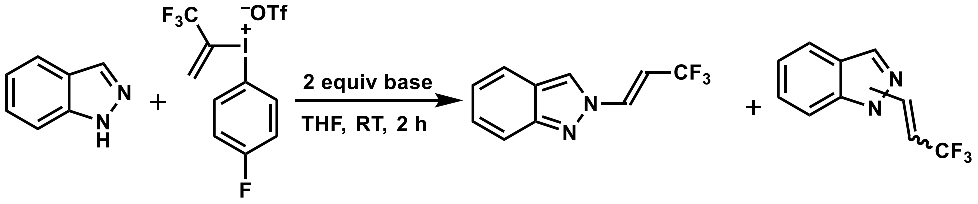

The base (0.10 mmol, 2.0 equiv), 5.9 mg 1*H*-indazole (0.05 mmol, 1.0 equiv), 28 mg (4-fluorophenyl)(3,3,3-trifluoroprop-1-en-2-yl)iodonium triflate (0.06 mmol, 1.2 equiv) and 0.5 mL of THF were added to a 4 mL screw capped vial with a magnetic stir bar, and the mixture was stirred at room temperature for 2 hours.

Entry	Base	Conversion*				
		29 / %	29a / %	29b / %	29c / %	indazole/ %
**1**	**Li_2CO_3**	**83**	12	4	1	0
**2**	**Na_2CO_3**	**80**	14	6.5	0.5	0
**3**	**K_2CO_3**	**59**	22	10	2	7
**4**	**Cs_2CO_3**	**69**	20	7	2	2
**5**	**NaH**	**75**	18	6	1	<0.3
**6**	**NaOH**	**78**	18	4	1	0
**7**	**K_3PO_4**	**71**	20	8	1	0
**8**	**Collidine**	**84**	10	1	<0.1	0
**9**	**DIPEA**	**76**	16	4	1	3

*GC-MS conversion

2.2. Optimalization of solvent

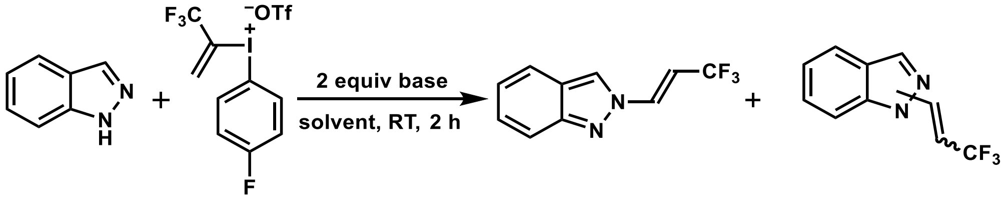

5.9 mg 1*H*-indazole (0.05 mmol, 1.0 equiv), base (0.10 mmol, 2.0 equiv), 28 mg (4-fluorophenyl)(3,3,3-trifluoroprop-1-en-2-yl)iodonium triflate (0.06 mmol, 1.2 equiv) and 0.5 mL of solvent were added to a 4 mL screw capped vial with a magnetic stir bar, and the mixture was stirred at room temperature for 2 hours.

Entry	Base	Solvent	Conversion*				
			29 / %	29a / %	29b / %	29c / %	indazole / %
**1**	**Li_2CO_3**	PhMe	**88**	8	3	1	0
**2**		Et_2O	**85**	8	6	1	0
**3**		DCM	**89**	6	4	<1	0
**4**		EtOAc	**80**	10	10	<1	0
**5**		DMF	**78**	21	1	<0.1	0
**6**		MeCN	**93**	2	5	-	0
**7**	**Collidine**	PhMe	**85**	5	1	-	9
**8**		Et_2O	**72**	4	1	-	23
**9**		DCM	**72**	5	1	-	22
**10**		EtOAc	**80**	8	1	-	11
**11**		DMF	**78**	12	1	-	9
**12**		MeCN	**69**	3	1	-	27

*GC-MS conversion

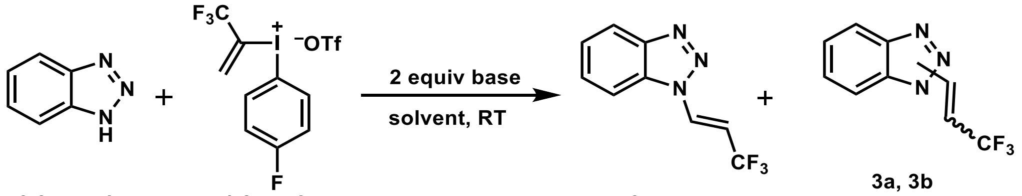

0.3 mmol 1.2 equiv 3 3a, 3b

36.0 mg 1*H*-benzotriazole (0.3 mmol, 1.0 equiv), base (0.6 mmol, 2.0 equiv), 28 mg (4-fluorophenyl)(3,3,3-trifluoroprop-1-en-2-yl)iodonium triflate (0.36 mmol, 1.2 equiv) and 3 mL of solvent were added to a 4 mL screw capped vial with a magnetic stir bar, and the mixture was stirred at room temperature for 2 hours. The solvent was evaporated under reduced pressure and the crude product was purified by column chromatography using hexanes:ethyl acetate eluent gradient.

Entry	Base	Solvent	Conversion* (Isolated Yield)		
			3 / %	3a / %	3b / %
**1**	**K_3PO_4**	THF	**44**	7	-
**2**		MeCN	**55**	6	3
**3**	**Li_2CO_3**	THF	**85 (76)**	4	6
**4**		MeCN	**96 (95)**	2	-
**5**		DCM	**83**		
**6**		EtOAc	**80**		
**7**	**Na_2CO_3**	THF	**80 (73)**	5	-
**8**		MeCN	**83 (76)**	7	-
**9**		DCM	**80**		
**10**	**K_2CO_3**	DCM	**60**		
**11**	**NaH**	DCM	**75**		
**12**	**Collidine**	MeCN	**66**		
**13**		DCM	**85**		

*GC-MS conversion

2.3. Optimization of required amount of iodonium salt

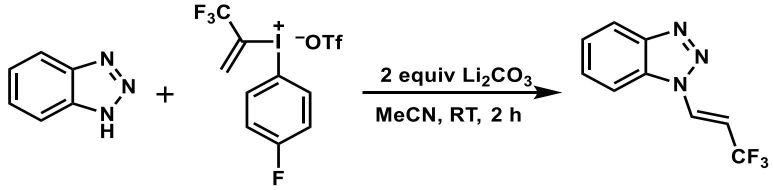

0.3 mmol 1.2 equiv 3

36.0 mg 1*H*-benzotriazole (0.3 mmol, 1.0 equiv), 44.4 mg Li_2CO_3 (0.6 mmol, 2.0 equiv), (4-fluorophenyl)(3,3,3-trifluoroprop-1-en-2-yl)iodonium triflate (0.3-0.45 mmol, 1.0-1.5 equiv) and 3 mL of MeCN were added to a 4 mL screw capped vial with a magnetic stir bar, and the mixture was stirred at room temperature for 2 hours. The solvent was evaporated under reduced pressure and the crude product was purified by column chromatography using hexanes:ethyl acetate eluent gradient.

Entry	Iodonium salt / equiv	Yield / %
**1**	1.0	86
**2**	1.1	**91**
**3**	1.2	90
**4**	1.3	90
**5**	1.5	90

*Isolated yield

3. Reaction mechanism investigation

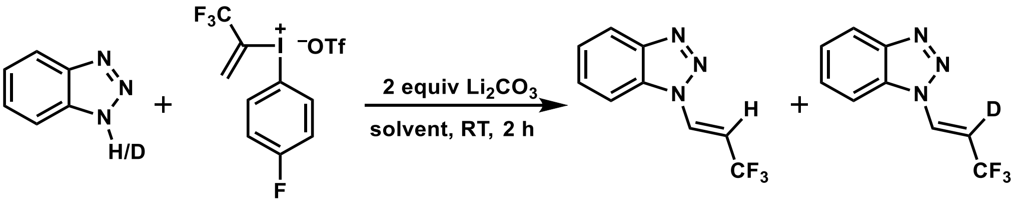

1*H*-Benzotriazole or 1*D*-benzotriazole (0.3-1.0 mmol, 1.0 equiv), Li_2CO_3 (0.6-2.0 mmol, 2.0 equiv), (4-fluorophenyl)(3,3,3-trifluoroprop-1-en-2-yl)iodonium triflate (0.36-1.2 mmol, 1.2 equiv) and 3-10 mL of MeCN were added to a 4-20 mL screw capped vial with a magnetic stir bar, and the mixture was stirred at room temperature for 2 hours. The solvent was evaporated under reduced pressure and the crude product was purified by column chromatography using hexanes:ethyl acetate eluent gradient.

Entry	Benzotriazole	Solvent	D_2O equiv	Yield* / %	3/3D ratio
**1**	**2a**	MeCN	0	95	100:0
**2**	**2a**	deutero-MeCN	0	87	100:0
**3**	**[D]2a**	MeCN	0	92<br>94<br>average:<br>93%	89:11<br>72:28**<br>average:<br>81:19
**4**	**[D]2a**	deutero-MeCN	0	92	76:24
**5**	**[D]2a**	MeCN	1	85<br>95<br>average:90%	46:54<br>48:52**<br>average:<br>47:53
**6**	**[D]2a**	deutero-MeCN	1	81	60:40
**7**	**2a**	MeCN	1	95	75:25

*Isolated yield, **Second run

The measured deuterium incorporation in the product:

The ^{19}F NMR spectras of the reactions with both [1*H*] and [1*D*]-benzotriazole (**2a** and **[D]2a**) in MeCN and d_3-MeCN. ^{19}F NMR (282 MHz, CD_3CN)

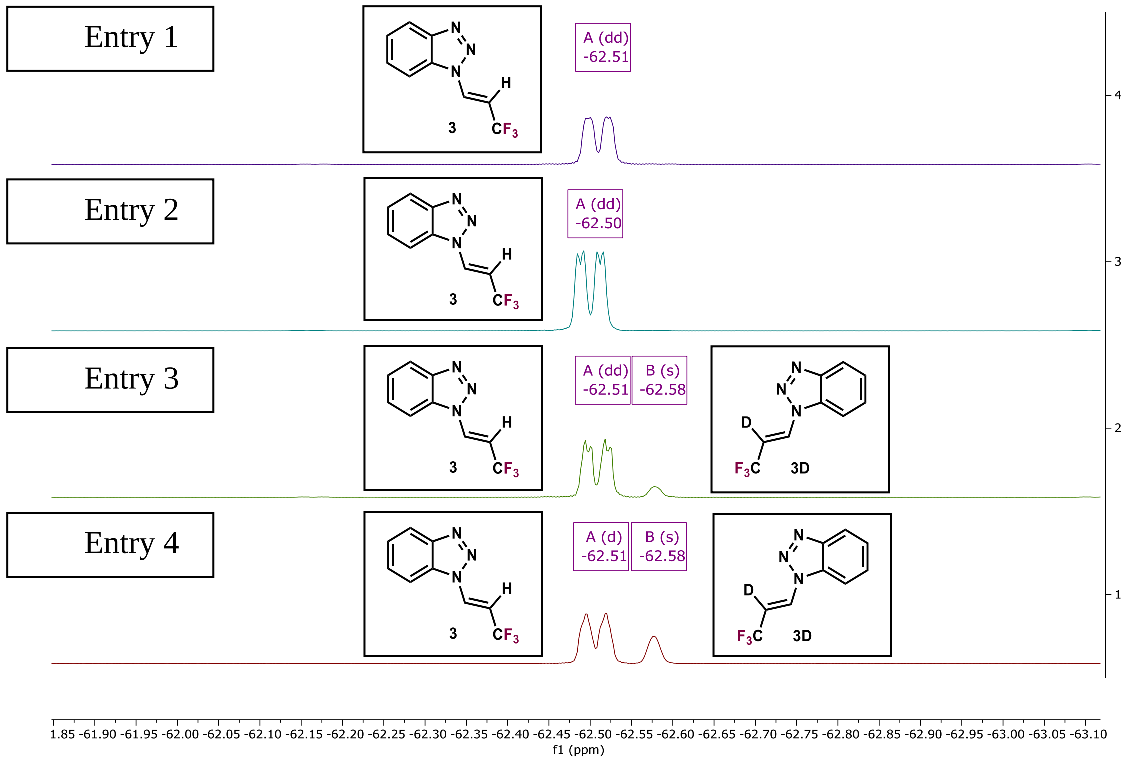

The ^{19}F NMR spectras of the reactions with both [1*H*] and [1*D*]-benzotriazole (**2a** and **[D]2a**) in MeCN and d_3-MeCN with 1-1 equiv D_2O. ^{19}F NMR (376 MHz, CD_3CN)

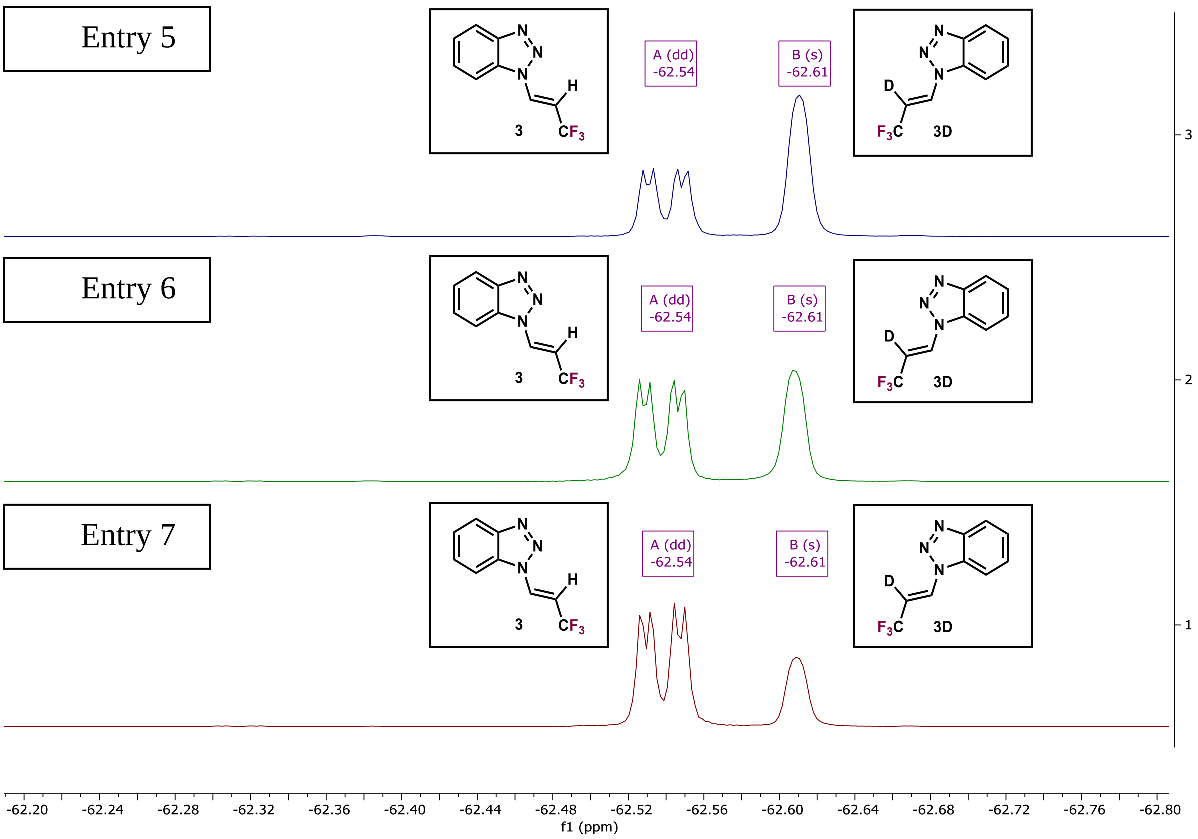

The [1*H*] substrate **2a** gave product **3** in CH_3CN. ^{1}H NMR (300 MHz, CD_3CN)

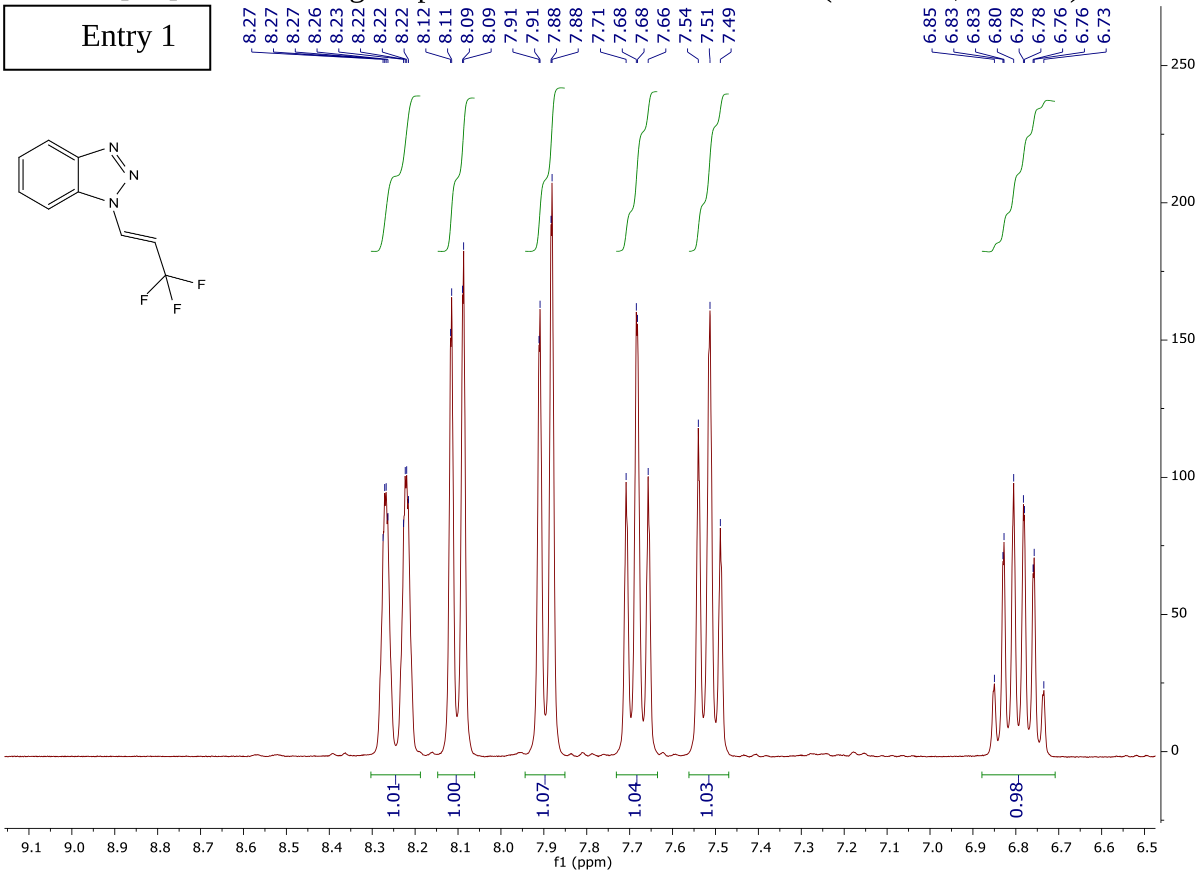

The [1*H*] substrate **2a** gave product **3** in CD_3CN. ^{1}H NMR (300 MHz, CD_3CN)

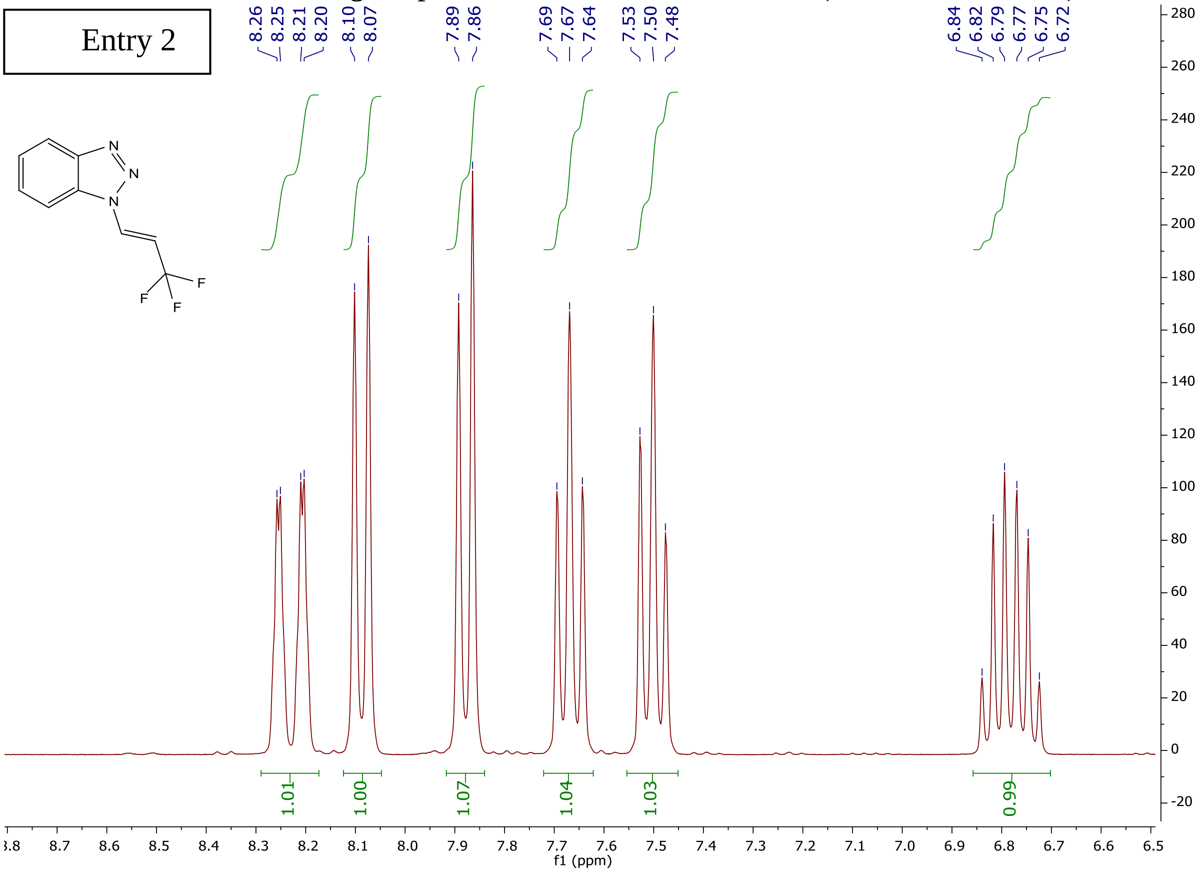

The [1*D*] substrate **[D]2a** gave product **3** and **3D** in CH_3CN. ^1H NMR (300 MHz, CD_3CN)

Entry 3

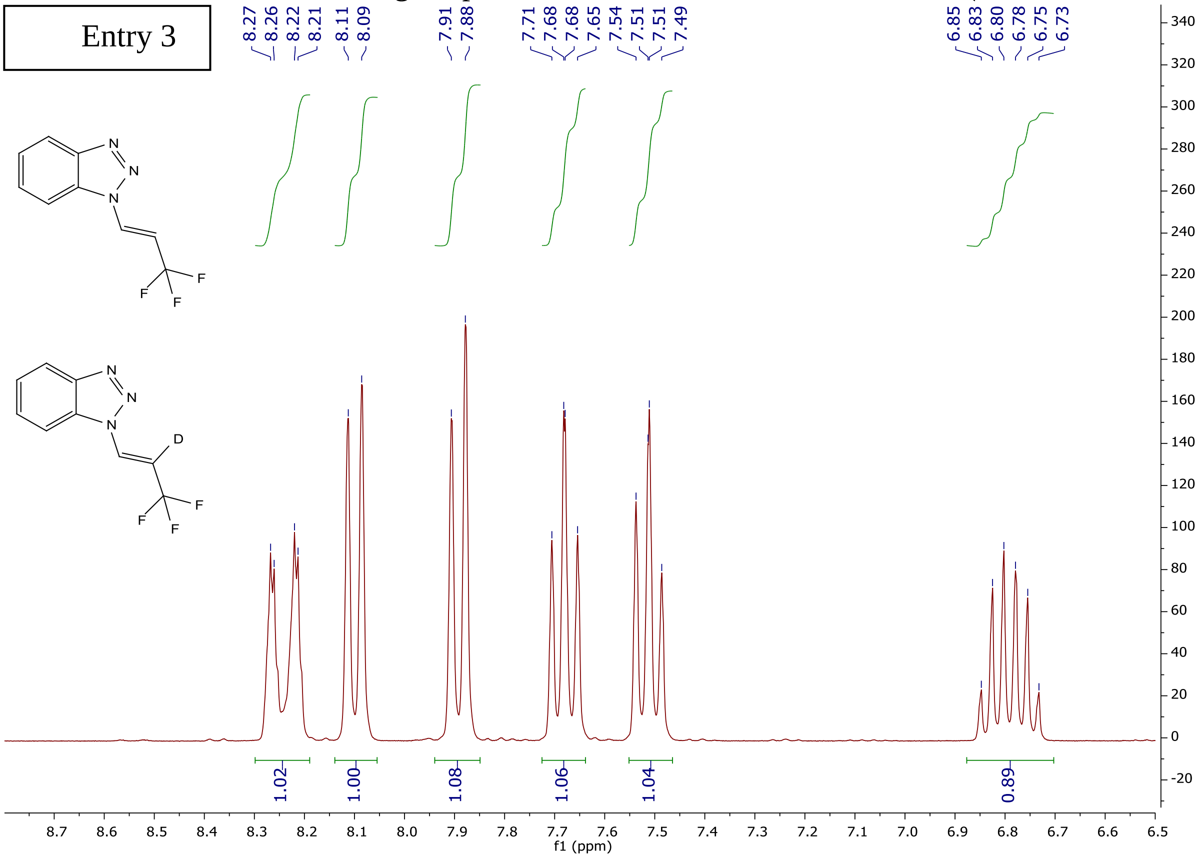

The [1*D*] substrate **[D]2a** gave product **3** and **3D** in CD_3CN. ^1H NMR (300 MHz, CD_3CN)

Entry 4

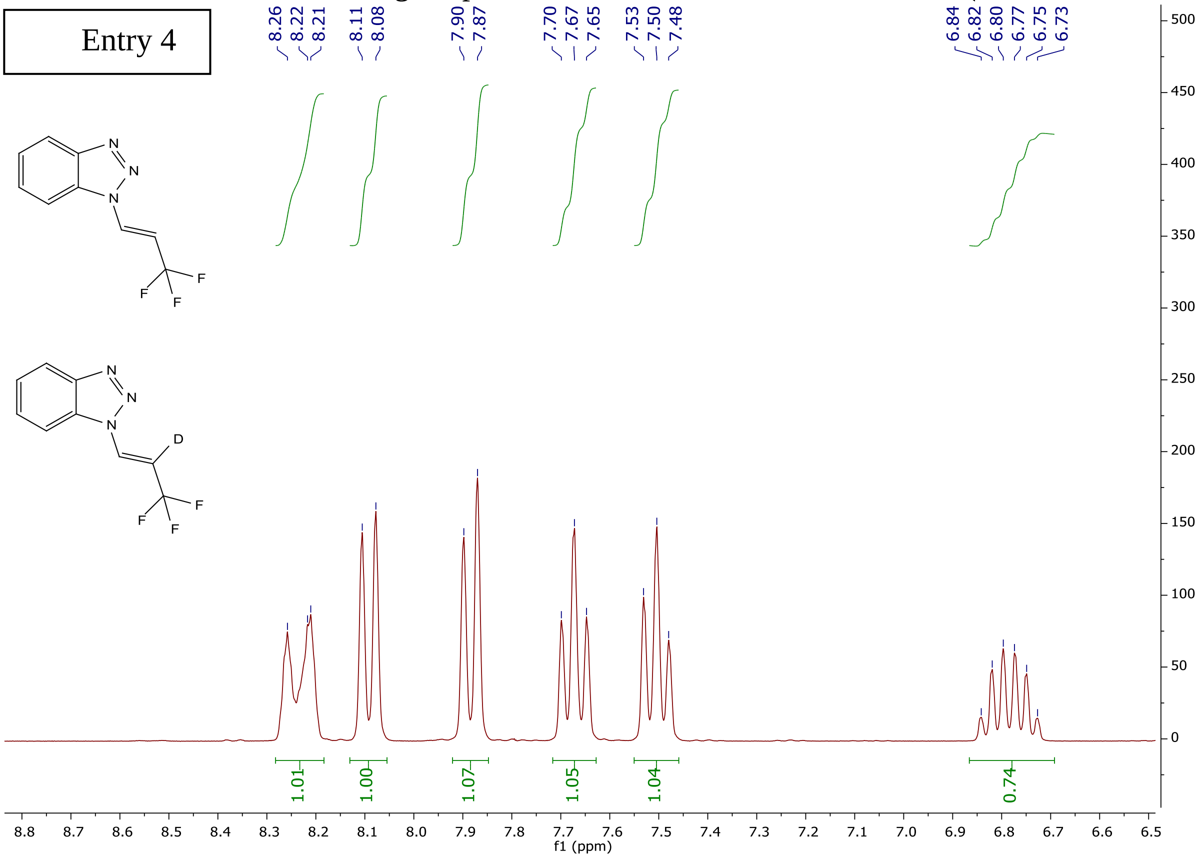

The [1*D*] substrate **[D]2a** gave product **3** and **3D** in CH_3CN+1 equiv D_2O. ^{1}H NMR (400 MHz, CD_3CN)

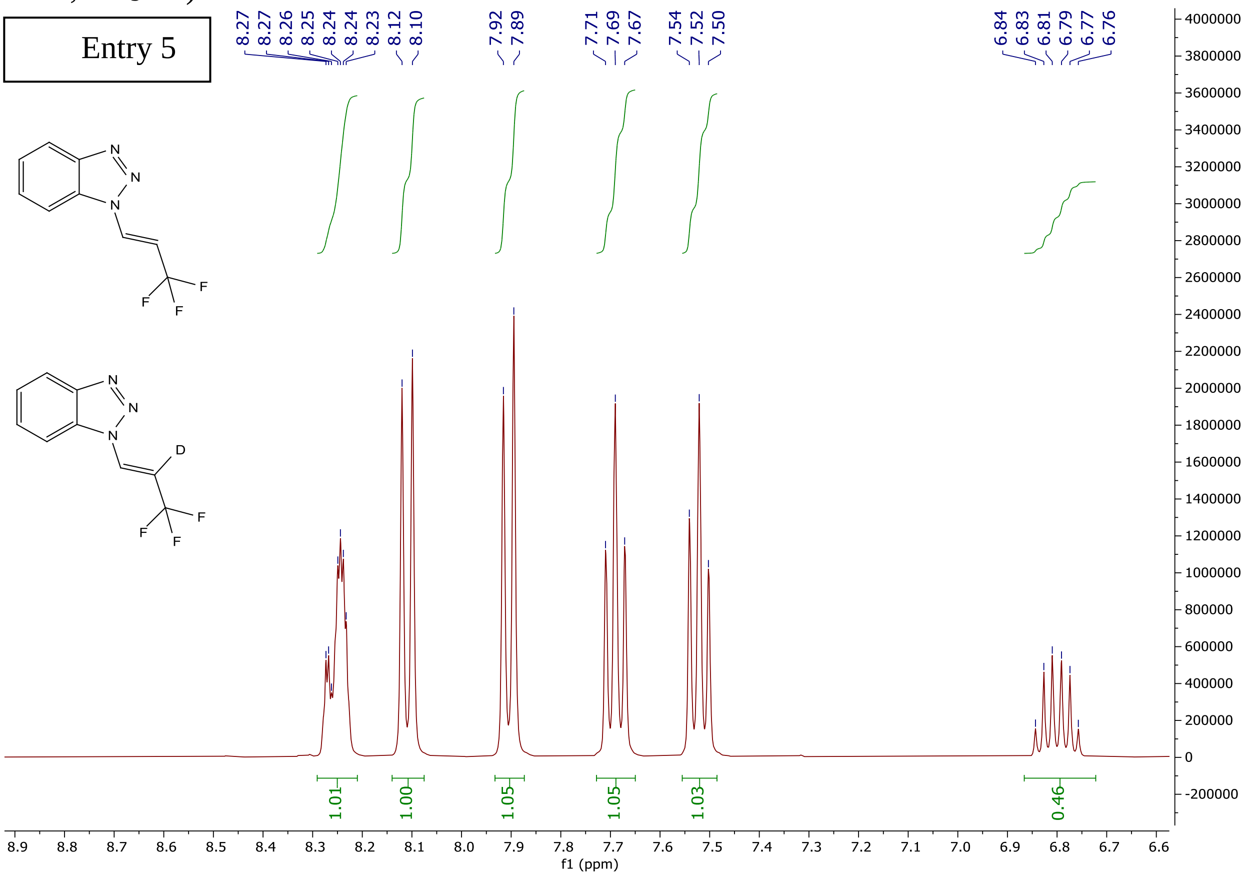

The [1*D*] substrate **[D]2a** gave product **3** and **3D** in CD_3CN+1 equiv D_2O. ^{1}H NMR (400 MHz, CD_3CN)

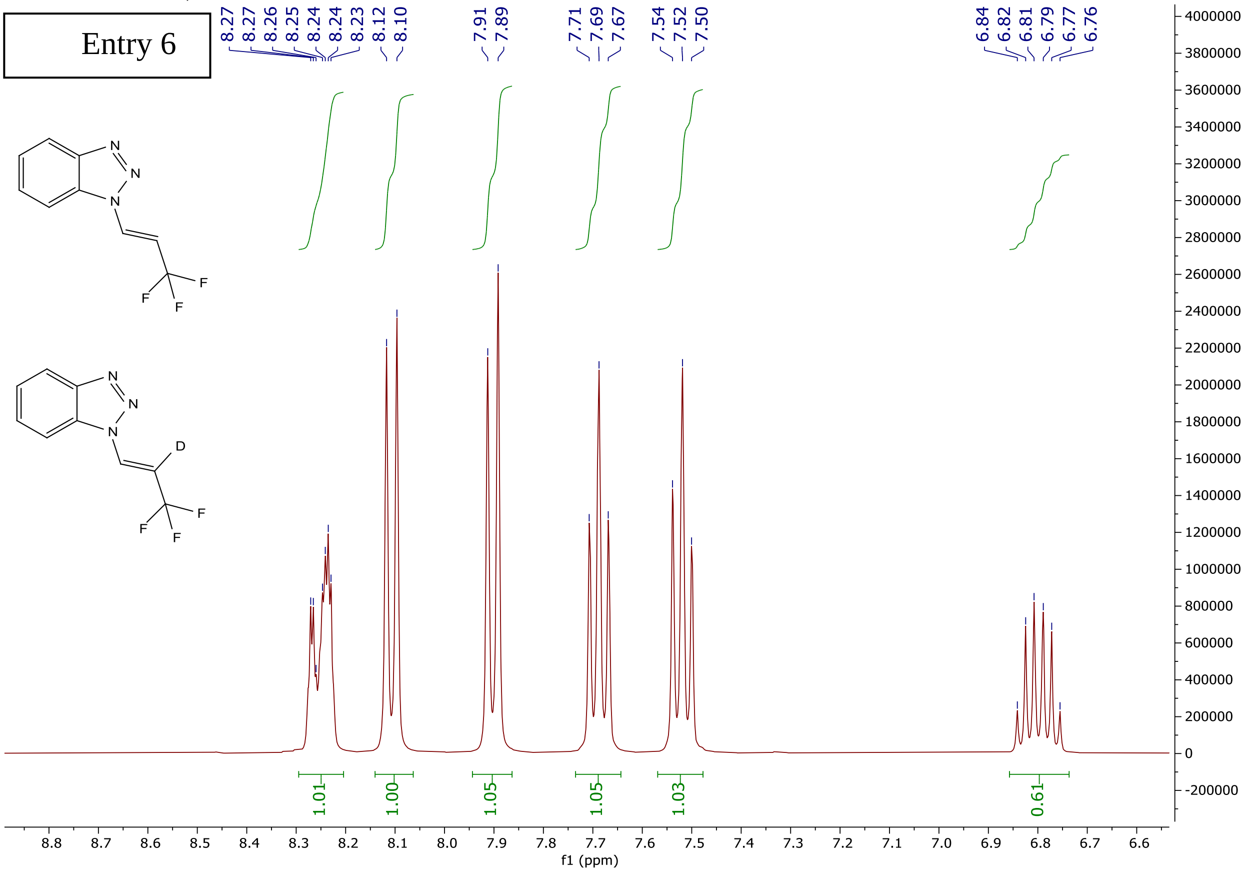

The [1*H*] substrate **2a** gave product **3** and **3D** in CD_3CN+1 equiv D_2O. ^1H NMR (400 MHz, CD_3CN)

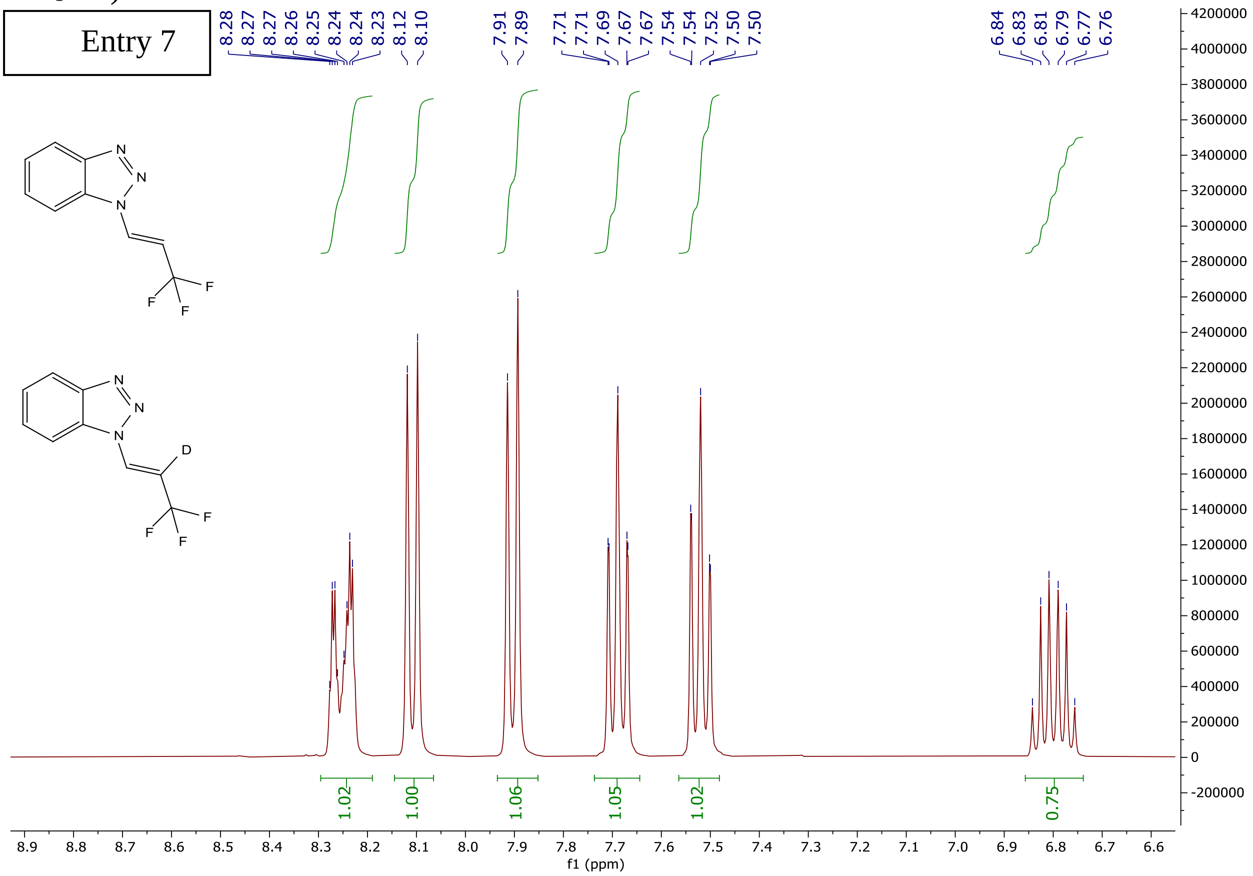

4. Synthesis of starting materials

(4-Fluorophenyl)(3,3,3-trifluoroprop-1-en-2-yl)iodonium trifluoromethanesulfonate (1)[1]

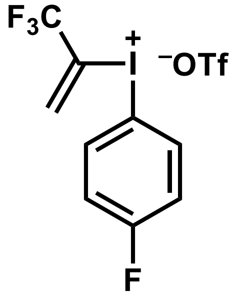

A 40 mL screwed cap vial with a stirring bar was evacuated and refilled with argon 3 times. Trifluoroacetic anhydride (10 mL, 70.5 mmol, 7.2 equiv) and trifluoroacetic acid (77 µL, 1 mmol, 10 mol%) was added via syringe. The mixture was cooled to 0 °C, then hydrogen peroxide (980 µL, 50 wt% in water, 17 mmol, 1.7 equiv) was added dropwise within 2 minutes. 2-Iodo-3,3,3-trifluoropropene (1080 µL, 10 mmol) was added dropwise via syringe. The off-white suspension was kept between 0 °C and 4 °C for 16 hours.

After that, the mixture was cooled to -20 °C, then HPLC grade dichloromethane (10 mL) was added slowly. Fluorobenzene (1.45 mL, 15.5 mmol, 1.55 equiv) was added dropwise to the suspension, followed by the addition of trifluoromethanesulfonic acid (880 µL, 10 mmol, 1 equiv) to form an emerald green solution. Consider that the temperature is not allowed to reach more than -20 °C throughout the addition of triflic acid! The reaction mixture was kept between 0 °C and 4 °C for 6 hours. After that, all volatiles were removed under reduced pressure at 0 °C, protected from light. The brownish green oil was shaken with cold diethyl ether (20 mL, -20 °C), getting white precipitate. The suspension was kept at -20°C for 12 hours, then the white precipitate was filtered and washed with cold ether 3 times.

The product was obtained as a white solid (4.37 g, 9.76 mmol, 97.6% yield). Mp. 144-147 °C.

^{1}H NMR (250 MHz, CD_3CN) δ = 8.20 (dd, J = 7.8, 4.2 Hz, 2H), 7.51 – 7.22 (m, 4H) ppm.

^{13}C NMR (63 MHz, CD_3CN) δ = 166.4 (d, J = 254.6 Hz), 145.1 (q, J = 4.7 Hz), 139.9 (d, J = 9.5 Hz), 121.9 (q, J = 321.0 Hz), 121.2 (q, J = 273.8 Hz), 111.8 (q, J = 39.8 Hz), 108.3 (d, J = 3.2 Hz) ppm.

^{19}F NMR (235 MHz, CD_3CN) δ = -64.53 (3F), -79.40 (3F), -105.44 (1F) ppm.

IR (ATR) ν = 3094, 3063, 3026, 2973, 2929, 2873, 1598, 1502, 1449, 1353, 1299, 1262, 1232, 1188, 1161, 1141, 1103, 1055, 1038, 973, 913, 888, 851, 795, 744, 692, 615, 579, 522, 468, 413 cm^{-1}.

N-Heterocycles (2)

Unless otherwise indicated, starting materials were obtained from commercial suppliers, and were used without further purification. (**2a-2e**, **2m**, **2n**, **2t-2w**, **2y**, **2aa-2ab**, **2ad-2am**)

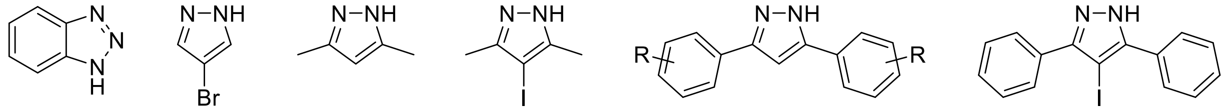

2a **2b** **2c** **2d** R = H **2e** **2l**

4-Me **2f**
4-Cl **2g**
4-Br **2h**
2-Br **2i**
4-OMe **2j**
4-NO_2 **2k**

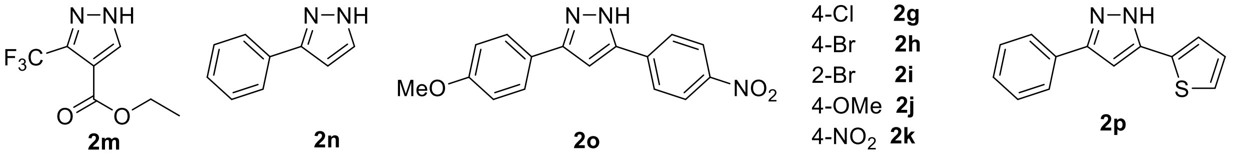

2m **2n** **2o** **2p**

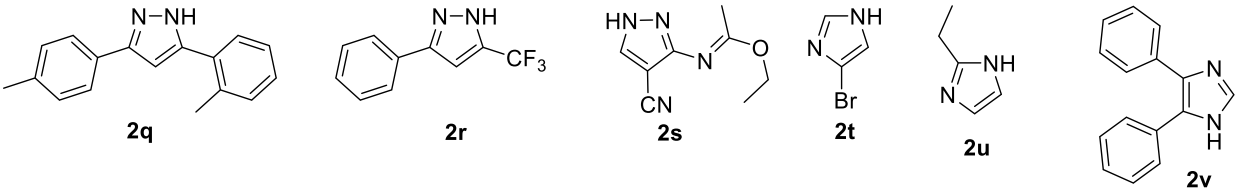

2q **2r** **2s** **2t** **2u** **2v**

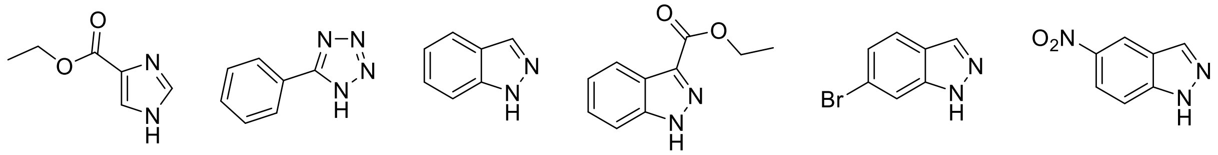

2w **2x** **2y** **2z** **2aa** **2ab**

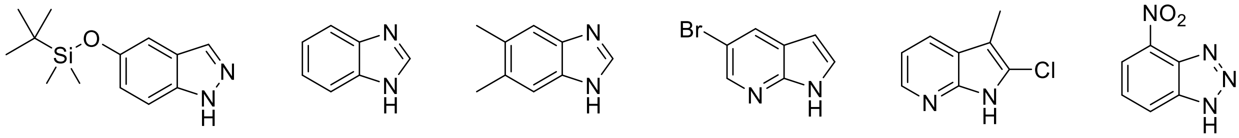

2ac **2ad** **2ae** **2af** **2ag** **2ah**

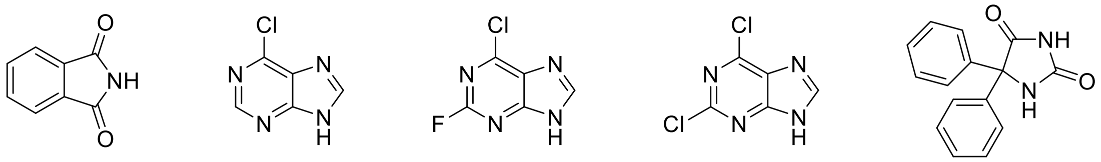

2ai **2aj** **2ak** **2al** **2am**

2f-2l, **2p** and **2q** prepared according to procedure given in the literature.[2]

5-Phenyl-3-(trifluoromethyl)-1*H*-pyrazole (2r)[3]

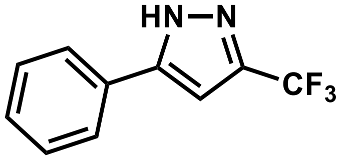

695,2 mg Ag_2O (3 mmol, 2.0 equiv), 246,1 mg NaOAc (3 mmol, 2.0 equiv), 165 μL phenylacetylene (1.5 mmol, 1.0 equiv) and 20 mL of DMF were added to a 50-mL Schlenk equipped with a stir bar, and the mixture was stirred vigorously at room temperature. To another 25-mL Schlenk charged with 813.1 mg $CF_3CH_2NH_2$·HCl (6 mmol, 3.0 equiv) and 517.5 mg $NaNO_2$ (7.5 mmol, 2.5 equiv) was added 5.5 mL of toluene and 273 μL of distilled H_2O, and the

mixture was stirred at 0 °C for 1 h. Anhydrous Na_2CO_3 (~200 mg) and anhydrous $MgSO_4$ (~820 mg) were added to the in situ prepared CF_3CHN_2 solution and stirred at 0 °C for 1 min, then the supernatant clear yellow solution was transferred via syringe to the first Schlenk containing the in situ generated silver(I) acetylides. The resulting mixture was stirred vigorously at 45 °C for 5 h. After completion of the reaction, the mixture was quenched with distilled water (30 mL), and ethyl acetate (30 mL) was added, the aqueous phase was extracted with ethyl acetate (2 × 20 mL). The organic layers were combined, dried over sodium sulfate and then concentrated in vacuo. The pure product was obtained by flash column chromatography using hexanes:ethyl acetate eluent gradient.

The product was obtained as off-white solid (269.3 mg, 1.27 mmol, 85%). Mp. 132-134 °C, R_f = 0.62 (hexanes:EtOAc = 2:1).

MS (EI, 70 eV): m/z (%): 212 (100, [M^+]), 193 (13), 164 (29), 143 (29), 133(13), 115 (14), 77 (15).

^{1}H NMR (250 MHz, $CDCl_3$) δ = 12.41 (s, 1H), 7.70 – 7.49 (m, 2H), 7.49 – 7.36 (m, 3H), 6.66 (s, 1H) ppm.

^{13}C NMR (63 MHz, $CDCl_3$) δ = 145.4, 143.5 (q, *J* = 38.2 Hz), 129.5, 129.3, 127.9, 125.7, 121.2 (q, *J* = 268.8 Hz), 101.0 ppm.

^{19}F NMR (235 MHz, $CDCl_3$) δ = -62.07 ppm.

Ethyl (*E*)-*N*-(4-cyano-1*H*-pyrazol-3-yl)acetimidate (2s)[4]

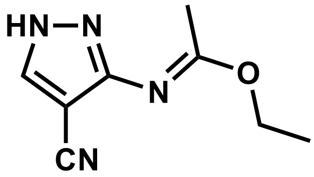

A solution of 3-amino-1*H*-pyrazole-4-carbonitrile 216.2 mg (2.00 mmol, 1.0 equiv), 1,1,1-triehoxyethane 440 μL (2.4 mmol, 1.2 equiv) and AcOH 1 drop in 15 ml MeCN was refluxed for 20 h. The resulting mixture was cooled down to room temperature, evaporated under vacuo. The residue was purified with column chromatography using hexanes:ethyl acetate eluent gradient.

The product was obtained as yellow oil (200.4 mg, 1.12 mmol, 56%). R_f = 0.65 (EtOAc).

MS (EI, 70 eV): m/z (%): 178 (38, [M^+]), 158 (11), 135 (100), 108 (63), 64 (15).

^{1}H NMR (300 MHz, $CDCl_3$) δ = 10.82 (s, 1H), 7.83 (s, 1H), 4.31 (t, *J* = 7.1 Hz, 2H), 2.10 (d, *J* = 2.6 Hz, 3H), 1.39 (t, *J* = 7.1 Hz, 3H) ppm.

^{13}C NMR (75 MHz, $CDCl_3$) δ = 167.7, 155.6, 138.7, 113.9, 83.8, 63.4, 17.8, 14.0 ppm.

5-Phenyl-1*H*-tetrazole (2x)[5]

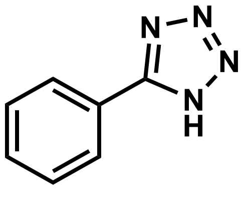

160.5 mg NH_4Cl (3.00 mmol, 1.0 equiv) and 234.0 mg NaN_3 (3.00 mmol, 3.0 equiv) were dissolved in DMF at room temperature. 306 μL benzonitrile (3.00 mmol, 1.0 equiv, d = 1.01 g/cm^3) was added, then heated to 110 °C for 24 hours. White precipitate formed. The mixture was allowed to cool to room temperature, then quenched with 1.2 mL 5 mol/dm^3 NaOH aqueous solution and stirred for 30 mins. The solvents were removed under reduced pressure. The crude mixture was dissolved in 3.0 mL distilled water, then 3.0 mL 3 mol/dm^3 HCl aqueous solution was added. White precipitate formed, which was filtered and washed with 2 × 3.0 mL 3 mol/dm^3 HCl aqueous solution. Dried under reduced pressure.

The product was obtained as white solid (388.2 mg, 2.66 mmol, 89%). Mp. >200 °C, R_f = 0.14 (EtOAc).

MS (EI, 70 eV): m/z (%): 146 (10, [M^+]), 118 (100), 103 (18), 91 (50), 77 (34).

^{1}H NMR (250 MHz, DMSO-d$_6$) δ = 8.10 – 8.00 (m, 2H), 7.59 (dd, *J* = 5.0, 2.0 Hz, 3H) ppm.

^{13}C NMR (63 MHz, DMSO-d$_6$) δ = 155.3, 131.3, 129.4, 127.0, 124.2 ppm.

Ethyl 1*H*-indazole-3-carboxylate (2z)[6]

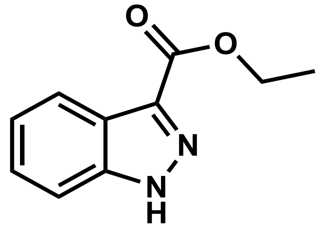

810.7 mg 1H-indazole-3-carboxylic acid (5.00 mmol, 1.0 equiv) was dissolved in 30 mL ethanol under argon atmosphere, then 2 drops of cc. H2SO4 was added. The mixture was stirred at 80 °C for 24 hours. Concentrated to circa 1/3 of starting volume under reduced pressure, then diluted with 50 mL ethyl acetate. The organic layer was washed with 10 mL distilled water, then with 20 mL brine. Dried over Na_2SO_4, then after filtration, the solvent was evaporated under reduced pressure. The crude product was purified by column chromatography using hexanes:ethyl acetate eluent gradient.

The product was obtained as a white crystal (876.4 mg, 4.61 mmol, 93%). Mp. 133-135 °C. R_f = 0.27 (hexanes:EtOAc = 2:1).

MS (EI, 70 eV): m/z (%): 190 (76, [M^+]), 162 (26), 145 (100), 118 (97), 90 (61), 88 (41).

^{1}H NMR (250 MHz, DMSO-*d*$_6$) δ = 13.94 (s, 1H), 8.08 (d, *J* = 8.1 Hz, 1H), 7.67 (d, *J* = 8.3 Hz, 1H), 7.44 (t, *J* = 7.6 Hz, 1H), 7.30 (t, *J* = 7.6 Hz, 1H), 4.38 (q, *J* = 7.1 Hz, 2H), 1.36 (t, *J* = 7.1 Hz, 3H) ppm.

^{13}C NMR (63 MHz, DMSO-*d*$_6$) δ = 162.4, 141.0, 135.2, 126.7, 122.8, 122.2, 121.1, 111.1, 60.3, 14.3 ppm.

The spectral data are in agreement with data given in the literature.[6]

5-((*tert*-Butyldimethylsilyl)oxy)-1*H*-indazole (2ac)[7]

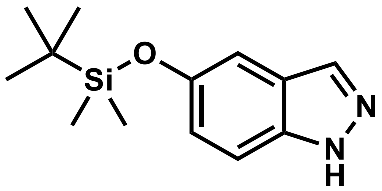

Prepared according to procedure given in the literature.

402.4 mg 5-hidroxyindazole (3.0 mmol, 1.0 equiv) was dissolved in 9 mL dry DMF (3 mL/1 mmol starting material) at room temperature, then 245.1 mg imidazole (3.6 mmol, 1.2 equiv) and 497.4 mg tert-butylchlorodimethylsilane (3.3 mmol, 1.1 equiv) was added carefully. The mixture was stirred at ambient temperature for 12 hours. Monitored by TLC, using hexanes-ethyl acetate as eluent. After the disappearance of starting material, the reaction mixture was quenched with 50 mL distilled water, then extracted with 20 mL diethyl ether 2 times. The combined organic layer was washed with 30 mL distilled water, then with 40 mL brine. The solution was dried over Na_2SO_4, then after filtration the solvent was evaporated under reduced pressure. The crude product was purified by column chromatography using hexanes:ethyl acetate eluent gradient.

The product was obtained as white solid (648.7 mg, 2.61 mmol, 87%). Mp. 90-94 °C. R_f = 0.25 (hexanes:EtOAc = 4:1).

MS (EI, 70 eV): m/z (%): 248 (35, [M^+]), 191 (100), 164 (8), 135 (10).

^{1}H NMR (250 MHz, DMSO-d_6) δ =12.94 (s, 1H), 7.92 (d, *J* = 1.5 Hz, 1H), 7.42 (d, *J* = 9.0 Hz, 1H), 7.12 (s, 1H), 6.91 (dd, *J* = 8.9, 2.2 Hz, 1H), 0.95 (s, 9H), 0.16 (s, 6H) ppm.

^{13}C NMR (63 MHz, DMSO-d_6) δ = 148.8, 136.1, 132.8, 123.4, 121.1, 110.8, 108.0, 25.6, 17.9, -4.6 ppm.

***N*-Deutero-1*H*-benzotriazole ([D]2a)**[8]

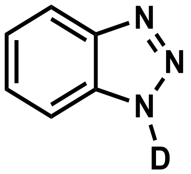

297.8 mg 1*H*-benzotriazole (2.50 mmol, 1.0 equiv) was dissolved in 1.5 mL deuteromethanol-d_4 at room temperature, then 0.25 mL D_2O was added. The mixture was stirred at 70 °C for 18 hours. Concentrated under reduced pressure The product was obtained as white solid (300.2 mg, 2.5 mmol, 100%).

MS (EI, 70 eV): m/z (%): 120 (12, [M^+]), 119 (100), 91 (83).

^{1}H NMR (300 MHz, CD_3CN) δ = 7.88 (dt, *J* = 5.7, 2.8 Hz, 2H), 7.43 (dt, *J* = 6.4, 3.1 Hz, 2H) ppm.

^{13}C NMR (75 MHz, CD_3CN) δ = 105.2, 96.9, 94.3 ppm.

5. General procedure

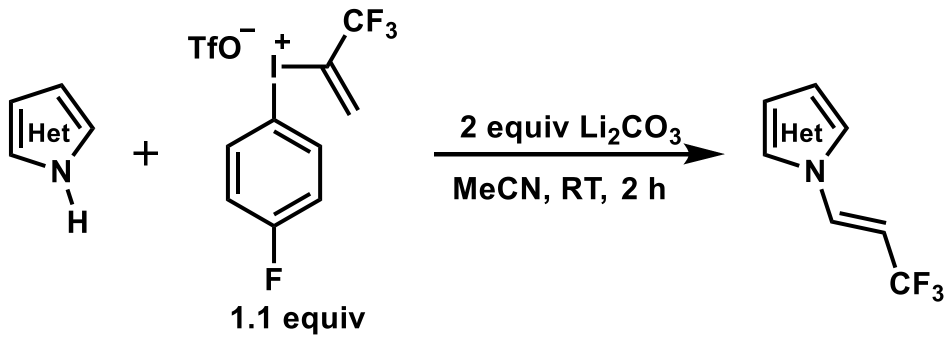

The appropriate N-H heterocycles (0.30-1.00 mmol, 1.0 equiv), Li_2CO_3 (0.60-2.00 mmol, 2.0 equiv), trifluoroisopropenyl iodonium salt (0.33-1.10 mmol, 1.1 equiv) and MeCN (0.1 M) were added to a 4 or 20 mL screw capped vial with a magnetic stir bar, and the mixture was stirred at room temperature for 2 hours. The solvent was evaporated under reduced pressure and the crude product was purified by column chromatography using hexanes:ethyl acetate eluent gradient.

(*E*)-1-(3,3,3-Trifluoroprop-1-en-1-yl)-1*H*-benzo[d][1,2,3]triazole (3)

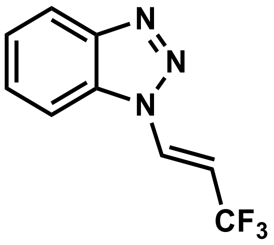

General procedure was followed using 35.7 mg (0.30 mmol) 1*H*-benzo[d][1,2,3]triazole, 44.3 mg (0.60 mmol) Li_2CO_3 and 153.8 mg (0.33 mmol) iodonium salt. The solvent was evaporated under reduced pressure and the crude product was purified by column chromatography using hexanes:ethyl acetate eluent gradient. The product was obtained as white powder (60.6 mg, 0.284 mmol, 95%). Mp. 101-103 °C. R_f = 0.63 (hexanes:EtOAc = 4:1).

MS (EI, 70 eV): m/z (%): 213 (43, [M^+]), 185 (23), 166 (53), 158 (151), 135 (33), 116 (14), 96 (50), 91 (94), 77 (56), 69 (100), 51 (35).

^{1}H NMR (500 MHz, DMSO-d_6) δ = 8.63 (dq, *J* = 14.2, 2.2 Hz, 1H), 8.27 (dt, *J* = 8.4, 0.9 Hz, 1H), 8.19 (dt, *J* = 8.4, 0.9 Hz, 1H), 7.73 (ddd, *J* = 8.2, 7.0, 1.0 Hz, 1H), 7.55 (ddd, *J* = 8.1, 7.0, 0.9 Hz, 1H), 7.06 (dq, *J* = 14.0, 6.9 Hz, 1H) ppm.

^{13}C NMR (126 MHz, DMSO-d_6) δ = 146.1, 132.1, 131.5 (q, *J* = 8.0 Hz), 129.9, 126.1, 124.3 (q, *J* = 268.4 Hz), 120.4, 111.9, 106.0 (q, *J* = 34.2 Hz) ppm.

^{19}F NMR (235 MHz, $CDCl_3$) δ = -62.24 ppm.

IR (thin film, ATR) ν = 1685, 1490, 1460, 1351, 1296, 1271, 1130, 1107, 1050, 951, 938, 874, 809, 766, 746, 703, 695, 675, 658 cm^{-1}.

HRMS m/z $[M+H]^+$ calculated for $C_9H_7F_3N_3^+$: 214.0592, found: 214.0587.

(*E*)-4-Bromo-1-(3,3,3-trifluoroprop-1-en-1-yl)-1*H*-pyrazole (4)

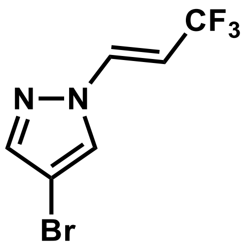

General procedure was followed using 58.8 mg (0.40 mmol) 4-bromo-1*H*-pyrazole, 59.1 mg (0.80 mmol) Li_2CO_3 and 205.1 mg (0.44 mmol) iodonium salt. The solvent was evaporated under reduced pressure and the crude product was purified by column chromatography using hexanes:ethyl acetate eluent gradient. The product was obtained as colorless oil (76.4 mg, 0.317 mmol, 80%). R_f = 0.75 (hexanes:EtOAc = 4:1).

MS (EI, 70 eV): m/z (%): 242 (94, $[M]^+$), 240 (100, $[M]^+$), 223 (14), 221 (16), 148 (27), 146 (30), 134 (16), 114 (16), 107 (16), 75 (19), 69 (52), 52 (14).

^{1}H NMR (250 MHz, $CDCl_3$) δ = 7.64 (s, 1H), 7.63 (s, 1H), 7.40 (dq, *J* = 14.1, 2.1 Hz, 1H), 6.23 (dq, *J* = 13.5, 6.6 Hz, 1H) ppm.

^{13}C NMR (63 MHz, $CDCl_3$) δ = 143.8, 133.3 (q, *J* = 7.7 Hz), 129.7, 123.5 (q, *J* = 267.9 Hz), 105.0 (q, *J* = 35.4 Hz), 97.4 ppm.

^{19}F NMR (235 MHz, $CDCl_3$) δ = -61.90 ppm.

IR (thin film, ATR): 1691, 1331, 1301, 1268, 1243, 1204, 1165, 1113, 967, 941, 792, 651 cm^{-1}.

HRMS m/z $[M+H]^+$ calculated for $C_6H_5N_2BrF_3^+$: 240.9588, found: 240.9589.

(*E*)-3,5-Dimethyl-1-(3,3,3-trifluoroprop-1-en-1-yl)-1*H*-pyrazole (5)

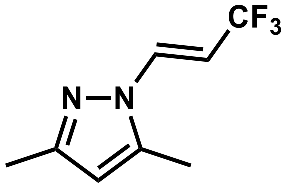

General procedure was followed using 48.1 mg (0.50 mmol) 3,5-dimethyl-1*H*-pyrazole, 78.9 mg (1.0 mmol) Li_2CO_3 and 256.4 mg (0.55 mmol) iodonium salt. The solvent was evaporated under reduced pressure and the crude product was purified by column chromatography using hexanes:ethyl acetate eluent gradient. The product was obtained as colorless liquid (59.3 mg, 0.311 mmol, 63%). R_f = 0.63 (hexanes:EtOAc = 4:1).

MS (EI, 70 eV): m/z (%): 190 (100, $[M^+]$), 175 (19), 171 (33), 148 (10), 121 (34), 95 (22), 80 (36), 75 (16), 69 (53).

^{1}H NMR (250 MHz, $CDCl_3$) δ = 7.29 (d, *J* = 15.3 Hz, 1H), 6.27 (dq, *J* = 13.8, 6.9 Hz, 1H), 5.94 (s, 1H), 2.31 (s, 3H), 2.26 (s, 3H) ppm.

^{13}C NMR (63 MHz, $CDCl_3$) δ = 152.5, 141.2, 129.9 (q, *J* = 8.0 Hz), 124.3 (q, *J* = 267.1 Hz), 108.7, 103.0 (q, *J* = 34.8 Hz), 13.8, 10.8 ppm.

^{19}F NMR (235 MHz, $CDCl_3$) δ = -61.44 (d, *J* = 6.9 Hz) ppm.

IR (thin film, ATR): 1687, 1579, 1277, 1111, 943, 652 cm^{-1}.

HRMS m/z $[M+H]^+$ calculated for $C_8H_{10}N_2F_3^+$: 191.0796, found: 191.0798.

(*E*)-4-Iodo-3,5-dimethyl-1-(3,3,3-trifluoroprop-1-en-1-yl)-1*H*-pyrazole (6)

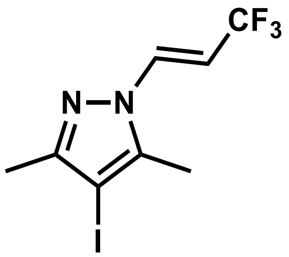

General procedure was followed using 66.6 mg (0.30 mmol) 4-iodo-3,5-dimethyl-1*H*-pyrazole, 44.3 mg (0.60 mmol) Li_2CO_3 and 153.8 mg (0.33 mmol) iodonium salt. The solvent was evaporated under reduced pressure and the crude product was purified by column chromatography using hexanes:ethyl acetate eluent gradient. The product was obtained as white crystals (75.0 mg, 0.237 mmol, 80%). Mp. 31-33 °C. R_f = 0.60 (hexanes:EtOAc = 10:1).

MS (EI, 70 eV): m/z (%): 316 (100, $[M^+]$), 297 (11), 206 (10), 148 (11), 136 (11), 69 (20).

^{1}H NMR (250 MHz, $CDCl_3$) δ = 7.33 (dd, *J* = 13.7, 2.1 Hz, 1H), 6.29 (dq, *J* = 13.6, 6.9 Hz, 1H), 2.37 (s, 3H), 2.26 (s, 3H) ppm.

^{13}C NMR (63 MHz, $CDCl_3$) δ = 154.0, 142.5, 130.2 (q, *J* = 7.9 Hz), 124.0 (q, *J* = 267.7 Hz), 103.9 (q, *J* = 34.9 Hz), 67.7, 14.4, 12.0 ppm.

^{19}F NMR (235 MHz, $CDCl_3$) δ = -61.53 ppm.

IR (thin film, ATR): ν = 1683, 1329, 1299, 1279, 1251, 1133, 1117, 1051, 995, 940, 740, 718, 658 cm^{-1}.

HRMS m/z $[M+H]^+$ calculated for $C_8H_9F_3N_2I^+$: 316.9763, found: 316.9769.

(*E*)-3,5-Diphenyl-1-(3,3,3-trifluoroprop-1-en-1-yl)-1*H*-pyrazole (7)

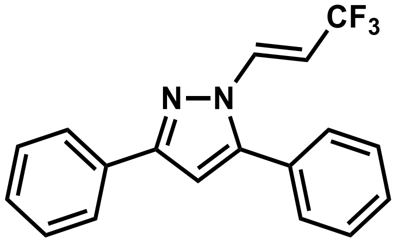

General procedure was followed using 66.1 mg (0.30 mmol) 3,5-diphenyl-1*H*-pyrazole, 44.3 mg (0.60 mmol) Li_2CO_3 and 153.8 mg (0.33 mmol) iodonium salt. The solvent was evaporated under reduced pressure and the crude product was purified by column chromatography using

hexanes:ethyl acetate eluent gradient. The product was obtained as white powder (77.4 mg, 0.246 mmol, 82%). Mp. 97-98 °C. R_f = 0.68 (hexanes:EtOAc = 5:1).

MS (EI, 70 eV): m/z (%): 314 (100, [M^+]), 295 (11), 145 (60), 189 (11), 168 (11), 142 (22), 115 (15), 104 (15), 89 (9), 77 (37), 69 (9), 51 (13).

^{1}H NMR (250 MHz, $CDCl_3$) δ = 7.94 (dd, *J* = 8.0, 1.7 Hz, 2H), 7.50 (dtd, *J* = 18.9, 5.1, 1.9 Hz, 9H), 6.77 (s, 1H), 6.59 (dq, *J* = 13.9, 7.0 Hz, 1H) ppm.

^{13}C NMR (63 MHz, $CDCl_3$) δ = 154.0, 146.8, 132.1, 131.3 (q, *J* = 7.9 Hz), 129.8, 129.5, 129.3, 129.1, 128.9, 128.7, 126.3, 124.2 (q, *J* = 266.8 Hz), 106.4, 104.9 (q, *J* = 34.9 Hz) ppm.

^{19}F NMR (235 MHz, $CDCl_3$) δ = -61.38 ppm.

IR (thin film, ATR): ν = 1683, 1558, 1489, 1460, 1331, 1299, 1266, 1217, 1167, 1120, 1077, 953, 822, 762, 738, 703 cm^{-1}.

HRMS m/z $[M+H]^+$ calculated for $C_{18}H_{14}F_3N_2^+$: 315.1109, found: 315.1100.

(*E*)-3,5-Di-p-tolyl-1-(3,3,3-trifluoroprop-1-en-1-yl)-1*H*-pyrazole (8)

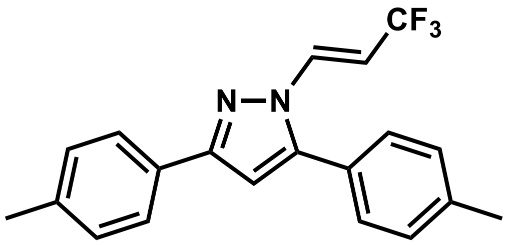

General procedure was followed using 74.5 mg (0.30 mmol) 3,5-di-4-tolyl-1*H*-pyrazole, 44.3 mg (0.60 mmol) Li_2CO_3 and 153.8 mg (0.33 mmol) iodonium salt. The solvent was evaporated under reduced pressure and the crude product was purified by column chromatography using hexanes:ethyl acetate eluent gradient. The product was obtained as white powder (80.9 mg, 0.236 mmol, 79%). Mp. 117-118 °C. R_f = 0.88 (hexanes:EtOAc = 2:1).

MS (EI, 70 eV): m/z (%): 342 (100, [M^+]), 327 (29), 273 (40), 258 (29), 202 (20), 182 (20), 156 (21), 129 (17), 115 (69), 91 (90), 89 (48), 77 (22), 69 (33), 65 (48).

^{1}H NMR (250 MHz, $CDCl_3$) δ = 7.72 (d, *J* = 8.2 Hz, 2H), 7.38 (dq, *J* = 13.6, 2.1 Hz, 1H), 7.23 (s, 4H), 7.17 (d, *J* = 7.9 Hz, 2H), 6.59 (s, 1H), 6.46 (dq, *J* = 14.0, 7.0 Hz, 1H), 2.36 (s, 3H), 2.31 (s, 3H) ppm.

^{13}C NMR (63 MHz, $CDCl_3$) δ = 154.1, 146.9, 139.9, 139.0, 131.5 (q, *J* = 7.7 Hz), 129.9, 129.6, 129.4, 129.3, 126.2, 125.9, 124.3 (q, *J* = 267.5 Hz), 106.0, 104.4 (q, *J* = 34.7 Hz), 21.4, 21.4 ppm.

^{19}F NMR (235 MHz, $CDCl_3$) δ = -61.16 ppm.

IR (thin film, ATR): 1683, 1504, 1297, 1284, 1269, 1250, 1161, 1111, 1062, 952, 941, 831, 797, 665 cm^{-1}.

HRMS m/z $[M+H]^+$ calculated for $C_{20}H_{18}N_2F_3^+$: 343.1422, found: 343.1417.

(*E*)-3,5-Bis(4-chlorophenyl)-1-(3,3,3-trifluoroprop-1-en-1-yl)-1*H*-pyrazole (9)

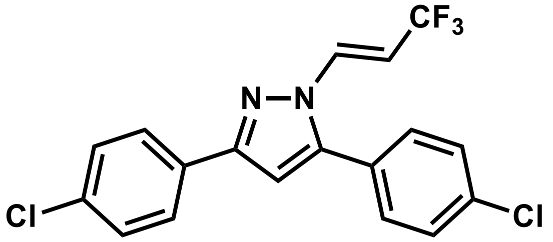

General procedure was followed using 86.7 mg (0.30 mmol) 3,5-bis(4-chlorophenyl)-1*H*-pyrazole, 44.3 mg (0.60 mmol) Li_2CO_3 and 153.8 mg (0.33 mmol) iodonium salt. The solvent was evaporated under reduced pressure and the crude product was purified by column chromatography using hexanes:ethyl acetate eluent gradient. The product was obtained as off-white solid (100.9 mg, 0.263 mmol, 88%). Mp. 128-130 °C. R_f = 0.83 (hexanes:EtOAc = 2:1).

MS (EI, 70 eV): m/z (%): 384 (42, [M^+]), 382 (80, [M^+]), 280 (21), 278 (50), 223 (11), 207 (16), 189 (25), 167 (21), 137 (41), 113 (37), 111 (100), 102 (28), 102 (28), 95 (14), 89 (13), 75 (87), 73 (50), 69 (79), 63 (13), 51 (18).

^{1}H NMR (250 MHz, $CDCl_3$) δ = 7.91 – 7.76 (m, 2H), 7.58 – 7.47 (m, 2H), 7.47 – 7.29 (m, 5H), 6.71 (s, 1H), 6.56 (dq, *J* = 13.7, 6.9 Hz, 1H) ppm.

^{13}C NMR (63 MHz, $CDCl_3$) δ = 153.0, 145.7, 136.3, 135.1, 130.8 (d, *J* = 18.8 Hz), 130.7, 130.5, 129.7, 129.2, 127.5, 127.0, 124.0 (q, *J* = 267.8 Hz), 106.4, 105.7 (q, *J* = 34.9 Hz) ppm.

^{19}F NMR (235 MHz, $CDCl_3$) δ = -61.49 ppm.

IR (thin film, ATR): 1685, 1488, 1434, 1283, 1094, 1016, 954, 837, 664 cm^{-1}.

HRMS m/z $[M+H]^+$ calculated for $C_{18}H_{12}N_2Cl_2F_3^+$: 383.0330, found: 383.0334.

(*E*)-3,5-Bis(4-bromophenyl)-1-(3,3,3-trifluoroprop-1-en-1-yl)-1*H*-pyrazole (10)

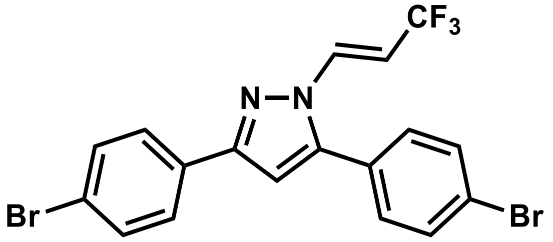

General procedure was followed using 113.4 mg (0.30 mmol) 3,5-bis(4-bromophenyl)-1*H*-pyrazole, 44.3 mg (0.60 mmol) Li_2CO_3 and 153.8 mg (0.33 mmol) iodonium salt. The solvent was evaporated under reduced pressure and the crude product was purified by column chromatography using hexanes:ethyl acetate eluent gradient. The product was obtained as white solid (75.1 mg, 0.159 mmol, 53%). Mp. 141-145 °C. R_f = 0.58 (hexanes:EtOAc = 10:1).

MS (EI, 70 eV): m/z (%): 472 (58, [M^+]), 470 (31, [M^+]), 324 (43), 322 (47), 242 (17), 216 (17), 206 (15), 187 (15), 167 (49), 157 (60), 155 (67), 140 (33), 127 (15), 115 (16), 102 (39), 75 (85), 69 (100).

^{1}H NMR (250 MHz, $CDCl_3$) δ = 7.70 – 7.63 (m, 2H), 7.61 – 7.54 (m, 2H), 7.50 – 7.42 (m, 2H), 7.28 (dt, *J* = 13.7, 2.1 Hz, 1H), 7.23 – 7.15 (m, 2H), 6.60 (s, 1H), 6.46 (dq, *J* = 13.9, 6.9 Hz, 1H) ppm.

^{13}C NMR (63 MHz, $CDCl_3$) δ = 153.0, 145.7, 132.6, 132.1, 131.0 (q, *J* = 7.8 Hz), 130.9, 127.8, 127.4, 124.5, 123.9 (q, *J* = 267.8 Hz), 123.3, 117.6, 106.4, 105.7 (q, *J* = 35.0 Hz) ppm.

^{19}F NMR (235 MHz, $CDCl_3$) δ = -61.44 ppm.

IR (thin film, ATR): 1680, 1484, 1266, 1105, 1012, 953, 835, 736, 662 cm^{-1}.

HRMS m/z $[M+H]^+$ calculated for $C_{18}H_{12}N_2Br_2F_3^+$: 470.9319, found: 470.9326.

(*E*)-3,5-Bis(2-bromophenyl)-1-(3,3,3-trifluoroprop-1-en-1-yl)-1*H*-pyrazole (11)

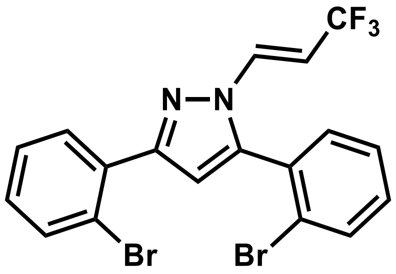

General procedure was followed using 113.4 mg (0.30 mmol) 3,5-bis(2-bromophenyl)-1*H*-pyrazole, 44.3 mg (0.60 mmol) Li_2CO_3 and 153.8 mg (0.33 mmol) iodonium salt. The solvent was evaporated under reduced pressure and the crude product was purified by column chromatography using hexanes:ethyl acetate eluent gradient. The product was obtained as colorless oil (100.2 mg, 0.212 mmol, 71%). R_f = 0.65 (hexanes:EtOAc = 4:1).

MS (EI, 70 eV): m/z (%): 474 (17, $[M^+]$), 472 (38, $[M^+]$), 470 (21, $[M^+]$), 393 (49), 391 (52), 324 (100), 322 (97), 312 (17), 243 (53), 241 (50), 216 (19), 210 (28), 190 (49), 189 (38), 167 (40), 163 (18), 157 (40), 155 (39), 140 (35), 102 (37), 75 (60), 69 (78).

^{1}H NMR (250 MHz, $CDCl_3$) δ = 7.76 (dd, *J* = 7.8, 1.8 Hz, 1H), 7.67 (dd, *J* = 7.6, 1.7 Hz, 1H), 7.63 – 7.56 (m, 1H), 7.45 – 7.26 (m, 4H), 7.17 (td, *J* = 7.8, 1.8 Hz, 1H), 7.03 (dt, *J* = 13.7, 2.2 Hz, 1H), 6.89 (s, 1H), 6.42 (dq, *J* = 13.8, 6.9 Hz, 1H) ppm.

^{13}C NMR (63 MHz, $CDCl_3$) δ = 153.3, 143.6, 133.9, 133.7, 133.3, 132.6, 131.7, 131.4, 131.2 (q, *J* = 7.9 Hz), 130.2, 129.8, 127.9, 127.7, 124.6, 124.0 (q, *J* = 267.8 Hz), 122.3, 111.2, 106.0, 105.2 (q, *J* = 34.9 Hz) ppm.

^{19}F NMR (235 MHz, $CDCl_3$) δ = -61.48 ppm.

IR (thin film, ATR): 1685, 1448, 1284, 1113, 1025, 943, 824, 757, 665 cm^{-1}.

HRMS m/z $[M+H]^+$ calculated for $C_{18}H_{12}N_2Br_2F_3^+$: 470.9319, found: 470.9322.

(*E*)-3,5-Bis(4-methoxyphenyl)-1-(3,3,3-trifluoroprop-1-en-1-yl)-1*H*-pyrazole (12)

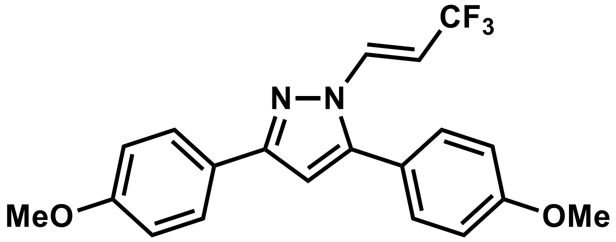

General procedure was followed using 84.1 mg (0.30 mmol) 3,5-bis(4-methoxyphenyl)-1*H*-pyrazole, 44.3 mg (0.60 mmol) Li_2CO_3 and 153.8 mg (0.33 mmol) iodonium salt. The solvent was evaporated under reduced pressure and the crude product was purified by column chromatography using hexanes:ethyl acetate eluent gradient. The product was obtained as off-white solid (100.1 mg, 0.267 mmol, 90%). Mp. 116-118 °C. R_f = 0.69 (hexanes:EtOAc = 2:1).

MS (EI, 70 eV): m/z (%): 374 (100, [M^+]), 359 (18), 305 (13), 247 (8), 198 (11), 165 (17), 133 (11), 89 (18), 77 (30), 69 (21), 63 (17).

^{1}H NMR (250 MHz, $CDCl_3$) δ = 7.85 (d, *J* = 8.8 Hz, 2H), 7.45 (dq, *J* = 13.7, 2.1 Hz, 1H), 7.35 (d, *J* = 8.7 Hz, 2H), 7.01 (dd, *J* = 14.0, 8.7 Hz, 4H), 6.62 (s, 1H), 6.52 (dq, *J* = 14.0, 7.1 Hz, 1H), 3.88 (s, 3H), 3.86 (s, 3H) ppm.

^{13}C NMR (63 MHz, $CDCl_3$) δ = 160.7, 160.4, 153.8, 146.7, 131.4 (q, *J* = 7.8 Hz), 130.8, 127.6, 124.9, 124.3 (q, *J* = 267.4 Hz), 121.0, 114.7, 114.3, 105.6, 104.1 (q, *J* = 34.7 Hz), 55.5, 55.4 ppm.

^{19}F NMR (235 MHz, $CDCl_3$) δ = -61.10 ppm.

IR (thin film, ATR): 1685, 1614, 1504, 1437, 1251, 1174, 1105, 1031, 951, 839, 665 cm^{-1}.

HRMS m/z $[M+H]^+$ calculated for $C_{20}H_{18}N_2O_2F_3^+$: 375.1320, found: 375.1324.

(*E*)-3,5-Bis(4-nitrophenyl)-1-(3,3,3-trifluoroprop-1-en-1-yl)-1*H*-pyrazole (13)

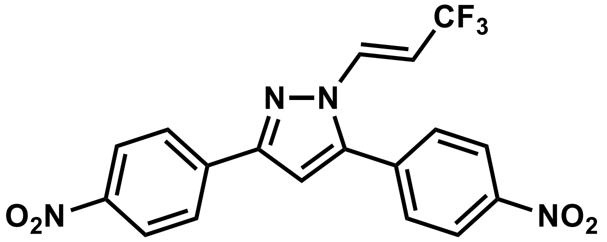

General procedure was followed using 93.1 mg (0.30 mmol) 3,5-bis(4-nitrophenyl)-1*H*-pyrazole, 44.3 mg (0.60 mmol) Li_2CO_3 and 153.8 mg (0.33 mmol) iodonium salt. The solvent was evaporated under reduced pressure and the crude product was purified by column chromatography using hexanes:ethyl acetate eluent gradient. The product was obtained as pale yellow solid (88.7 mg, 0.217 mmol, 73%). Mp. 174-176 °C. R_f = 0.0.33 (hexanes:EtOAc = 5:1).

MS (EI, 70 eV): m/z (%): 404 (100, [M^+]), 357 (11), 289 (25), 242 (17), 210 (12), 190 (14), 141 (15), 75 (12)

^{1}H NMR (300 MHz, DMSO-d_6) δ = 8.43 (d, *J* = 8.8 Hz, 2H), 8.36 (d, *J* = 8.9 Hz, 2H), 8.26 (d, *J* = 8.9 Hz, 2H), 7.90 (d, *J* = 8.8 Hz, 2H), 7.63 (dd, *J* = 13.6, 2.2 Hz, 1H), 7.57 (s, 1H), 6.79 (dq, *J* = 14.2, 7.2 Hz, 1H) ppm.

^{13}C NMR (63 MHz, DMSO) δ = 151.2, 148.0, 147.6, 145.1, 137.5, 134.1, 132.1 (q, *J* = 7.6 Hz), 130.7, 127.0, 124.3, 124.3, 124.0 (q, *J* = 267.7 Hz), 108.7, 105.5 (q, *J* = 34.1 Hz) ppm.

^{19}F NMR (235 MHz, DMSO-d_6) δ = -60.03 ppm.

IR (thin film, ATR): ν = 1685, 1516, 1342, 1325, 1299, 1275, 1262, 1171, 1126, 1107, 940, 867, 854, 818, 753, 703, 662 cm^{-1}.

HRMS m/z [M]$^+$ calculated for $C_{18}H_{11}N_4O_4F_3^+$: 404.0732, found: 404.07551.

(*E*)-4-Iodo-3,5-diphenyl-1-(3,3,3-trifluoroprop-1-en-1-yl)-1*H*-pyrazole (14)

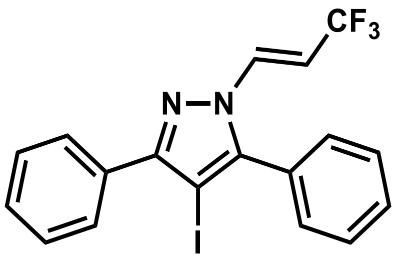

General procedure was followed using 103.9 mg (0.30 mmol) 4-iodo-3,5-diphenyl-1*H*-pyrazole, 44.3 mg (0.60 mmol) Li_2CO_3 and 153.8 mg (0.33 mmol) iodonium salt. The solvent was evaporated under reduced pressure and the crude product was purified by column chromatography using hexanes:ethyl acetate eluent gradient. The product was obtained as white crystals (65.2 mg, 0.148 mmol, 50%). Mp. 93-95 °C. R_f = 0.73 (hexanes:EtOAc = 5:1).

MS (EI, 70 eV): m/z (%): 440 (100, [M$^+$]), 371 (25), 244 (57), 189 (71), 163 (10(, 104 (15), 89 (10), 77 (27), 69 (15), 51 (13).

^{1}H NMR (250 MHz, $CDCl_3$) δ = 7.84 (dd, *J* = 7.6, 2.1 Hz, 2H), 7.58 – 7.32 (m, 8H), 7.29 – 7.13 (m, 1H), 6.43 (dq, *J* = 13.8, 6.9 Hz, 1H) ppm.

^{13}C NMR (63 MHz, $CDCl_3$) δ = 154.8, 147.8, 131.9, 131.3 (q, *J* = 7.9 Hz), 130.7, 130.4, 129.3, 129.3, 128.8, 128.5, 128.1, 123.8 (q, *J* = 267.9 Hz), 105.6 (q, *J* = 35.0 Hz) ppm.

^{19}F NMR (235 MHz, $CDCl_3$) δ = -61.64 ppm.

IR (thin film, ATR): ν = 1683, 1296, 1264, 1184, 1115, 975, 947, 831, 766, 733, 701, 673, 664 cm^{-1}.

HRMS m/z [M+H]$^+$ calculated for $C_{18}H_{13}F_3N_2I^+$: 441.0076, found: 441.0063.

Ethyl (*E*)-3-(trifluoromethyl)-1-(3,3,3-trifluoroprop-1-en-1-yl)-1*H*-pyrazole-4-carboxylate (15)

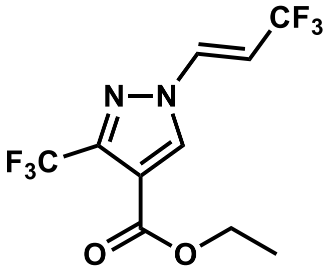

General procedure was followed using 104.1 mg (0.50 mmol) ethyl 3-(trifluoromethyl)-1*H*-pyrazole-4-carboxylate, 73.9 mg (1.0 mmol) Li_2CO_3 and 256.4 mg (0.55 mmol) iodonium salt. The solvent was evaporated under reduced pressure and the crude product was purified by column chromatography using hexanes:ethyl acetate eluent gradient. The product was obtained as white solid (95.4 mg, 0.316 mmol, 63%). Mp. 65-66 °C. R_f = 0.54 (hexanes:EtOAc = 4:1).

MS (EI, 70 eV): m/z (%): 302 (8, $[M]^+$), 283 (6), 274 (44), 257 (100), 230 (8), 210 (3), 163 (7), 122 (4), 69 (9).

^{1}H NMR (250 MHz, $CDCl_3$) δ = 8.27 (d, *J* = 1.3 Hz, 1H), 7.49 (dd, *J* = 14.0, 2.1 Hz, 1H), 6.54 (dq, *J* = 13.3, 6.5 Hz, 1H), 4.34 (q, *J* = 7.1 Hz, 2H), 1.35 (t, *J* = 7.1 Hz, 3H) ppm.

^{13}C NMR (63 MHz, $CDCl_3$) δ = 160.1, 144.3 (q, *J* = 39.3 Hz), 136.2, 132.7 (q, *J* = 7.8 Hz), 122.9 (q, *J* = 268.6 Hz), 119.9 (q, *J* = 270.4 Hz), 115.8, 109.6 (q, *J* = 35.9 Hz), 61.7, 14.0 ppm.

^{19}F NMR (235 MHz, $CDCl_3$) δ = -62.75, -62.82 ppm.

IR (thin film, ATR): 1702, 1691, 1558, 1493, 1355, 1299, 1254, 1204, 1176, 1122, 1053, 1010, 958, 848, 777, 747, 671.

HRMS m/z $[M]^+$ calculated for $C_{10}H_8N_2O_2F_6^+$: 302.0490, found: 302.04436.

(*E*)-3-Phenyl-1-(3,3,3-trifluoroprop-1-en-1-yl)-1*H*-pyrazole (16)

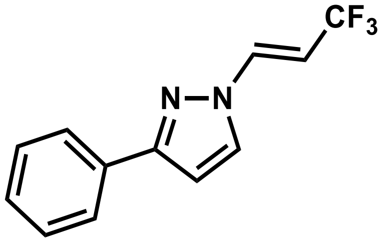

General procedure was followed using 43.3 mg (0.30 mmol) 3-phenyl-1*H*-pyrazole, 44.3 mg (0.60 mmol) Li_2CO_3 and 153.8 mg (0.33 mmol) iodonium salt. The solvent was evaporated under reduced pressure and the crude product was purified by column chromatography using hexanes:ethyl acetate eluent gradient. The product was obtained as white crystals (61.4 mg, 0.258 mmol, 86%). Mp. 37-39 °C. R_f = 0.63 (hexanes:EtOAc = 4:1).

MS (EI, 70 eV): m/z (%): 238 (100, $[M^+]$), 219 (10), 169 (12), 144 (15), 115 (9), 89 (7), 77 (24), 69 (8), 51 (11).

^{1}H NMR (250 MHz, $CDCl_3$) δ = 7.88 (dd, *J* = 8.1, 1.6 Hz, 2H), 7.57 (d, *J* = 2.7 Hz, 1H), 7.54 – 7.31 (m, 4H), 6.74 (d, *J* = 2.7 Hz, 1H), 6.37 (dq, *J* = 13.7, 6.8 Hz, 1H).

^{13}C NMR (63 MHz, $CDCl_3$) δ = 155.1, 133.6 (q, *J* = 7.7 Hz), 132.1, 131.7, 129.0, 128.9, 126.2, 124.0 (q, *J* = 267.5 Hz), 106.4, 103.9 (q, *J* = 35.1 Hz) ppm.

^{19}F NMR (235 MHz, $CDCl_3$) δ = -61.43 ppm.

IR (thin film, ATR): 1687, 1538, 1344, 1299, 1212, 1111, 941, 749, 656 cm^{-1}.

HRMS m/z $[M+H]^+$ calculated for $C_{12}H_{10}N_2F_3^+$: 239.0796, found: 239.0797.

(*E*)-3-(4-Methoxyphenyl)-5-(4-nitrophenyl)-1/2-(3,3,3-trifluoroprop-1-en-1-yl)-1*H*-pyrazole (17, mixture of regioisomers)

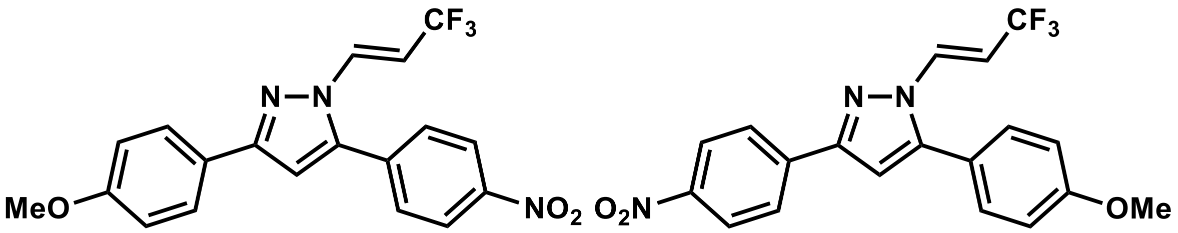

General procedure was followed using 88.6 mg (0.30 mmol) 3-(4-methoxyphenyl)-5-(4-nitrophenyl)-1*H*-pyrazole, 44.3 mg (0.60 mmol) Li_2CO_3 and 153.8 mg (0.33 mmol) iodonium salt. The solvent was evaporated under reduced pressure and the crude product was purified by column chromatography using hexanes:ethyl acetate eluent gradient. The product was obtained as pale yellow solid (97.5 mg, 0.250 mmol, 84%). Mp. 130-131 °C. R_f = 0.68 (hexanes:EtOAc = 2:1).

MS (EI, 70 eV): m/z (%): 389 (100, $[M^+]$), 320 (23), 274 (20), 207 (20), 176 (12), 147 (10), 73 (42), 69 (12).

^{1}H NMR (250 MHz, DMSO-d_6) δ = 8.37 (d, *J* = 8.7 Hz, 2H), 8.28 (d, *J* = 8.9 Hz, 2H), 8.18 (d, *J* = 8.9 Hz, 2H), 7.94 – 7.79 (m, 4H), 7.58 – 7.43 (m, 4H), 7.24 (d, *J* = 1.4 Hz, 2H), 7.13 (d, *J* = 8.7 Hz, 2H), 7.03 (d, *J* = 8.8 Hz, 2H), 6.62 (ddt, *J* = 18.9, 14.4, 7.2 Hz, 2H), 3.84 (s, 3H), 3.80 (s, 3H) ppm.

^{13}C NMR (63 MHz, DMSO-d_6) δ = 160.9, 160.6, 153.8, 151.6, 148.3, 147.9, 147.5, 144.9, 138.3, 134.9, 132.4 (qd, *J* = 7.8 Hz), 131.2, 131.0, 127.9, 127.3, 124.6, 124.6 (qd, *J* = 267.6, 8.6 Hz), 124.6, 124.2, 120.3, 115.2, 114.8, 108.0, 107.5, 104.5 (qd, *J* = 42.6, 33.9 Hz), 55.8, 55.7 ppm.

^{19}F NMR (235 MHz, DMSO-d_6) δ -59.76, -60.11 ppm.

NMR analysis is in favor of the following mixture (1:1).

IR (thin film, ATR): 1685, 1605, 1517, 1344, 1251, 1109, 954, 839, 664 cm^{-1}.

HRMS m/z $[M+H]^+$ calculated for $C_{19}H_{15}N_3O_3F_3^+$: 390.1066, found: 390.1073.

(*E*)-3-Phenyl-5-(thiophen-2-yl)-1/2-(3,3,3-trifluoroprop-1-en-1-yl)-1*H*-pyrazole (18, mixture of regioisomers 18a, 18b)

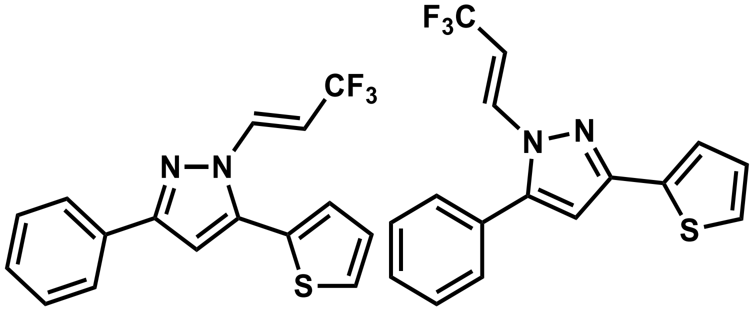

General procedure was followed using 67.9 mg (0.30 mmol) 3-phenyl-5-(thiophen-2-yl)-1*H*-pyrazole, 44.3 mg (0.60 mmol) Li_2CO_3 and 153.8 mg (0.33 mmol) iodonium salt. The solvent was evaporated under reduced pressure and the crude product was purified by column chromatography using hexanes:ethyl acetate eluent gradient.

The product was obtained as off-white powder (83.0 mg, 0.259 mmol, 75%, two regioisomer). Mp. 84-86 °C. R_f = 0.79 (hexanes:EtOAc = 2:1).

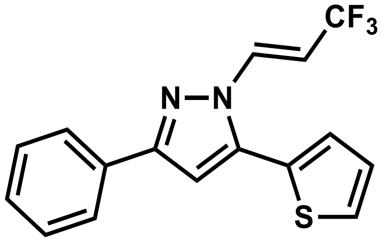

18a

MS (EI, 70 eV): m/z (%): 320 (57, [M^+]), 251 (28), 223 (6), 198 (8), 194 (8), 174 (18), 152 (20), 148 (31), 142 (25), 108 (25), 77 (100), 69 (78), 51 (40).

^{1}H NMR (500 MHz, DMSO-d_6) δ = 7.99 (d, *J* = 7.0 Hz, 2H), 7.88 (dd, *J* = 5.1, 1.1 Hz, 1H), 7.73 (dq, *J* = 13.5, 2.1 Hz, 1H), 7.51 (dd, *J* = 3.6, 1.2 Hz, 1H), 7.49 (t, *J* = 1.6 Hz, 2H), 7.43 (t, *J* = 4.8 Hz, 1H), 7.30 (dd, *J* = 5.1, 3.6 Hz, 1H), 7.29 (s, 1H), 6.66 (dq, *J* = 14.2, 7.2 Hz, 1H) ppm.

^{13}C NMR (126 MHz, DMSO-d_6) δ = 153.9, 140.1, 132.1 (q, *J* = 8.0 Hz), 131.7, 130.2, 130.0, 129.7, 129.4, 129.1, 128.3, 126.5, 124.6 (qd, *J* = 267.5, 4.8 Hz), 107.8, 104.2 (q, *J* = 34.0 Hz) ppm.

^{19}F NMR (235 MHz, $CDCl_3$) δ = -61.30 ppm.

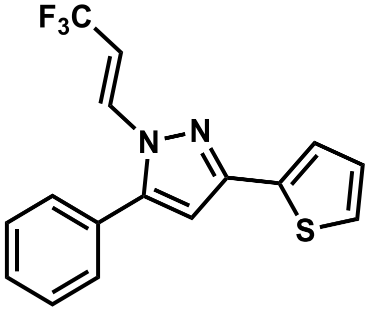

18b

MS (EI, 70 eV): m/z (%): 320 (68, [M^+]), 251 (30), 223 (8), 198 (16), 154 (18), 142 (25), 108 (33), 77 (33), 69 (100).

^{1}H NMR (500 MHz, DMSO-d_6) δ = 7.66 (dd, *J* = 3.6, 1.2 Hz, 1H), 7.64 (dd, *J* = 5.0, 1.2 Hz, 1H), 7.62 – 7.55 (m, 5H), 7.47 (d, *J* = 2.1 Hz, 1H), 7.18 (dd, *J* = 5.1, 3.6 Hz, 1H), 7.13 (s, 1H), 6.50 (dq, *J* = 14.2, 7.1 Hz, 1H) ppm.

^{13}C NMR (126 MHz, DMSO-d_6) δ = 149.5, 147.2, 134.8, 132.1 (q, *J* = 8.0 Hz), 128.5, 128.3, 127.8, 127.3, 124.6 (qd, *J* = 267.5, 4.8 Hz), 107.1, 103.6 (q, *J* = 34.0 Hz) ppm.

^{19}F NMR (235 MHz, $CDCl_3$) δ = -61.25 ppm.

NMR analysis is in favor of the following mixture (**18a**/**18b** 2:1).

GCMS analysis is in favor of the following mixture (**18a**/**18b** 4.5:1).

IR (thin film, ATR): 1681, 1269, 1105, 943, 815, 766, 693 cm^{-1}.

HRMS m/z [M+H]$^+$ calculated for $C_{16}H_{12}N_2SF_3^+$: 321.0673, found: 321.0666.

(*E*)-5-(2-Tolyl)-3-(4-tolyl)-1-(3,3,3-trifluoroprop-1-en-1-yl)-1*H*-pyrazole (19, mixture of regioisomers 19a, 19b)

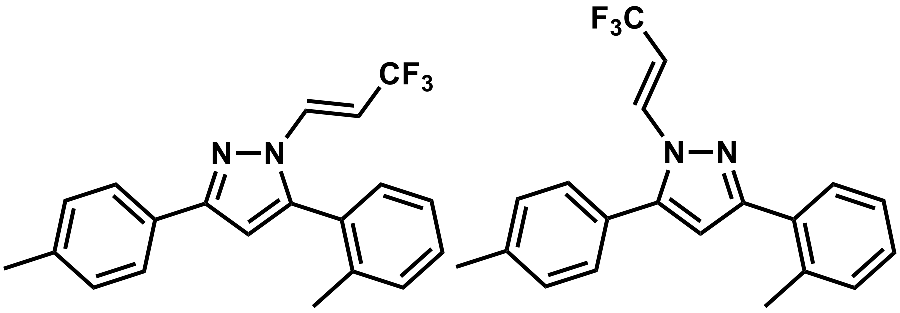

General procedure was followed using 74.5 mg (0.30 mmol) 5-(2-tolyl)-3-(4-tolyl)-1*H*-pyrazole, 44.3 mg (0.60 mmol) Li_2CO_3 and 153.8 mg (0.33 mmol) iodonium salt. The solvent was evaporated under reduced pressure and the crude product was purified by column chromatography using hexanes:ethyl acetate eluent gradient. The product was obtained as yellow oil (67.7 mg, 0.198 mmol, 66%). R_f = 0.84 (hexanes:EtOAc = 4:1).

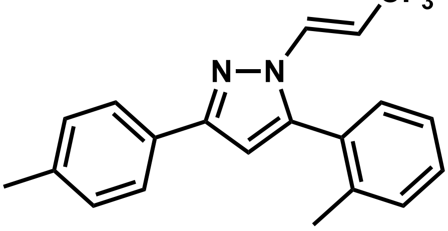

19a

MS (EI, 70 eV): m/z (%): 342 (100, [M$^+$]), 323 (10), 273 (16), 259 (78), 225 (12), 156 (17), 129 (34), 115 (34), 91 (18), 69 (4), 65 (14).

^{1}H NMR (500 MHz, DMSO-d_6) δ = 7.89 (d, *J* = 8.2 Hz, 2H), 7.48 (t, *J* = 2.1 Hz, 1H), 7.46 – 7.43 (m, 1H), 7.37 (m, 1H), 7.36 – 7.33 (m, 1H), 7.30 (d, *J* = 7.8 Hz, 2H), 7.13 (s, 1H), 7.08 (dq, *J* = 13.8, 2.2 Hz, 1H), 6.56 (dd, *J* = 13.9, 7.0 Hz, 1H), 2.36 (s, 3H), 2.18 (s, 3H) ppm.

^{13}C NMR (126 MHz, DMSO-d_6) δ = 153.4, 145.3, 138.6, 137.3, 131.2 (q, *J* = 7.7 Hz), 130.7, 130.7, 130.1, 129.8, 129.5, 128.8, 126.3, 125.9, 124.2 (dq, *J* = 267.4, 8.3 Hz), 107.1, 102.7 (q, *J* = 34.0 Hz), 21.0, 19.6 ppm.

^{19}F NMR (235 MHz, $CDCl_3$) δ = -61.18 ppm.

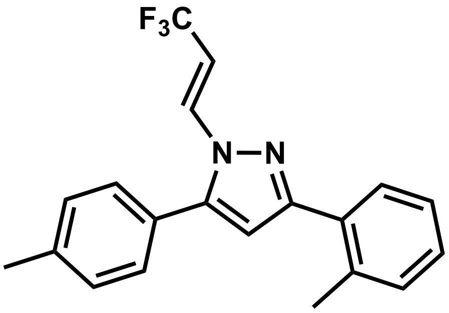

19b

MS (EI, 70 eV): m/z (%): 342 (100, [M^+]), 321 (17), 274 (14), 259 (27), 212 (43), 156 (16), 129 (15), 118 (24), 91 (17), 69 (4).

^{1}H NMR (500 MHz, DMSO-d_6) δ = 7.69 (d, *J* = 6.9 Hz, 1H), 7.53 (dq, *J* = 13.6, 2.1 Hz, 1H), 7.49 (d, *J* = 3.7 Hz, 2H), 7.48 (t, *J* = 2.1 Hz, 1H), 7.40 (d, *J* = 7.9 Hz, 2H), 7.33 – 7.32 (m, 2H), 6.98 (s, 1H), 6.56 (dq, *J* = 13.9, 7.0 Hz, 1H), 2.56 (s, 3H), 2.40 (s, 3H) ppm.

^{13}C NMR (126 MHz, DMSO) δ 154.2, 145.3, 139.4, 136.0, 131.9 (q, *J* = 7.7 Hz), 131.1, 129.3, 129.2, 129.2, 128.6, 127.6, 126.0, 125.1, 124.2 (dq, *J* = 267.4, 8.3 Hz), 109.0, 103.1 (q, *J* = 34.0 Hz), 21.4, 20.9 ppm.

^{19}F NMR (235 MHz, $CDCl_3$) δ = -61.22 ppm.

NMR and GCMS analysis is in favor of the following mixture (**19a**/**19b** 3:1).

IR (thin film, ATR): 1683, 1299, 1290, 1268, 1165, 1111, 952, 828, 762.

HRMS m/z $[M+H]^+$ calculated for $C_{20}H_{18}N_2F_3^+$: 343.1422, found: 343.1421.

(*E*)-3-Phenyl-5-(trifluoromethyl)-1-(3,3,3-trifluoroprop-1-en-1-yl)-1*H*-pyrazole (20) & (*E*)-5-phenyl-3-(trifluoromethyl)-1-(3,3,3-trifluoroprop-1-en-1-yl)-1*H*-pyrazole (21) (regioisomers)

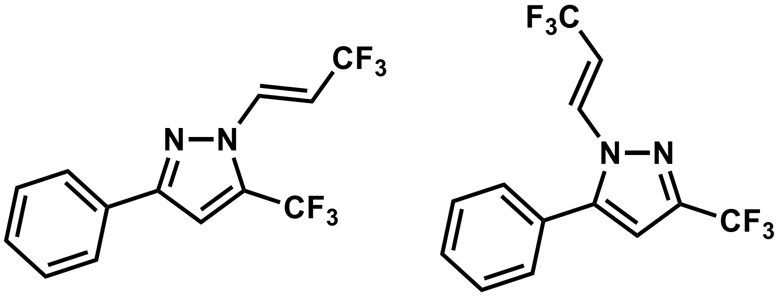

General procedure was followed using 106.1 mg (0.50 mmol) 3-phenyl-5-(trifluoromethyl)-1*H*-pyrazole, 73.9 mg (1.0 mmol) Li_2CO_3 and 256.4 mg (0.55 mmol) iodonium salt. The solvent was evaporated under reduced pressure and the crude product was purified by column chromatography using hexanes:ethyl acetate eluent gradient.

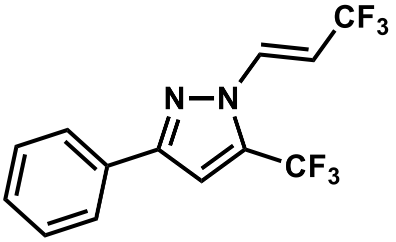

(20)

The product was obtained as white solid (46.8 mg, 0.153 mmol, 31%). Mp. 40-41 °C. R_f = 0.68 (hexanes:EtOAc = 10:1).

MS (EI, 70 eV): m/z (%): 306 (100, [M^+]), 287 (19), 237 (15), 217 (44), 212 (18), 153 (7), 133 (7), 103 (9), 89 (7), 77 (40), 69 (15).

^{1}H NMR (250 MHz, $CDCl_3$) δ = 7.90 – 7.81 (m, 2H), 7.62 – 7.39 (m, 4H), 7.08 (s, 2H), 6.67 (dq, *J* = 13.6, 6.8 Hz, 1H) ppm.

^{13}C NMR (63 MHz, $CDCl_3$) δ = 153.7, 134.1 (q, *J* = 40.0 Hz), 130.8, 130.3 (q, *J* = 7.9 Hz), 129.8, 129.1, 126.3, 123.5 (q, *J* = 267.2 Hz), 115.2 (q, *J* = 269.8 Hz), 108.0 (q, *J* = 35.4 Hz), 107.9 (q, *J* = 2.3 Hz) ppm.

^{19}F NMR (235 MHz, $CDCl_3$) δ = -58.99, -62.20 ppm.

IR (thin film, ATR): ν = 1687, 1452, 1318, 1294, 1260, 1234, 1193, 1169, 1122, 1038, 1027, 936, 822, 768, 738, 688, 667 cm^{-1}.

HRMS m/z $[M]^+$ calculated for $C_{13}H_8N_2F_6^+$: 306.0562, found: 306.05807.

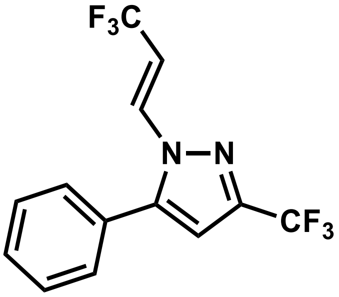

(21)

The product was obtained as white solid (53.9 mg, 0.176 mmol, 36%). Mp. 42-44 °C. R_f = 0.58 (hexanes:EtOAc = 10:1).

MS (EI, 70 eV): m/z (%): 306 (45, [M^+]), 287 (24), 237 (70), 217 (100), 168 (16), 133 (13), 115 (8), 102 (10), 77 (25), 69 (25).

^{1}H NMR (250 MHz, $CDCl_3$) δ = 7.55 (q, *J* = 3.5 Hz, 3H), 7.50 – 7.35 (m, 3H), 6.67 (s, 1H), 6.59 (qd, *J* = 13.6, 6.8 Hz, 1H) ppm.

^{13}C NMR (63 MHz, $CDCl_3$) δ = 147.0, 145.3 (q, *J* = 39.1 Hz), 130.9 (q, *J* = 7.8 Hz), 130.5, 129.6, 129.5, 127.5, 123.4 (q, *J* = 268.1 Hz), 120.9 (q, *J* = 269.4 Hz), 108.6 (q, *J* = 35.4 Hz), 106.4 (q, *J* = 2.1 Hz) ppm.

^{19}F NMR (235 MHz, $CDCl_3$) δ = -62.24, -63.04 ppm.

IR (thin film, ATR): ν = 1689, 1577, 1471, 1303, 1271, 1232, 1193, 1120, 1087, 1076, 971, 947, 824, 762, 740, 699, 682, 662 cm^{-1}.

HRMS m/z $[M]^+$ calculated for $C_{13}H_8N_2F_6^+$: 306.0592, found: 306.05831.

Ethyl (*Z*)-*N*-(4-cyano-1-((*E*)-3,3,3-trifluoroprop-1-en-1-yl)-1*H*-pyrazol-5-yl)acetimidate (22) & ethyl (*E*)-*N*-(4-cyano-1-((*E*)-3,3,3-trifluoroprop-1-en-1-yl)-1*H*-pyrazol-3-yl)acetimidate (23) (regioisomers)

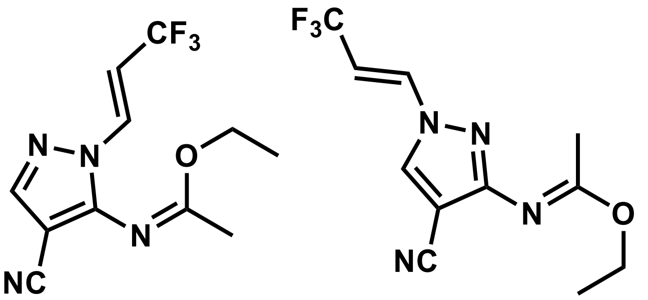

General procedure was followed using 71.3 mg (0.40 mmol) ethyl (*E*)-*N*-(4-cyano-1*H*-pyrazol-5-yl)acetimidate, 59.1 mg (0.80 mmol) Li_2CO_3 and 205.1 mg (0.44 mmol) iodonium salt. The solvent was evaporated under reduced pressure and the crude product was purified by column chromatography using hexanes:ethyl acetate eluent gradient.

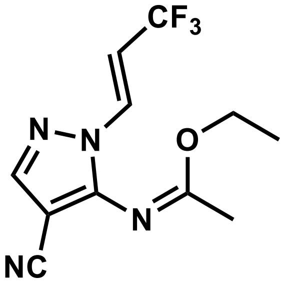

(22)

The product was obtained as white solid (51.4 mg, 0.189 mmol, 48%). Mp. 47-48 °C. R_f = 0.72 (hexanes:EtOAc = 3:1).

MS (EI, 70 eV): m/z (%): 272 (72, $[M^+]$), 229 (49), 202 (51), 175 (100), 159 (35), 133 (60), 69 (23).

^{1}H NMR (250 MHz, $CDCl_3$) δ = 7.77 (s, 1H), 7.38 (dq, *J* = 14.0, 2.2 Hz, 1H), 6.37 (dq, *J* = 13.7, 6.8 Hz, 1H), 4.35 (q, *J* = 7.1 Hz, 2H), 2.17 (s, 3H), 1.40 (t, *J* = 7.1 Hz, 3H) ppm.

^{13}C NMR (63 MHz, $CDCl_3$) δ = 169.5, 152.1, 143.5, 129.0 (q, *J* = 7.8 Hz), 123.6 (q, *J* = 268.0 Hz), 113.3, 106.7 (q, *J* = 35.5 Hz), 83.8, 64.5, 18.7, 14.0 ppm.

^{19}F NMR (235 MHz, $CDCl_3$) δ = -62.06 ppm.

IR (thin film, ATR): ν = 2229, 1687, 1640, 1551, 1400, 1379, 1322, 1305, 1292, 1269, 1184, 1107, 1051, 951, 880, 818, 710 cm^{-1}.

HRMS m/z $[M+H]^+$ calculated for $C_{11}H_{12}N_4OF_3^+$: 273.0963, found: 273.0953.

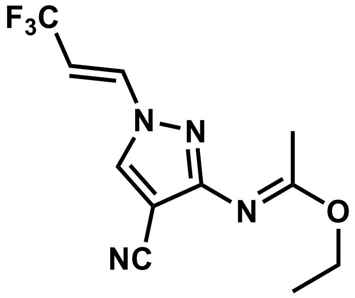

(23)

The product was obtained as white solid at -20 °C, melting between -20 °C and room temperature (41.8 mg, 0.154 mmol, 39%). R_f = 0.70 (hexanes:EtOAc = 3:1).

MS (EI, 70 eV): m/z (%): 272 ([M^+]), 228 (31), 213 (21), 202 (100), 167 (13), 122 (6), 95 (5), 69 (12).

^{1}H NMR (250 MHz, $CDCl_3$) δ = 7.87 (s, 1H), 7.38 – 7.24 (m, 1H), 6.35 (dq, *J* = 13.6, 6.7 Hz, 1H), 4.32 (q, *J* = 7.1 Hz, 2H), 2.10 (s, 2H), 1.35 (t, *J* = 7.1 Hz, 3H) ppm.

^{13}C NMR (63 MHz, $CDCl_3$) δ = 167.6, 160.2, 135.9, 132.5 (q, *J* = 7.8 Hz), 123.3 (q, *J* = 268.2 Hz), 112.2, 107.2 (q, *J* = 35.5 Hz), 90.8, 63.4, 18.1, 14.1 ppm.

^{19}F NMR (235 MHz, $CDCl_3$) δ = -62.07 ppm.

IR (thin film, ATR): ν = 2237, 1689, 1650, 1557, 1378, 1348, 1301, 1269, 1251, 1204, 1167, 1117, 1051, 954, 940, 830, 669 cm^{-1}.

HRMS m/z $[M+H]^+$ calculated for $C_{11}H_{12}N_4OF_3^+$: 273.0963, found: 273.0962.

(*E*)-4-Bromo-1-(3,3,3-trifluoroprop-1-en-1-yl)-1*H*-imidazole (24)

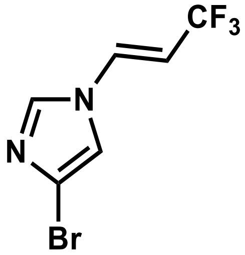

General procedure was followed using 73.5 mg (0.50 mmol) 4-bromo-1*H*-imidazole, 73.9 mg (1.0 mmol) Li_2CO_3 and 256.4 mg (0.55 mmol) iodonium salt. The solvent was evaporated under reduced pressure and the crude product was purified by column chromatography using hexanes:ethyl acetate eluent gradient. The product was obtained as pale yellow solid (73.6 mg, 0.305 mmol, 61%). Mp. 65-66 °C. R_f = 0.34 (hexanes:EtOAc = 4:1).

MS (EI, 70 eV): m/z (%): 242 (96, $[M]^+$), 240 (100, $[M]^+$), 161 (23), 135 (18), 119 (19), 107 (19), 75 (16), 69 (42), 52 (13).

^{1}H NMR (250 MHz, $CDCl_3$) δ = 7.64 (d, *J* = 1.6 Hz, 1H), 7.36 (dd, *J* = 14.3, 2.1 Hz, 1H), 7.22 (d, *J* = 1.6 Hz, 1H), 5.91 (dq, *J* = 14.2, 6.1 Hz, 1H) ppm.

^{13}C NMR (63 MHz, $CDCl_3$) δ = 137.2, 130.2 (q, *J* = 7.7 Hz), 122.9 (q, *J* = 268.7 Hz), 119.0, 115.5, 106.5 (q, *J* = 35.6 Hz) ppm.

^{19}F NMR (235 MHz, $CDCl_3$) δ = -62.23ppm.
IR (thin film, ATR): ν = 1683, 1521, 1491, 1480, 1385, 1314, 1269, 1245, 1217, 1115, 1029, 945, 882, 828, 789, 774, 660 cm^{-1}.
HRMS m/z $[M+H]^+$ calculated for $C_6H_5N_2BrF_3^+$: 240.9588, found: 240.9589.

(*E*)-2-Ethyl-1-(3,3,3-trifluoroprop-1-en-1-yl)-1*H*-imidazole (25)

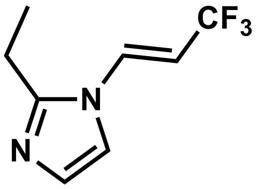

General procedure was followed using 38.5 mg (0.40 mmol) 2-ethyl-1*H*-imidazole, 59.1 mg (0.80 mmol) Li_2CO_3 and 205.1 mg (0.44 mmol) iodonium salt. The solvent was evaporated under reduced pressure and the crude product was purified by column chromatography using hexanes:ethyl acetate eluent gradient. The product was obtained as pale-yellow liquid (37.8 mg, 0.199 mmol, 50%). R_f = 0.23 (hexanes:EtOAc = 1:1).
MS (EI, 70 eV): m/z (%): 190 (75, $[M^+]$), 175 (31), 171 (14), 121 (100), 106 (28), 81 (43), 69 (21).
^1H NMR (250 MHz, $CDCl_3$) δ = 7.37 (dd, *J* = 14.2, 2.1 Hz, 1H), 7.12 (d, *J* = 1.7 Hz, 1H), 7.03 (d, *J* = 1.3 Hz, 1H), 5.78 (dq, *J* = 14.1, 6.2 Hz, 1H), 2.79 (q, *J* = 7.5 Hz, 2H), 1.34 (t, *J* = 7.5 Hz, 3H) ppm.
^{13}C NMR (63 MHz, $CDCl_3$) δ = 174.0, 150.8, 129.7 (q, *J* = 8.1 Hz), 129.7, 123.3 (q, *J* = 268.3 Hz), 115.3, 105.1 (q, *J* = 35.1 Hz), 20.3, 12.0 ppm.
^{19}F NMR (235 MHz, $CDCl_3$) δ = -61.82 ppm.
IR (thin film, ATR): ν = 1685, 1542, 1506, 1432, 1351, 1344, 1269, 1236, 1113, 1051, 1040, 945, 882, 805, 716, 684, 664 cm^{-1}.
HRMS m/z $[M+H]^+$ calculated for $C_8H_{10}N_2F_3^+$: 191.0796, found: 191.0793.

(*E*)-4,5-Diphenyl-1-(3,3,3-trifluoroprop-1-en-1-yl)-1*H*-imidazole (26)

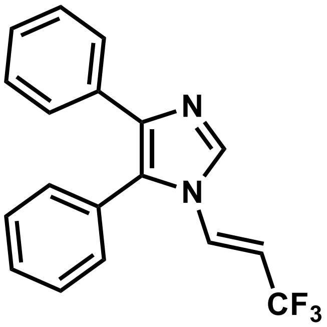

General procedure was followed using 66.1 mg (0.30 mmol) 4,5-diphenyl-1*H*-imidazole, 44.3 mg (0.60 mmol) Li_2CO_3 and 153.8 mg (0.33 mmol) iodonium salt. The solvent was evaporated under reduced pressure and the crude product was purified by column chromatography using

hexanes:ethyl acetate eluent gradient. The product was obtained as white powder (83.9 mg, 0.267 mmol, 89%). Mp. 145-146 °C. R_f = 0.23 (hexanes:EtOAc = 5:1).

MS (EI, 70 eV): m/z (%): 315 (20, $[M]^+$), 314 (100, $[M]^+$), 293 (22), 245 (8), 218 (16), 190 (10), 165 (43), 115 (9), 89 (7), 69 (6).

^{1}H NMR (250 MHz, $CDCl_3$) δ = 8.07 (s, 1H), 7.61 – 7.19 (m, 10H), 7.10 (d, *J* = 14.6 Hz, 1H), 5.92 (dq, *J* = 11.9, 5.3 Hz, 1H) ppm.

^{13}C NMR (63 MHz, $CDCl_3$) δ = 139.7, 134.5, 133.1, 130.0 (q, *J* = 7.8 Hz), 129.8, 129.7, 129.5, 128.6, 128.4, 127.8, 127.4, 127.0, 123.1 (q, *J* = 268.6 Hz), 106.2 (q, *J* = 35.3 Hz) ppm.

^{19}F NMR (235 MHz, $CDCl_3$) δ = -62.17 ppm.

IR (thin film, ATR): ν = 1683, 1495, 1394, 1258, 1245, 1187, 1132, 1118, 1074, 954, 807, 777, 766, 720, 697, 686 cm^{-1}.

HRMS m/z $[M+H]^+$ calculated for $C_{18}H_{14}N_2F_3^+$: 315.1109, found: 315.1110.

Ethyl (*E*)-1-(3,3,3-trifluoroprop-1-en-1-yl)-1*H*-imidazole-4-carboxylate (27)

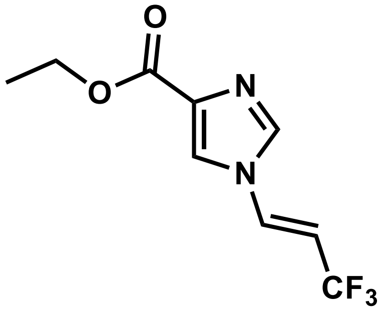

General procedure was followed using 42.0 mg (0.30 mmol) ethyl 1*H*-imidazole-4-carboxylate, 44.3 mg (0.60 mmol) Li_2CO_3 and 153.8 mg (0.33 mmol) iodonium salt. The solvent was evaporated under reduced pressure and the crude product was purified by column chromatography using hexanes:ethyl acetate eluent gradient. The product was obtained as white powder (66.5 mg, 0.284 mmol, 95%). Mp. 92-93 °C. R_f = 0.33 (hexanes:EtOAc = 1:1).

MS (EI, 70 eV): m/z (%): 234 (14), 206 (6), 189 (100), 162 (66), 135 (8), 122 (16), 69 (10).

^{1}H NMR (250 MHz, $CDCl_3$) δ = 7.94 (s, 1H), 7.82 (s, 1H), 7.47 (dt, *J* = 14.5, 2.0 Hz, 1H), 6.28 – 6.00 (m, 1H), 4.35 (q, *J* = 7.1 Hz, 2H), 1.35 (t, *J* = 7.1 Hz, 3H) ppm.

^{13}C NMR (63 MHz, $CDCl_3$) δ = 162.1, 137.8, 136.1, 130.5 (q, *J* = 7.7 Hz), 121.8, 122.7 (q, *J* = 268.8 Hz), 107.8 (q, *J* = 35.6 Hz), 61.2, 14.3 ppm.

^{19}F NMR (235 MHz, $CDCl_3$) δ = -62.52 ppm.

IR (thin film, ATR): ν = 1711, 1689, 1558, 1381, 1314, 1266, 1215, 1195, 1124, 1040, 1022, 969, 945, 884, 734, 703, 658 cm^{-1}.

HRMS m/z $[M+H]^+$ calculated for $C_9H_{10}N_2O_2F_3^+$: 235.0694, found: 235.0695.

(*E*)-5-Phenyl-1-(3,3,3-trifluoroprop-1-en-1-yl)-1*H*-tetrazole (28)

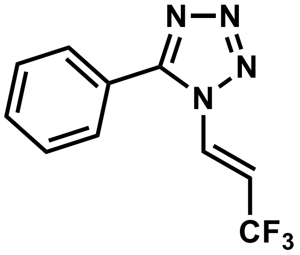

General procedure was followed using 73.1 mg (0.50 mmol) 5-phenyl-1*H*-tetrazole, 73.9 mg (1.0 mmol) Li_2CO_3 and 256.4 mg (0.55 mmol) iodonium salt. The solvent was evaporated under reduced pressure and the crude product was purified by column chromatography using hexanes:ethyl acetate eluent gradient. The product was obtained as white powder (90.4 mg, 0.376 mmol, 76%). Mp. 58-59 °C. R_f = 0.81 (hexanes:EtOAc = 4:1).

MS (EI, 70 eV): m/z (%): 212 (100, $[M-N_2]^+$), 193 (11), 165 (14), 133 (10), 115 (9), 77 (23).

^{1}H NMR (250 MHz, $CDCl_3$) δ = 8.26 – 8.16 (m, 2H), 8.04 (qd, *J* = 14.2, 2.1 Hz, 1H), 7.59 – 7.48 (m, 3H), 6.85 (dq, *J* = 14.3, 6.5 Hz, 1H) ppm.

^{13}C NMR (63 MHz, $CDCl_3$) δ 166.1, 131.4, 130.7 (q, *J* = 7.7 Hz), 129.2, 127.5, 126.3, 122.5 (q, *J* = 269.0 Hz), 111.9 (q, *J* = 36.4 Hz) ppm.

^{19}F NMR (235 MHz, $CDCl_3$) δ = -62.98 ppm.

IR (thin film, ATR): ν = 1689, 1452, 1329, 1299, 1266, 1212, 1148, 1137, 1109, 1018, 990, 941, 824, 731, 692, 679, 658 cm^{-1}.

HRMS m/z $[M-N_2]^+$ calculated for $C_{10}H_7N_2F_3^+$: 212.0561, found $[M-N_2]^+$: 212.05559.

(*E*)-2-(3,3,3-Trifluoroprop-1-en-1-yl)-2*H*-indazole (29)

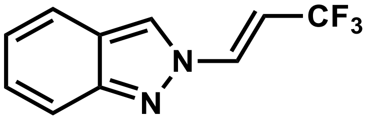

General procedure was followed using 35.4 mg (0.30 mmol) 1*H*-indazole, 44.3 mg (0.60 mmol) Li_2CO_3 and 153.8 mg (0.33 mmol) iodonium salt. The solvent was evaporated under reduced pressure and the crude product was purified by column chromatography using hexanes:ethyl acetate eluent gradient. The product was obtained as white crystals (52.8 mg, 0.249 mmol, 83%). Mp. 69-70 °C. R_f = 0.55 (hexanes:EtOAc = 5:1).

MS (EI, 70 eV): m/z (%): 212 (100, [M+]), 193 (13), 165 (10), 118 (56), 116 (11), 103 (13), 91 (12), 90 (11), 89 (14), 76 (17), 75 (15), 69 (22), 64 (13), 63 (21).

^{1}H NMR (250 MHz, $CDCl_3$) δ = 8.04 (s, 1H), 7.77 – 7.56 (m, 3H), 7.34 (dd, *J* = 8.9, 6.5 Hz, 1H), 7.11 (dd, *J* = 8.5, 6.6 Hz, 1H), 6.71 (dq, *J* = 13.6, 6.7 Hz, 1H) ppm.

^{13}C NMR (63 MHz, $CDCl_3$) δ = 150.8, 134.3 (q, *J* = 7.6 Hz), 128.8, 124.3, 123.7, 123.4 (q, *J* = 268.4 Hz), 122.7, 120.7, 118.0, 108.5 (q, *J* = 35.4 Hz) ppm.

^{19}F NMR (235 MHz, $CDCl_3$) δ = -62.25 ppm.

IR (thin film, ATR): ν = 1687, 1633, 1519, 1372, 1297, 1279, 1240, 1230, 1169, 1145, 1113, 936, 848, 787, 759, 742, 660 cm^{-1}.

HRMS m/z $[M+H]^+$ calculated for $C_{10}H_8N_2F_3^+$: 213.0640, found: 213.0639.

Ethyl (*E*)-2-(3,3,3-trifluoroprop-1-en-1-yl)-2*H*-indazole-3-carboxylate (30) & ethyl (*E*)-1-(3,3,3-trifluoroprop-1-en-1-yl)-1*H*-indazole-3-carboxylate (31)

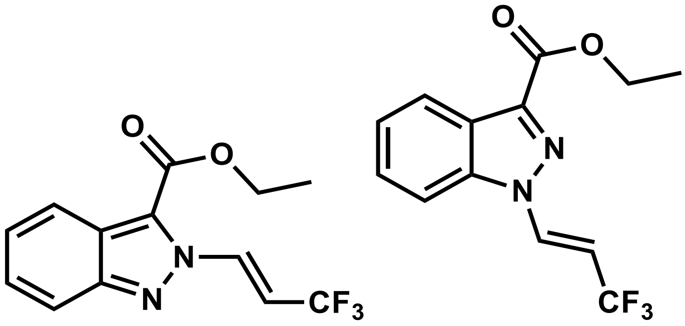

General procedure was followed using 95.1 mg (0.50 mmol) ethyl 1*H*-indazole-3-carboxylate, 73.9 mg (1.0 mmol) Li_2CO_3 and 256.4 mg (0.55 mmol) iodonium salt. The solvent was evaporated under reduced pressure and the crude product was purified by column chromatography using hexanes:ethyl acetate eluent gradient.

(30) 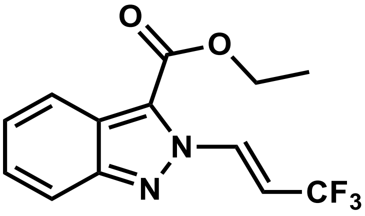

The product was obtained as white powder (44.8 mg, 0.158 mmol, 53%). Mp. 109-110 °C. R_f = 0.77 (hexanes:EtOAc = 4:1).

MS (EI, 70 eV): m/z (%): 284 (100, $[M^+]$), 256 (29), 239 (19), 219 (14), 212 (15), 191 (17), 187 (55), 162 (12), 145 (16), 129 (9), 117 (14), 103 (25), 90 (36), 69 (23).

^1H NMR (250 MHz, $CDCl_3$) δ = 8.92 (dd, *J* = 13.8, 2.2 Hz, 1H), 7.90 (dt, *J* = 8.5, 1.1 Hz, 1H), 7.64 (dt, *J* = 8.8, 1.1 Hz, 1H), 7.31 (ddd, *J* = 8.7, 6.6, 1.2 Hz, 1H), 7.27 – 7.13 (m, 1H), 6.80 (dq, *J* = 13.8, 6.9 Hz, 1H), 4.43 (q, *J* = 7.1 Hz, 2H), 1.43 (t, *J* = 7.1 Hz, 3H) ppm.

^{13}C NMR (63 MHz, $CDCl_3$) δ = 159.9, 149.2, 133.6 (q, *J* = 7.9 Hz), 128.8, 126.7, 124.7, 124.4, 123.5 (q, *J* = 268.4 Hz), 121.9, 118.7, 111.0 (q, *J* = 35.2 Hz), 61.9, 14.4 ppm.

^{19}F NMR (235 MHz, $CDCl_3$) δ = -62.31 ppm.

IR (thin film, ATR): ν = 1724, 1430, 1312, 1286, 1217, 1122, 1076, 1018, 956, 759 cm^{-1}.

HRMS m/z $[M+H]^+$ calculated for $C_{13}H_{12}N_2O_2F_3^+$: 285.0851, found: 285.0857.

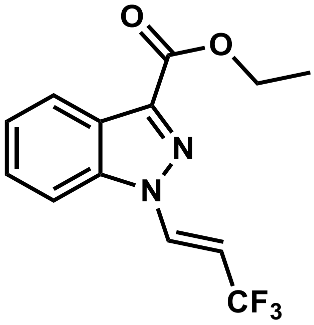

(31)

The product was obtained as white crystals (20.3 mg, 0.071 mmol, 24%). Mp. 79-81 °C. R_f = 0.67 (hexanes:EtOAc = 4:1).

MS (EI, 70 eV): m/z (%): 284 (78, [M^+]), 256 (28), 239 (100), 212 (15), 192 (69), 165 (18), 142 (23), 123 (19), 103 (64), 102 (64), 76 (27), 75 (27), 69 (12), 51 (17).

^{1}H NMR (500 MHz, DMSO-d_6) δ 8.55 (dq, *J* = 13.8, 2.2 Hz, 1H), 8.27 (d, *J* = 8.5 Hz, 1H), 8.15 (dd, *J* = 8.1, 1.1 Hz, 1H), 7.66 (ddd, *J* = 8.4, 7.0, 1.1 Hz, 1H), 7.52 – 7.46 (m, 1H), 6.68 (dq, *J* = 14.3, 7.2 Hz, 1H), 4.46 (q, *J* = 7.1 Hz, 2H), 1.40 (t, *J* = 7.1 Hz, 3H) ppm.

^{13}C NMR (126 MHz, DMSO-d_6) δ 161.1, 140.7, 139.1, 132.3 (q, *J* = 8.2, 7.7 Hz), 128.9, 125.0, 124.2 (d, *J* = 267.9 Hz), 123.3, 121.9, 111.3, 103.5 (q, *J* = 34.6 Hz), 61.3, 14.2 ppm.

^{19}F NMR (235 MHz, $CDCl_3$) δ = -62.63 ppm.

IR (thin film, ATR): ν = 1732, 1719, 1683, 1495, 1308, 1281, 1256, 1195, 1158, 1113, 1079, 1033, 936, 844, 749 cm^{-1}.

HRMS m/z [M+H]$^+$ calculated for $C_{13}H_{12}N_2O_2F_3^+$: 285.0851, found: 285.0849.

(*E*)-5-Bromo-1-(3,3,3-trifluoroprop-1-en-1-yl)-1*H*-pyrrolo[2,3-b]pyridine (32)

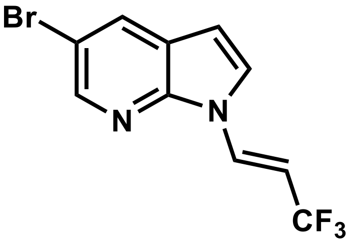

General procedure was followed using 70.9 mg (0.30 mmol) 5-bromo-1*H*-pyrrolo[2,3-b]pyridine, 44.3 mg (0.60 mmol) Li_2CO_3 and 153.8 mg (0.33 mmol) iodonium salt. The solvent was evaporated under reduced pressure and the crude product was purified by column chromatography using hexanes:ethyl acetate eluent gradient. The product was obtained as dark green solid (40.8 mg, 0.140 mmol, 47%). Mp. 65-66 °C. R_f = 0.48 (hexanes:EtOAc = 5:1).

MS (EI, 70 eV): m/z (%): 292 (11, [M^+]), 290 (9, [M^+]), 223 (67), 222 (17), 221 (71), 198 (10), 196 (15), 142 (40), 117 (15), 116 (19), 114 (10), 90 (13), 88 (14), 76 (18), 75 (47), 74 (13), 69 (100), 63 (32), 62 (26), 61 (14), 50 (14).

^{1}H NMR (250 MHz, $CDCl_3$) δ = 8.29 (d, *J* = 14.3 Hz, 1H), 8.21 (s, 1H), 7.94 (s, 1H), 7.89 (d, *J* = 2.8 Hz, 1H), 7.31 (dq, *J* = 12.6, 6.3 Hz, 1H), 6.69 (d, *J* = 2.9 Hz, 1H) ppm.

^{13}C NMR (63 MHz, $CDCl_3$) δ = 147.1, 146.3, 134.9 (q, *J* = 8.3 Hz), 134.8, 126.3, 122.8 (q, *J* = 270.3, 269.5 Hz), 114.2 (q, *J* = 35.6 Hz), 103.8, 103.0 ppm.

^{19}F NMR (235 MHz, $CDCl_3$) δ = -62.50 ppm.

IR (thin film, ATR): ν = 1681, 1333, 1320, 1279, 1247, 1221, 1118, 1081, 1046, 904, 878, 772, 727, 703, 680, 671, 654 cm^{-1}.

HRMS m/z $[M+H]^+$ calculated for $C_{10}H_7F_3N_2Br^+$: 290.9745, found: 290.9736.

(*E*)-5-Nitro-1-(3,3,3-trifluoroprop-1-en-1-yl)-1*H*-indazole (33)

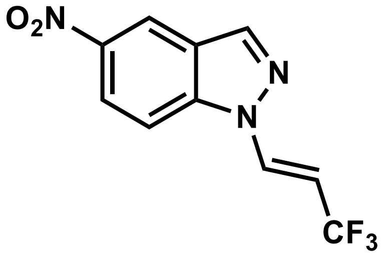

General procedure was followed using 48.9 mg (0.30 mmol) 5-nitro-1*H*-indazole, 44.3 mg (0.60 mmol) Li_2CO_3 and 153.8 mg (0.33 mmol) iodonium salt. The solvent was evaporated under reduced pressure and the crude product was purified by column chromatography using hexanes:ethyl acetate eluent gradient. The product was obtained as pale yellow solid (66.8 mg, 0.260 mmol, 87%). Mp. 99-100 °C. R_f = 0.40 (hexanes:EtOAc = 5:1).

MS (EI, 70 eV): m/z (%): 258 (12, $[M]^+$), 257 (100, $[M]^+$), 238 (11), 227 (18), 191 (32), 164 (10), 144 (13), 122 (7), 90 (10), 75 (9), 69 (9).

^{1}H NMR (250 MHz, $CDCl_3$) δ = 8.72 (dd, *J* = 2.1, 0.8 Hz, 1H), 8.43 (d, *J* = 1.0 Hz, 1H), 8.11 (dd, *J* = 9.6, 2.2 Hz, 1H), 7.88 – 7.65 (m, 2H), 6.85 (dq, *J* = 13.5, 6.7 Hz, 1H) ppm.

^{13}C NMR (63 MHz, $CDCl_3$) δ = 151.1, 144.4, 133.8 (q, *J* = 7.8 Hz), 128.6, 122.9 (q, *J* = 268.8 Hz), 122.5, 120.9, 119.9, 119.3, 111.3 (q, *J* = 35.7 Hz) ppm.

^{19}F NMR (235 MHz, $CDCl_3$) δ = -62.57 ppm.

IR (thin film, ATR): ν = 1687, 1627, 1504, 1357, 1338, 1309, 1284, 1269, 1243, 1230, 1159, 1117, 1070, 945, 833, 753, 660 cm^{-1}.

HRMS m/z $[M+H]^+$ calculated for $C_{10}H_6N_3O_2F_3^+$: 257.0412, found: 257.04057.

(*E*)-5-((tert-Butyldimethylsilyl)oxy)-1-(3,3,3-trifluoroprop-1-en-1-yl)-1*H*-indazole (34)

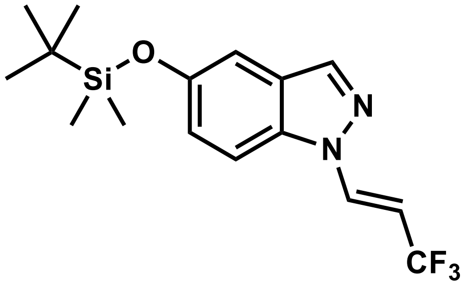

General procedure was followed using 74.5 mg (0.30 mmol) 5-((tert-butyldimethylsilyl)oxy)-1*H*-indazole, 44.3 mg (0.60 mmol) Li_2CO_3 and 153.8 mg (0.33 mmol) iodonium salt. The solvent was evaporated under reduced pressure and the crude product was purified by column chromatography using hexanes:ethyl acetate eluent gradient. The product was obtained as light brown solid (89.8 mg, 0.262 mmol, 88%). Mp. 50-51 °C. R_f = 0.59 (hexanes:EtOAc = 2:1).

MS (EI, 70 eV): m/z (%): 342 (28, [M^+]), 285 (100), 229 (19), 191 (5), 134 (5), 95 (5), 77 (23), 73 (26), 69 (4), 57 (10).

^{1}H NMR (250 MHz, $CDCl_3$) δ = 7.91 (s, 1H), 7.66 (dt, *J* = 13.8, 2.1 Hz, 1H), 7.57 (d, *J* = 9.3 Hz, 1H), 6.99 (dd, *J* = 9.3, 2.3 Hz, 1H), 6.89 (d, *J* = 2.2 Hz, 1H), 6.61 (dq, *J* = 13.5, 6.7 Hz, 1H), 1.00 (s, 9H), 0.23 (s, 6H) ppm.

^{13}C NMR (63 MHz, $CDCl_3$) δ = 151.7, 147.9, 134.4 (q, *J* = 7.6 Hz), 127.4, 123.5 (q, *J* = 268.1 Hz), 123.2, 122.8, 119.1, 107.7 (q, *J* = 35.5 Hz), 105.5, 25.8, 18.3, -4.3 ppm.

^{19}F NMR (235 MHz, $CDCl_3$) δ = -62.12 ppm.

IR (thin film, ATR): ν = 2934, 2861, 1687, 1526, 1306, 1240, 1202, 1118, 940, 900, 878, 839, 800, 781 cm^{-1}.

HRMS m/z $[M+H]^+$ calculated for $C_{16}H_{22}N_2OSiF_3^+$: 343.1454, found: 343.1446.

(*E*)-1-(3,3,3-Trifluoroprop-1-en-1-yl)-1*H*-benzo[d]imidazole (35)

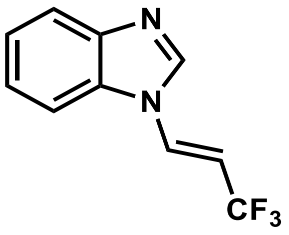

General procedure was followed using 35.4 mg (0.30 mmol) 1*H*-benzo[d]imidazole, 44.3 mg (0.60 mmol) Li_2CO_3 and 153.8 mg (0.33 mmol) iodonium salt. The solvent was evaporated under reduced pressure and the crude product was purified by column chromatography using hexanes:ethyl acetate eluent gradient. The product was obtained as off-white solid (46.6 mg, 0.220 mmol, 74%). Mp. 94-97 °C. R_f = 0.48 (hexanes:EtOAc = 3:1).

MS (EI, 70 eV): m/z (%): 212 (100, $[M]^+$), 192 (20), 165 (12), 143 (18), 103 (28), 90 (13), 75 (24), 69 (31), 51 (16).

^{1}H NMR (250 MHz, $CDCl_3$) δ = 8.13 (s, 1H), 7.92 – 7.74 (m, 1H), 7.63 (dq, *J* = 14.5, 2.1 Hz, 1H), 7.58 – 7.47 (m, 1H), 7.47 – 7.31 (m, 2H), 6.13 (dq, *J* = 14.5, 6.1 Hz, 1H) ppm.

^{13}C NMR (63 MHz, $CDCl_3$) δ = 144.5, 141.2, 132.1, 129.8 (q, *J* = 7.7 Hz), 125.1, 124.4, 123.5 (q, *J* = 268.8 Hz), 121.3, 110.7, 104.6 (q, *J* = 35.3 Hz) ppm.

^{19}F NMR (235 MHz, $CDCl_3$) δ = -61.73 ppm.

IR (thin film, ATR): ν = 1685, 1501, 1463, 1258, 1089, 953, 744, 677 cm^{-1}.

HRMS m/z $[M+H]^+$ calculated for $C_{10}H_8N_2F_3^+$: 213.0640, found: 213.0641.

(*E*)-5,6-Dimethyl-1-(3,3,3-trifluoroprop-1-en-1-yl)-1*H*-benzo[d]imidazole (36)

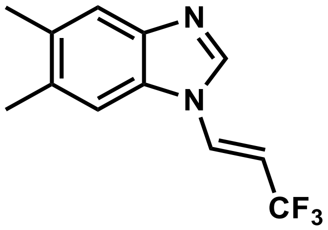

General procedure was followed using 43.9 mg (0.30 mmol) 5,6-dimethyl-1*H*-benzo[d]-imidazole, 44.3 mg (0.60 mmol) Li_2CO_3 and 153.8 mg (0.33 mmol) iodonium salt. The solvent was evaporated under reduced pressure and the crude product was purified by column chromatography using hexanes:ethyl acetate eluent gradient. The product was obtained as pale yellow solid (28.4 mg, 0.118 mmol, 40%). Mp. 97-98 °C. R_f = 0.48 (hexanes:EtOAc = 1:1).

MS (EI, 70 eV): m/z (%): 240 (100, $[M]^+$), 239 (45, $[M]^+$), 225 (60), 221 (12), 205 (7), 171 (8), 145 (5), 116 (6), 103 (8), 91 (9), 77 (8), 69 (8).

^{1}H NMR (250 MHz, $CDCl_3$) δ = 8.14 (s, 1H), 7.66 – 7.55 (m, 2H), 7.32 (s, 1H), 6.14 (dq, *J* = 12.5, 6.1 Hz, 1H), 2.42 (s, 3H), 2.38 (s, 3H) ppm.

^{13}C NMR (63 MHz, $CDCl_3$) δ = 142.4, 140.6, 134.6, 130.0 (q, *J* = 7.7 Hz), 123.6 (q, *J* = 268.3 Hz), 121.1, 111.1, 104.1 (q, *J* = 35.2 Hz). 20.8, 20.4 ppm.

^{19}F NMR (235 MHz, $CDCl_3$) δ = -61.67 ppm.

IR (thin film, ATR) ν = 1685, 1516, 1361, 1288, 1271, 1242, 1186, 1115, 945, 839, 667 cm^{-1}.

HRMS m/z $[M+H]^+$ calculated for $C_{12}H_{12}N_2F_3^+$: 241.0953, found: 241.0956.

(*E*)-6-Bromo-1-(3,3,3-trifluoroprop-1-en-1-yl)-1*H*-indazole (37)

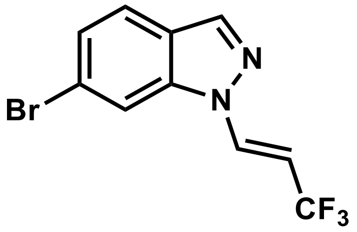

General procedure was followed using 59.1 mg (0.30 mmol) 6-bromo-1*H*-indazole, 44.3 mg (0.60 mmol) Li_2CO_3 and 153.8 mg (0.33 mmol) iodonium salt. The solvent was evaporated under reduced pressure and the crude product was purified by column chromatography using hexanes:ethyl acetate eluent gradient. The product was obtained as off-white solid (72.3 mg, 0.248 mmol, 83%). Mp. 67-68 °C. R_f = 0.53 (hexanes:EtOAc = 5:1).

MS (EI, 70 eV): m/z (%): 292 (97, $[M]^+$), 290 (100, $[M]^+$), 271 (12), 198 (34), 196 (37), 181 (7), 142 (20), 117 (11), 88 (17), 69 (30).

^{1}H NMR (250 MHz, $CDCl_3$) δ = 8.03 (s, 1H), 7.86 (s, 1H), 7.66 (dt, *J* = 13.8, 2.1 Hz, 1H), 7.50 (d, *J* = 9.0 Hz, 1H), 7.17 (dd, *J* = 9.0, 1.6 Hz, 1H), 6.70 (dq, *J* = 13.6, 6.6 Hz, 1H) ppm.

^{13}C NMR (63 MHz, $CDCl_3$) δ = 151.2, 134.1 (q, *J* = 7.6 Hz), 127.6, 124.8, 122.9, 122.8 (p, *J* = 268.4 Hz), 122.0, 121.2, 121.1, 120.4, 109.2 (q, *J* = 35.5 Hz) ppm.

^{19}F NMR (235 MHz, $CDCl_3$) δ = -62.34 ppm.

IR (thin film, ATR): ν = 1687, 1624, 1365, 1346, 1303, 1279, 1264, 1238, 1158, 1113, 1031, 938, 921, 850, 805, 734, 660 cm^{-1}.

HRMS m/z $[M+H]^+$ calculated for $C_{10}H_7N_2BrF_3^+$: 290.9745, found: 290.9751.

(*E*)-2-Chloro-3-methyl-1-(3,3,3-trifluoroprop-1-en-1-yl)-1*H*-pyrrolo[2,3-b]pyridine (38)

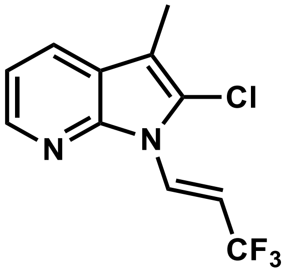

General procedure was followed using 50.0 mg (0.30 mmol) 2-chloro-3-methyl-1*H*-pyrrolo[2,3-b]pyridine, 44.3 mg (0.60 mmol) Li_2CO_3 and 153.8 mg (0.33 mmol) iodonium salt. The solvent was evaporated under reduced pressure and the crude product was purified by column chromatography using hexanes:ethyl acetate eluent gradient. The product was obtained as orange solid (28.4 mg, 0.109 mmol, 37%). Mp. 102-103 °C. R_f = 0.28 (hexanes:EtOAc = 5:1).

MS (EI, 70 eV): m/z (%): 261 (18, $[M]^+$), 260 (51, $[M]^+$), 259 (37, $[M]^+$), 241 (12), 193 (33), 191 (100), 165 (33), 155 (12), 131 (16), 102 (23), 78 (14), 69 (16).

^1H NMR (250 MHz, $CDCl_3$) δ = 8.16 (dq, *J* = 14.4, 2.1 Hz, 1H), 7.88 (dd, *J* = 7.3, 1.0 Hz, 1H), 7.67 (dd, *J* = 6.7, 1.0 Hz, 1H), 7.19 (dq, *J* = 14.4, 6.3 Hz, 1H), 6.93 (t, *J* = 7.0 Hz, 1H), 2.28 (s, 3H) ppm.

^{13}C NMR (63 MHz, $CDCl_3$) δ = 145.4, 144.1, 135.1 (q, *J* = 8.0 Hz), 132.4, 128.9, 125.9, 120.9 (q, *J* = 269.6 Hz), 113.2 (q, *J* = 35.4 Hz), 110.0, 107.1, 8.6 ppm.

^{19}F NMR (235 MHz, $CDCl_3$) δ = -62.35 ppm.

IR (thin film, ATR): ν = 1681, 1398, 1346, 1322, 1251, 1238, 1171, 1102, 1068, 1061, 1042, 943, 865, 772, 734, 690, 677 cm^{-1}.

HRMS m/z $[M+H]^+$ calculated for $C_{11}H_9F_3N_2Cl^+$: 261.0406, found: 261.0397.

(*E*)-4-Nitro-2-(3,3,3-trifluoroprop-1-en-1-yl)-2*H*-benzo[*d*][1,2,3]triazole (39) & (*E*)-7-nitro-1-(3,3,3-trifluoroprop-1-en-1-yl)-1*H*-benzo[*d*][1,2,3]triazole (40) & (*E*)-4-nitro-1-(3,3,3-trifluoroprop-1-en-1-yl)-1*H*-benzo[d][1,2,3]triazole (41)

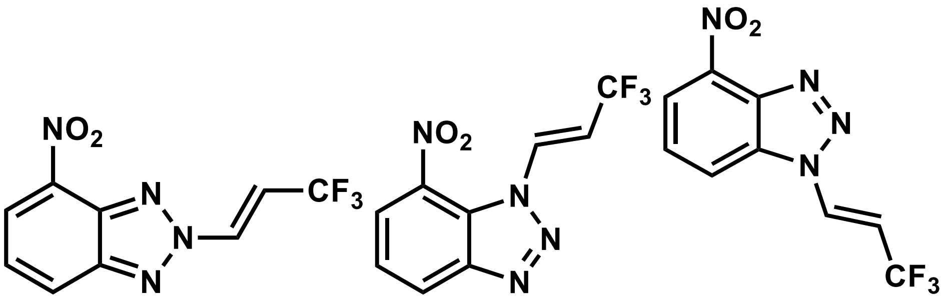

General procedure was followed using 164.1 mg (1.0 mmol) 4-nitro-1*H*-benzo[d][1,2,3]triazole, 147.8 mg (2.0 mmol) Li_2CO_3 and 512.7 mg (1.1 mmol) iodonium salt. The solvent was evaporated under reduced pressure and the crude product was purified by column chromatography using hexanes:ethyl acetate eluent gradient.

(39)

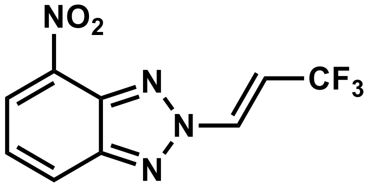

The product was obtained as off-white solid (154.3 mg, 0.598 mmol, 60%). Mp. 105-106 °C. R_f = 0.35 (hexanes:EtOAc = 5:1).

MS (EI, 70 eV): m/z (%): 258 (100, [M^+]), 239 (31), 228 (51), 200 (20), 153 (11), 134 (11), 117 (16), 103 (21), 89 (45), 76 (43), 69 (70), 63 (24).

^{1}H NMR (250 MHz, $CDCl_3$) δ = 8.45 (dd, *J* = 7.6, 0.9 Hz, 1H), 8.28 (dd, *J* = 8.7, 0.9 Hz, 1H), 8.13 (dq, *J* = 14.1, 2.1 Hz, 1H), 7.63 (dd, *J* = 8.7, 7.6 Hz, 1H), 7.21 – 7.06 (m, 1H) ppm.

^{13}C NMR (63 MHz, $CDCl_3$) δ = 147.3, 138.5, 138.4, 135.0 (q, *J* = 7.9 Hz), 127.1, 126.6, 126.6, 122.5 (q, *J* = 269.2 Hz), 114.4 (q, *J* = 36.2 Hz) ppm.

^{19}F NMR (235 MHz, $CDCl_3$) δ = -62.99 ppm.

IR (thin film, ATR): ν = 1680, 1529, 1359, 1327, 1284, 1266, 1251, 1230, 1200, 1118, 954, 904, 841, 816, 775, 736, 705, 680 cm^{-1}.

HRMS m/z [M]$^+$ calculated for $C_9H_5F_3N_4O_2^+$: 258.0365, found: 258.03618.

(40)

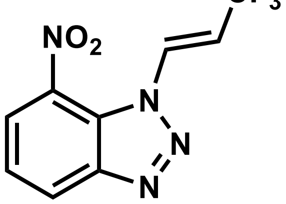

The product was obtained as light brown solid (46.9 mg, 0.182 mmol, 18%). Mp. 107-108 °C. R_f = 0.25 (hexanes:EtOAc = 5:1).

MS (EI, 70 eV): m/z (%): 258 (2, [M^+]), 230 (14), 200 (20), 172 (13), 152 (27), 138 (17), 134 (27), 121 (14), 91 (40), 75 (92), 69 (100), 63 (61).

^{1}H NMR (500 MHz, DMSO-d_6) δ = 8.69 (dd, *J* = 8.3, 0.9 Hz, 1H), 8.53 (dd, *J* = 7.8, 0.9 Hz, 1H), 8.50 (dq, *J* = 14.0, 2.2 Hz, 1H), 7.74 (t, *J* = 8.1 Hz, 1H), 7.10 (dq, *J* = 13.9, 6.9 Hz, 1H) ppm.

^{13}C NMR (126 MHz, DMSO-d_6) δ = 148.8, 135.9, 134.3 (q, *J* = 8.6 Hz), 128.1, 127.5, 125.8, 124., 123.9 (q, *J* = 268.7 Hz), 109.2 (q, *J* = 33.8 Hz) ppm.

^{19}F NMR (235 MHz, $CDCl_3$) δ -62.74 ppm.

IR (thin film, ATR): ν = 1681, 1529, 1346, 1337, 1316, 1294, 1256, 1230, 1158, 1113, 1048, 938, 895, 818, 802, 738, 684 cm^{-1}.

HRMS m/z $[M]^+$ calculated for $C_9H_5F_3N_4O_2^+$: 258.0365, found: 258.03328.

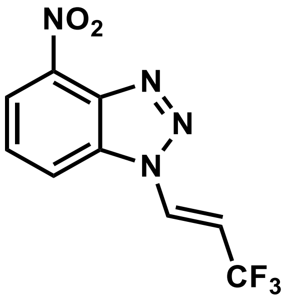

(41)

The product was obtained as yellowish white powder (38.8 mg, 0.150 mmol, 15%). Mp. 132-133 °C. R_f = 0.65 (hexanes:EtOAc = 1:1).

MS (EI, 70 eV): m/z (%): 258 (67, [M^+]), 200 (14), 184 (42), 172 (34), 164 (19), 152 (40), 137 (45), 121 (20), 107 (28), 88 (34), 75 (100), 69 (93), 63 (40).

^{1}H NMR (500 MHz, DMSO-d_6) δ = 8.75 (d, *J* = 8.4 Hz, 1H), 8.73 (dq, *J* = 14.2, 2.2 Hz, 1H), 8.41 (d, *J* = 7.6 Hz, 1H), 7.94 (t, *J* = 8.1 Hz, 1H), 7.24 (dq, *J* = 14.0, 6.9 Hz, 1H) ppm.

^{13}C NMR (126 MHz, DMSO-d_6) δ = 138.6, 138.3, 134.6, 131.1 (q, *J* = 8.1 Hz), 129.5, 124.0 (q, *J* = 268.7 Hz), 123.0, 119.5, 108.2 (q, *J* = 34.5 Hz) ppm.

^{19}F NMR (235 MHz, $CDCl_3$) δ = -62.66 ppm.

IR (thin film, ATR): ν = 1689, 1530, 1342, 1310, 1284, 1269, 1253, 1236, 1180, 1163, 1141, 1117, 1064, 941, 880, 755, 736, 684 cm^{-1}.

HRMS m/z $[M+H]^+$ calculated for $C_9H_6F_3N_4O_2^+$: 259.0443, found: 259.0442.

(*E*)-6-Chloro-9-(3,3,3-trifluoroprop-1-en-1-yl)-9*H*-purine (42)

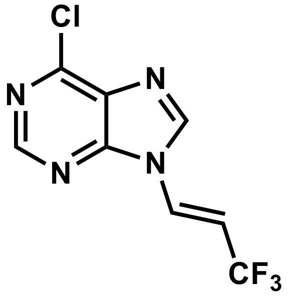

General procedure was followed using 46.4 mg (0.30 mmol) 6-chloro-9*H*-purine, 44.3 mg (0.60 mmol) Li_2CO_3 and 153.8 mg (0.33 mmol) iodonium salt. The solvent was evaporated under reduced pressure and the crude product was purified by column chromatography using hexanes:ethyl acetate eluent gradient. The product was obtained as colorless oil (63.1 mg, 0.254 mmol, 85%). R_f = 0.38 (hexanes:EtOAc = 3:1).

MS (EI, 70 eV): m/z (%): 250 (30, [M^+]), 248 (94, [M^+]), 223 (32), 221 (100), 181 (24), 179 (74), 169 (10), 167 (30), 154 (18), 144 (18), 127 (7), 100 (11), 86 (13), 77 (23), 69 (48).

^{1}H NMR (250 MHz, $CDCl_3$) δ = 8.85 (s, 1H), 8.29 (s, 1H), 7.70 (dt, *J* = 14.5, 2.2 Hz, 1H), 7.17 (dq, *J* = 13.1, 6.2, 5.8 Hz, 1H) ppm.

^{13}C NMR (63 MHz, $CDCl_3$) δ = 153.3, 152.3, 151.2, 143.6, 132.8, 127.8 (q, *J* = 7.9 Hz), 123.0 (q, *J* = 268.9 Hz), 110.1 (q, *J* = 35.5 Hz) ppm.

^{19}F NMR (235 MHz, $CDCl_3$) δ = -62.95 ppm.

IR (thin film, ATR): ν = 1687, 1588, 1566, 1417, 1370, 1342, 1320, 1294, 1264, 1225, 1182, 1118, 951, 936, 798, 790, 656 cm^{-1}.

HRMS m/z $[M+H]^+$ calculated for $C_8H_5F_3N_4Cl^+$: 249.0155, found: 249.0152.

(*E*)-6-Chloro-2-fluoro-9-(3,3,3-trifluoroprop-1-en-1-yl)-9*H*-purine (43)

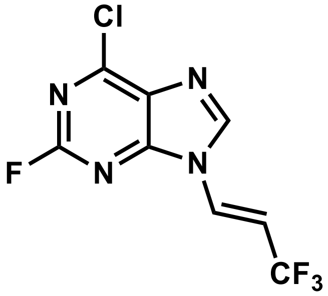

General procedure was followed using 51.8 mg (0.30 mmol) 6-chloro-2-fluoro-9*H*-purine, 44.3 mg (0.60 mmol) Li_2CO_3 and 153.8 mg (0.33 mmol) iodonium salt. The solvent was evaporated under reduced pressure and the crude product was purified by column chromatography using hexanes:ethyl acetate eluent gradient. The product was obtained as white solid (65.6 mg, 0.246 mmol, 82%). Mp. 87-88 °C. R_f = 0.38 (hexanes:EtOAc = 3:1).

MS (EI, 70 eV): m/z (%): 268 (32, [M^+]), 266 (100, [M^+]), 241 (27), 239 (84), 199 (21), 197 (63), 174 (5), 172 (16), 162 (14), 122 (6), 95 (20), 75 (17), 69 (46).

^{1}H NMR (250 MHz, $CDCl_3$) δ = 8.27 (s, 1H), 7.64 (dd, *J* = 14.5, 2.2 Hz, 1H), 7.00 (dq, *J* = 14.5, 6.2 Hz, 1H) ppm.

^{13}C NMR (63 MHz, $CDCl_3$) δ = 157.8 (d, *J* = 223.8 Hz), 154.3 (d, *J* = 17.3 Hz), 152.8 (d, *J* = 16.8 Hz), 144.0 (d, *J* = 3.5 Hz), 131.5 (d, *J* = 5.3 Hz), 127.4 (q, *J* = 8.2 Hz), 122.8 (q, *J* = 269.1 Hz), 110.7 (q, *J* = 35.8 Hz) ppm.

^{19}F NMR (235 MHz, $CDCl_3$) δ = -47.00 (s, 1H), -63.06 (s, 3H) ppm.

IR (thin film, ATR): ν = 1689, 1583, 1514, 1407, 1393, 1361, 1351, 1333, 1312, 1266, 1240, 1184, 1165, 1118, 1005, 949, 923, 789, 660 cm^{-1}.

HRMS m/z $[M]^+$ calculated for $C_8H_3F_4N_4Cl^+$: 265.9982, found: 265.99572.

(*E*)-2,6-Dichloro-9-(3,3,3-trifluoroprop-1-en-1-yl)-9*H*-purine (44)

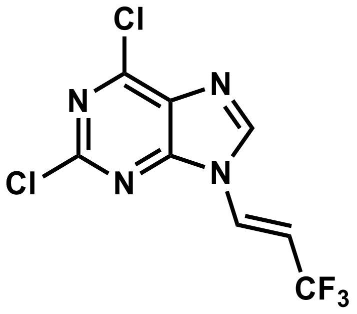

General procedure was followed using 56.7 mg (0.30 mmol) 2,6-dichloro-9*H*-purine, 44.3 mg (0.60 mmol) Li_2CO_3 and 153.8 mg (0.33 mmol) iodonium salt. The solvent was evaporated under reduced pressure and the crude product was purified by column chromatography using hexanes:ethyl acetate eluent gradient. The product was obtained as white crystals (69.8 mg, 0.247 mmol, 83%). Mp. 126-127 °C. R_f = 0.43 (hexanes:EtOAc = 3:1).

MS (EI, 70 eV): m/z (%): 284 (49, [M^+]), 282 (77, [M^+]), 257 (38), 255 (60), 214 (64), 213 (100), 188 (16), 178 (18), 167 (21). 111 (15), 85 (15), 75 (22), 69 (57).

^{1}H NMR (250 MHz, $CDCl_3$) δ = 8.29 (d, *J* = 1.6 Hz, 1H), 7.67 (dt, *J* = 14.5, 2.2 Hz, 1H), 7.01 (dq, *J* = 13.5, 6.2, 5.6 Hz, 1H) ppm.

^{13}C NMR (63 MHz, $CDCl_3$) δ = 154.6, 153.1, 152.3, 143.8, 127.4 (q, *J* = 8.0 Hz), 122.8 (q, *J* = 269.1 Hz), 110.6 (q, *J* = 35.8 Hz) ppm.

^{19}F NMR (235 MHz, $CDCl_3$) δ = -62.96 ppm.

IR (thin film, ATR): ν = 1691, 1588, 1558, 1374, 1350, 1329, 1264, 1217, 1184, 1163, 1137, 1122, 949, 874, 815, 734, 703, 684, 664 cm^{-1}.

HRMS m/z $[M+H]^+$ calculated for $C_8H_4N_4Cl_2F_3^+$: 282.9765, found: 282.9772.

(*E*)-2-(3,3,3-Trifluoroprop-1-en-1-yl)isoindoline-1,3-dione (45)

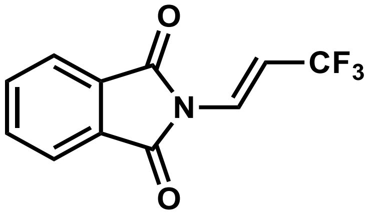

General procedure was followed using 44.1 mg (0.30 mmol) phthalimide, 44.3 mg (0.60 mmol) Li_2CO_3 and 153.8 mg (0.33 mmol) iodonium salt. The solvent was evaporated under reduced pressure and the crude product was purified by column chromatography using hexanes:ethyl acetate eluent gradient. The product was obtained as white powder (53.7 mg, 0.238 mmol, 80%). Mp. 104-105 °C. R_f = 0.63 (hexanes:EtOAc = 4:1).

MS (EI, 70 eV): m/z (%): 241 (47 [M^+]), 222 (11), 172 (100), 104 (36), 76 (56), 66 (11), 50 (25).

^{1}H NMR (250 MHz, $CDCl_3$) δ = 7.92 (td, *J* = 5.2, 2.0 Hz, 2H), 7.83 (td, *J* = 5.2, 2.0 Hz, 2H), 7.42 (dq, *J* = 14.8, 2.1 Hz, 1H), 6.85 (dq, *J* = 14.8, 6.5 Hz, 1H) ppm.

^{13}C NMR (63 MHz, $CDCl_3$) δ = 165.6, 135.4, 131.4, 125.2 (q, *J* = 8.2 Hz), 124.4, 124.0 (q, *J* = 268.4 Hz), 106.8 (q, *J* = 34.7 Hz) ppm.

^{19}F NMR (235 MHz, $CDCl_3$) δ = -62.72 ppm.

IR (thin film, ATR): 3067, 2922, 1801, 1784, 1720, 1668, 1401, 1337, 1298, 1250, 1215, 1143, 1098, 1074, 989, 959, 894, 853, 833, 790, 714, 678, 601, 562, 533, 523, 423.

HRMS m/z $[M]^+$ calculated for $C_{11}H_7F_3NO_2^+$: 241.0351, found: 241.03276.

(*E*)-5,5-Diphenyl-3-(3,3,3-trifluoroprop-1-en-1-yl)imidazolidine-2,4-dione (46)

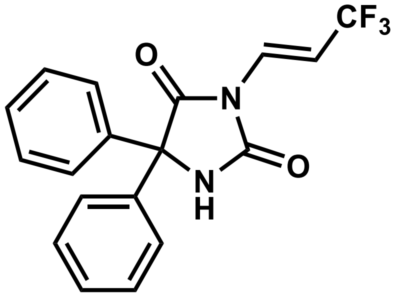

General procedure was followed using 75.7 mg (0.30 mmol) 5,5-diphenylimidazolidine-2,4-dione (Phenytoin), 44.3 mg (0.60 mmol) Li_2CO_3 and 153.8 mg (0.33 mmol) iodonium salt. The solvent was evaporated under reduced pressure and the crude product was purified by column chromatography using hexanes:ethyl acetate eluent gradient.

The product1 **(46)** was obtained as white powder (61.5 mg, 0.178 mmol, 60%). Mp. 133-134 °C. R_f = 0.30 (hexanes:EtOAc = 5:1).

MS (EI, 70 eV): m/z (%): 346 (14, [M^+]), 318 (28), 180 (100), 165 (21), 104 (37), 77 (26).

^{1}H NMR (250 MHz, $CDCl_3$) δ = 7.86 (s, 1H), 7.40 (s, 9H), 7.35 – 7.22 (m, 1H), 6.87 (dq, *J* = 14.8, 6.5 Hz, 1H) ppm.

^{13}C NMR (63 MHz, $CDCl_3$) δ = 171.2, 153.8, 138.2, 129.2, 129.2, 126.9, 125.5 (q, *J* = 8.3 Hz), 123.7 (q, *J* = 268.7 Hz), 107.8 (q, *J* = 34.8 Hz), 69.7 ppm.

^{19}F NMR (235 MHz, $CDCl_3$) δ = -62.99 ppm.

IR (thin film, ATR): ν = 1734, 1678, 1422, 1344, 1305, 1264, 1154, 1124, 964, 899, 734, 703, 669 cm^{-1}.

HRMS m/z $[M]^+$ calculated for $C_{18}H_{13}F_3N_2O_2^+$: 346.0929, found: 346.09052.

Unsuccessful attempts

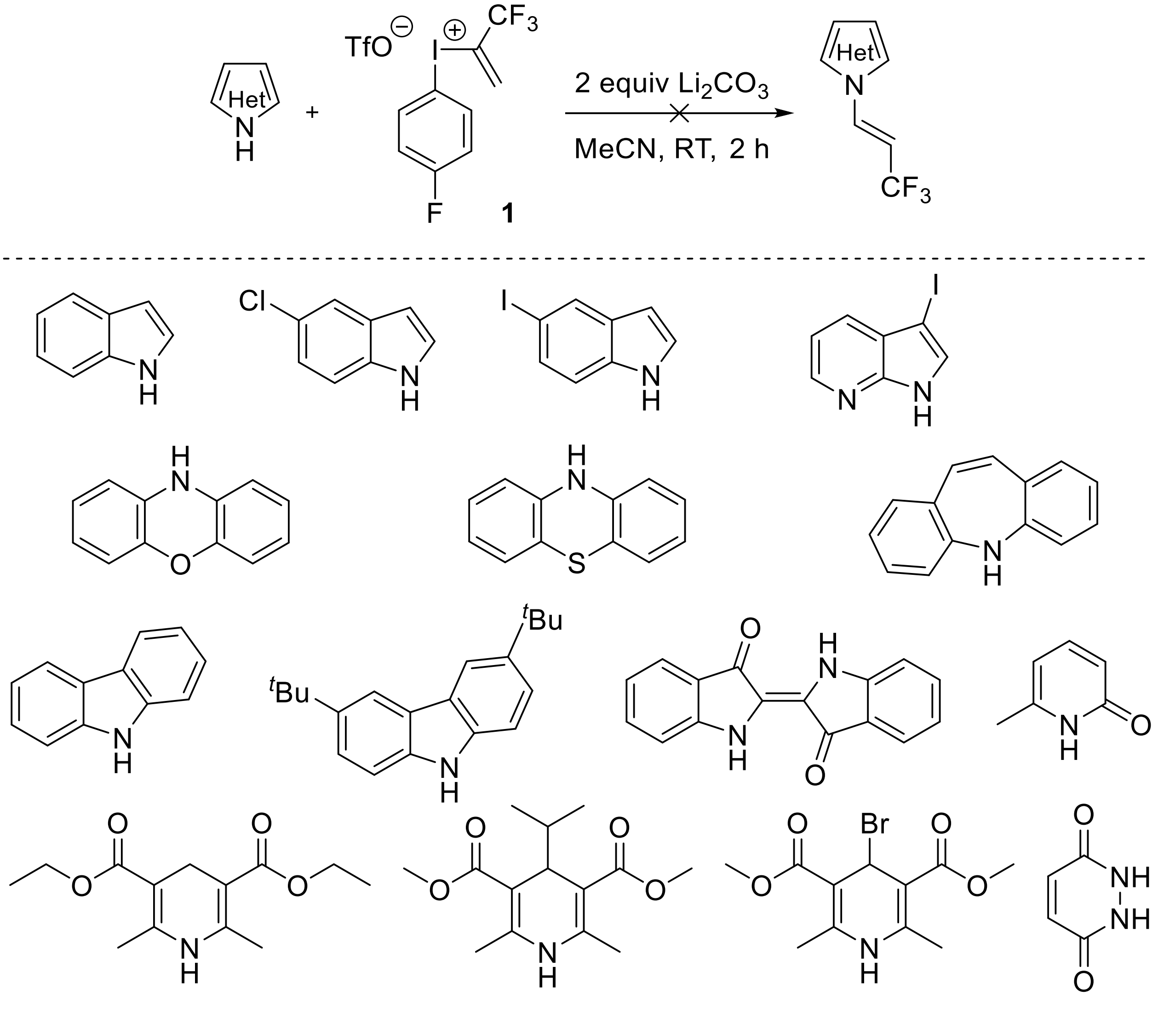

5-Phenyl-3-(trifluoromethyl)-1*H*-pyrazole (2r)

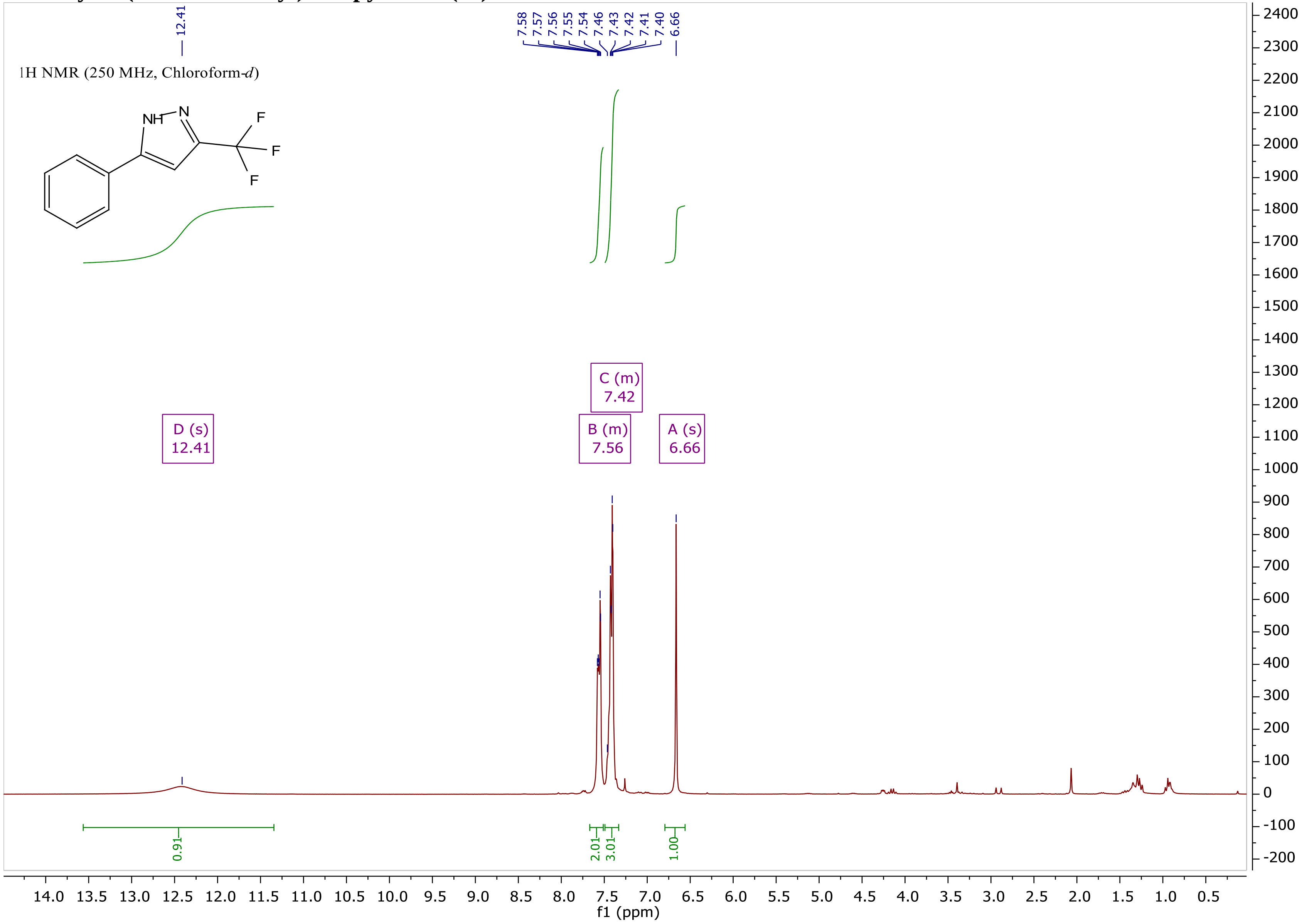

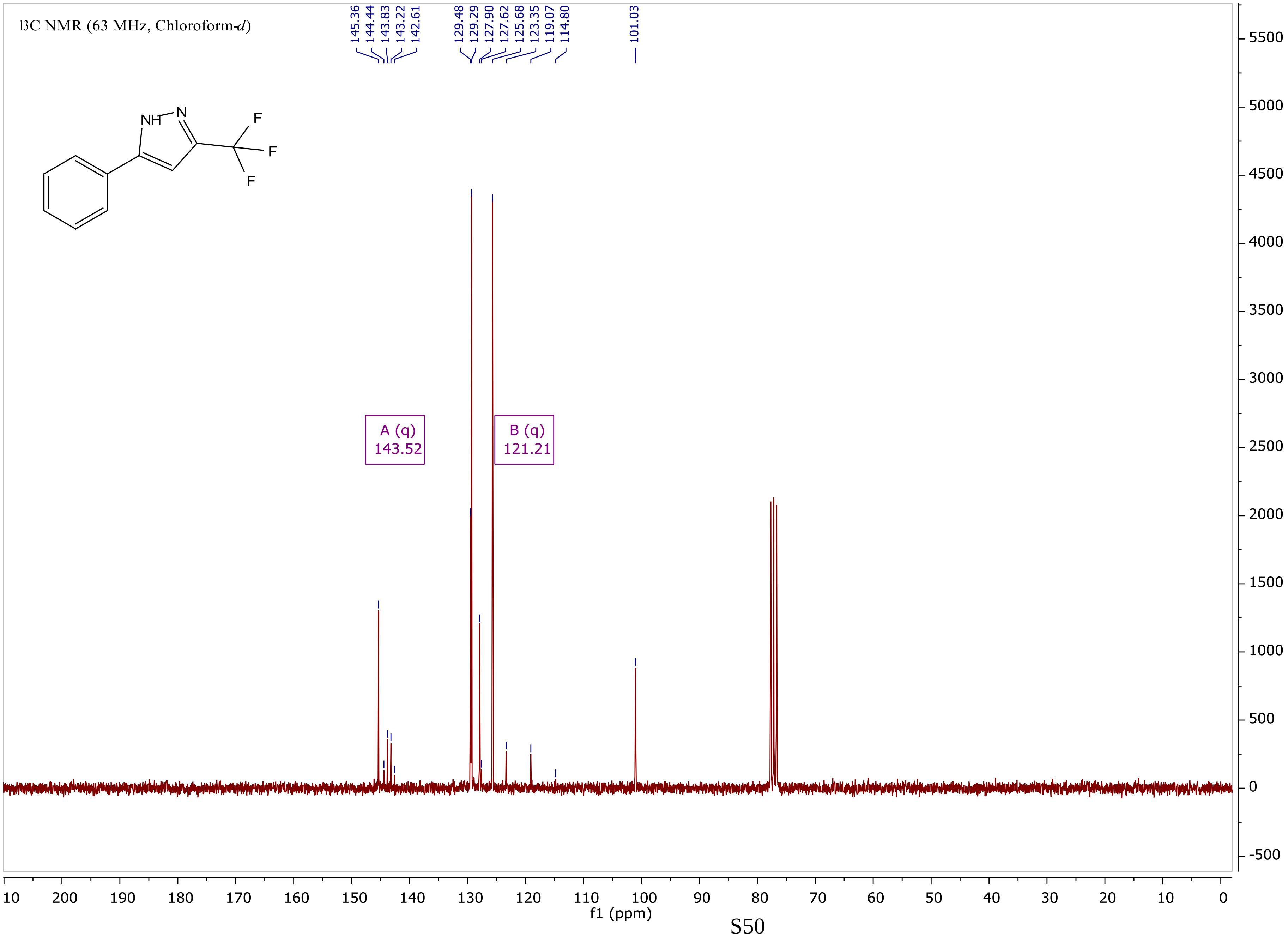
13C NMR (63 MHz, Chloroform-d)
NH
N
F
F
F
145.36
144.44
143.83
143.22
142.61
129.48
129.29
127.90
127.62
125.68
123.35
119.07
114.80
101.03
A (q)
143.52
B (q)
121.21
f1 (ppm)

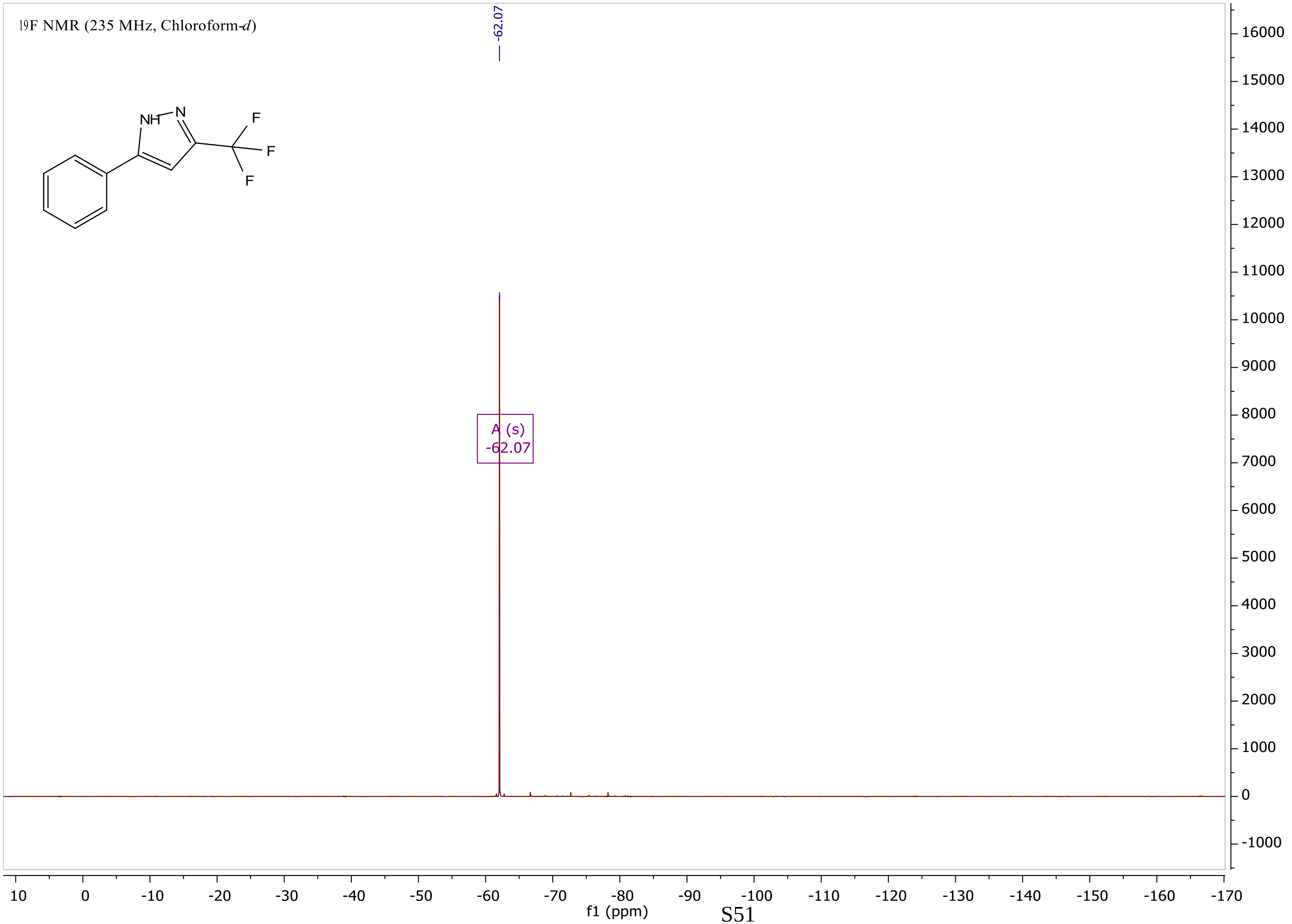
19F NMR (235 MHz, Chloroform-d)
-62.07
NH
N
F
F
F
A (s)
-62.07
f1 (ppm)

Ethyl (*E*)-*N*-(4-cyano-1*H*-pyrazol-3-yl)acetimidate (2s)

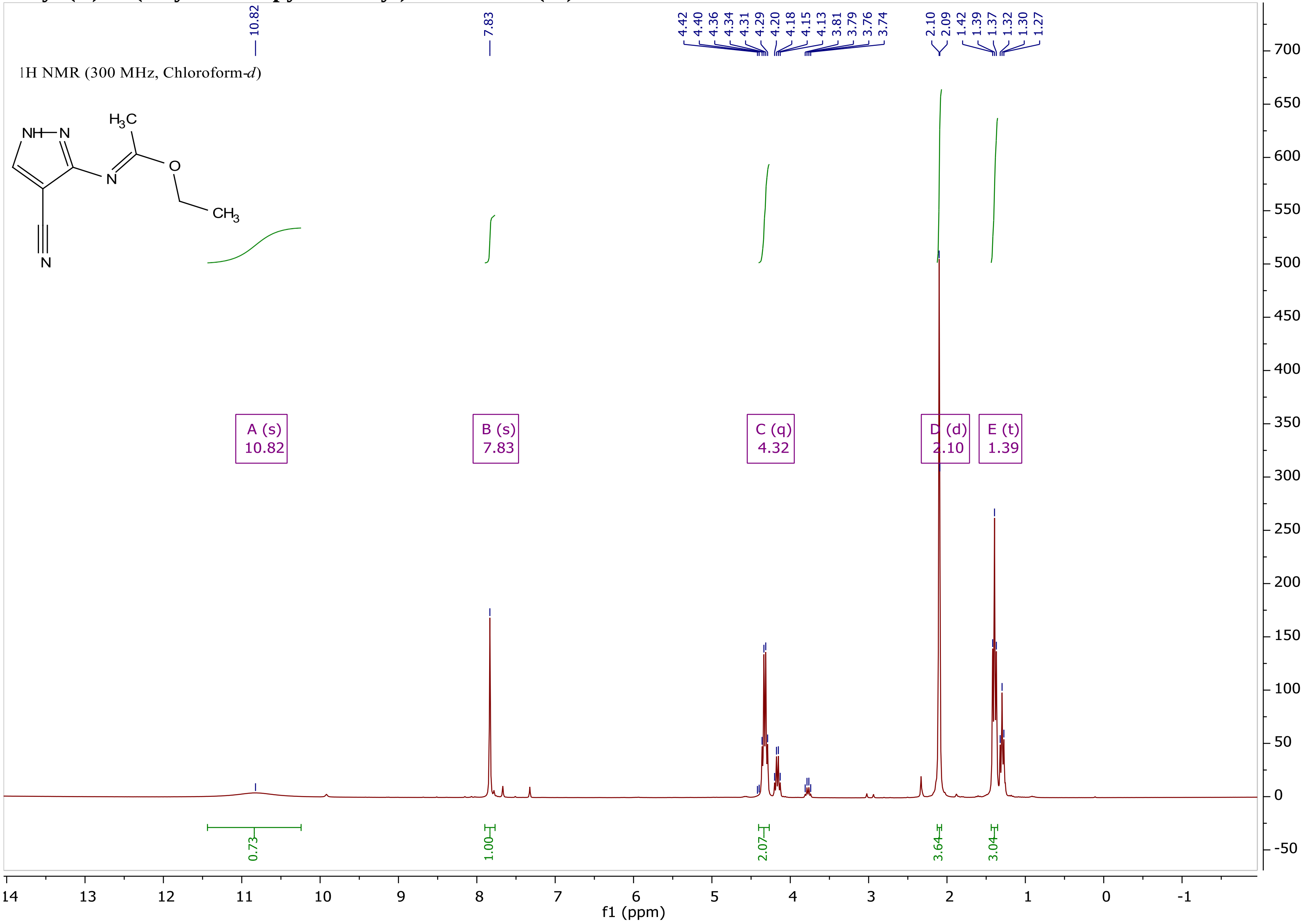

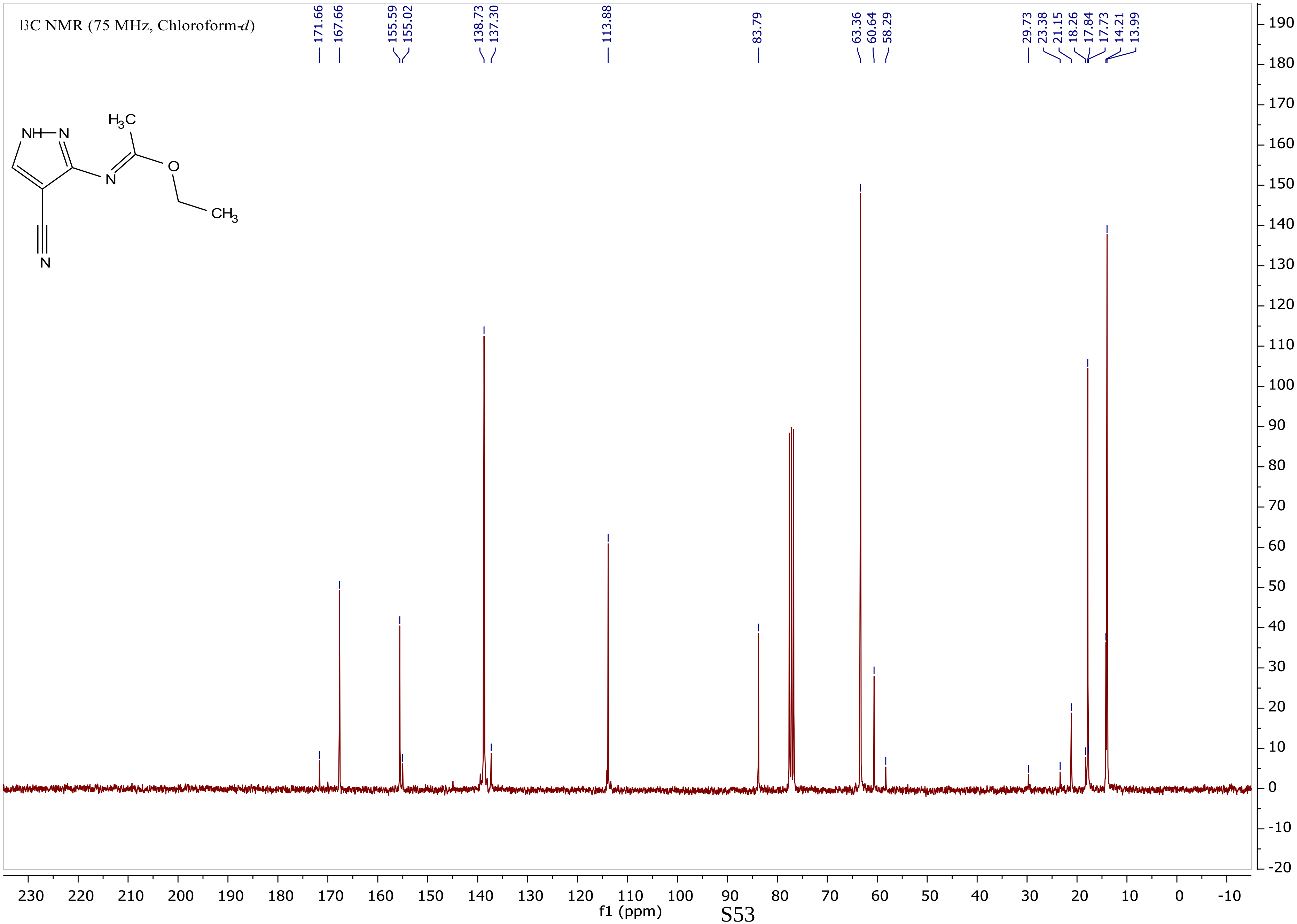
13C NMR (75 MHz, Chloroform-d)
171.66
167.66
155.59
155.02
138.73
137.30
113.88
83.79
63.36
60.64
58.29
29.73
23.38
21.15
18.26
17.84
17.73
14.21
13.99
f1 (ppm)

5-Phenyl-1*H*-tetrazole (2x)

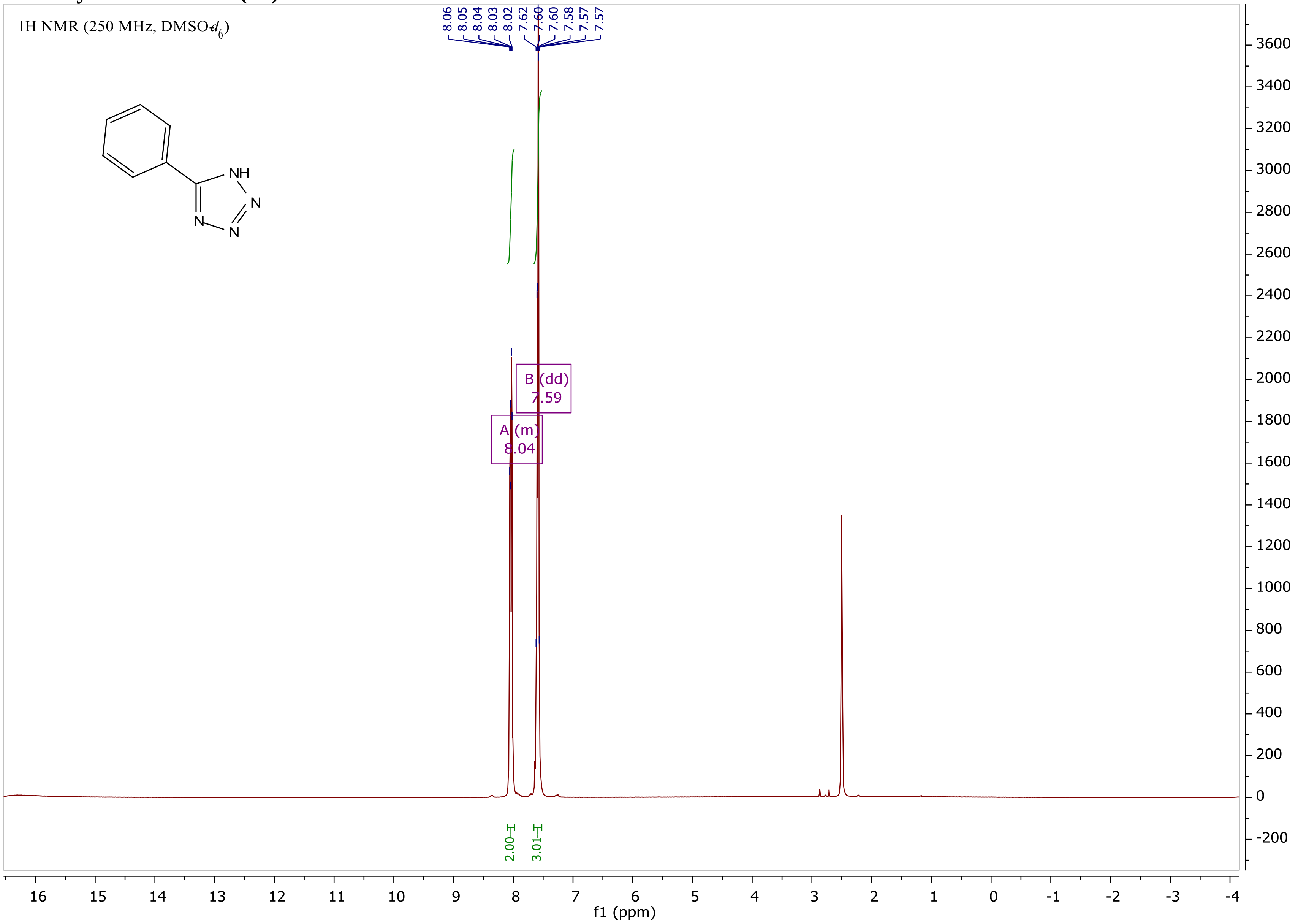

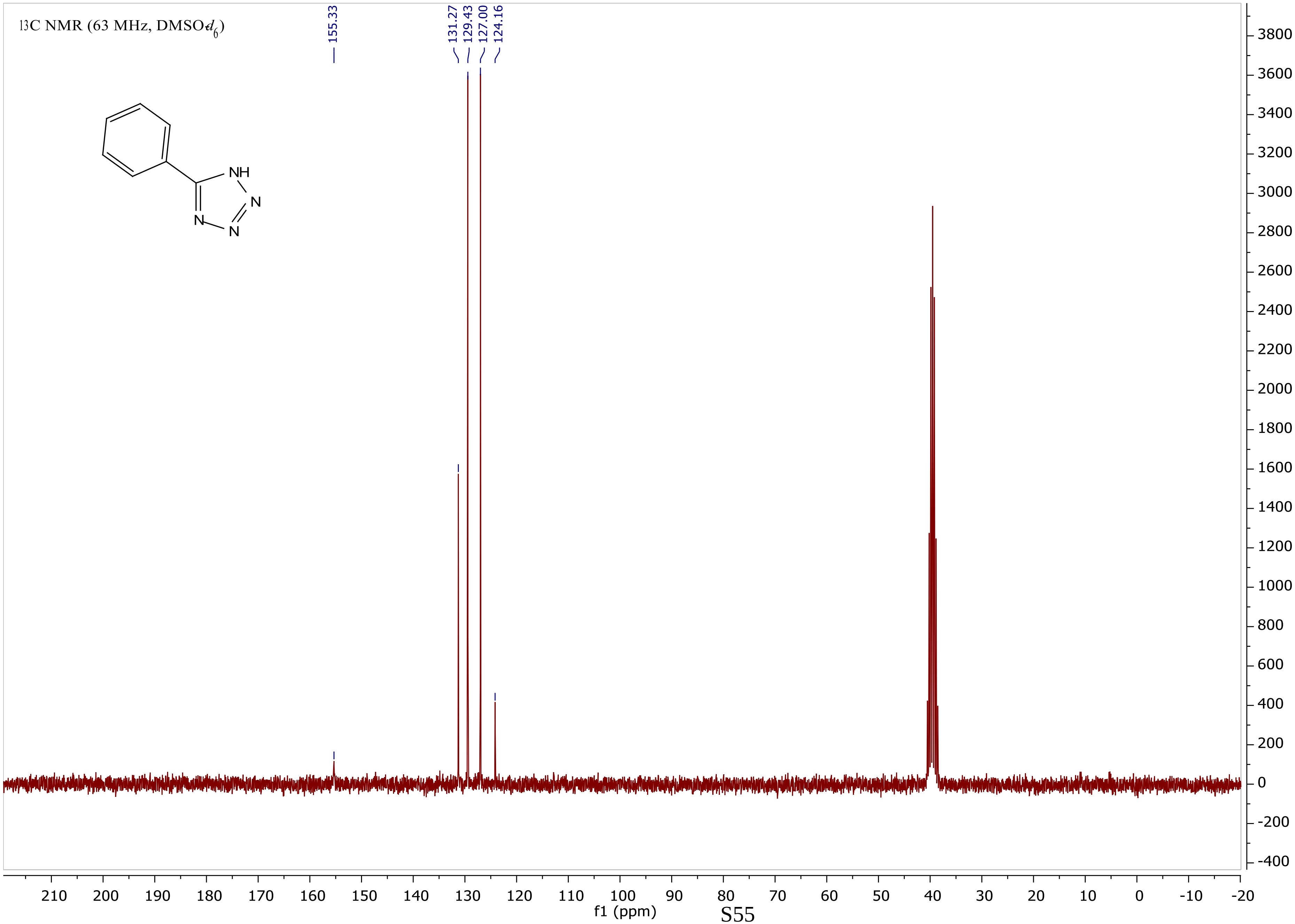

13C NMR (63 MHz, DMSO-d6)
155.33
131.27
129.43
127.00
124.16
NH
N
N
N
f1 (ppm)

Ethyl 1*H*-indazole-3-carboxylate (2z)

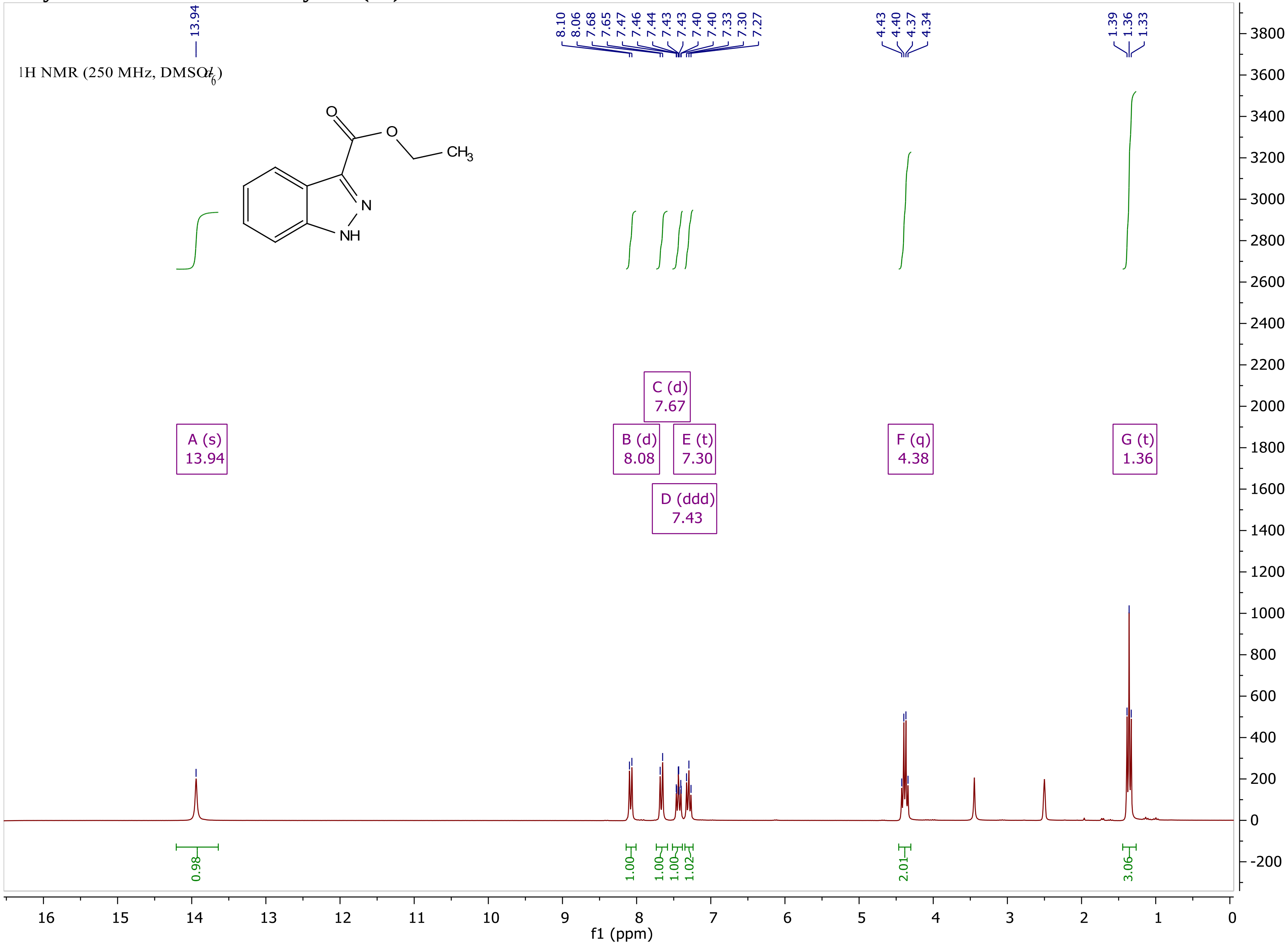

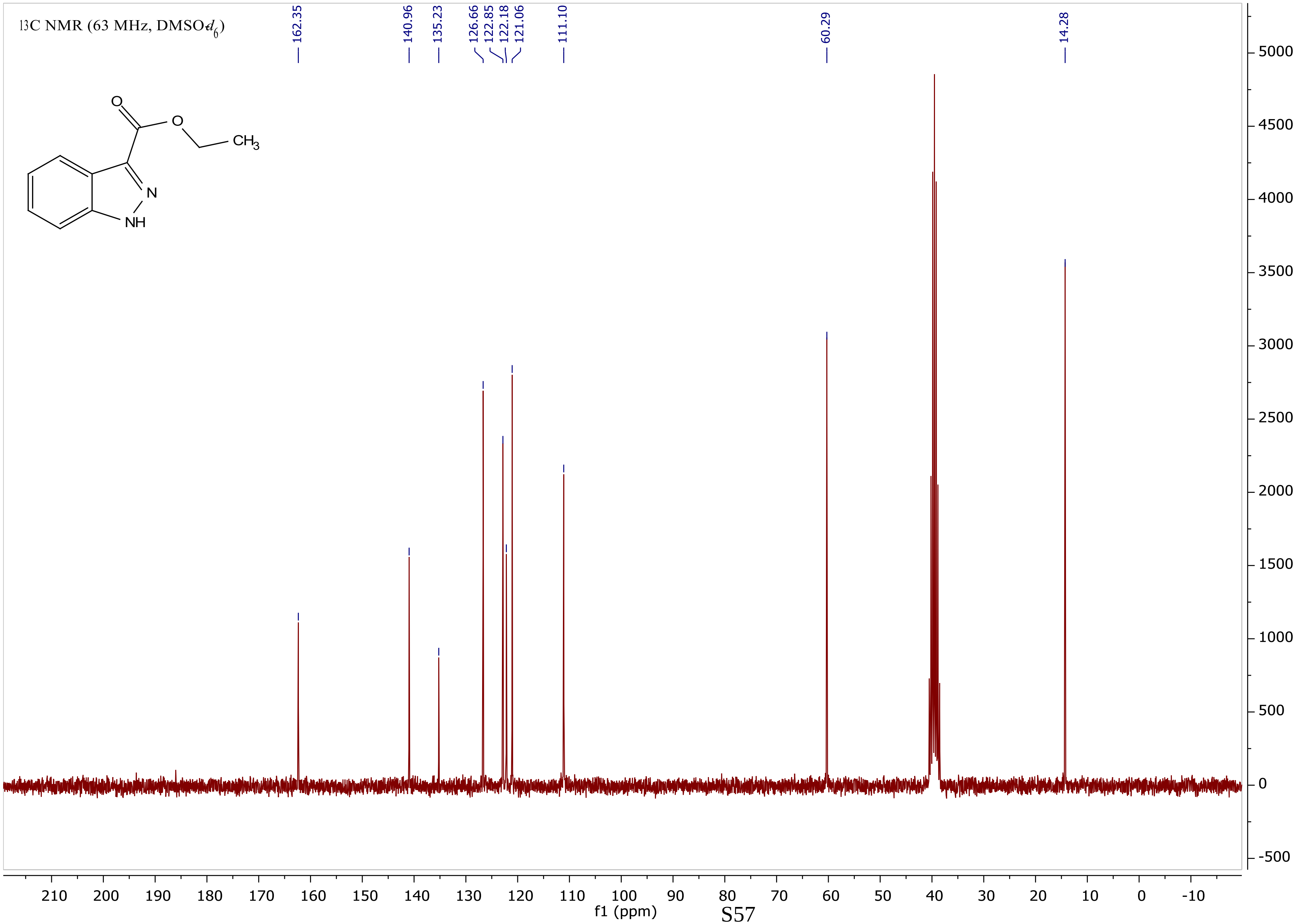
13C NMR (63 MHz, DMSO-d_6)
O
O
CH3
N
NH
162.35
140.96
135.23
126.66
122.85
122.18
121.06
111.10
60.29
14.28
5000
4500
4000
3500
3000
2500
2000
1500
1000
500
0
-500
210
200
190
180
170
160
150
140
130
120
110
100
90
80
70
60
50
40
30
20
10
0
-10
f1 (ppm)

5-((tert-Butyldimethylsilyl)oxy)-1*H*-indazole (2ac)

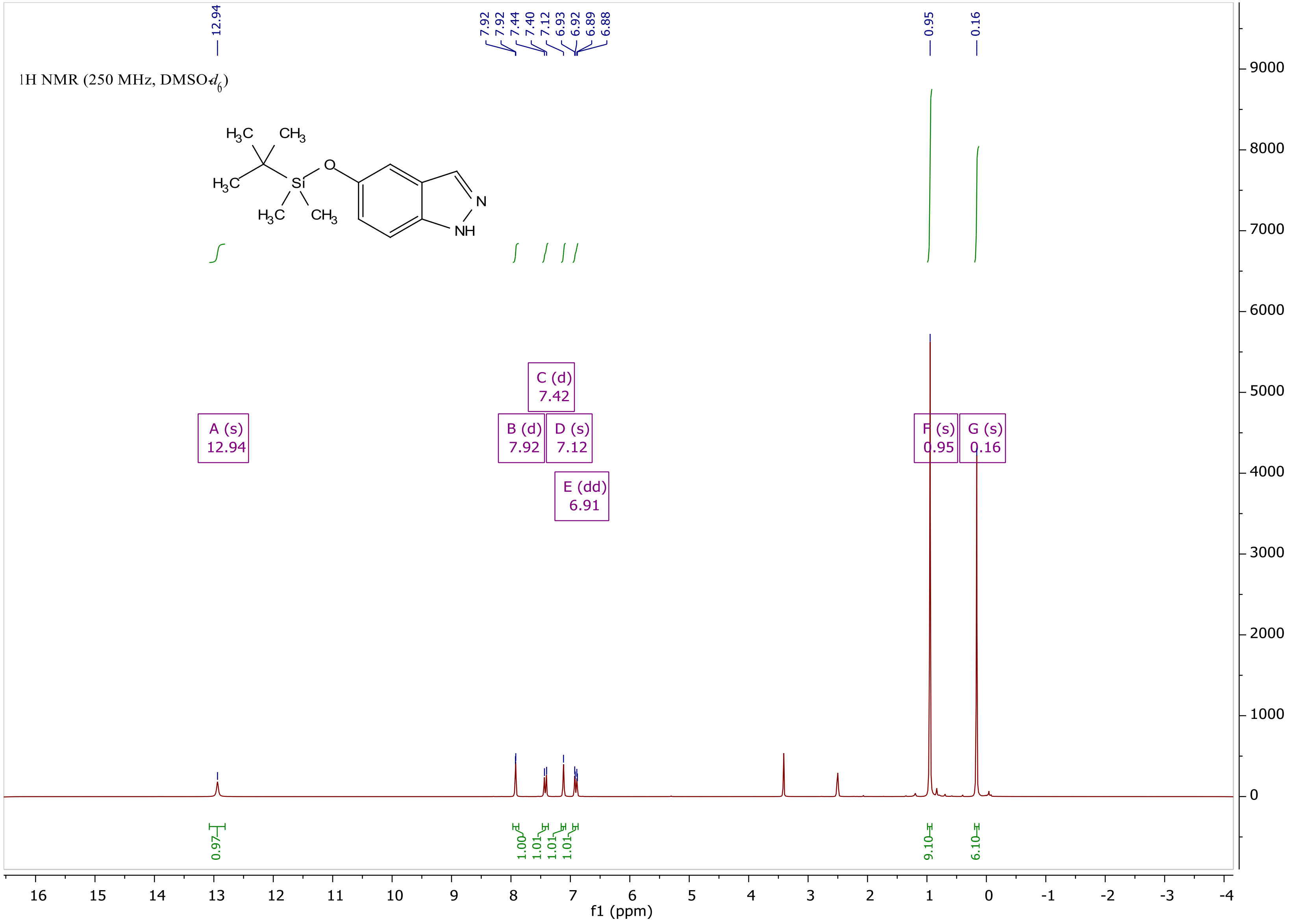

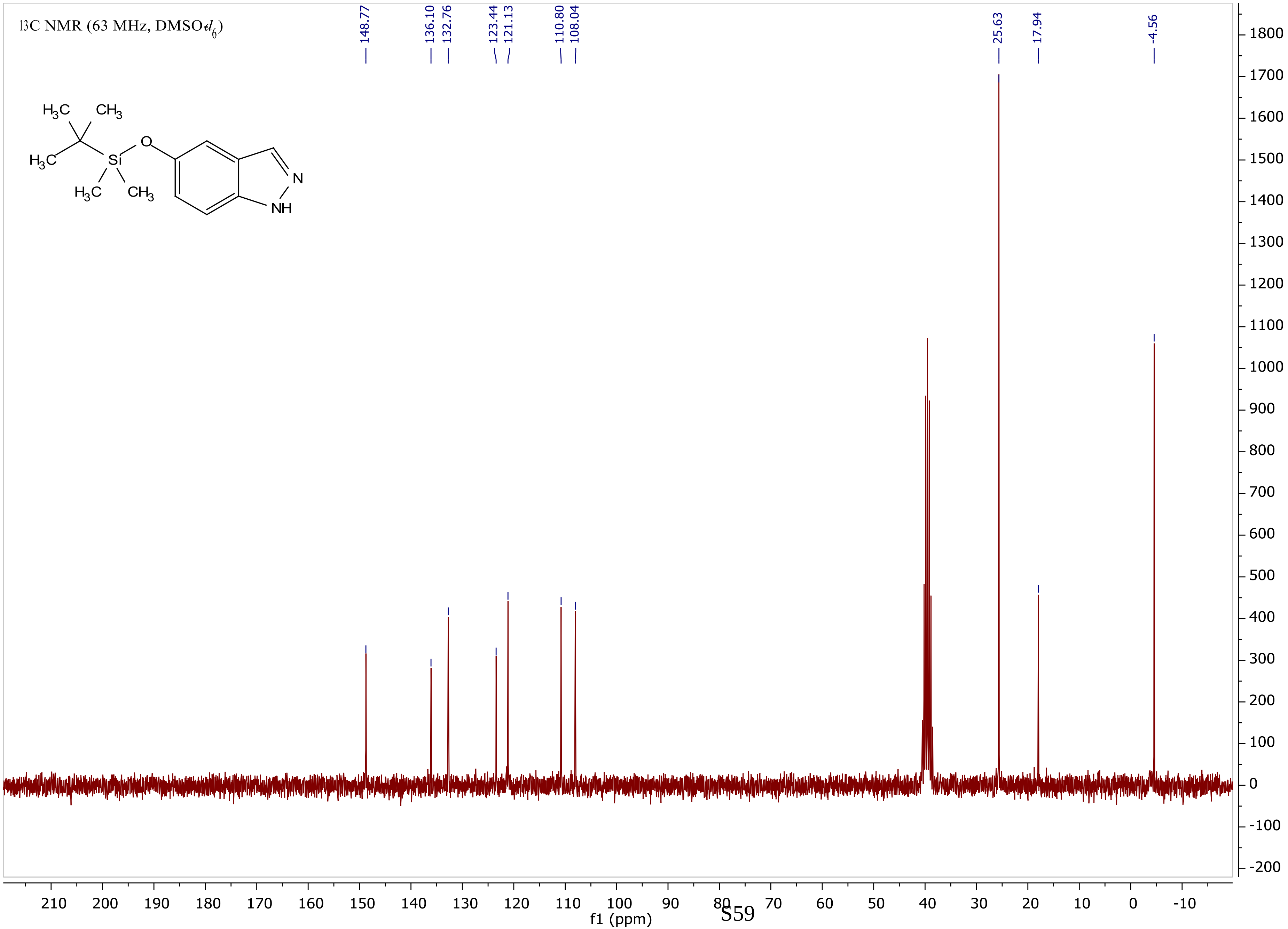
13C NMR (63 MHz, DMSO-d_6)
148.77
136.10
132.76
123.44
121.13
110.80
108.04
25.63
17.94
-4.56
H3C
CH3
H3C
Si
O
H3C
CH3
N
NH
f1 (ppm)

N-deutero-1*H*-benzotriazole ([D]2a)

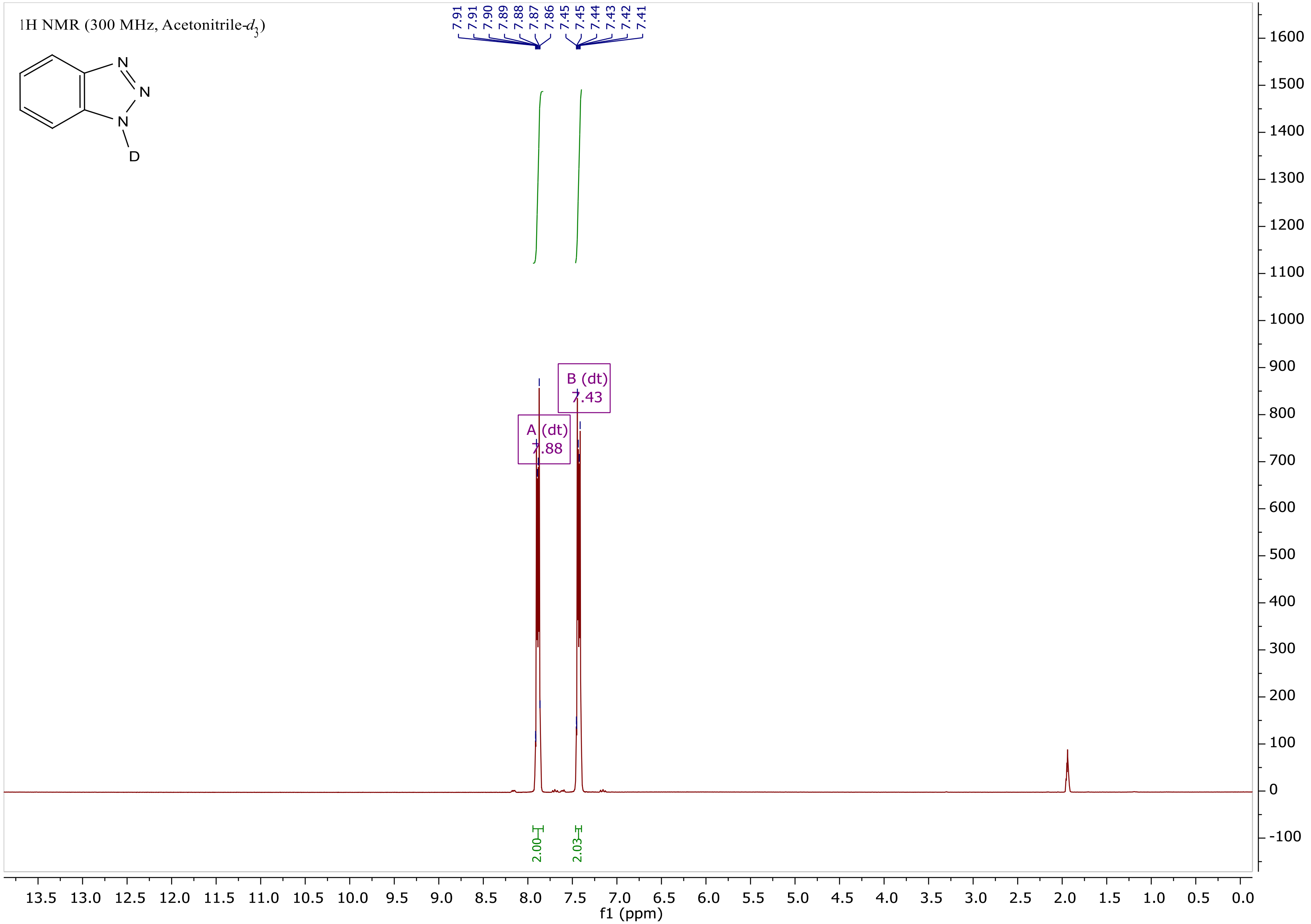

13C NMR (75 MHz, Acetonitrile-d_3)

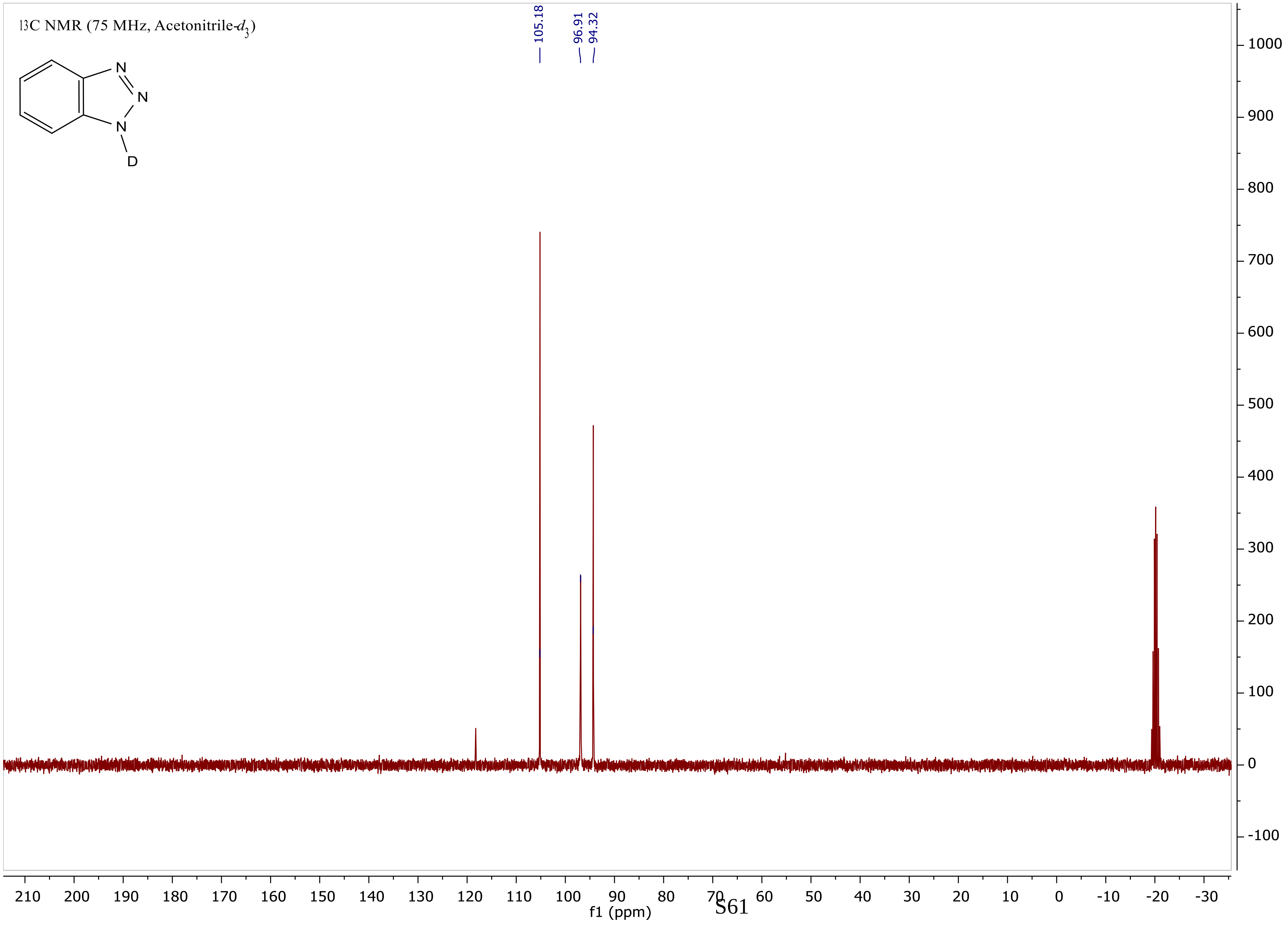

(*E*)-1-(3,3,3-Trifluoroprop-1-en-1-yl)-1*H*-benzo[d][1,2,3]triazole (3)

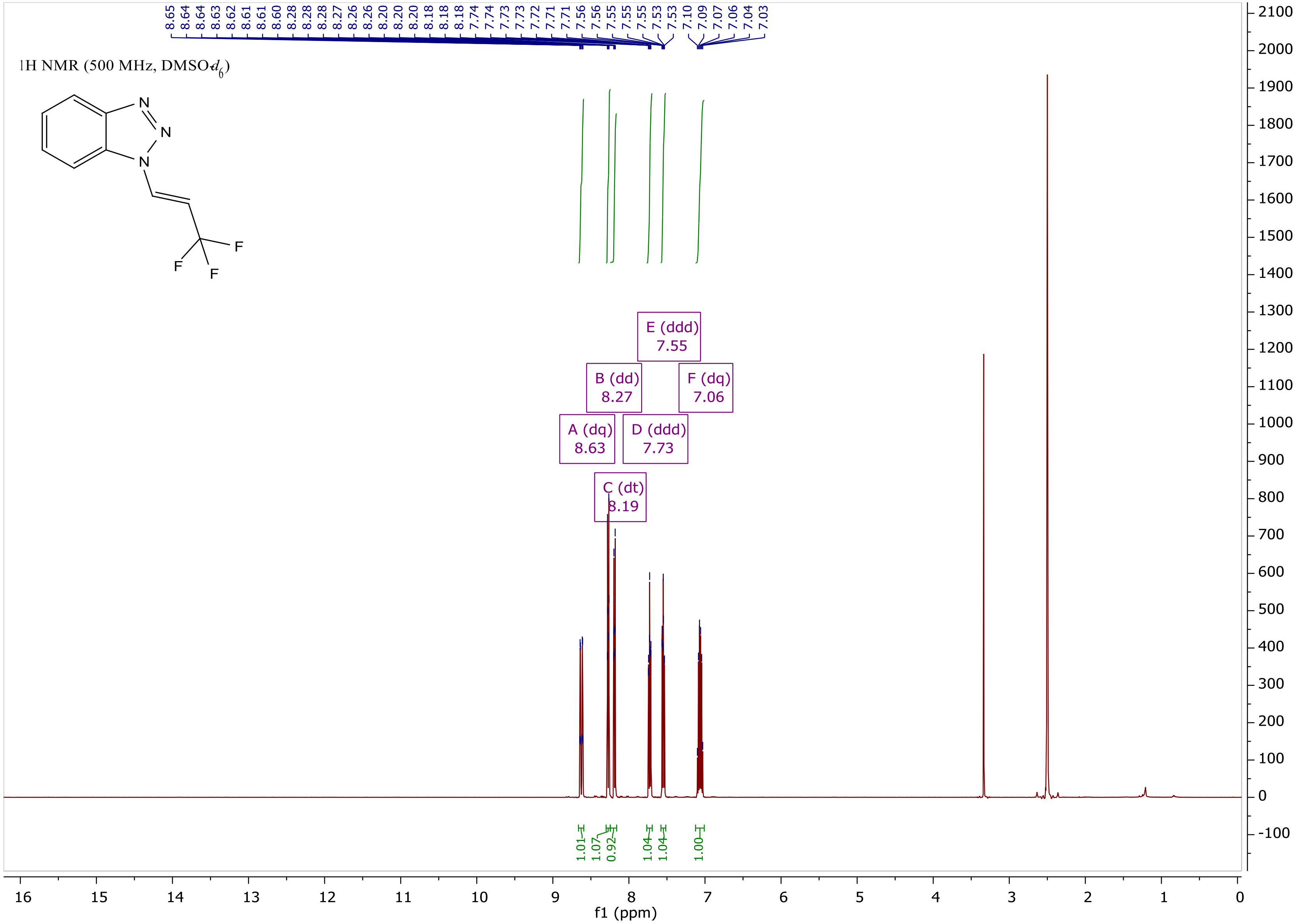

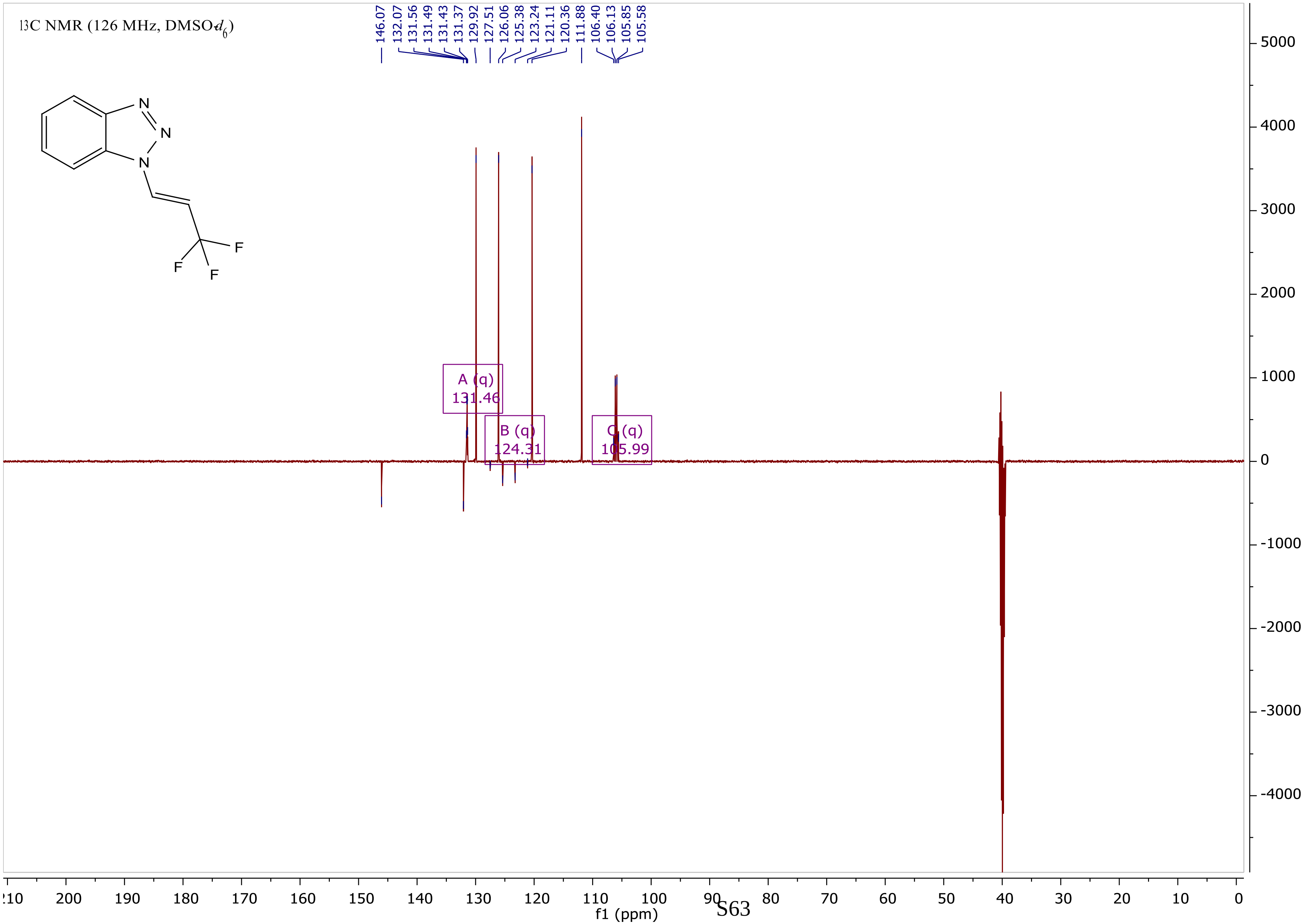
13C NMR (126 MHz, DMSO-d_6)
N
N
N
F
F
F
146.07
132.07
131.56
131.49
131.43
131.37
129.92
127.51
126.06
125.38
123.24
121.11
120.36
111.88
106.40
106.13
105.85
105.58
A (q)
131.46
B (q)
124.31
C (q)
105.99
5000
4000
3000
2000
1000
0
-1000
-2000
-3000
-4000
210
200
190
180
170
160
150
140
130
120
110
100
90
80
70
60
50
40
30
20
10
0
f1 (ppm)

19F NMR (235 MHz, Chloroform-*d*)

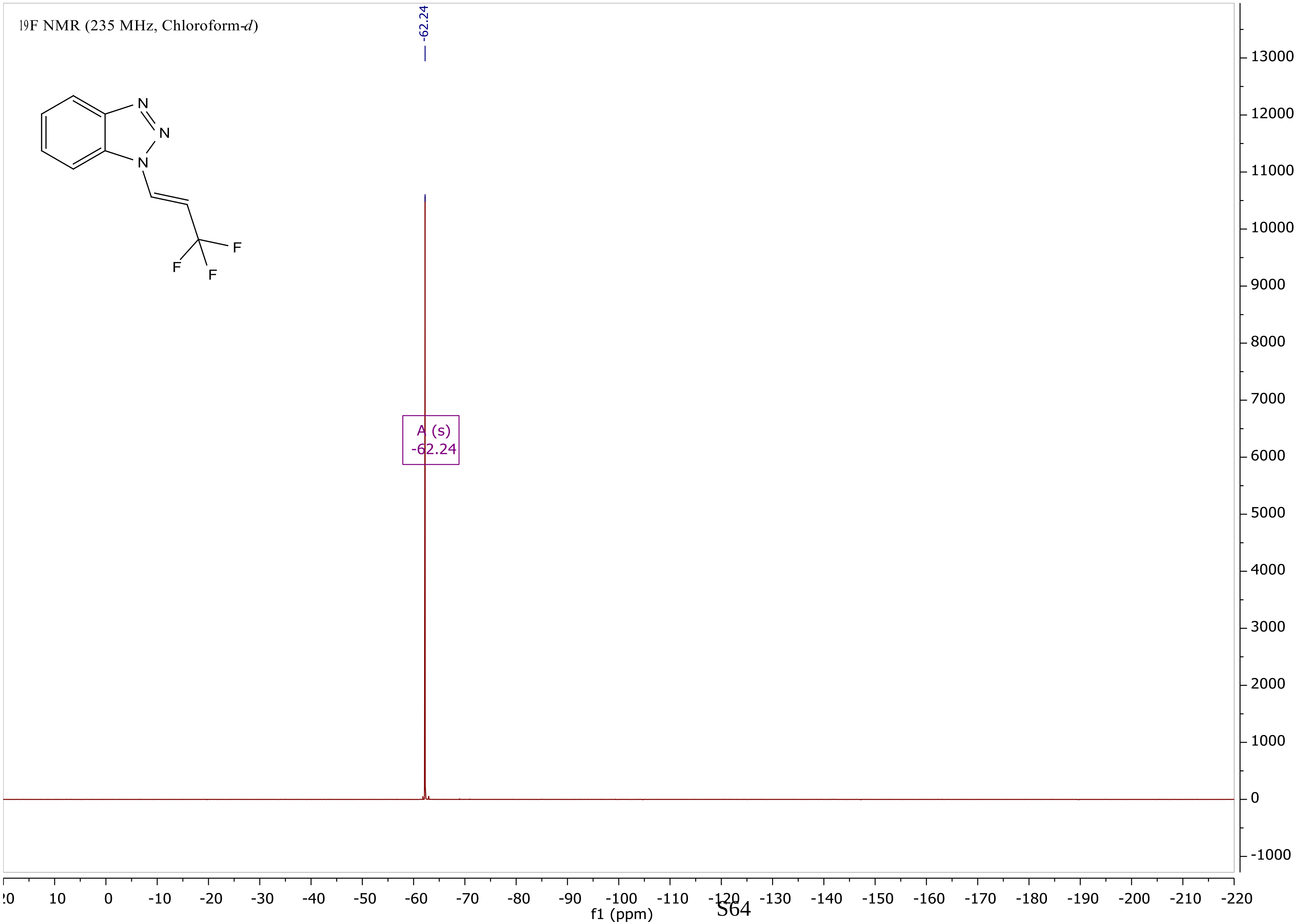

(*E*)-4-Bromo-1-(3,3,3-trifluoroprop-1-en-1-yl)-1*H*-pyrazole (4)

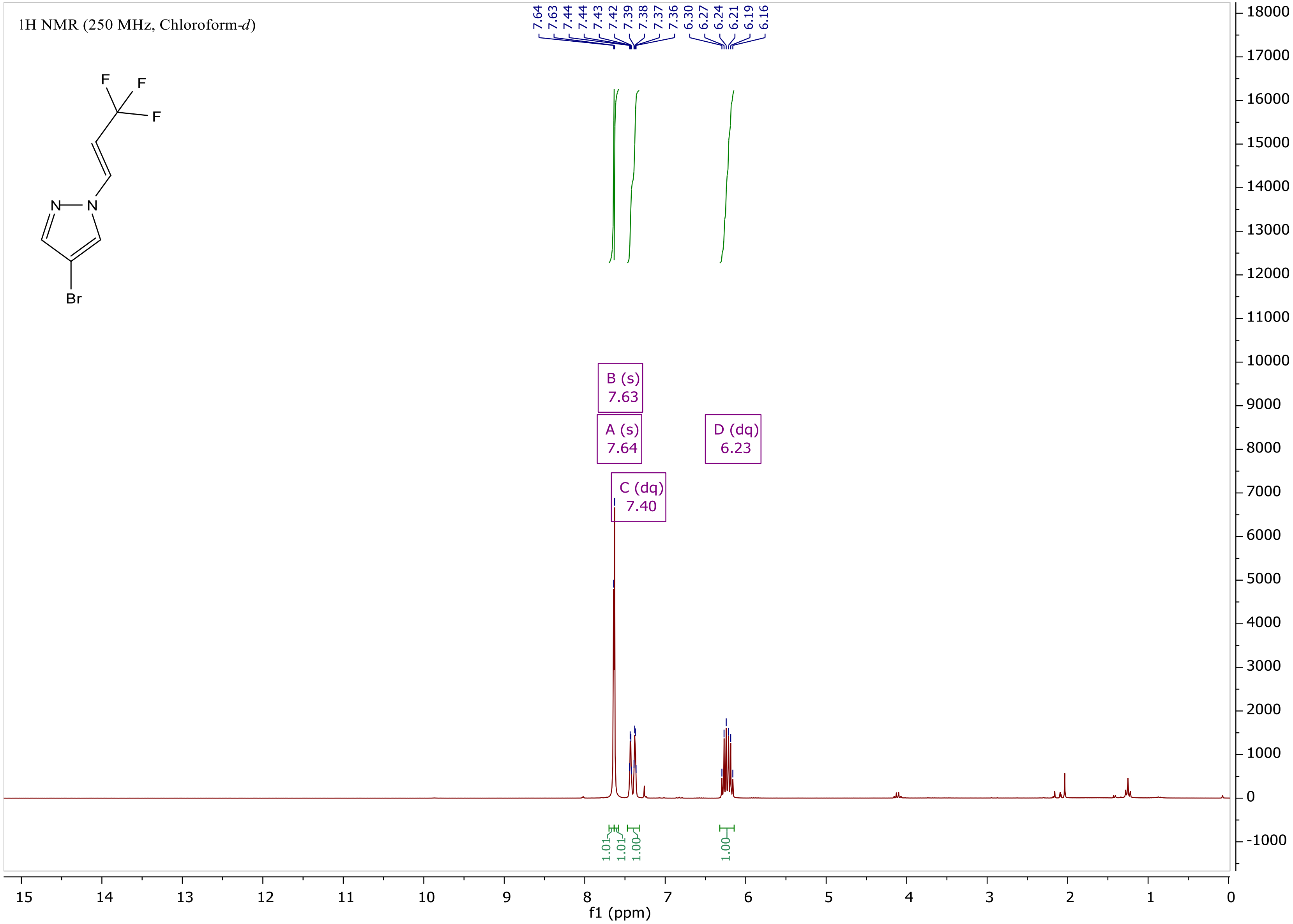

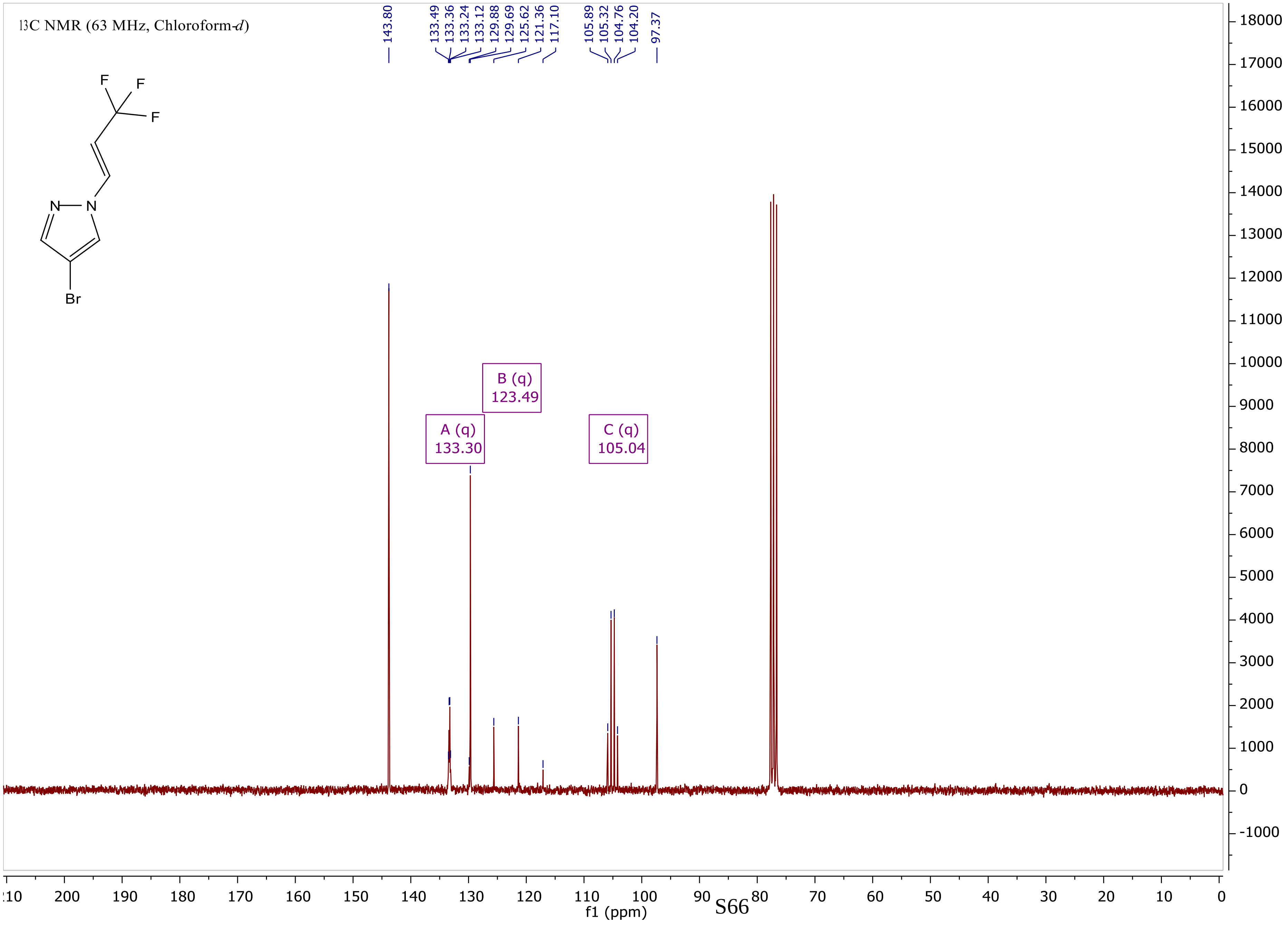

13C NMR (63 MHz, Chloroform-d)
F
F
F
N
N
Br
143.80
133.49
133.36
133.24
133.12
129.88
129.69
125.62
121.36
117.10
105.89
105.32
104.76
104.20
97.37
B (q)
123.49
A (q)
133.30
C (q)
105.04
f1 (ppm)

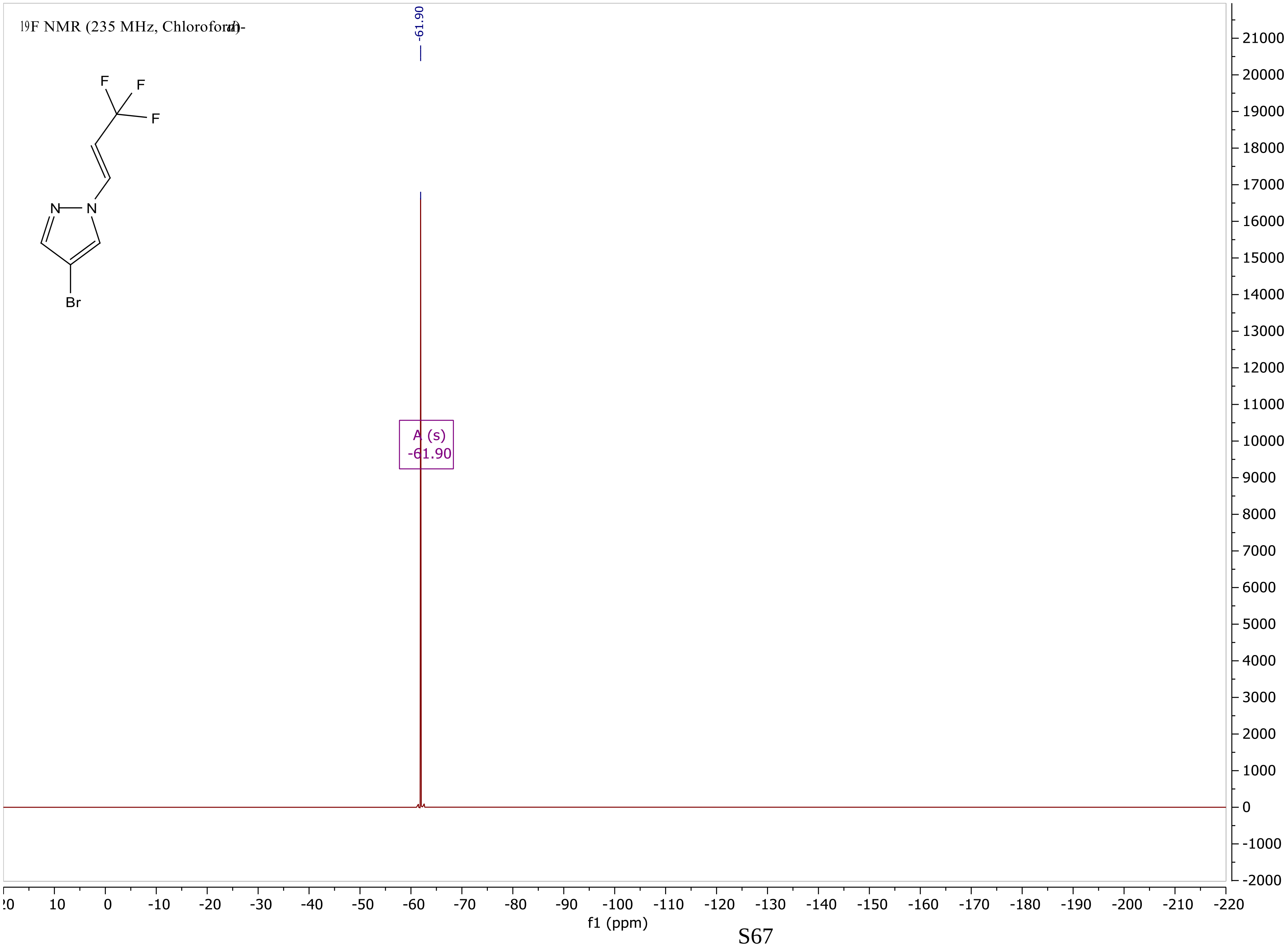
19F NMR (235 MHz, Chloroform-d)
-61.90
A (s)
-61.90
F
F
F
N
N
Br
f1 (ppm)

(*E*)-3,5-Dimethyl-1-(3,3,3-trifluoroprop-1-en-1-yl)-1*H*-pyrazole (5)

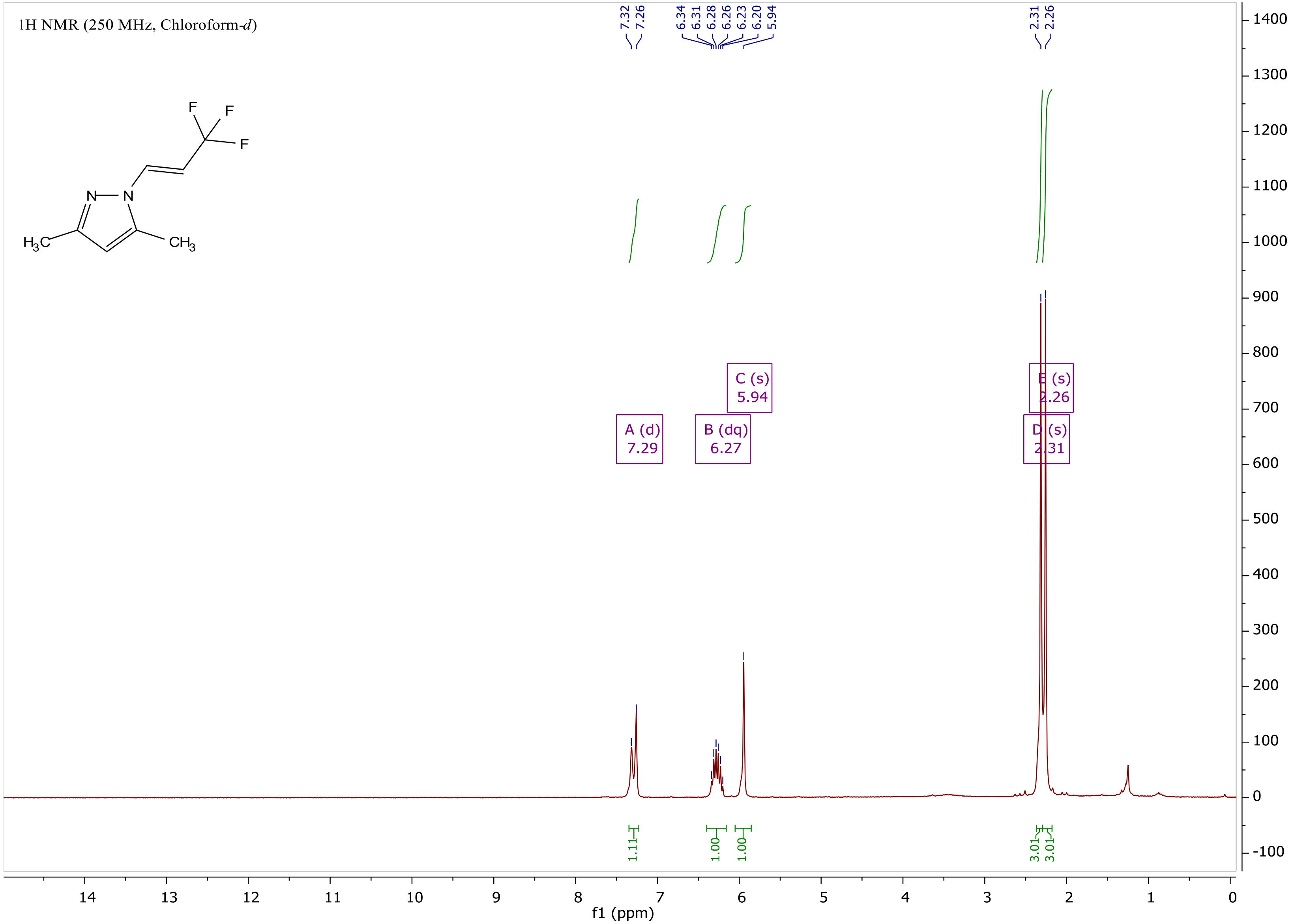

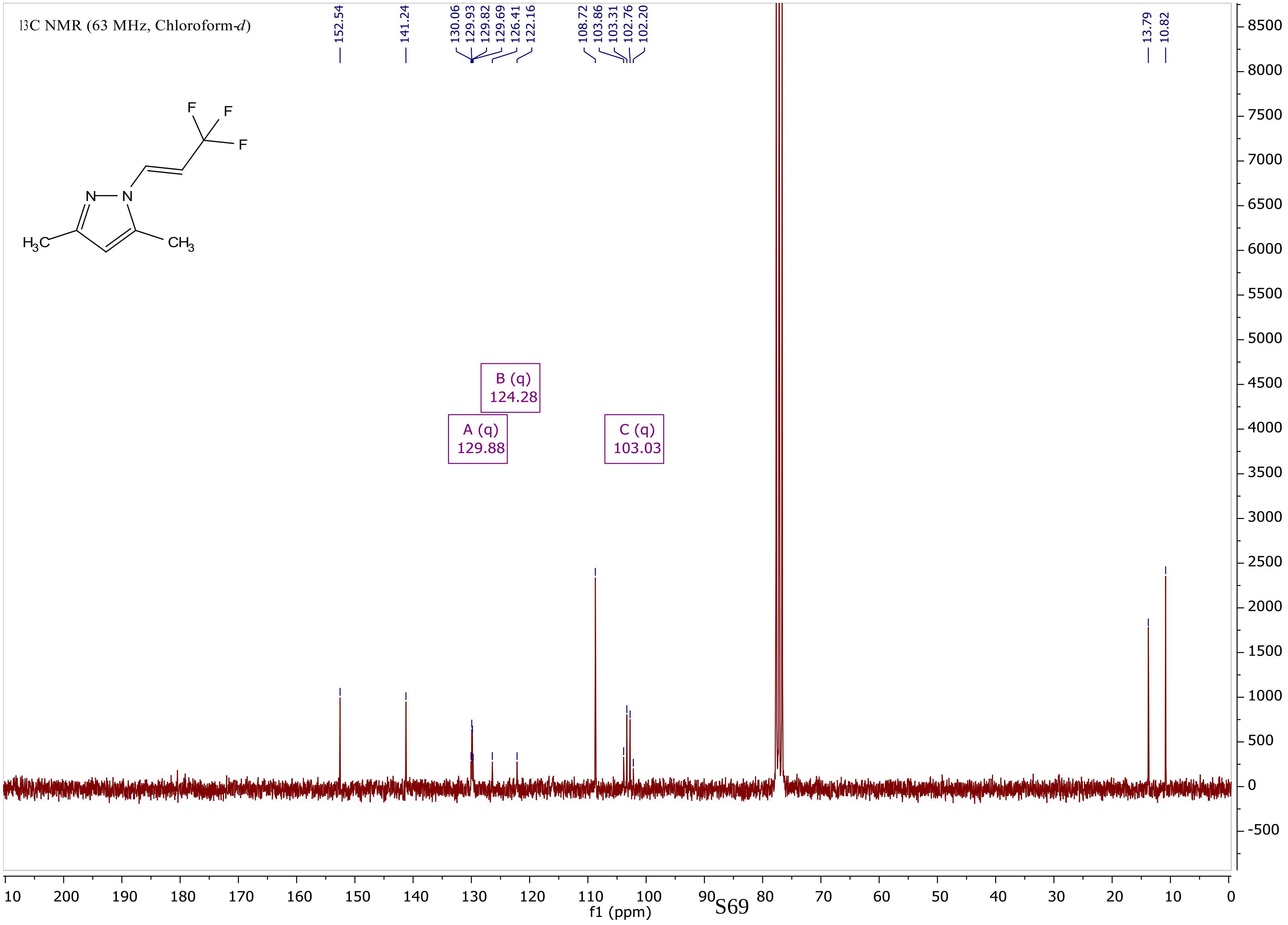
13C NMR (63 MHz, Chloroform-d)
152.54
141.24
130.06
129.93
129.82
129.69
126.41
122.16
108.72
103.86
103.31
102.76
102.20
13.79
10.82
B (q)
124.28
A (q)
129.88
C (q)
103.03
f1 (ppm)

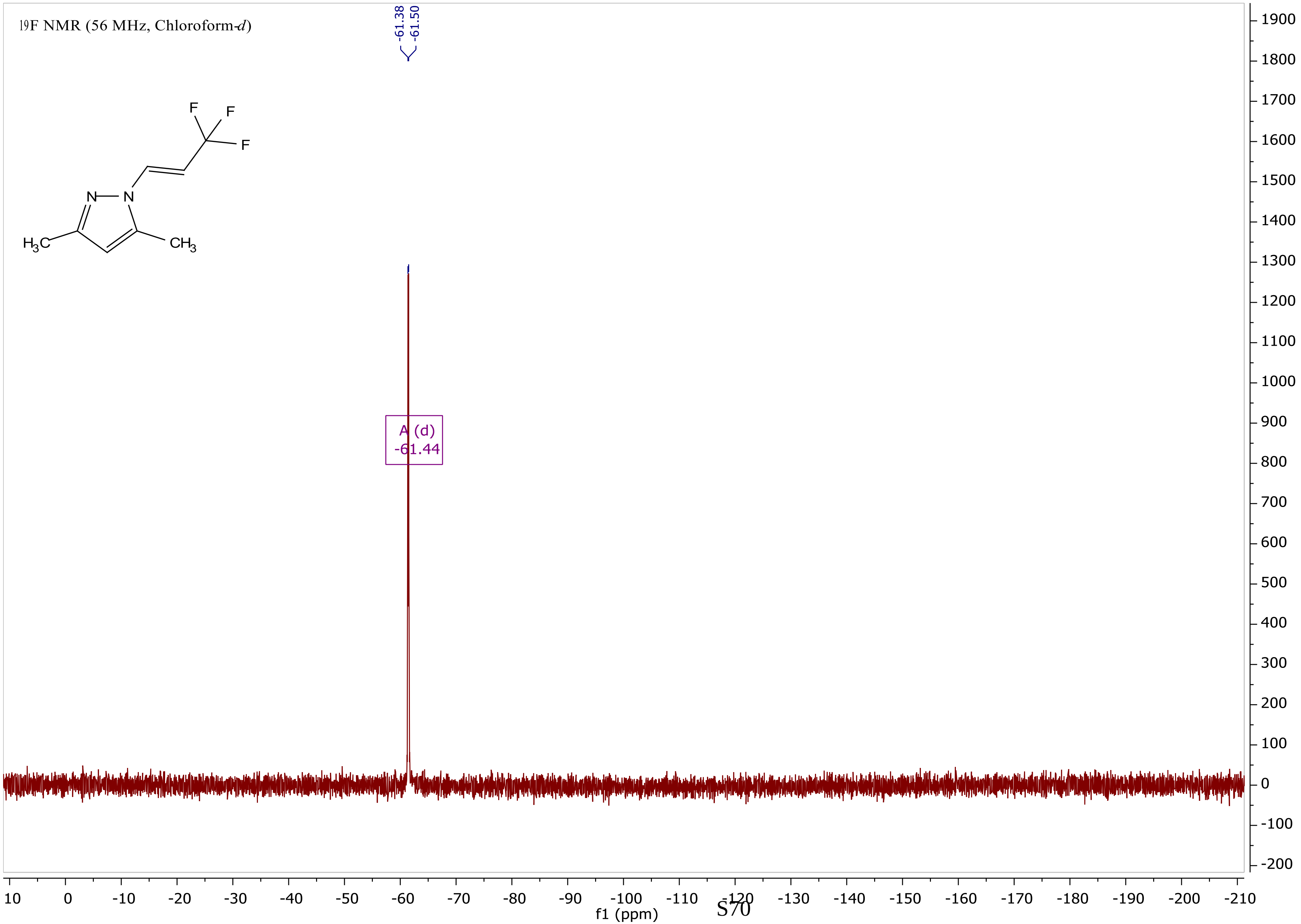

19F NMR (56 MHz, Chloroform-d)
-61.38
-61.50
A (d)
-61.44
F
F
F
N
N
H3C
CH3
f1 (ppm)

(*E*)-4-Iodo-3,5-dimethyl-1-(3,3,3-trifluoroprop-1-en-1-yl)-1*H*-pyrazole (6)

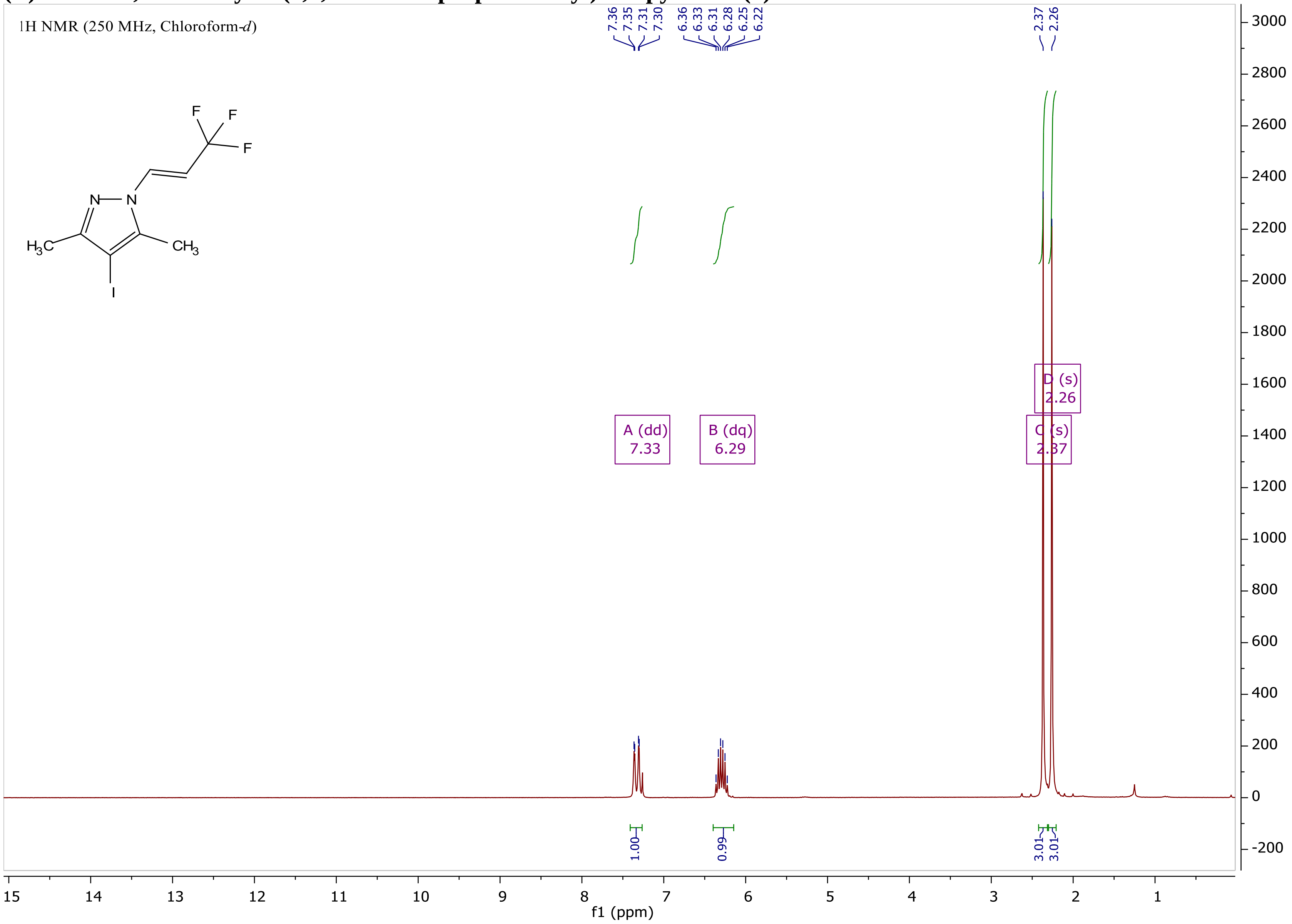

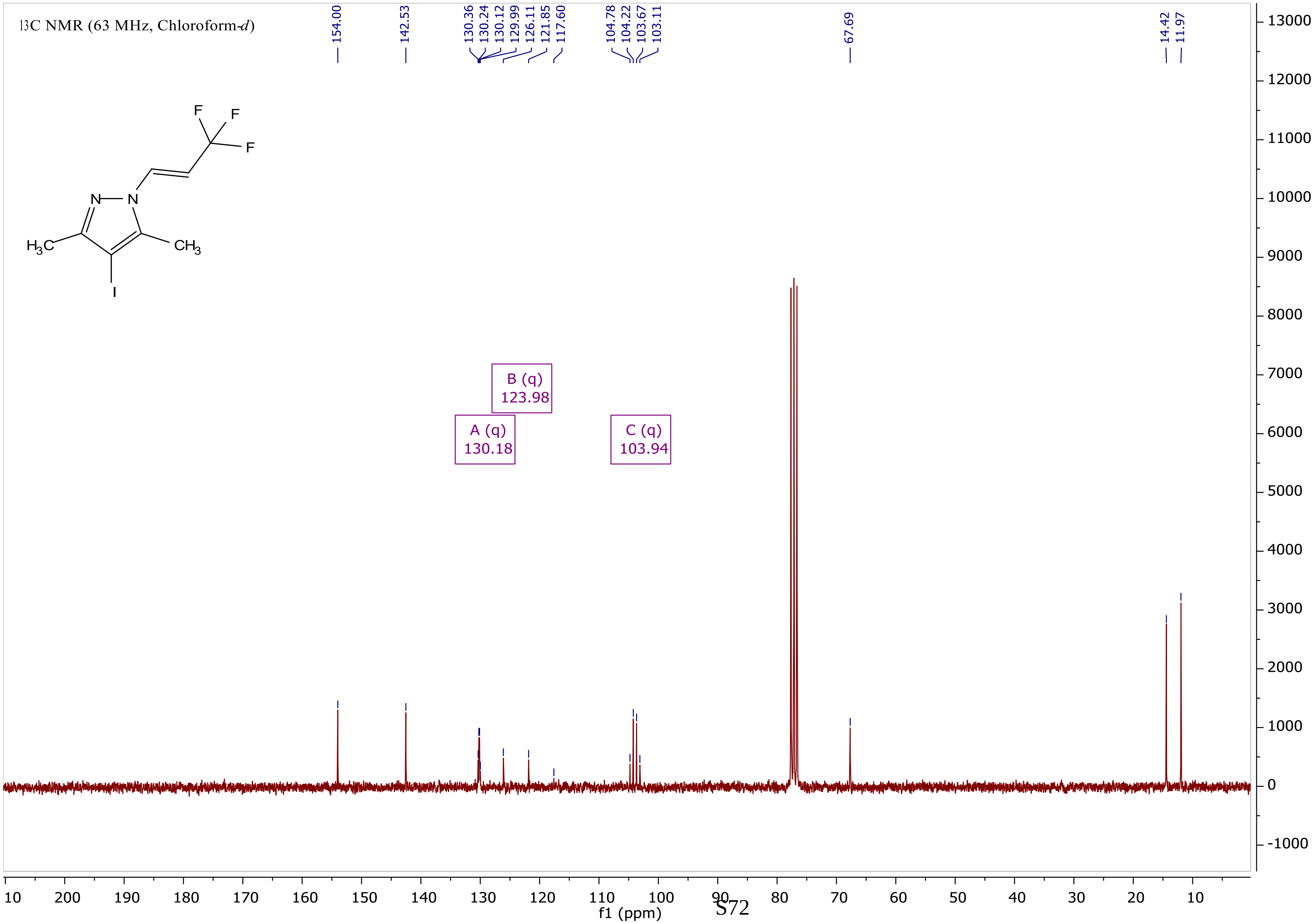
13C NMR (63 MHz, Chloroform-d)
154.00
142.53
130.36
130.24
130.12
129.99
126.11
121.85
117.60
104.78
104.22
103.67
103.11
67.69
14.42
11.97
F
F
F
N
N
H3C
CH3
I
B (q)
123.98
A (q)
130.18
C (q)
103.94
f1 (ppm)

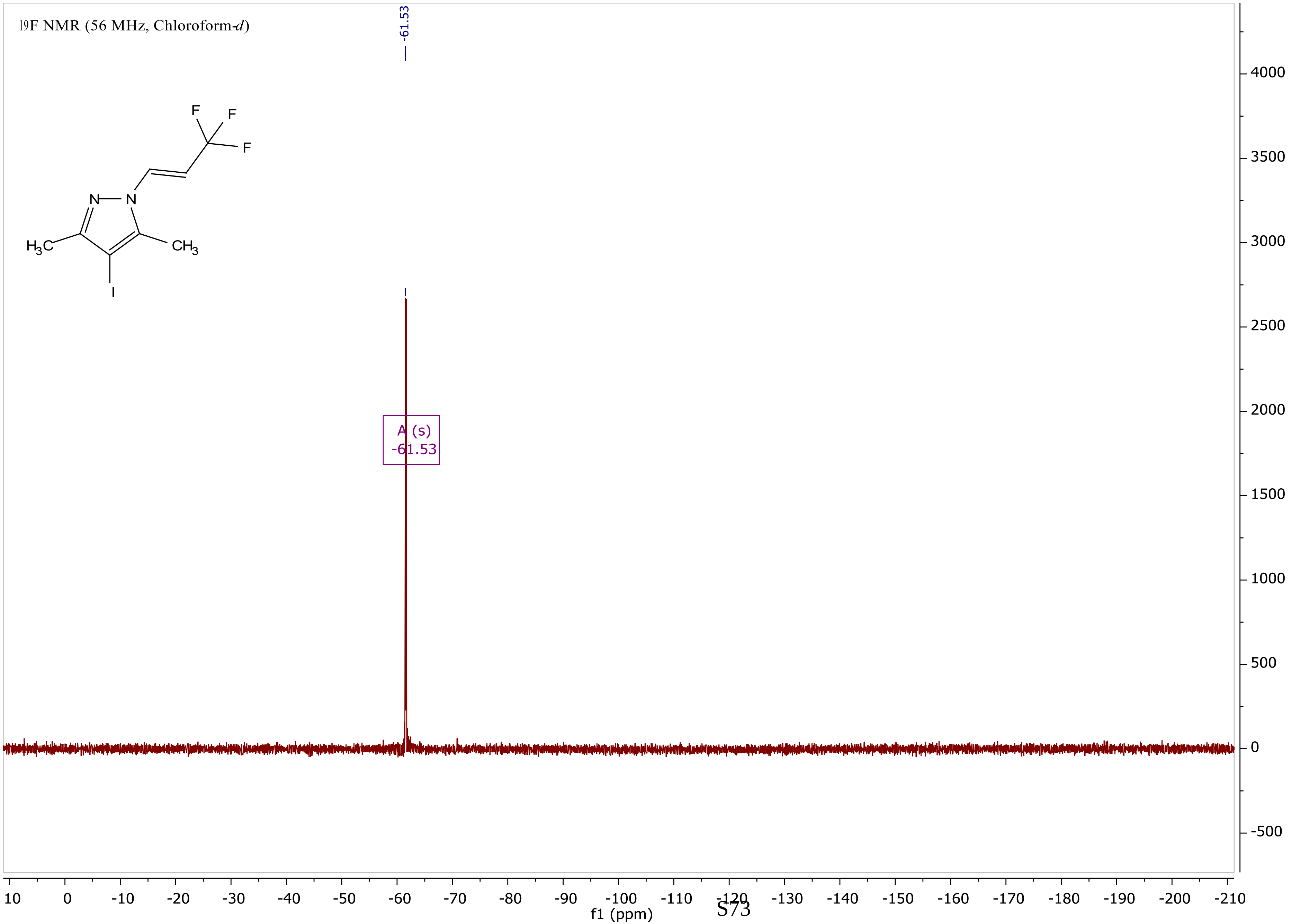
19F NMR (56 MHz, Chloroform-d)
-61.53
A (s)
-61.53
F
F
F
N
N
H3C
CH3
I
f1 (ppm)

(*E*)-3,5-Diphenyl-1-(3,3,3-trifluoroprop-1-en-1-yl)-1*H*-pyrazole (7)

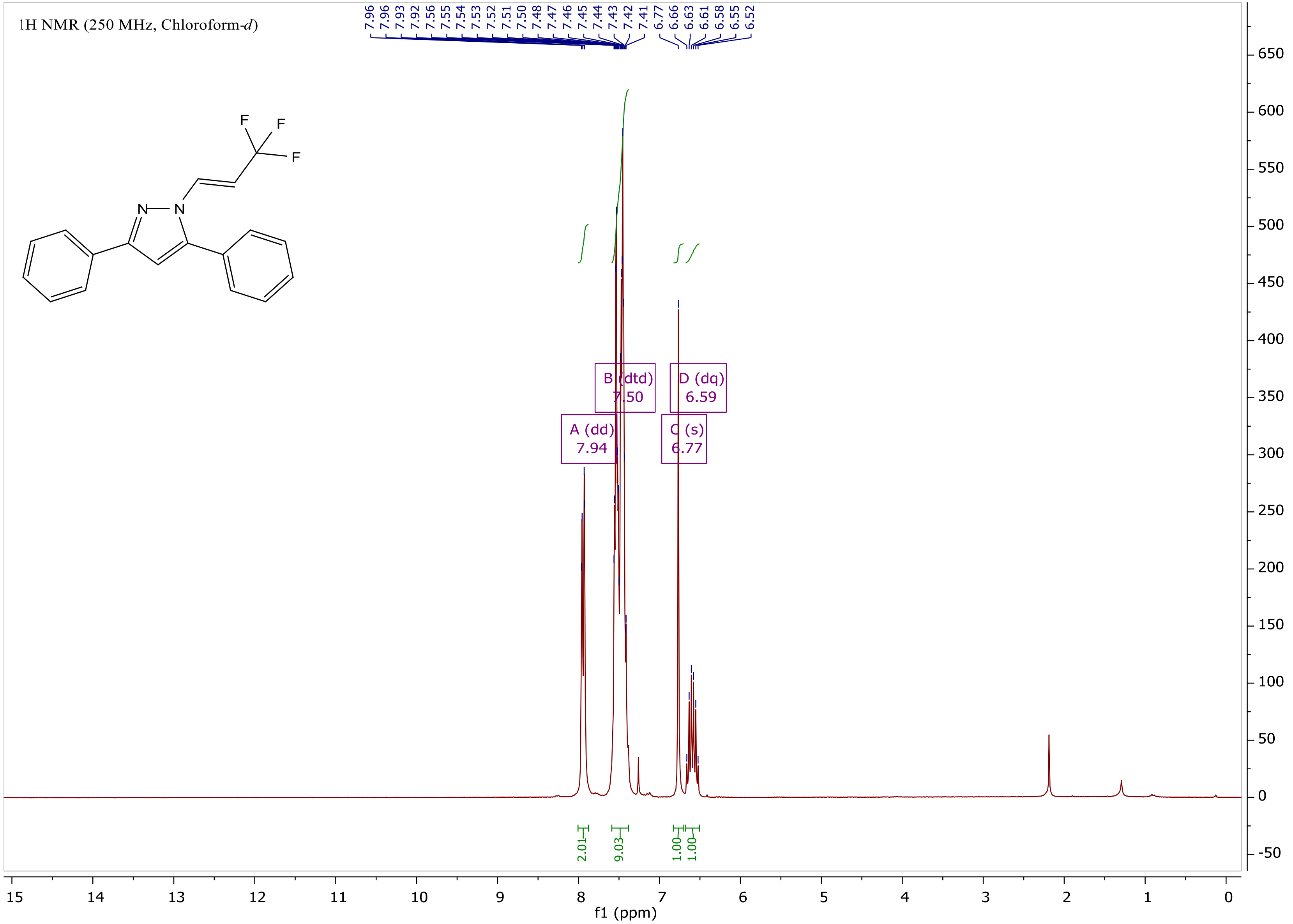

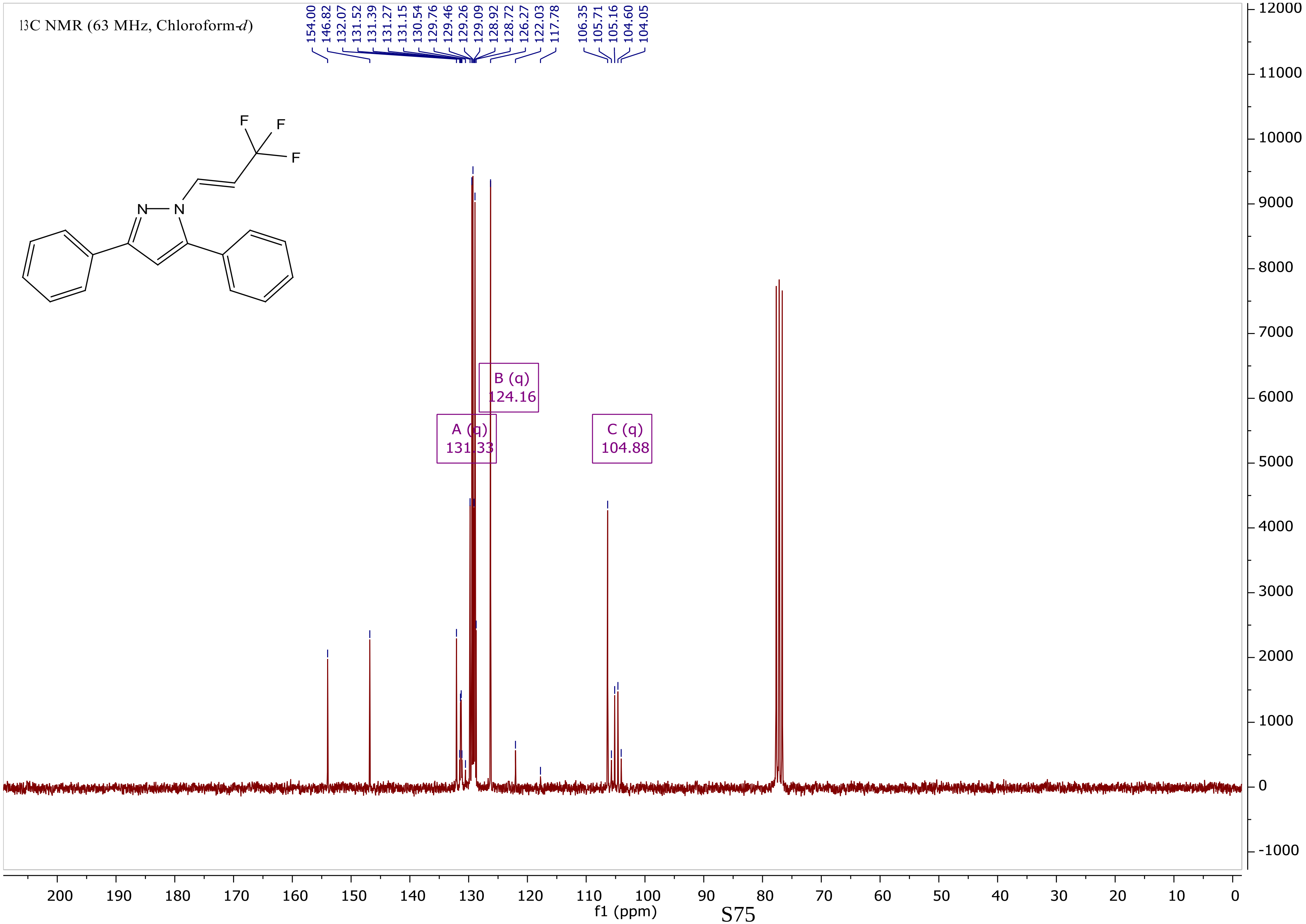
13C NMR (63 MHz, Chloroform-d)
154.00
146.82
132.07
131.52
131.39
131.27
131.15
130.54
129.76
129.46
129.26
129.09
128.92
128.72
126.27
122.03
117.78
106.35
105.71
105.16
104.60
104.05
F
F
F
N
N
B (q)
124.16
A (q)
131.33
C (q)
104.88
f1 (ppm)

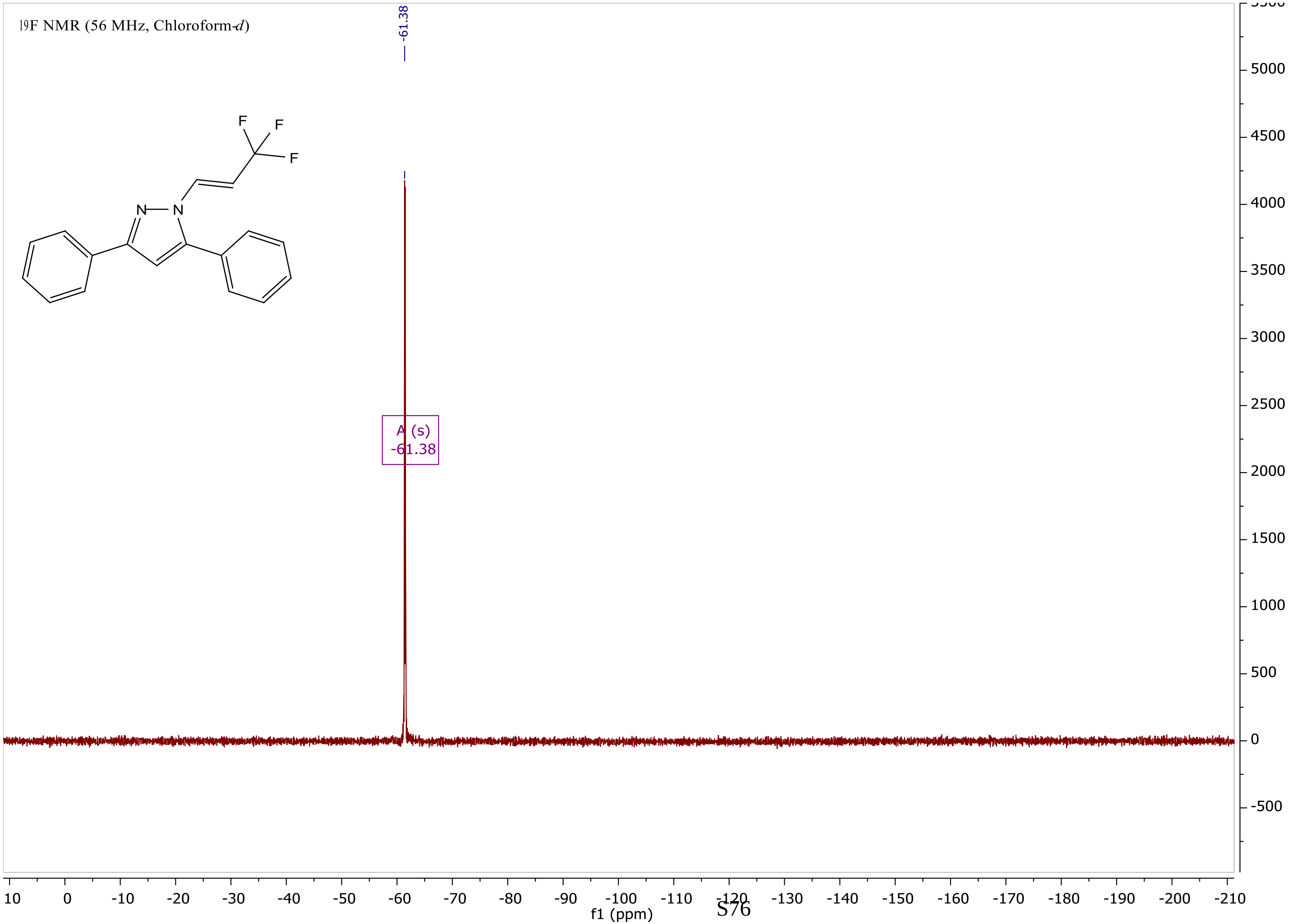

19F NMR (56 MHz, Chloroform-d)
-61.38
A (s)
-61.38
F
F
F
N
N
f1 (ppm)

(*E*)-3,5-Di-4-tolyl-1-(3,3,3-trifluoroprop-1-en-1-yl)-1*H*-pyrazole (8)

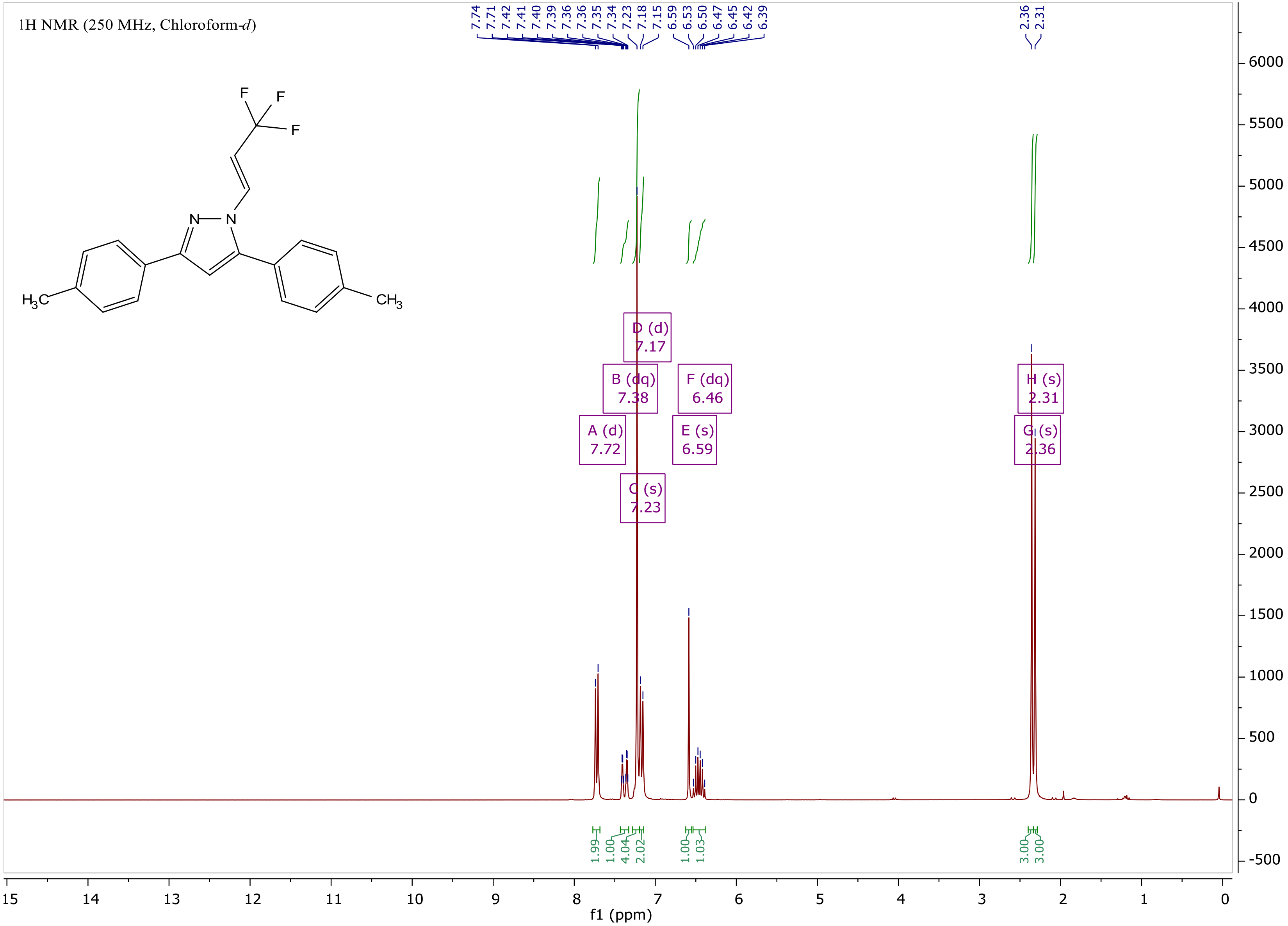

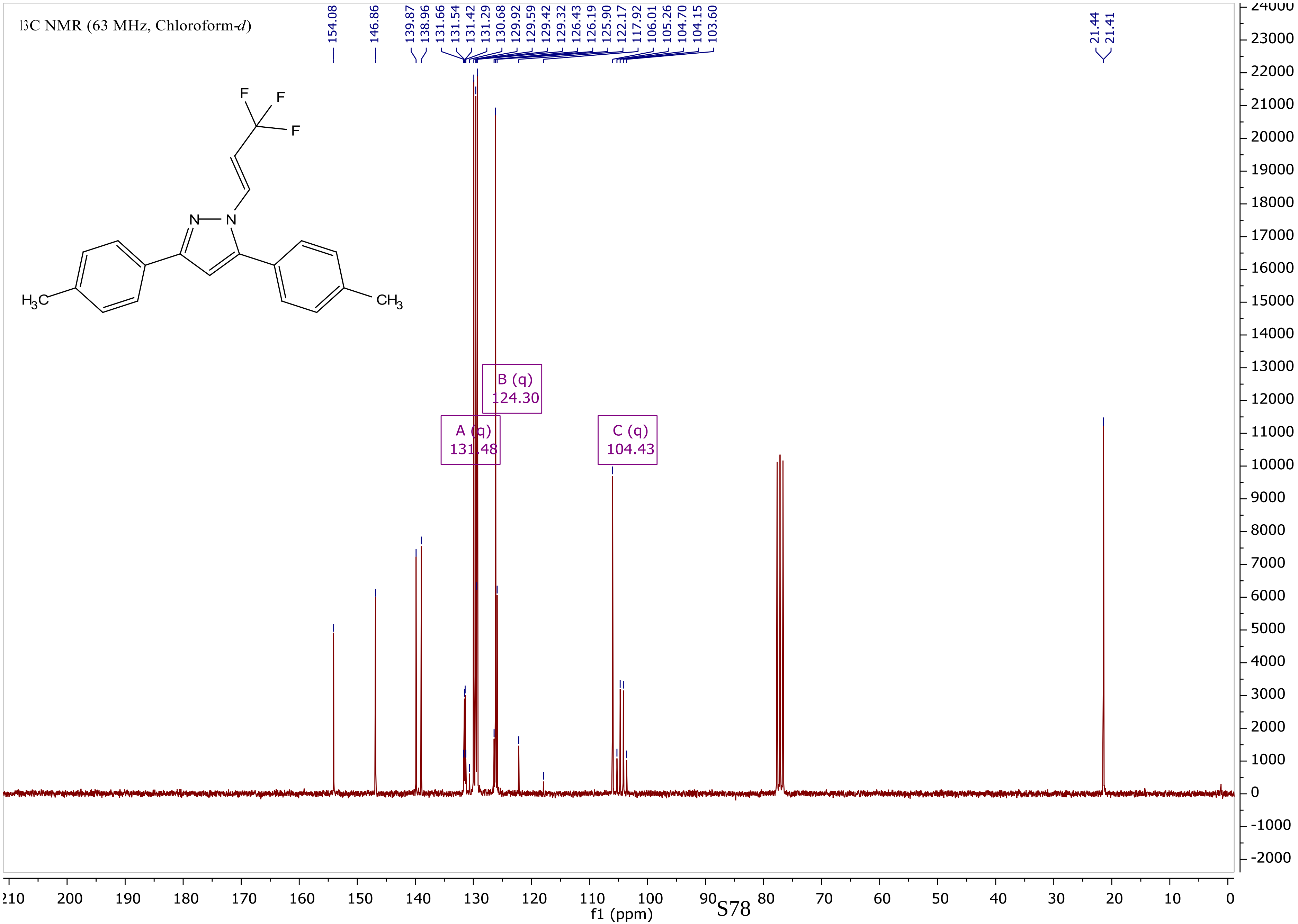

13C NMR (63 MHz, Chloroform-d)
154.08
146.86
139.87
138.96
131.66
131.54
131.42
131.29
130.68
129.92
129.59
129.42
129.32
126.43
126.19
125.90
122.17
117.92
106.01
105.26
104.70
104.15
103.60
21.44
21.41
A (q) 131.48
B (q) 124.30
C (q) 104.43
f1 (ppm)

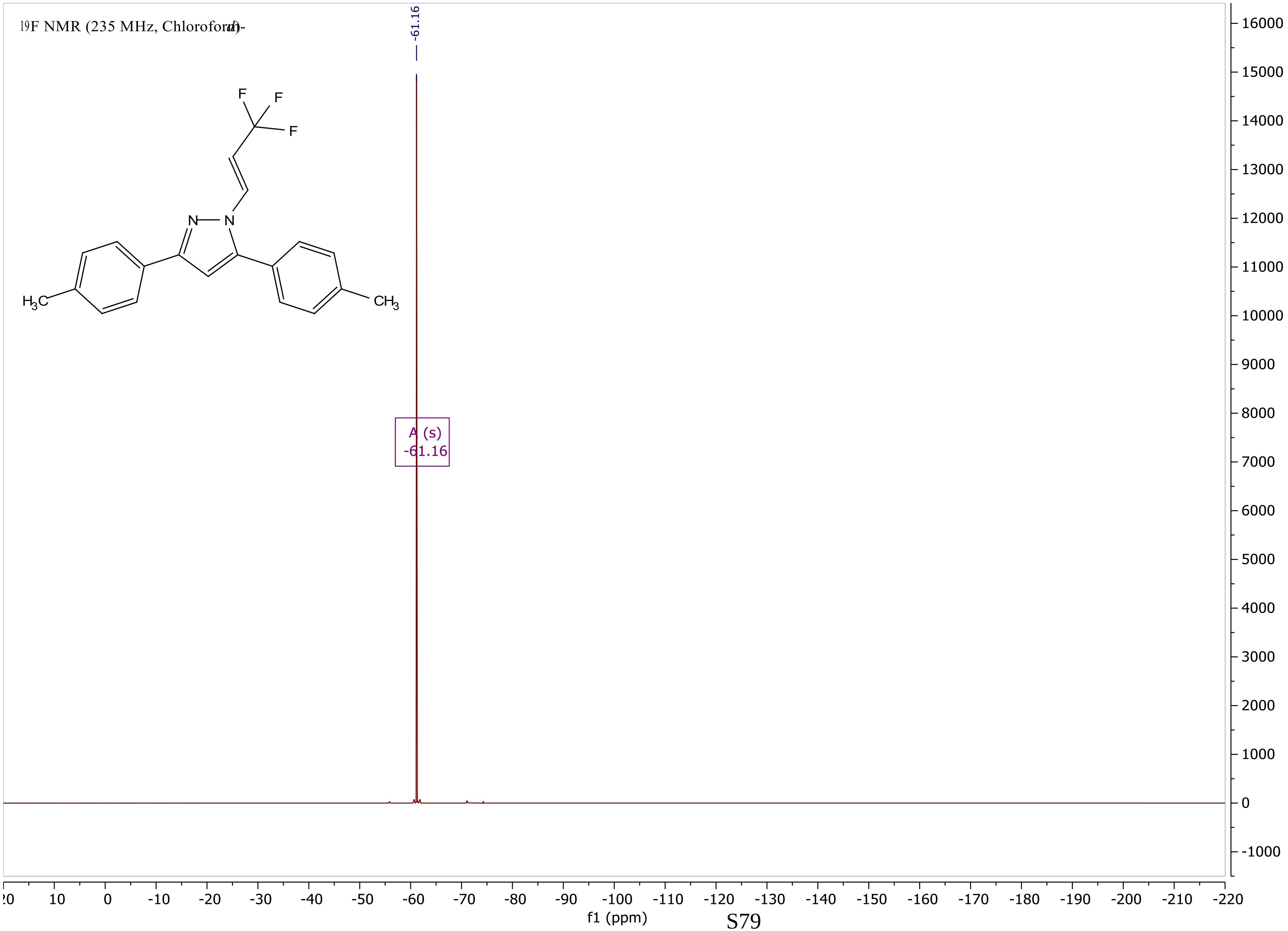
19F NMR (235 MHz, Chloroform-d)
-61.16
A (s)
-61.16
F
F
F
N
N
H3C
CH3
f1 (ppm)

(*E*)-3,5-Bis(4-chlorophenyl)-1-(3,3,3-trifluoroprop-1-en-1-yl)-1*H*-pyrazole (9)

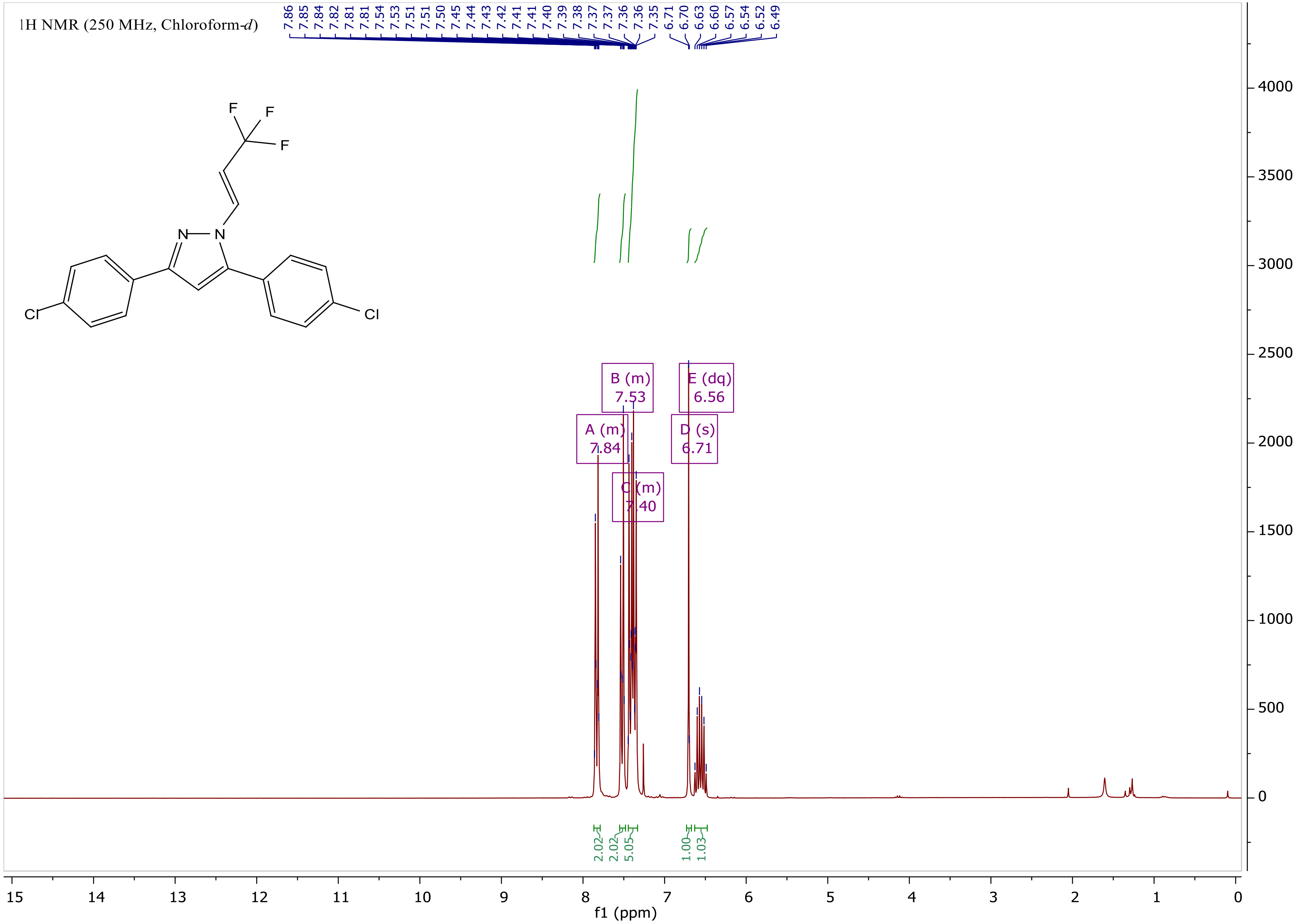

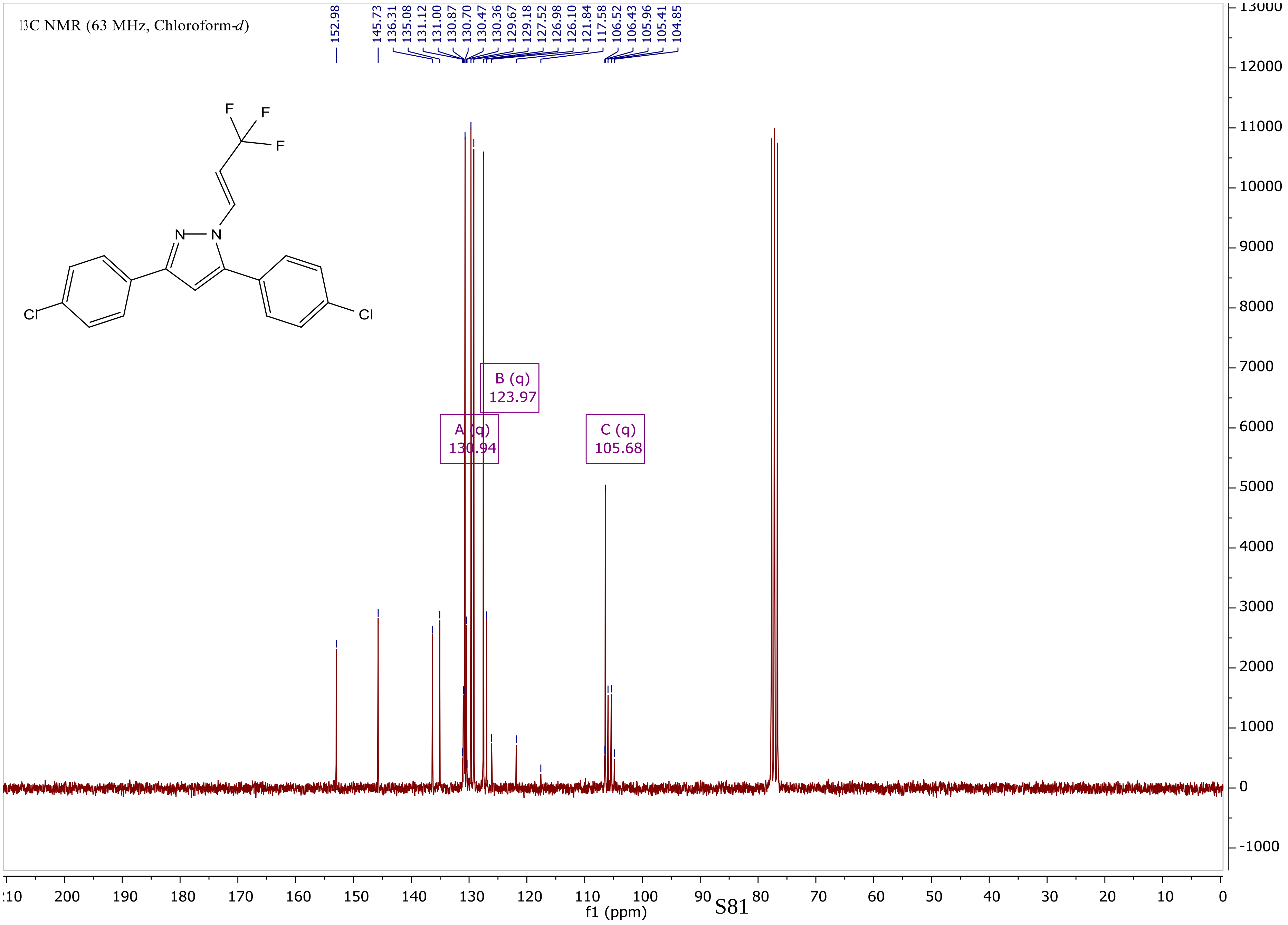
13C NMR (63 MHz, Chloroform-d)
152.98
145.73
136.31
135.08
131.12
131.00
130.87
130.70
130.47
130.36
129.67
129.18
127.52
126.98
126.10
121.84
117.58
106.52
106.43
105.96
105.41
104.85
F
F
F
N
N
Cl
Cl
B (q)
123.97
A (q)
130.94
C (q)
105.68
f1 (ppm)

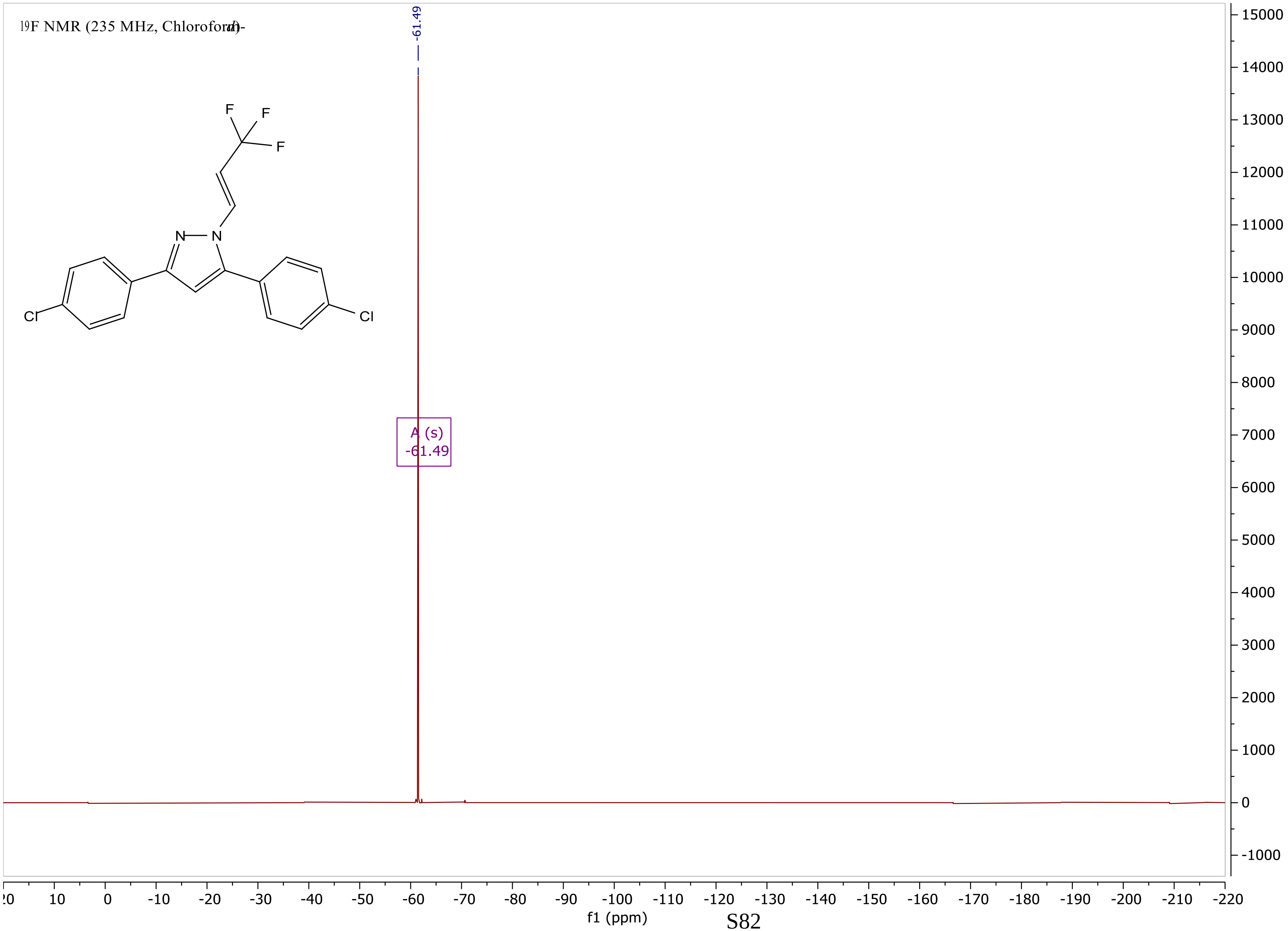

19F NMR (235 MHz, Chloroform-d)
-61.49
A (s)
-61.49
F
F
F
N
N
Cl
Cl
f1 (ppm)

(*E*)-3,5-Bis(4-bromophenyl)-1-(3,3,3-trifluoroprop-1-en-1-yl)-1*H*-pyrazole (10)

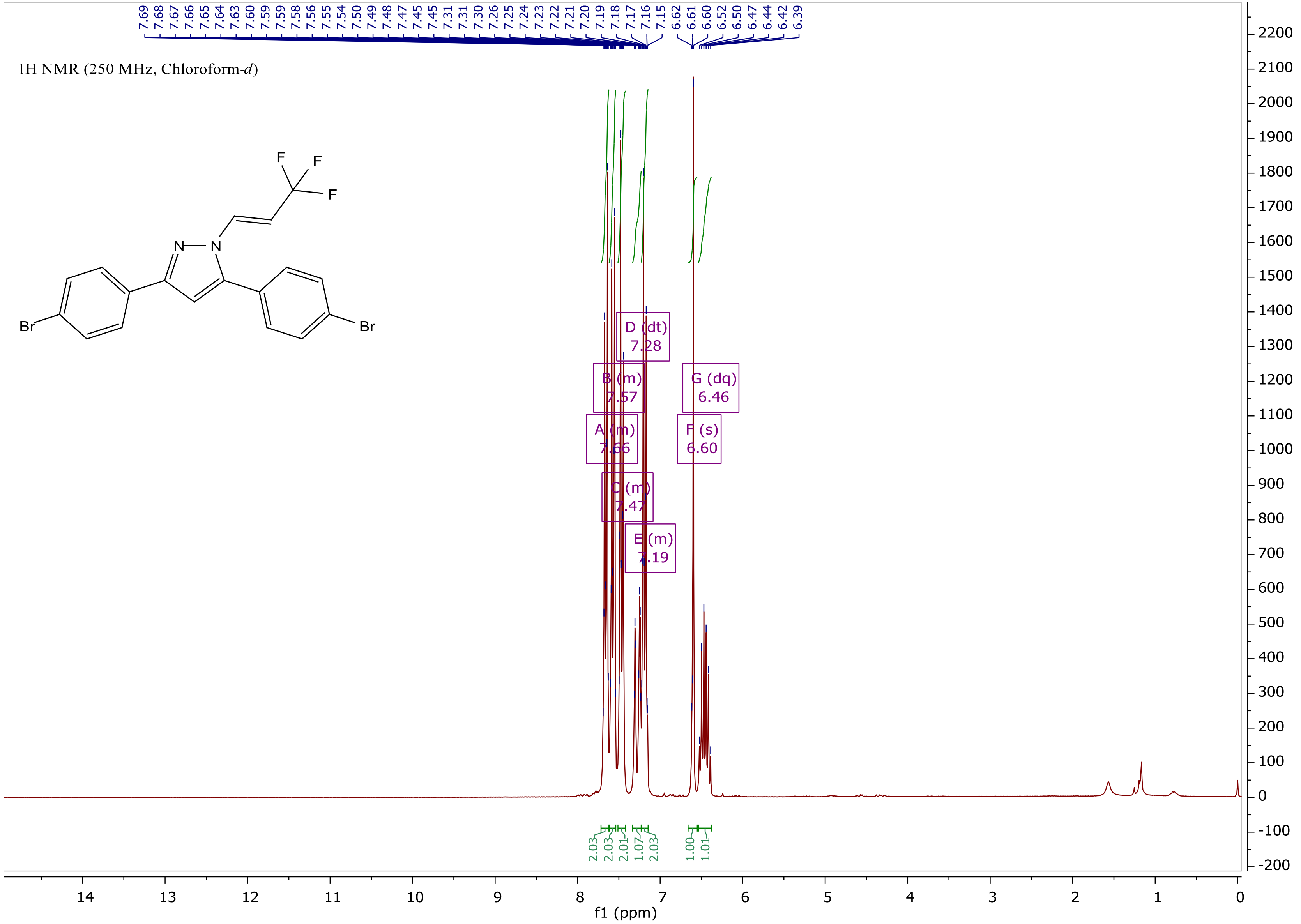

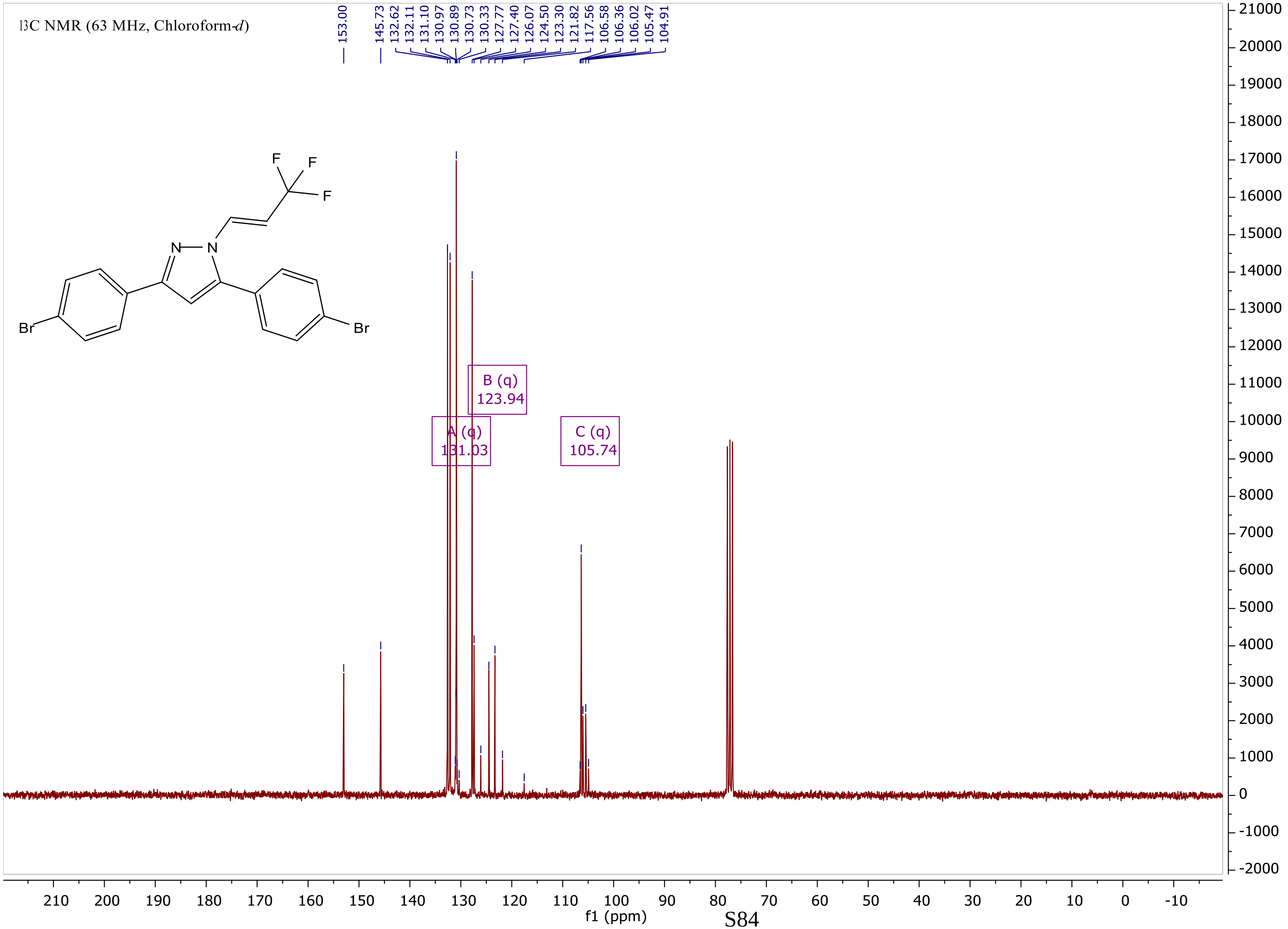
13C NMR (63 MHz, Chloroform-d)
153.00
145.73
132.62
132.11
131.10
130.97
130.89
130.73
130.33
127.77
127.40
126.07
124.50
123.30
121.82
117.56
106.58
106.36
106.02
105.47
104.91
F
F
F
N
N
Br
Br
A (q)
131.03
B (q)
123.94
C (q)
105.74
f1 (ppm)

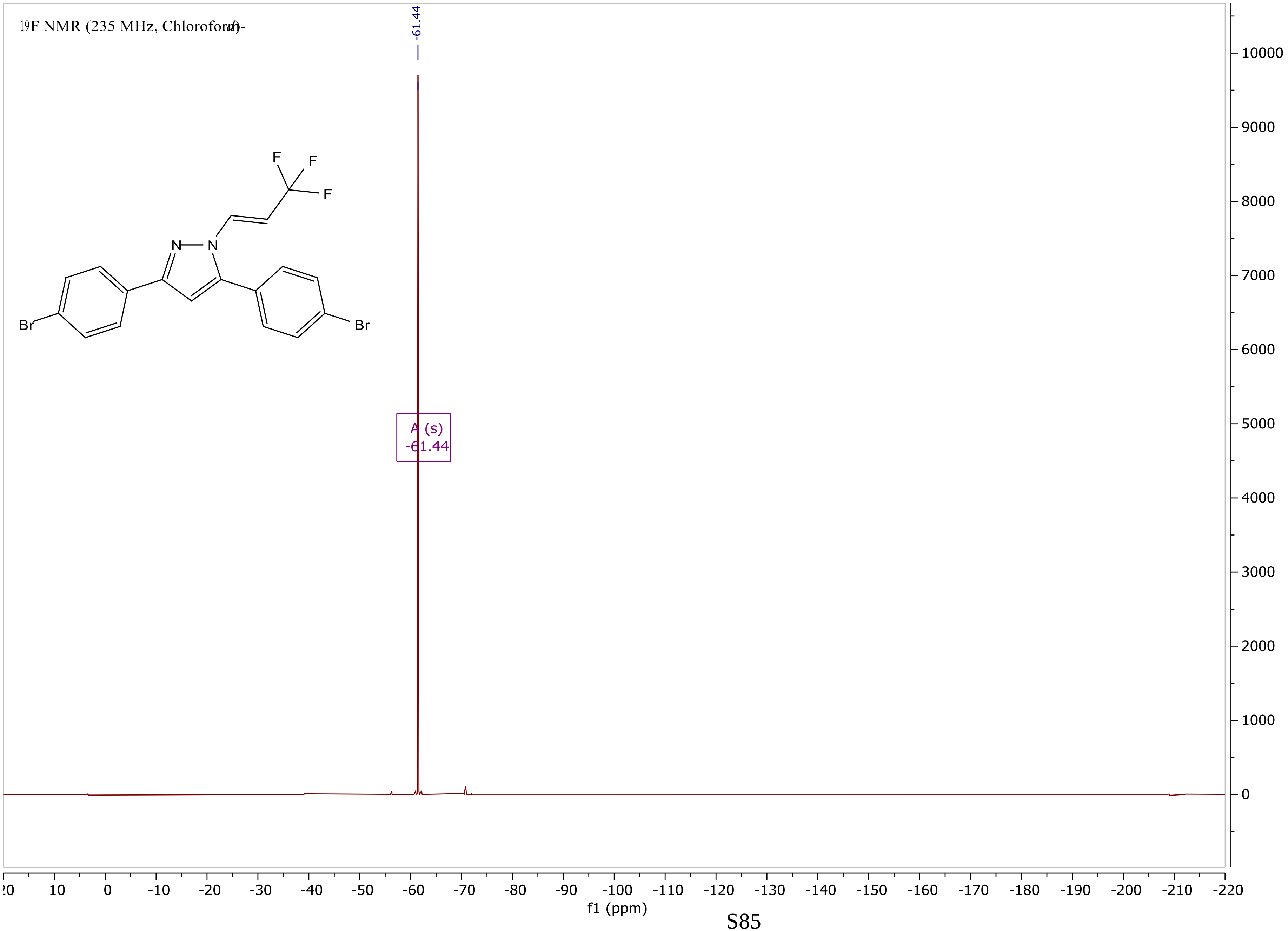
19F NMR (235 MHz, Chloroform-d)
-61.44
A (s)
-61.44
f1 (ppm)

(*E*)-3,5-Bis(2-bromophenyl)-1-(3,3,3-trifluoroprop-1-en-1-yl)-1*H*-pyrazole (11)

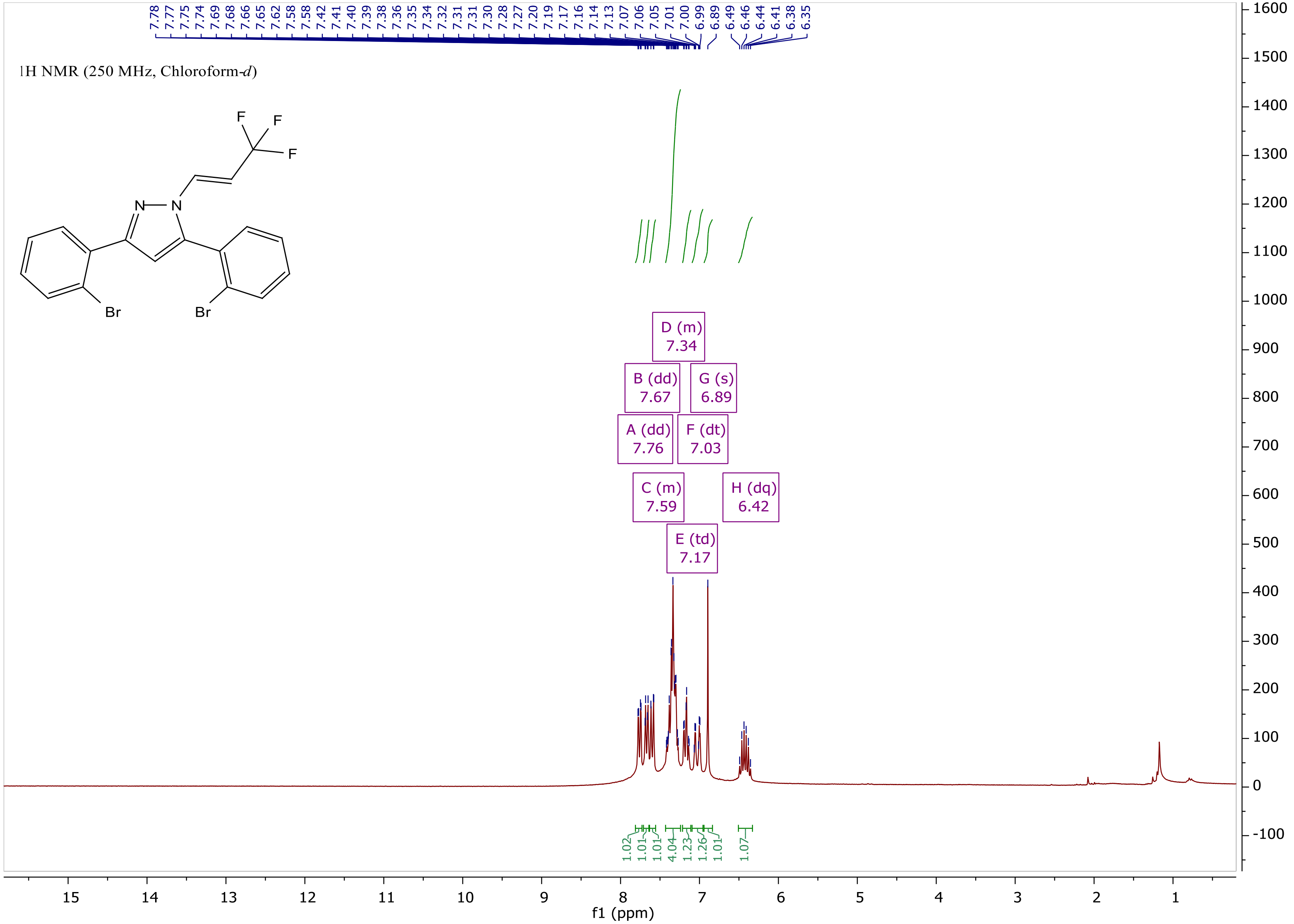

13C NMR (63 MHz, Chloroform-*d*)

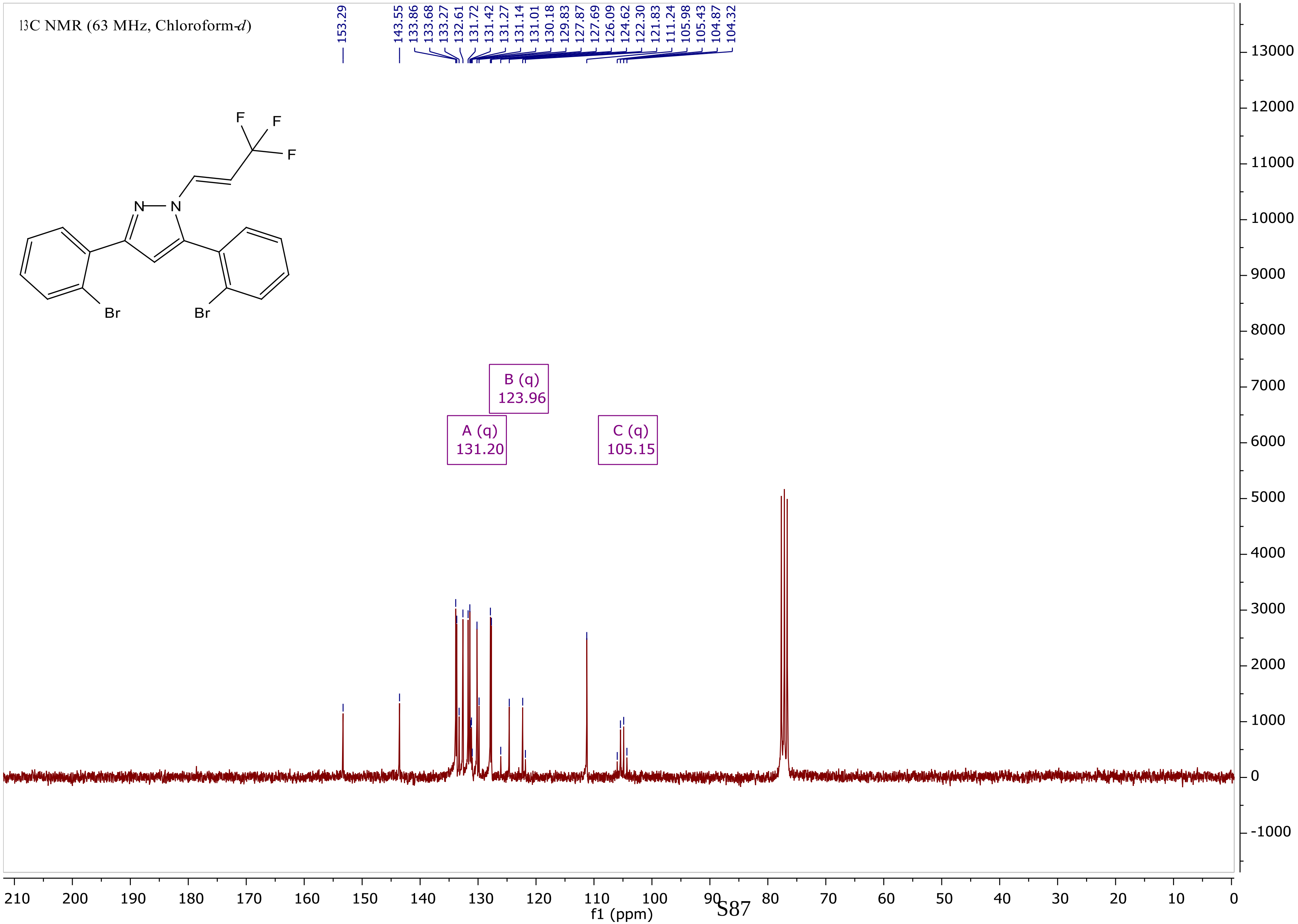

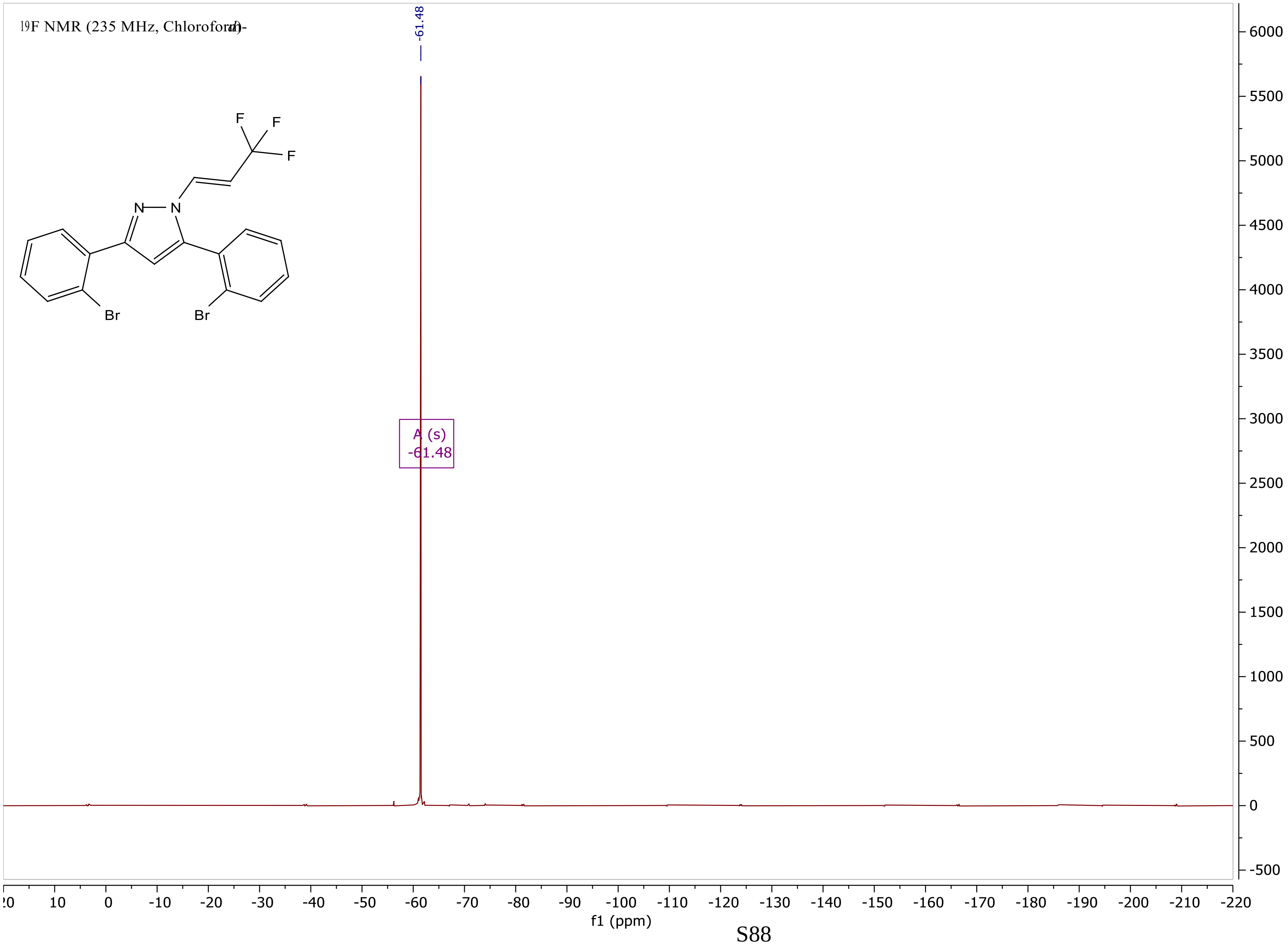
19F NMR (235 MHz, Chloroform-d)
-61.48
A (s)
-61.48
F
F
F
N
N
Br
Br
f1 (ppm)

(*E*)-3,5-Bis(4-methoxyphenyl)-1-(3,3,3-trifluoroprop-1-en-1-yl)-1*H*-pyrazole (12)

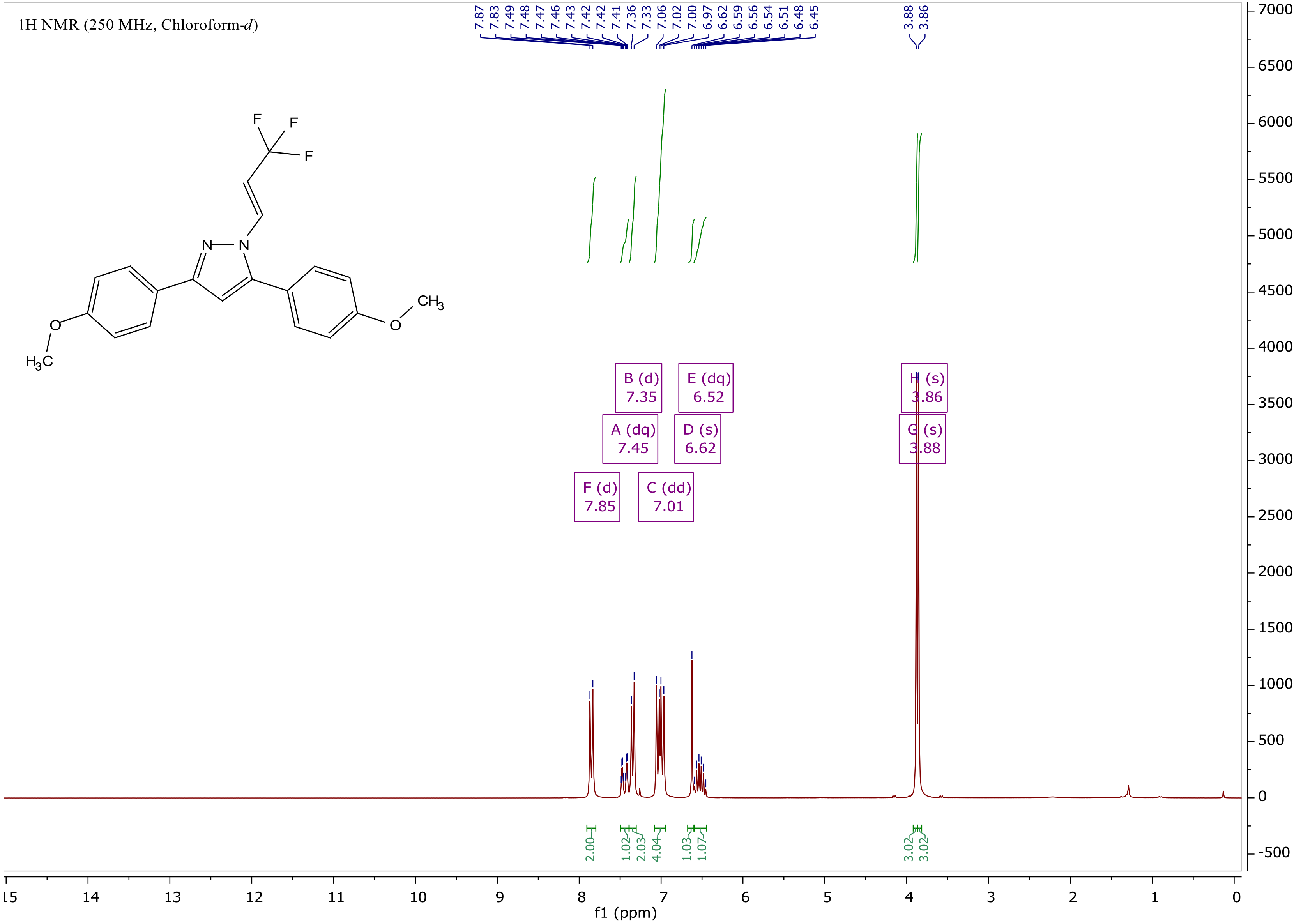

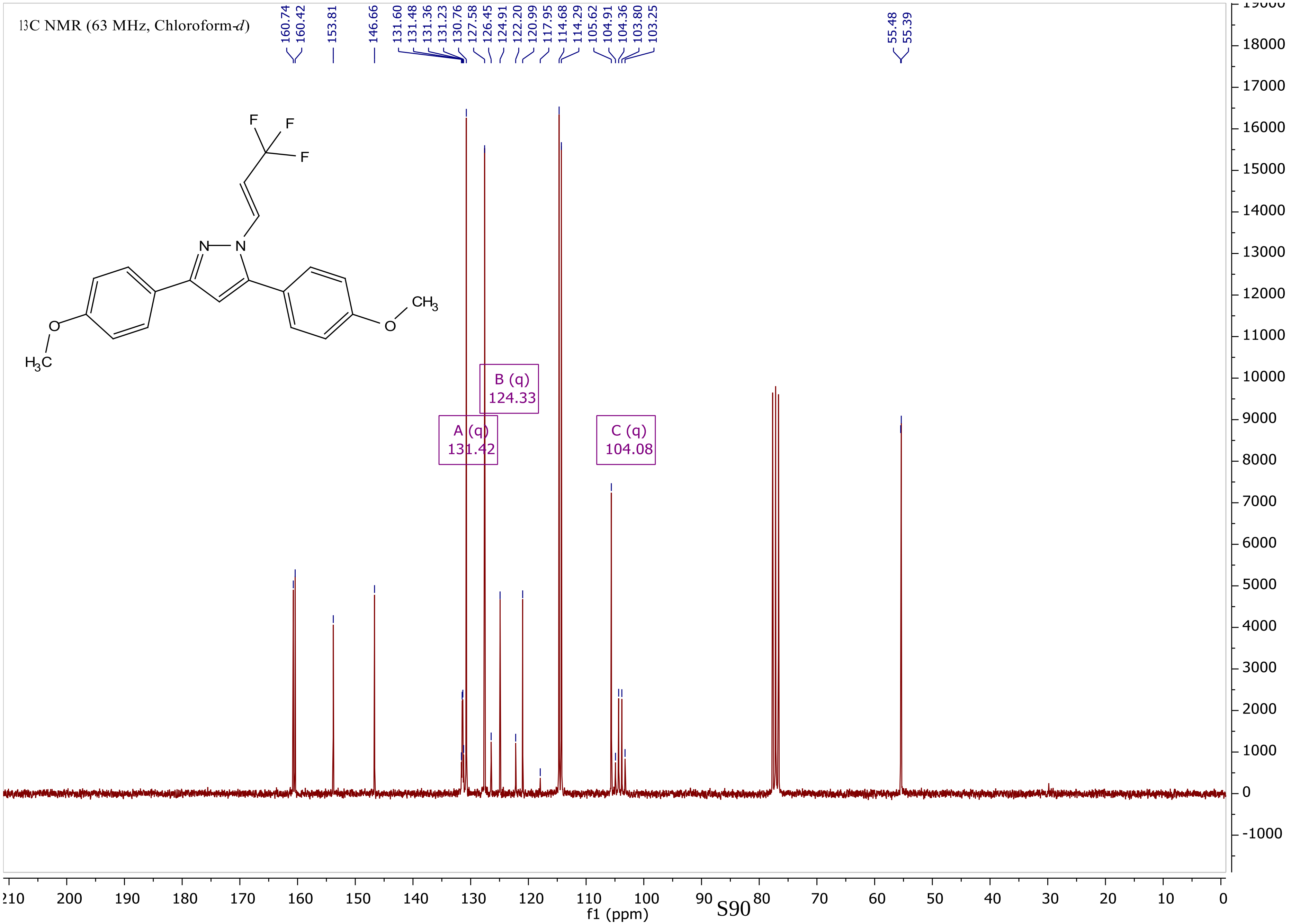

13C NMR (63 MHz, Chloroform-d)
160.74
160.42
153.81
146.66
131.60
131.48
131.36
131.23
130.76
127.58
126.45
124.91
122.20
120.99
117.95
114.68
114.29
105.62
104.91
104.36
103.80
103.25
55.48
55.39
A (q) 131.42
B (q) 124.33
C (q) 104.08
f1 (ppm)

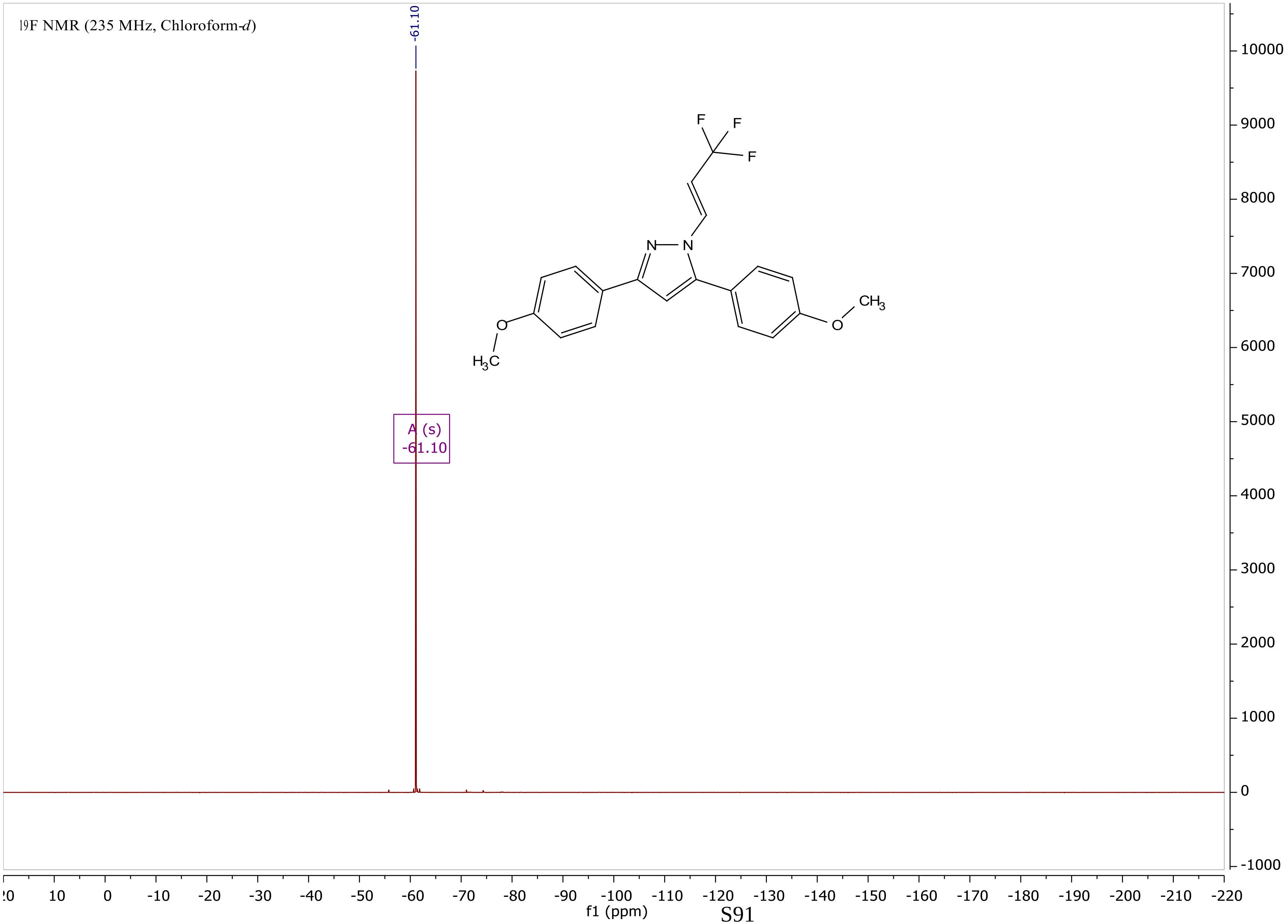
19F NMR (235 MHz, Chloroform-d)
-61.10
A (s)
-61.10
F
F
F
N
N
CH3
O
H3C
O
f1 (ppm)

(*E*)-3,5-Bis(4-nitrophenyl)-1-(3,3,3-trifluoroprop-1-en-1-yl)-1*H*-pyrazole (13)

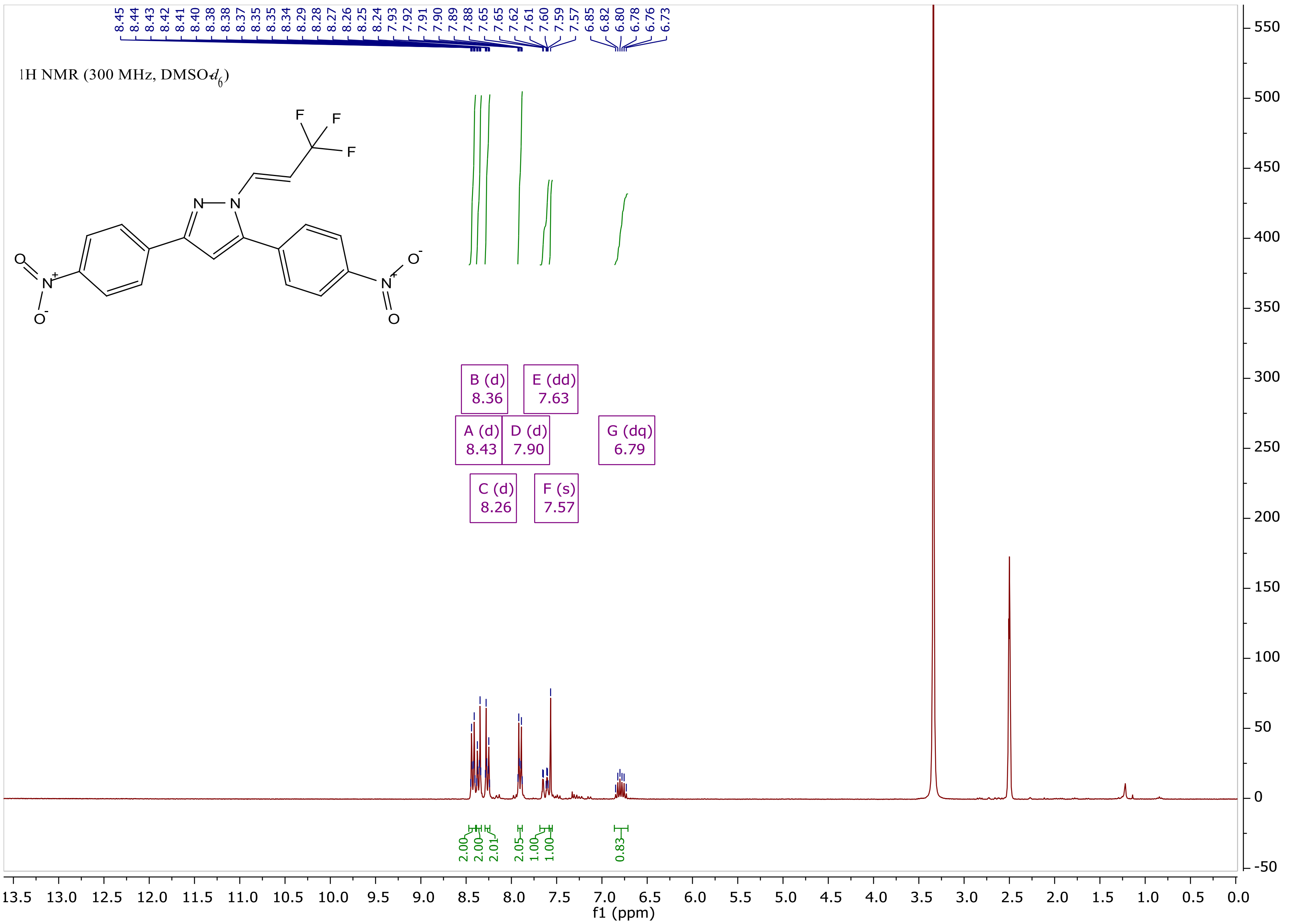

13C NMR (75 MHz, Chloroform-*d*)

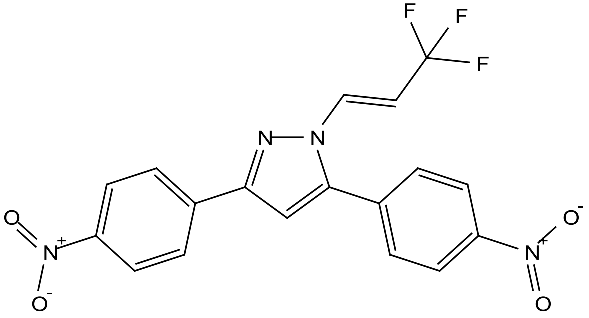

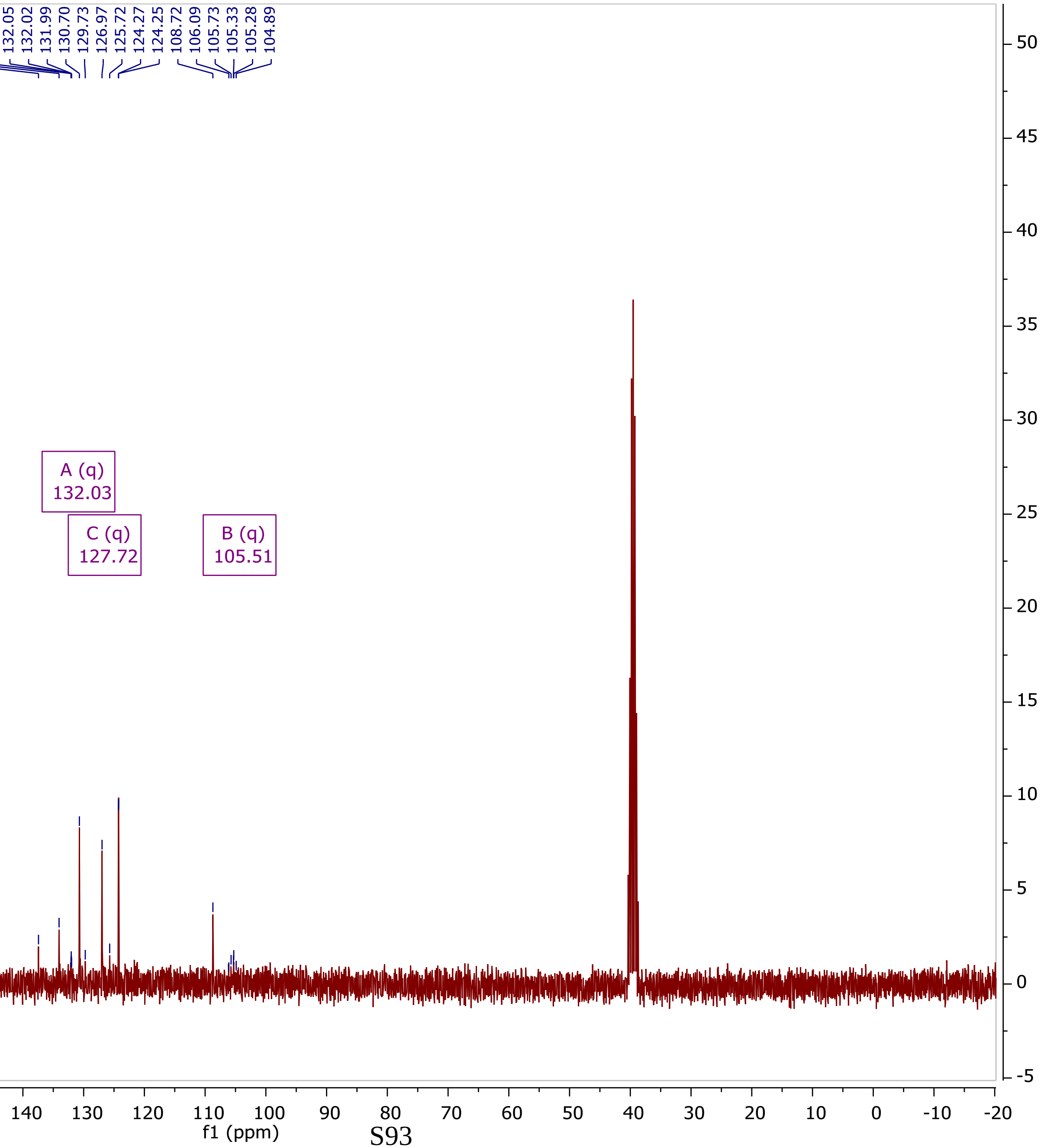

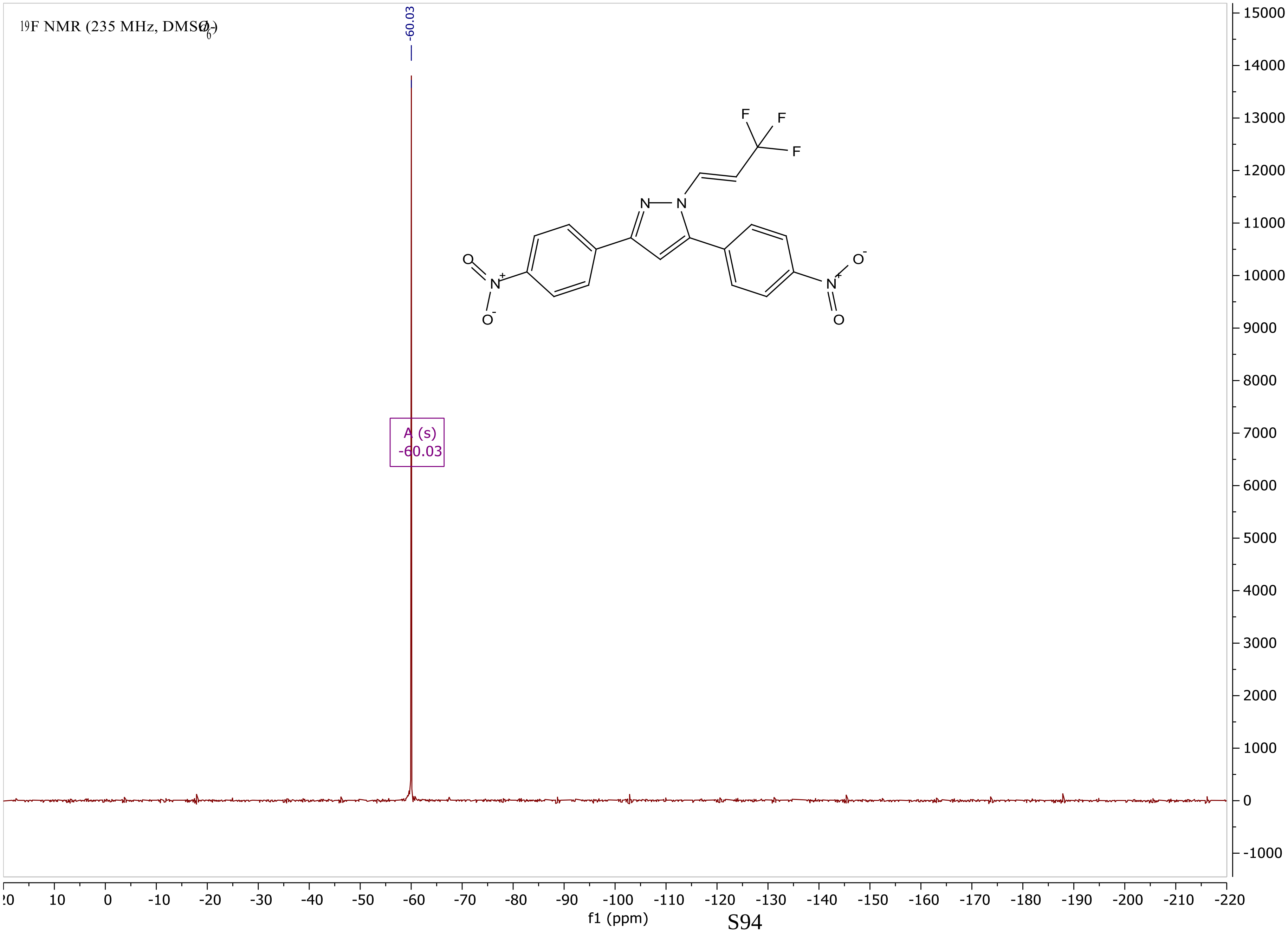

19F NMR (235 MHz, DMSO-d6)
-60.03
A (s)
-60.03
F
F
F
N
N
O
N+
O-
O-
N+
O
f1 (ppm)

(*E*)-3,5-(E)-4-Iodo-3,5-diphenyl-1-(3,3,3-trifluoroprop-1-en-1-yl)-1*H*-pyrazole (14)

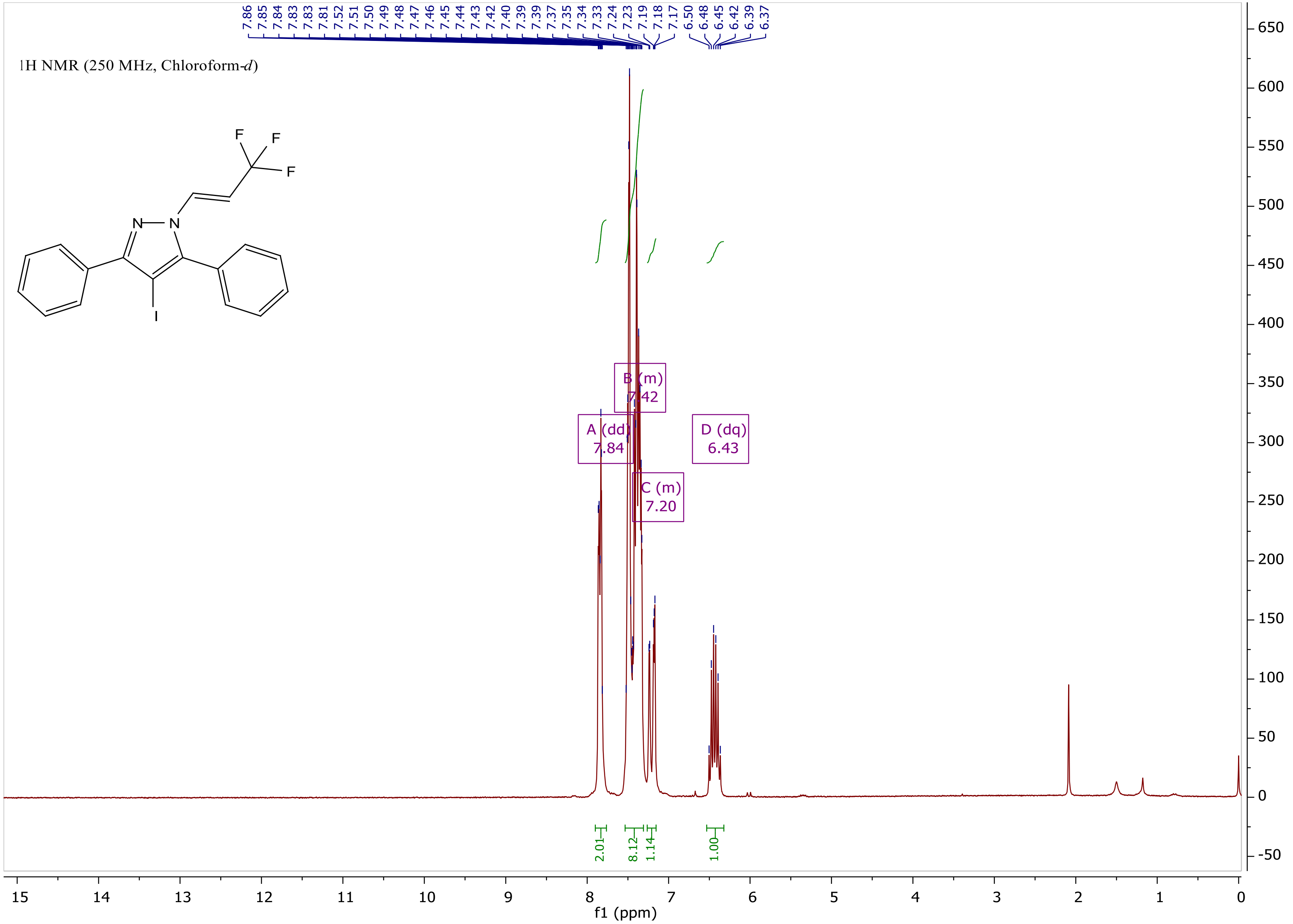

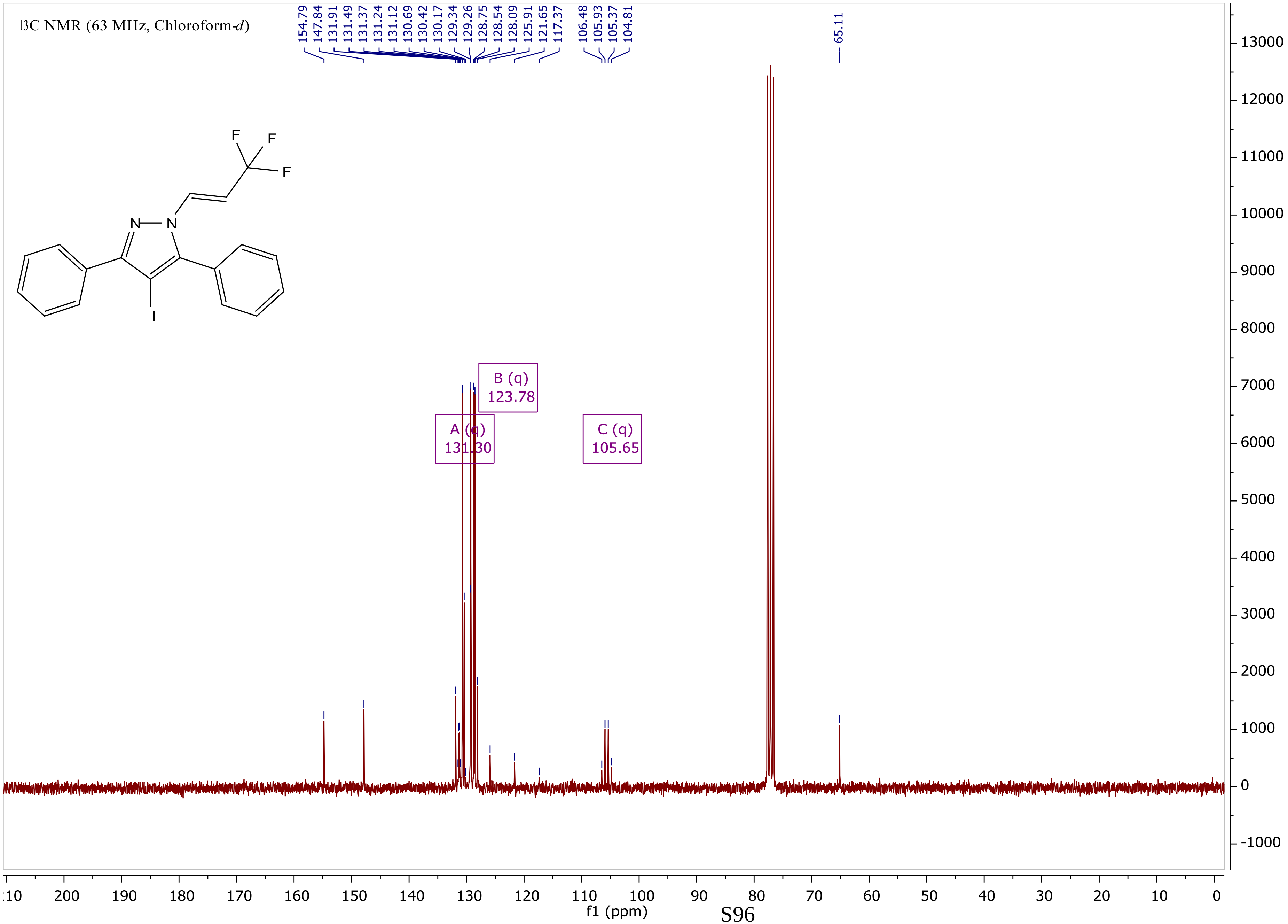
13C NMR (63 MHz, Chloroform-d)
154.79
147.84
131.91
131.49
131.37
131.24
131.12
130.69
130.42
130.17
129.34
129.26
128.75
128.54
128.09
125.91
121.65
117.37
106.48
105.93
105.37
104.81
65.11
F
F
F
N
N
I
A (q)
131.30
B (q)
123.78
C (q)
105.65
f1 (ppm)

19F NMR (56 MHz, Chloroform-*d*)

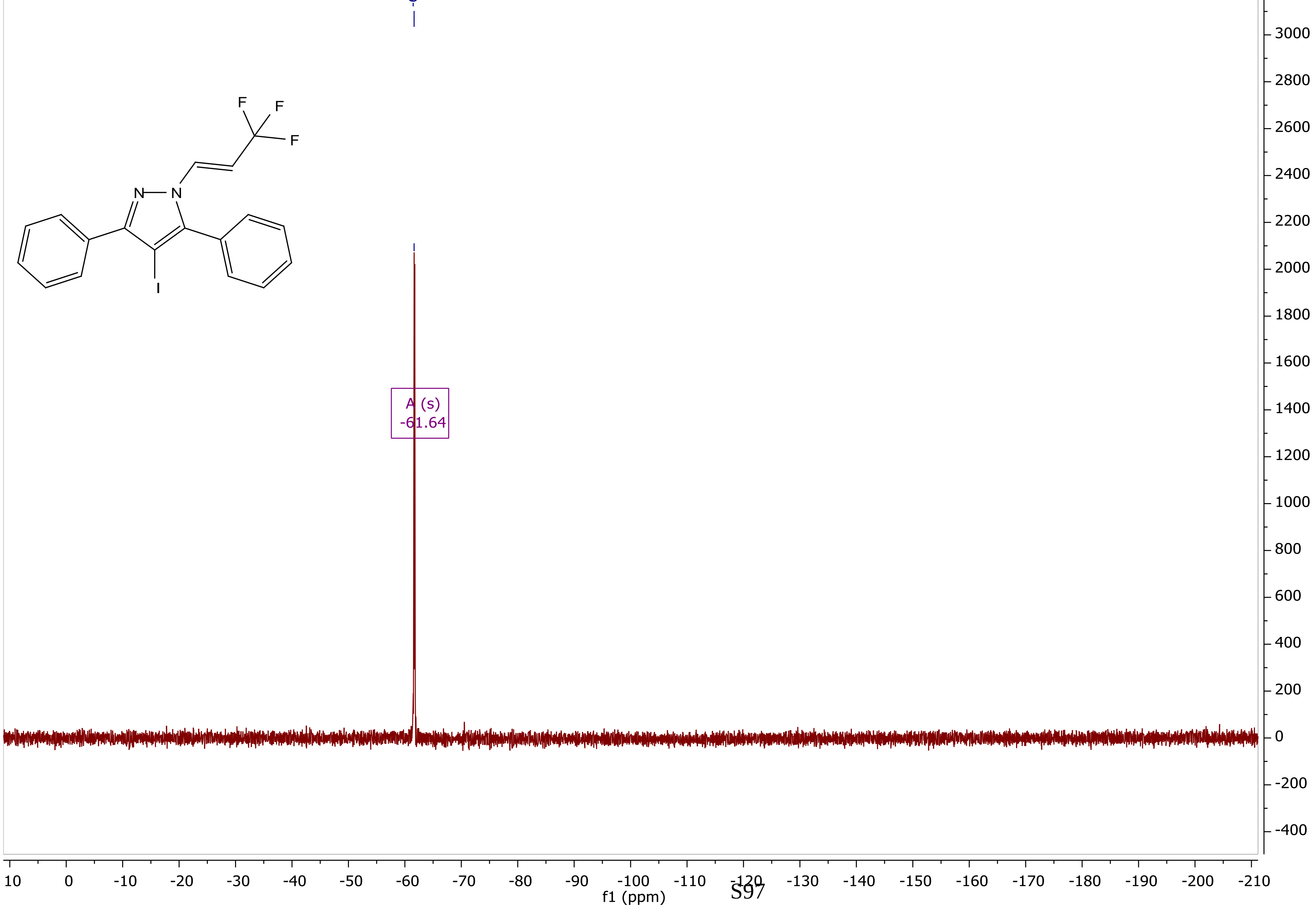

Ethyl (*E*)-3-(trifluoromethyl)-1-(3,3,3-trifluoroprop-1-en-1-yl)-1*H*-pyrazole-4-carboxylate (15)

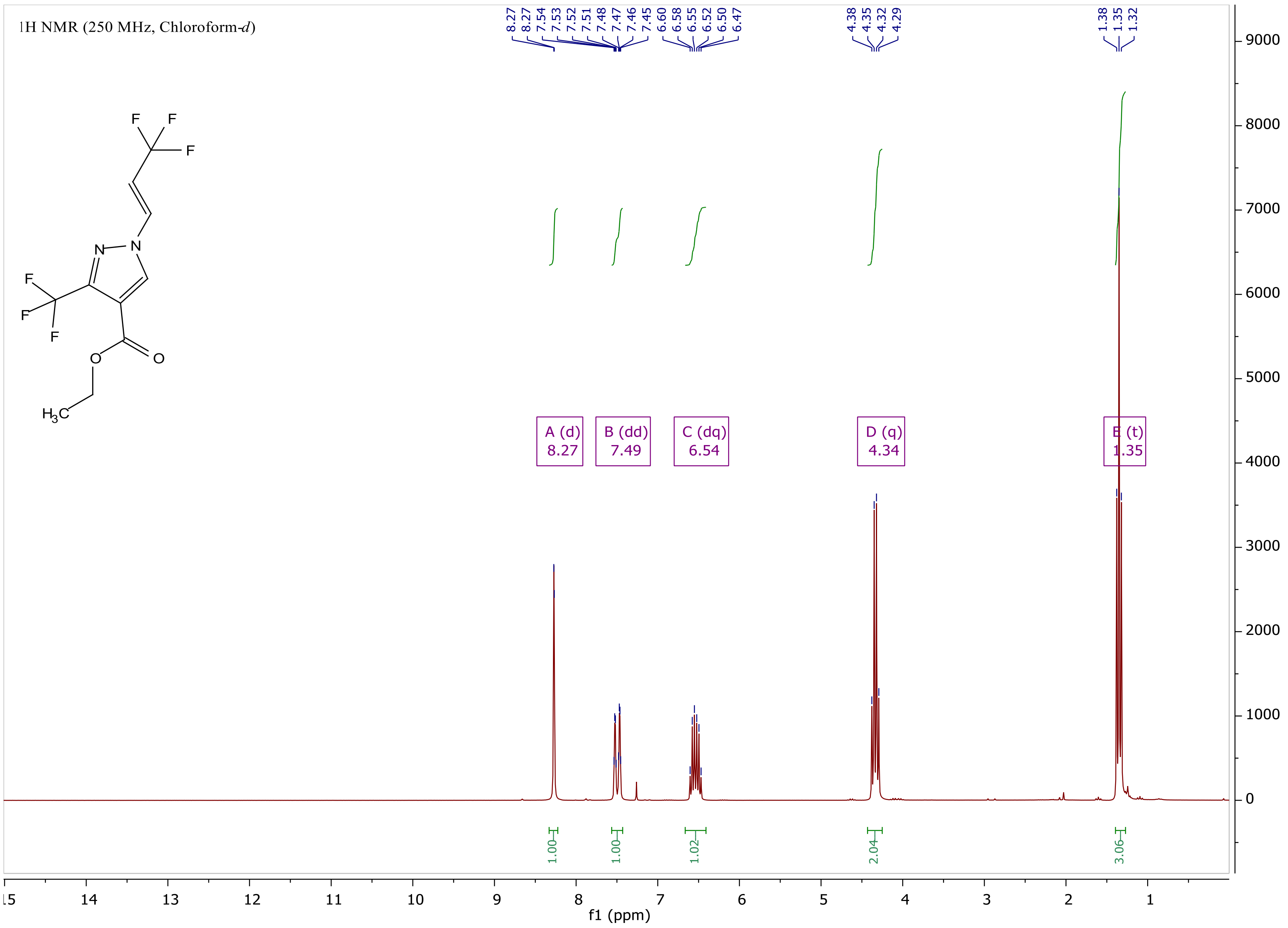

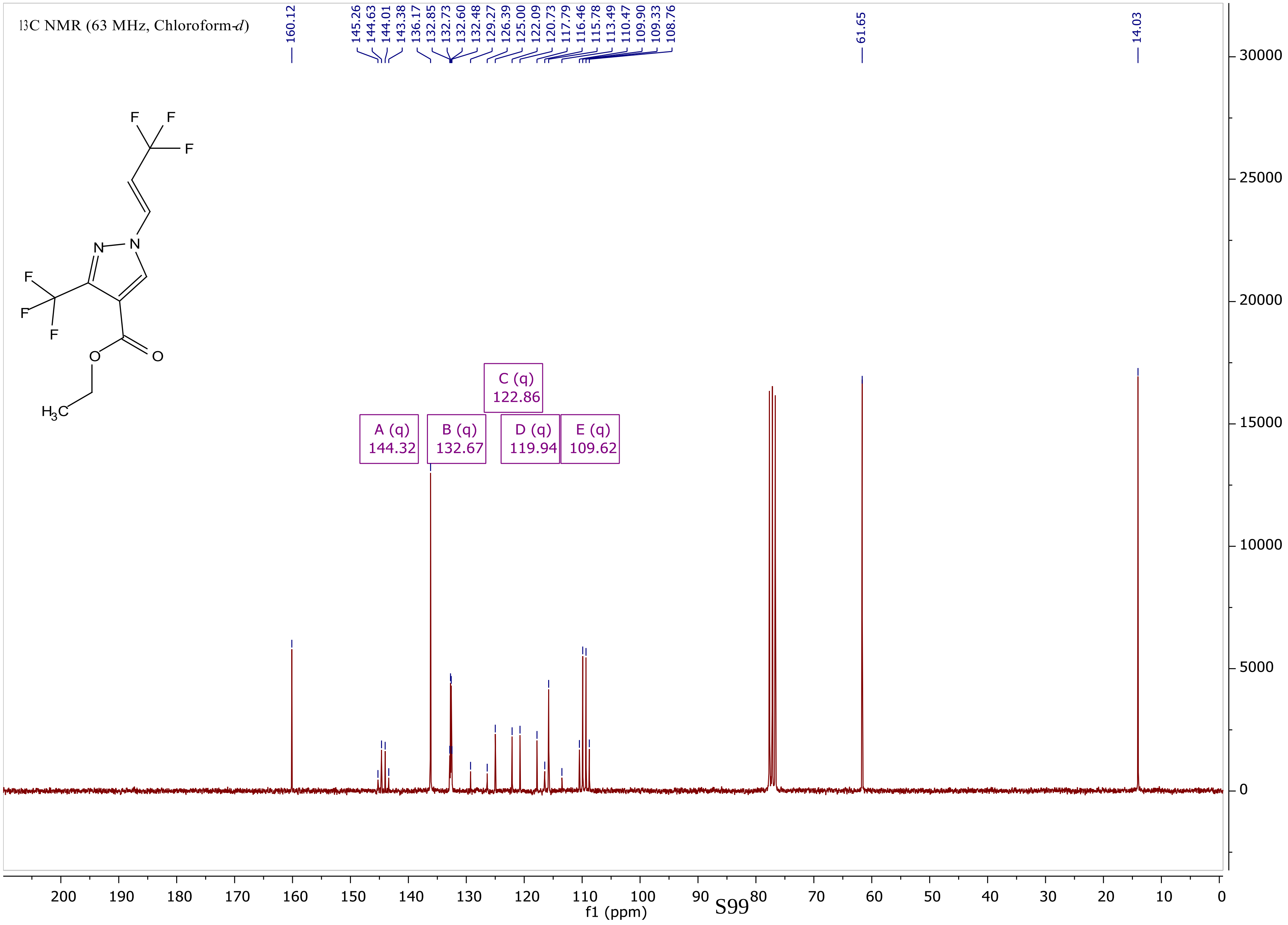
13C NMR (63 MHz, Chloroform-d)
160.12
145.26
144.63
144.01
143.38
136.17
132.85
132.73
132.60
132.48
129.27
126.39
125.00
122.09
120.73
117.79
116.46
115.78
113.49
110.47
109.90
109.33
108.76
61.65
14.03
A (q)
144.32
B (q)
132.67
C (q)
122.86
D (q)
119.94
E (q)
109.62
f1 (ppm)

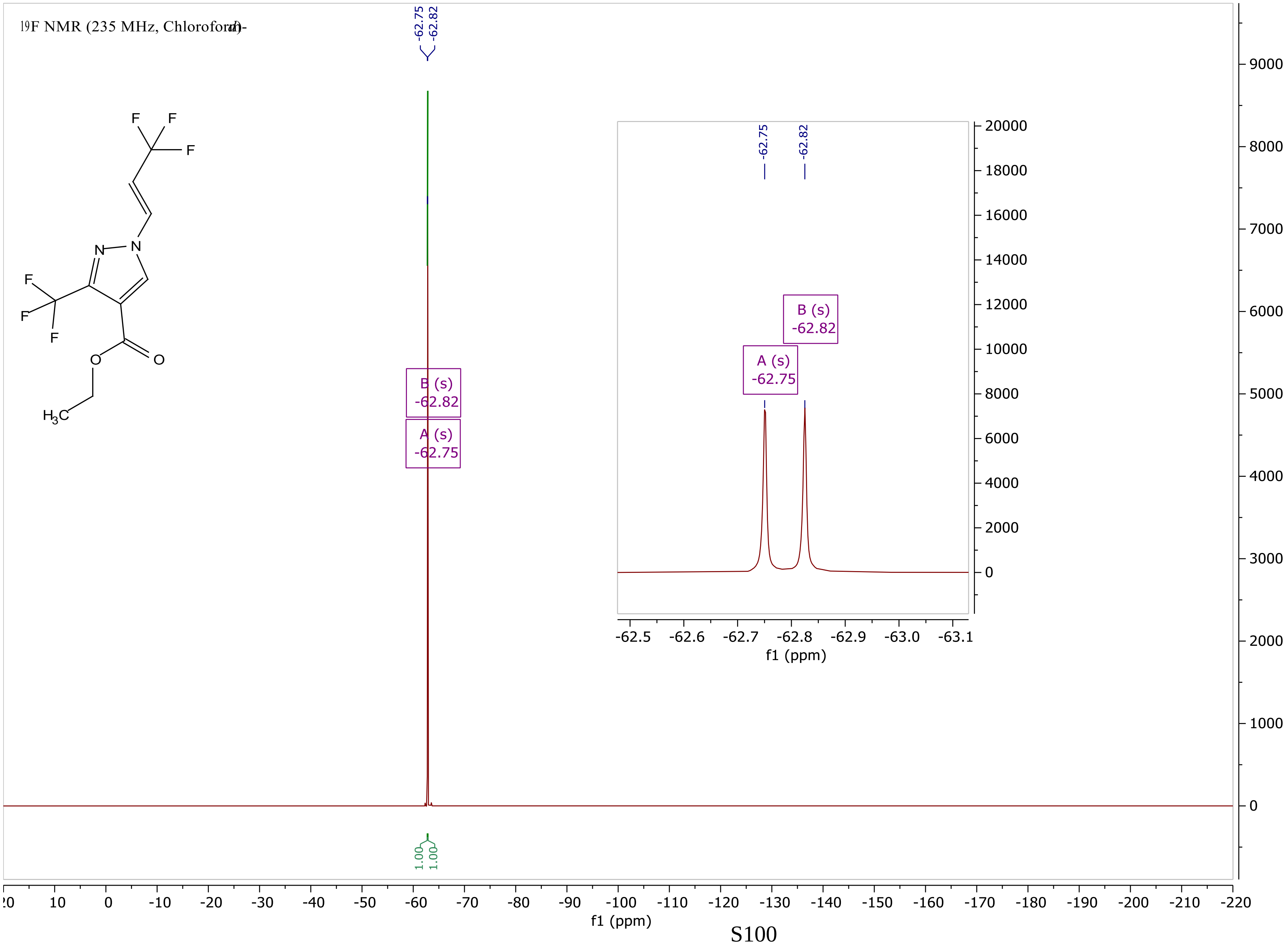
19F NMR (235 MHz, Chloroform-d)
-62.75
-62.82
B (s)
-62.82
A (s)
-62.75
1.00
1.00
f1 (ppm)
f1 (ppm)

(*E*)-3-phenyl-1-(3,3,3-trifluoroprop-1-en-1-yl)-1*H*-pyrazole (16)

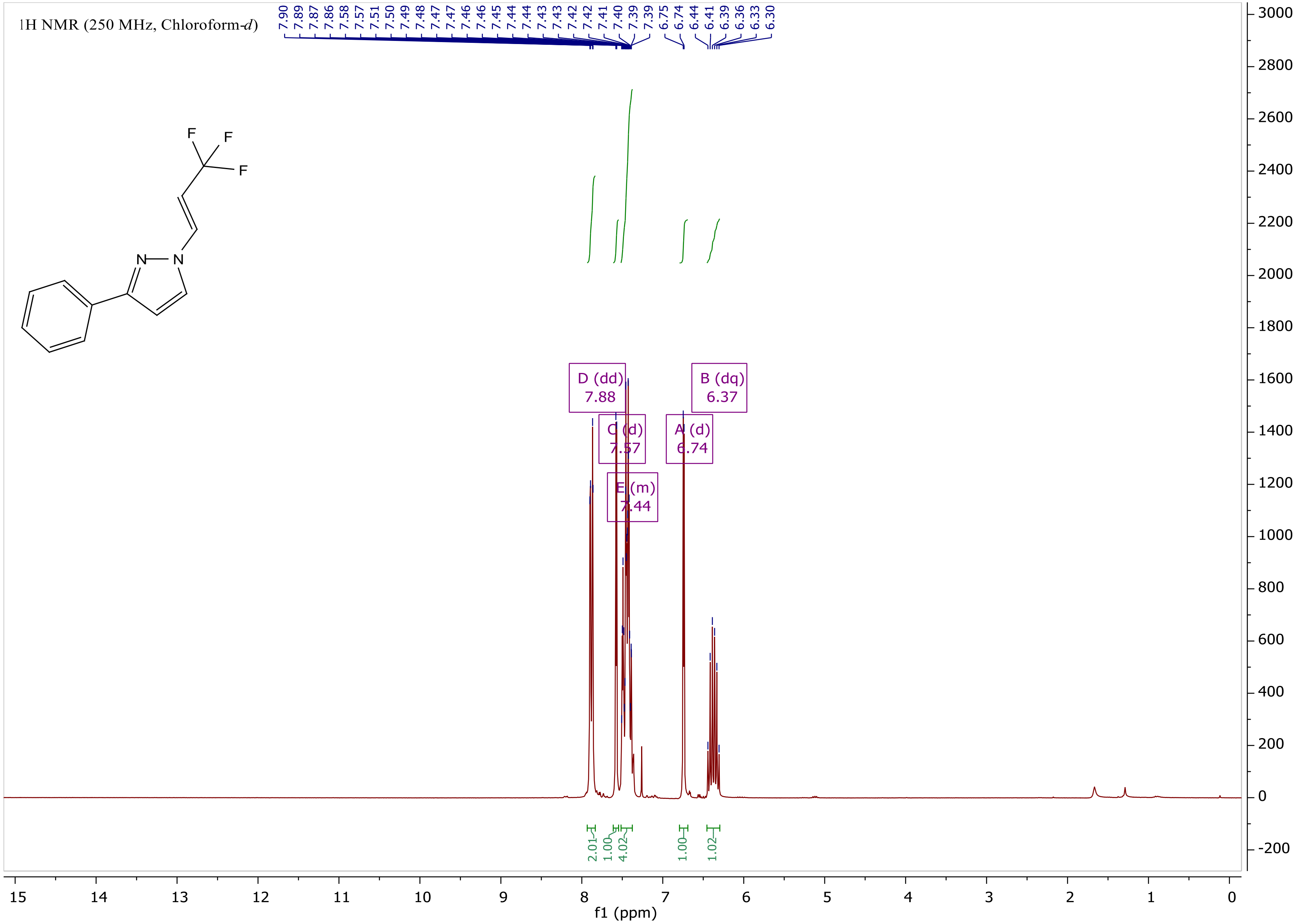

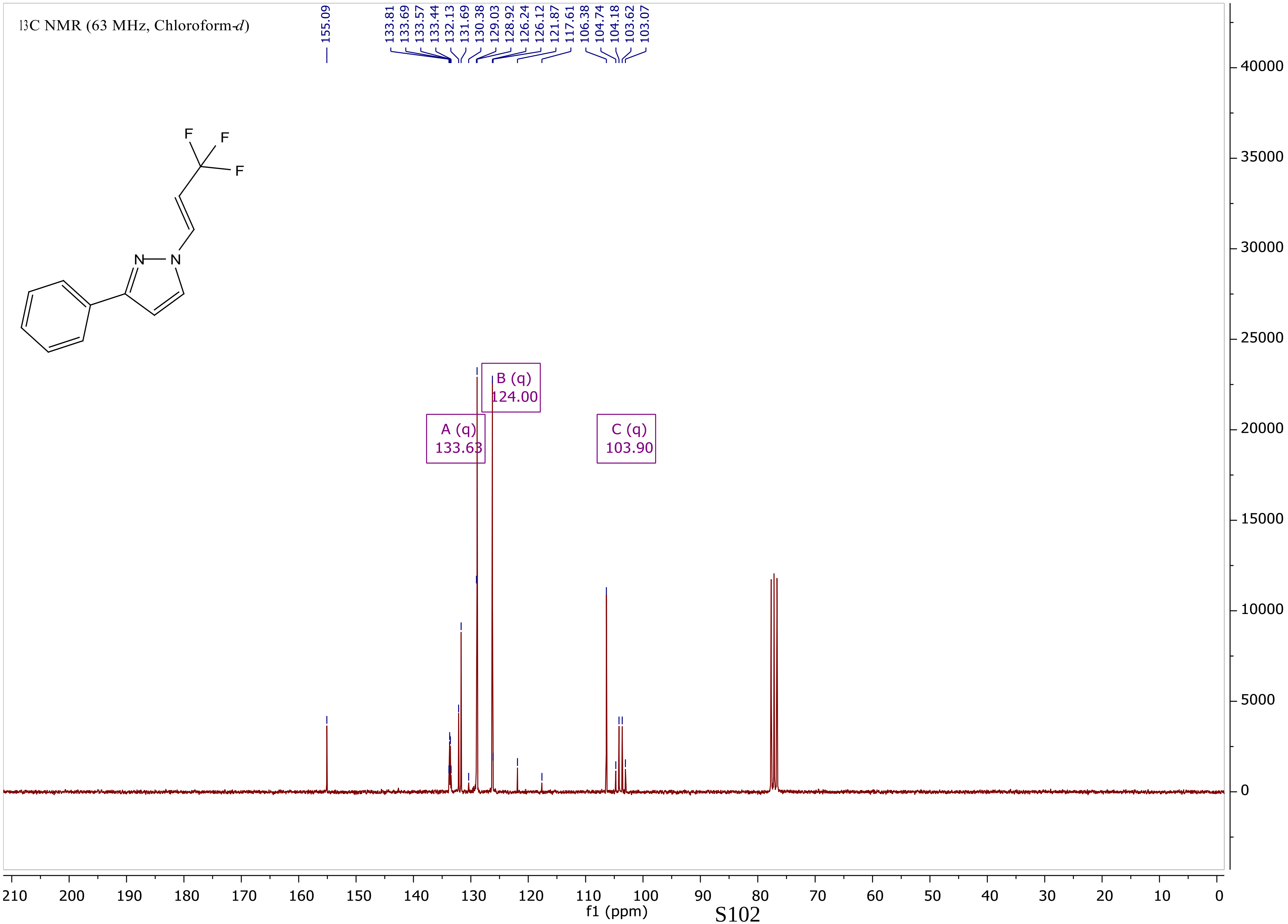
13C NMR (63 MHz, Chloroform-d)
155.09
133.81
133.69
133.57
133.44
132.13
131.69
130.38
129.03
128.92
126.24
126.12
121.87
117.61
106.38
104.74
104.18
103.62
103.07
F
F
F
N
N
B (q)
124.00
A (q)
133.63
C (q)
103.90
f1 (ppm)

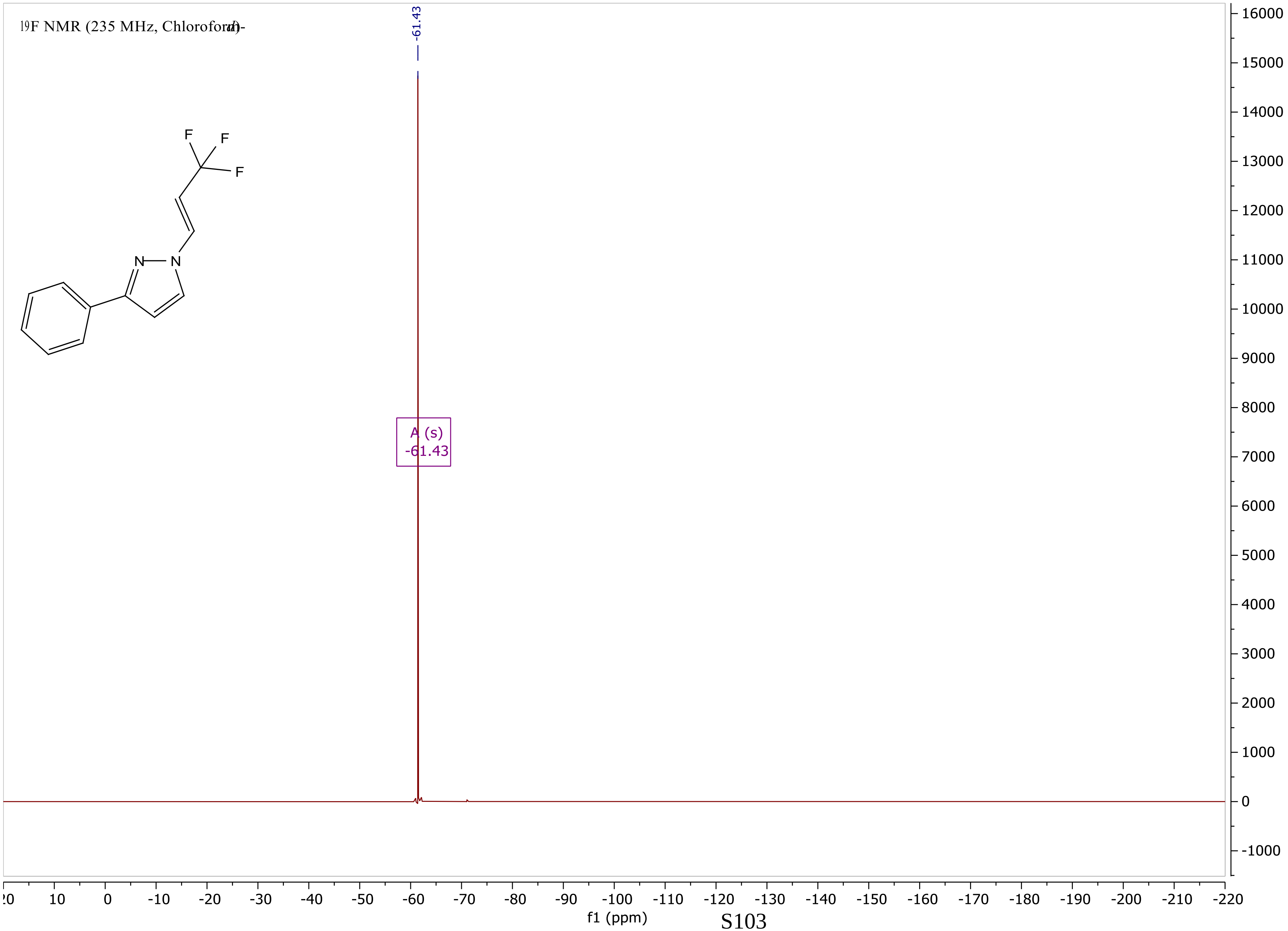
19F NMR (235 MHz, Chloroform-d)
-61.43
A (s)
-61.43
f1 (ppm)

(*E*)-3-(4-Methoxyphenyl)-5-(4-nitrophenyl)-1/2-(3,3,3-trifluoroprop-1-en-1-yl)-1*H*-pyrazole (17) mixture of regioisomers

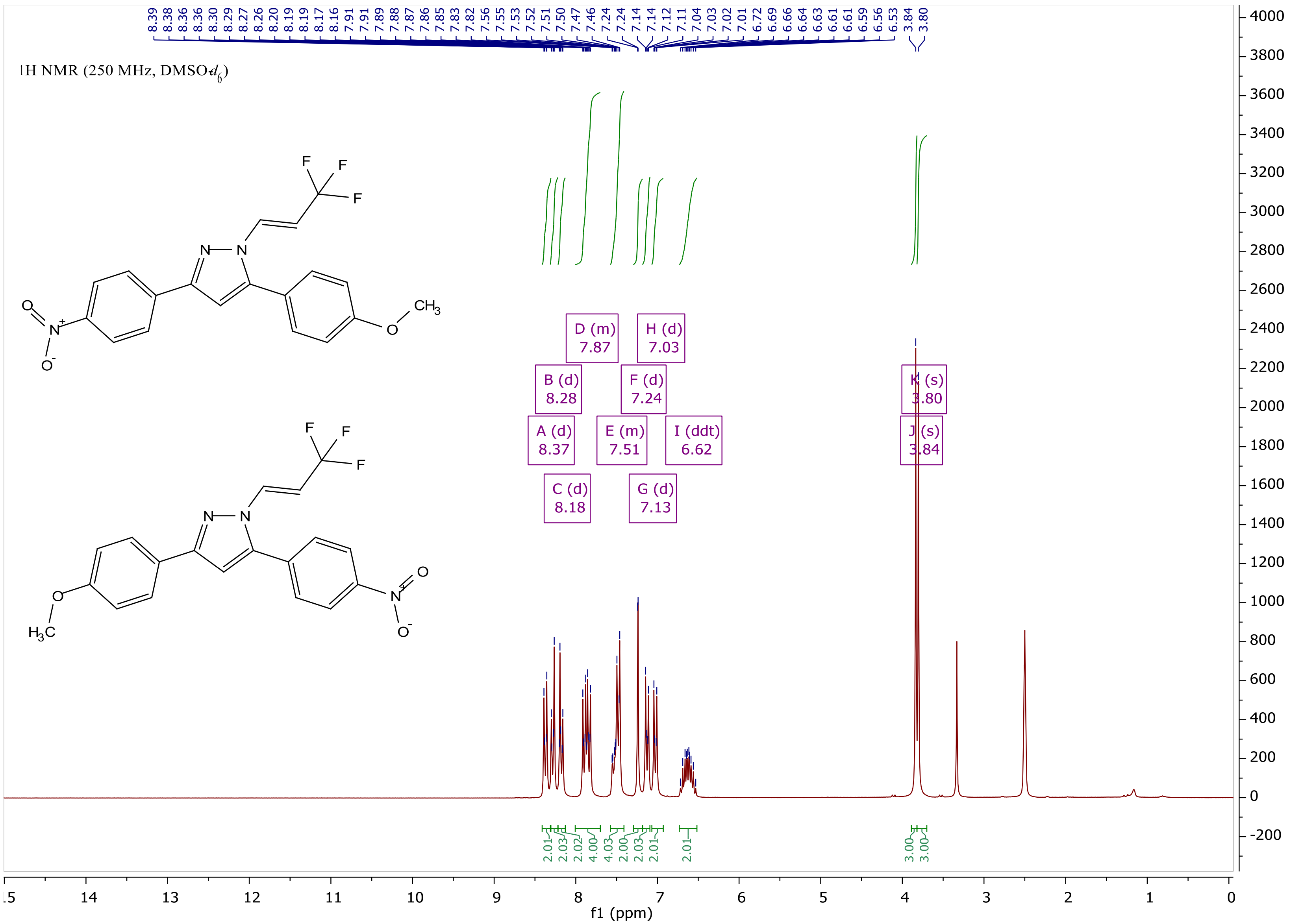

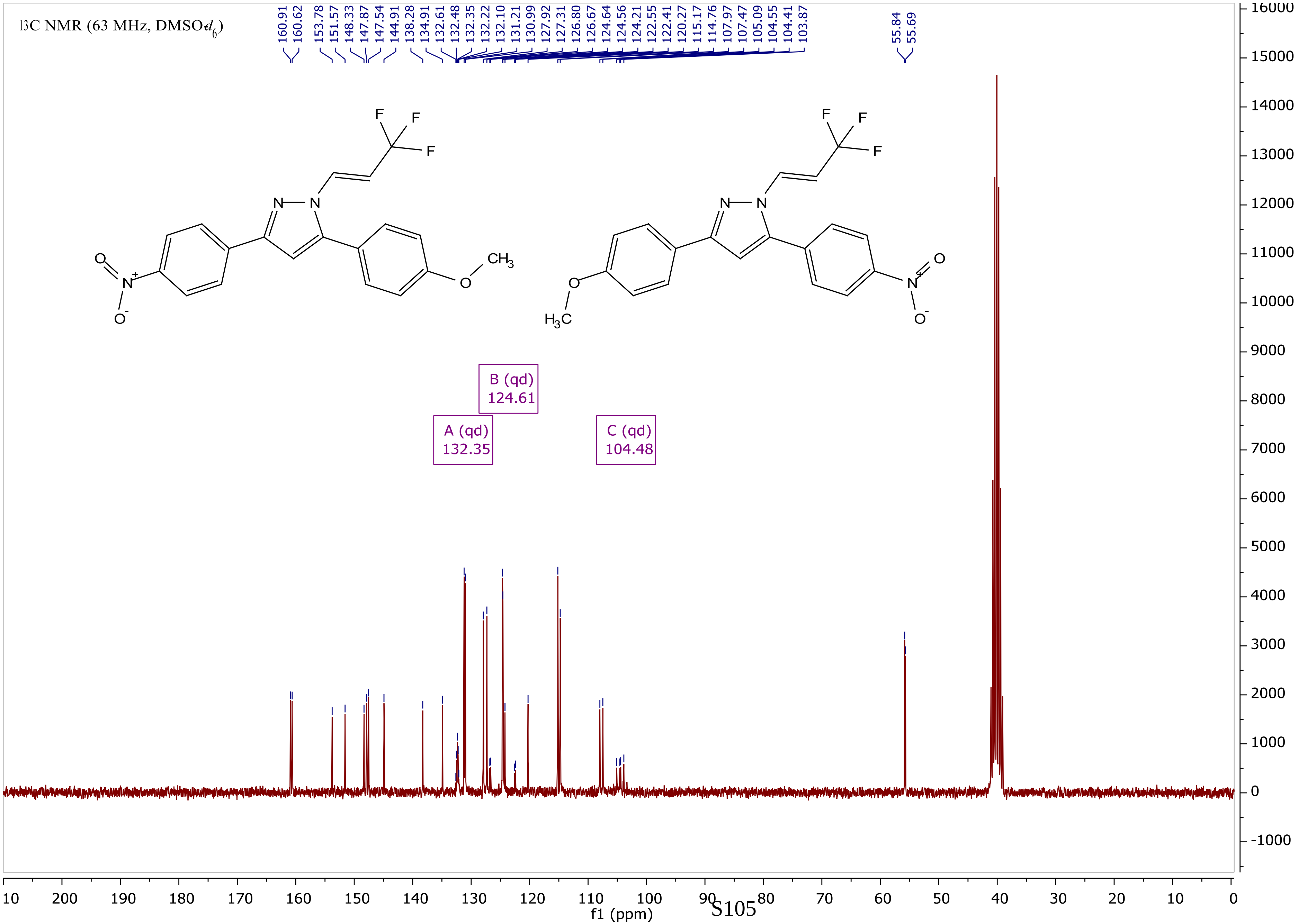
13C NMR (63 MHz, DMSO-d_6)
160.91
160.62
153.78
151.57
148.33
147.87
147.54
144.91
138.28
134.91
132.61
132.48
132.35
132.22
132.10
131.21
130.99
127.92
127.31
126.80
126.67
124.64
124.56
124.21
122.55
122.41
120.27
115.17
114.76
107.97
107.47
105.09
104.55
104.41
103.87
55.84
55.69
A (qd)
132.35
B (qd)
124.61
C (qd)
104.48
f1 (ppm)

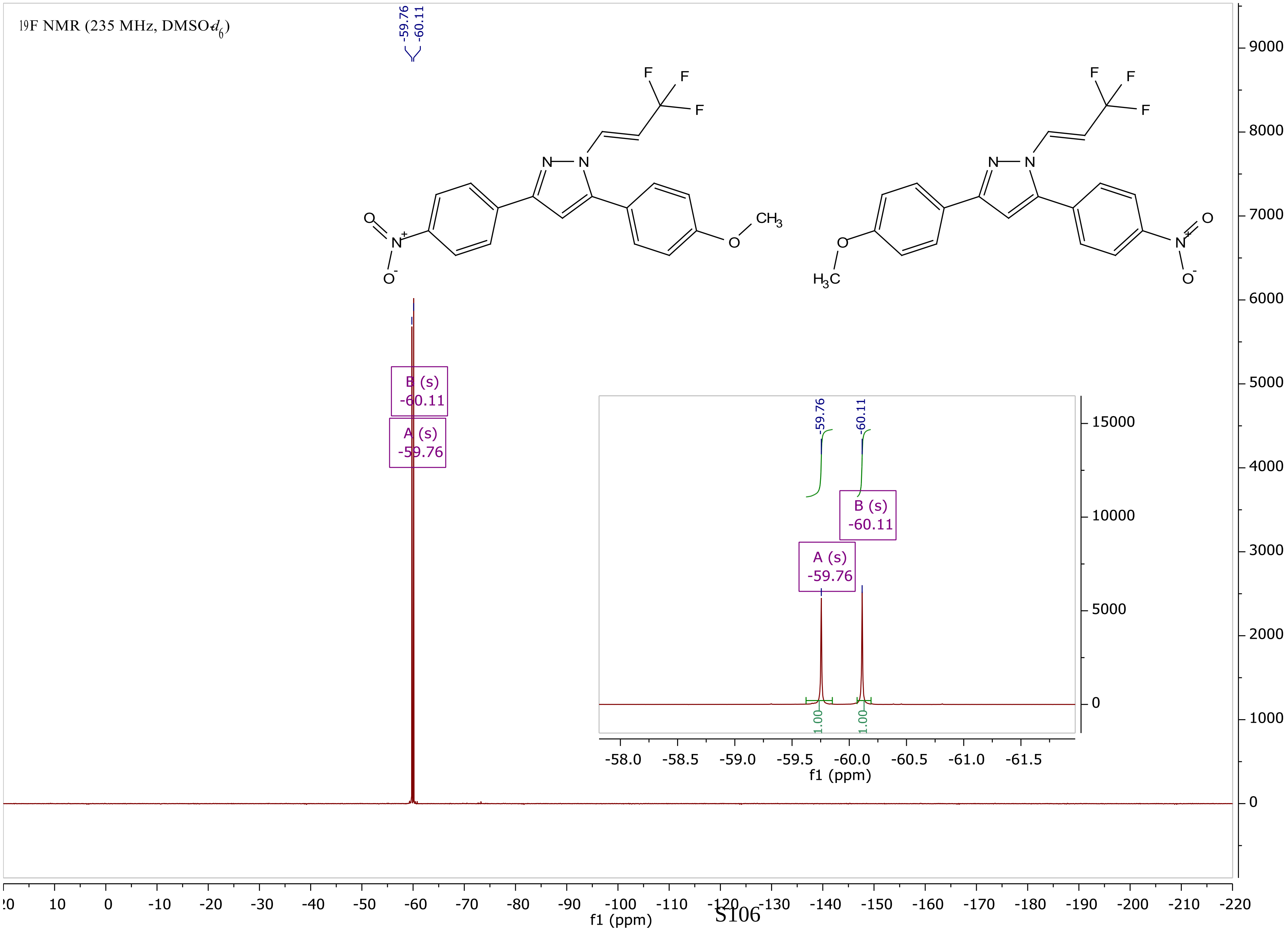
19F NMR (235 MHz, DMSO-d_6)
-59.76
-60.11
F
F
F
N
N
O
N+
O-
CH3
O
H3C
B (s)
-60.11
A (s)
-59.76
1.00
1.00
-58.0
-58.5
-59.0
-59.5
-60.0
-60.5
-61.0
-61.5
f1 (ppm)
15000
10000
5000
0
9000
8000
7000
6000
5000
4000
3000
2000
1000
0
20
10
0
-10
-20
-30
-40
-50
-60
-70
-80
-90
-100
-110
-120
-130
-140
-150
-160
-170
-180
-190
-200
-210
-220
f1 (ppm)

(*E*)-3-Phenyl-5-(thiophen-2-yl)-1/2-(3,3,3-trifluoroprop-1-en-1-yl)-1*H*-pyrazole (18) mixture of regioisomers

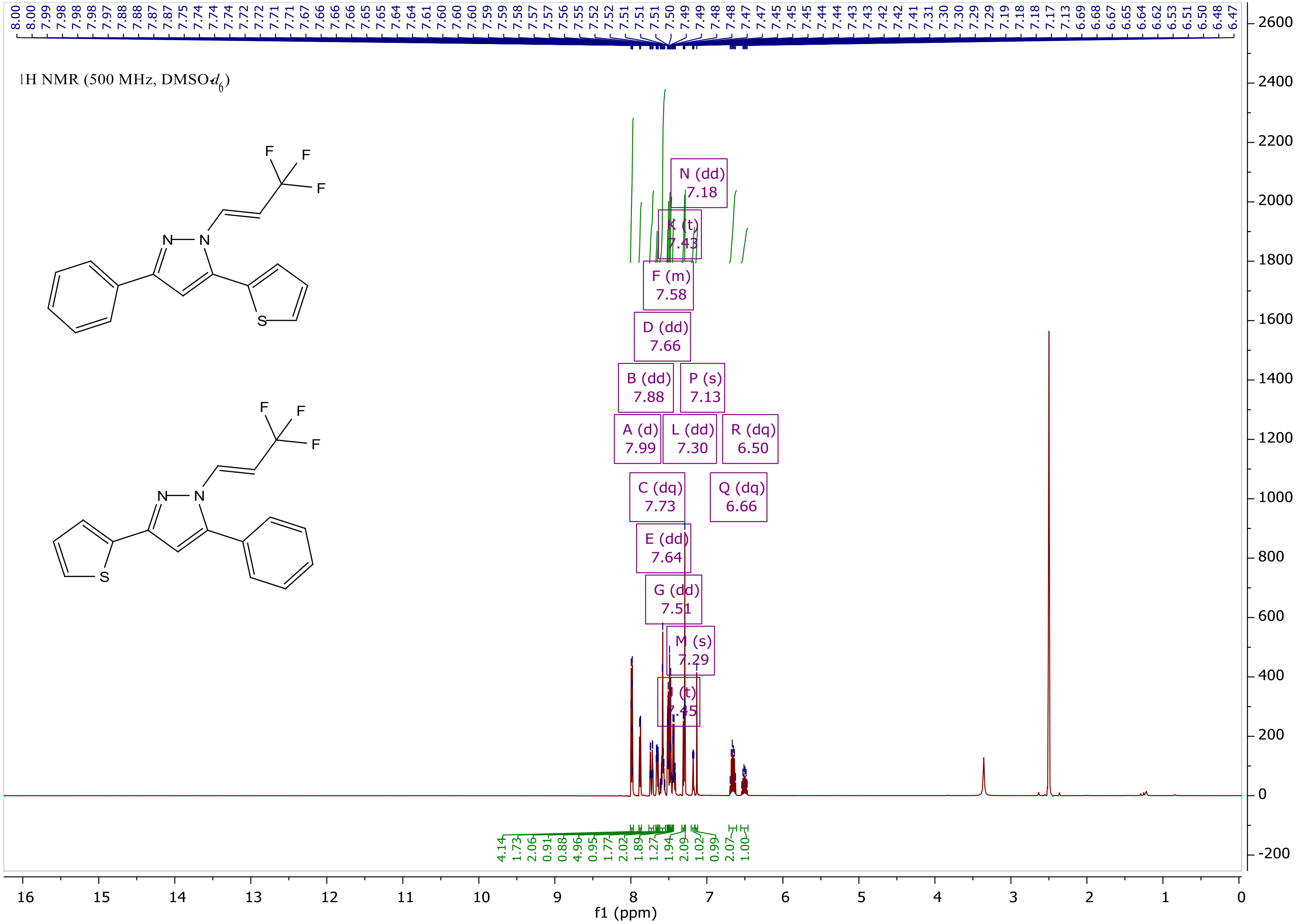

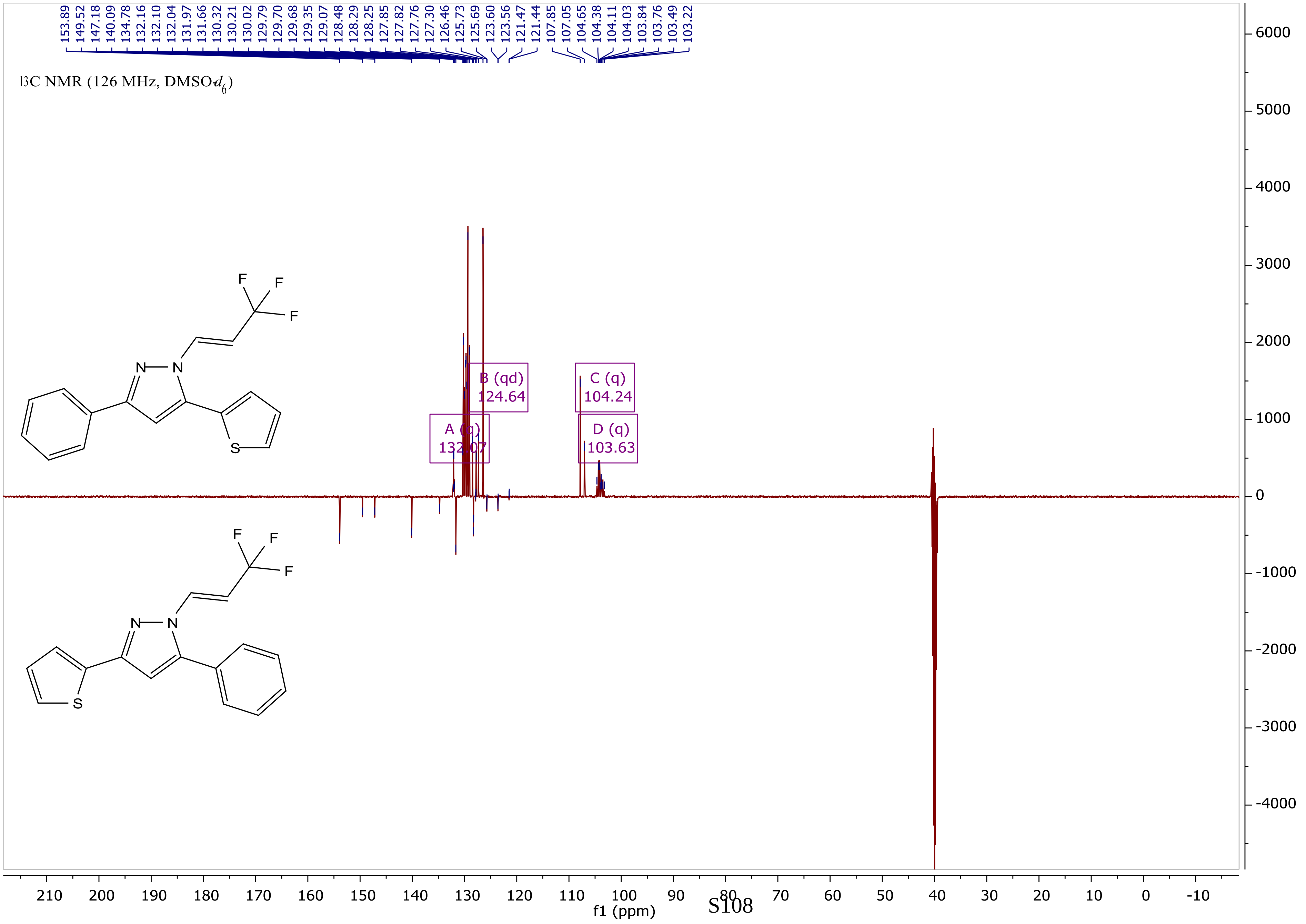
153.89
149.52
147.18
140.09
134.78
132.16
132.10
132.04
131.97
131.66
130.32
130.21
130.02
129.79
129.70
129.68
129.35
129.07
128.48
128.29
128.25
127.85
127.82
127.76
127.30
126.46
125.73
125.69
123.60
123.56
121.47
121.44
107.85
107.05
104.65
104.38
104.11
104.03
103.84
103.76
103.49
103.22
13C NMR (126 MHz, DMSO-d6)
A (q)
132.07
B (qd)
124.64
C (q)
104.24
D (q)
103.63
f1 (ppm)

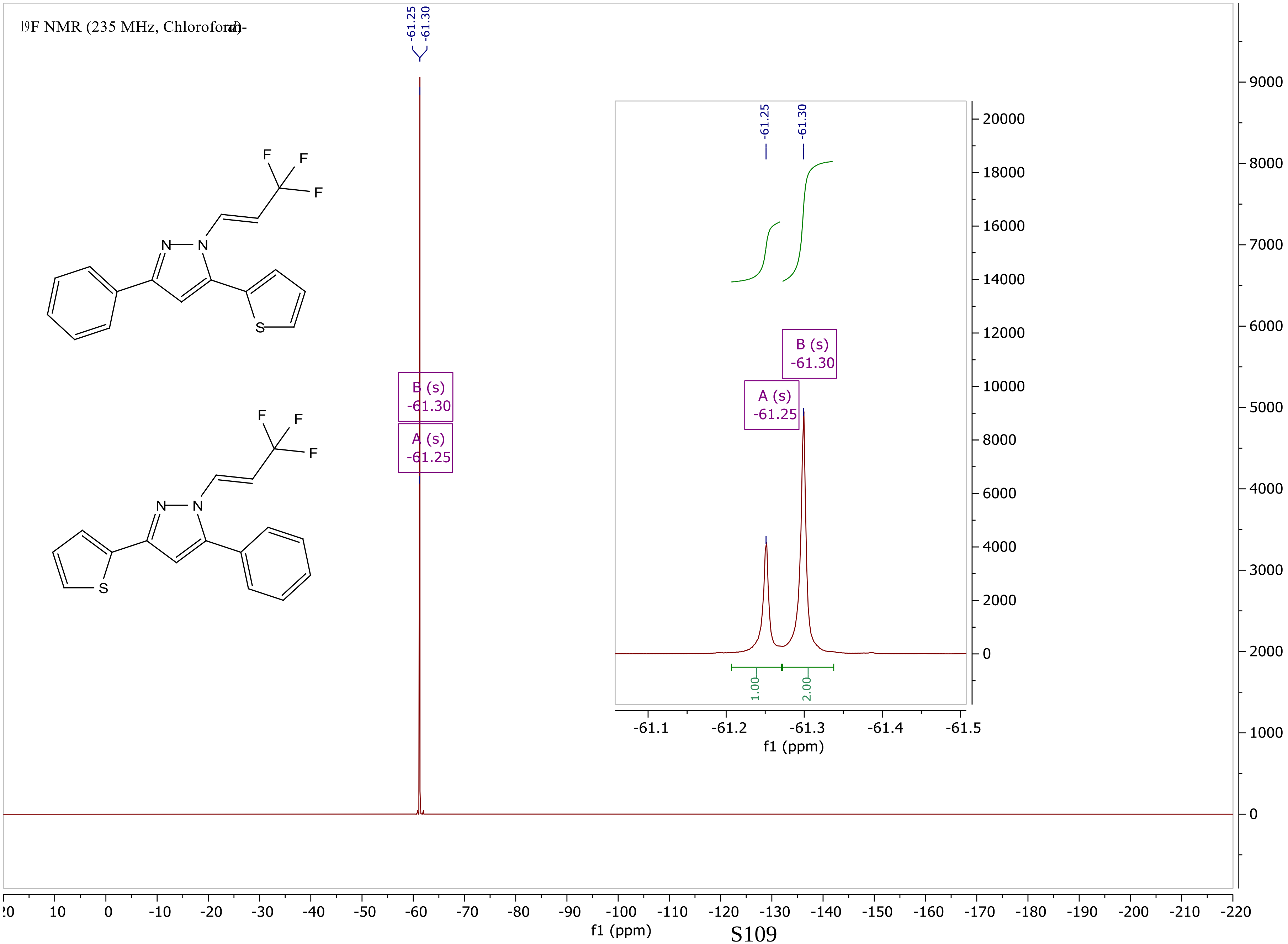

19F NMR (235 MHz, Chloroform-d)
-61.25
-61.30
B (s)
-61.30
A (s)
-61.25
1.00
2.00
f1 (ppm)

(*E*)-3-(2-Tolyl)-5-(4-tolyl)-1-(3,3,3-trifluoroprop-1-en-1-yl)-1*H*-pyrazole (19)

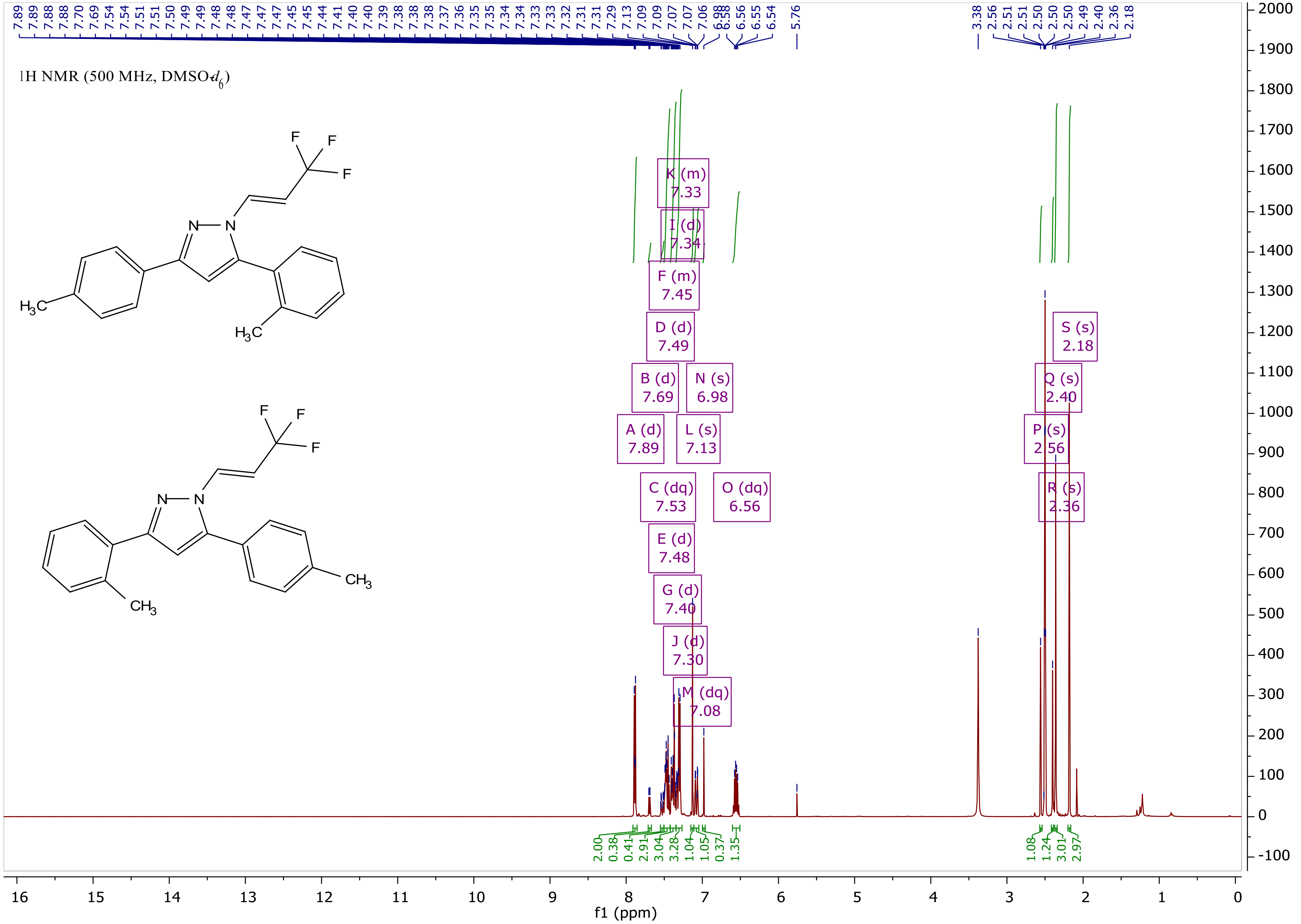

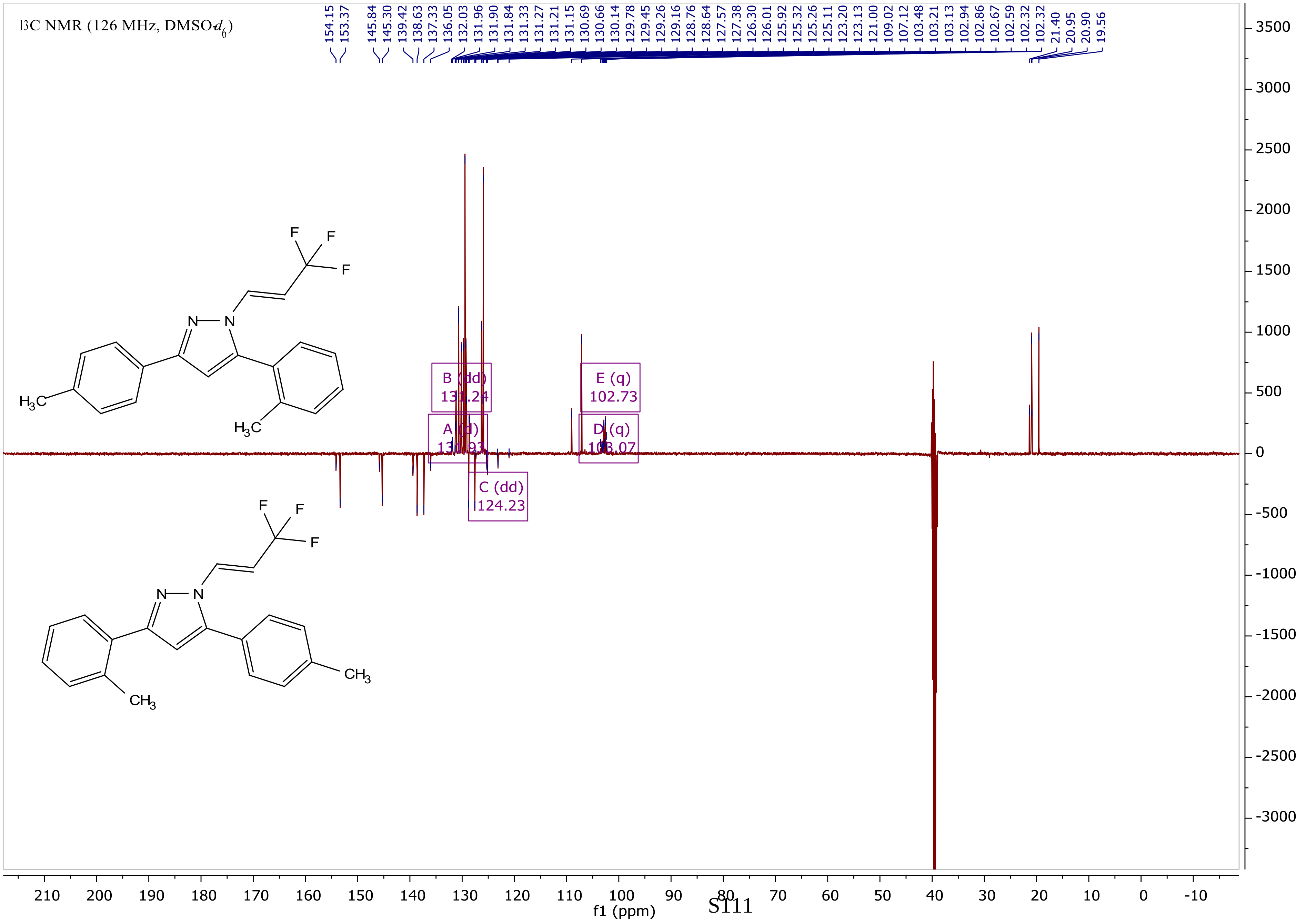
13C NMR (126 MHz, DMSO-d_6)
154.15
153.37
145.84
145.30
139.42
138.63
137.33
136.05
132.03
131.96
131.90
131.84
131.33
131.27
131.21
131.15
130.69
130.66
130.14
129.78
129.45
129.26
129.16
128.76
128.64
127.57
127.38
126.30
126.01
125.92
125.32
125.26
125.11
123.20
123.13
121.00
109.02
107.12
103.48
103.21
103.13
102.94
102.86
102.67
102.59
102.32
102.32
21.40
20.95
20.90
19.56
B (dd)
131.24
A (d)
130.93
C (dd)
124.23
E (q)
102.73
D (q)
103.07
f1 (ppm)

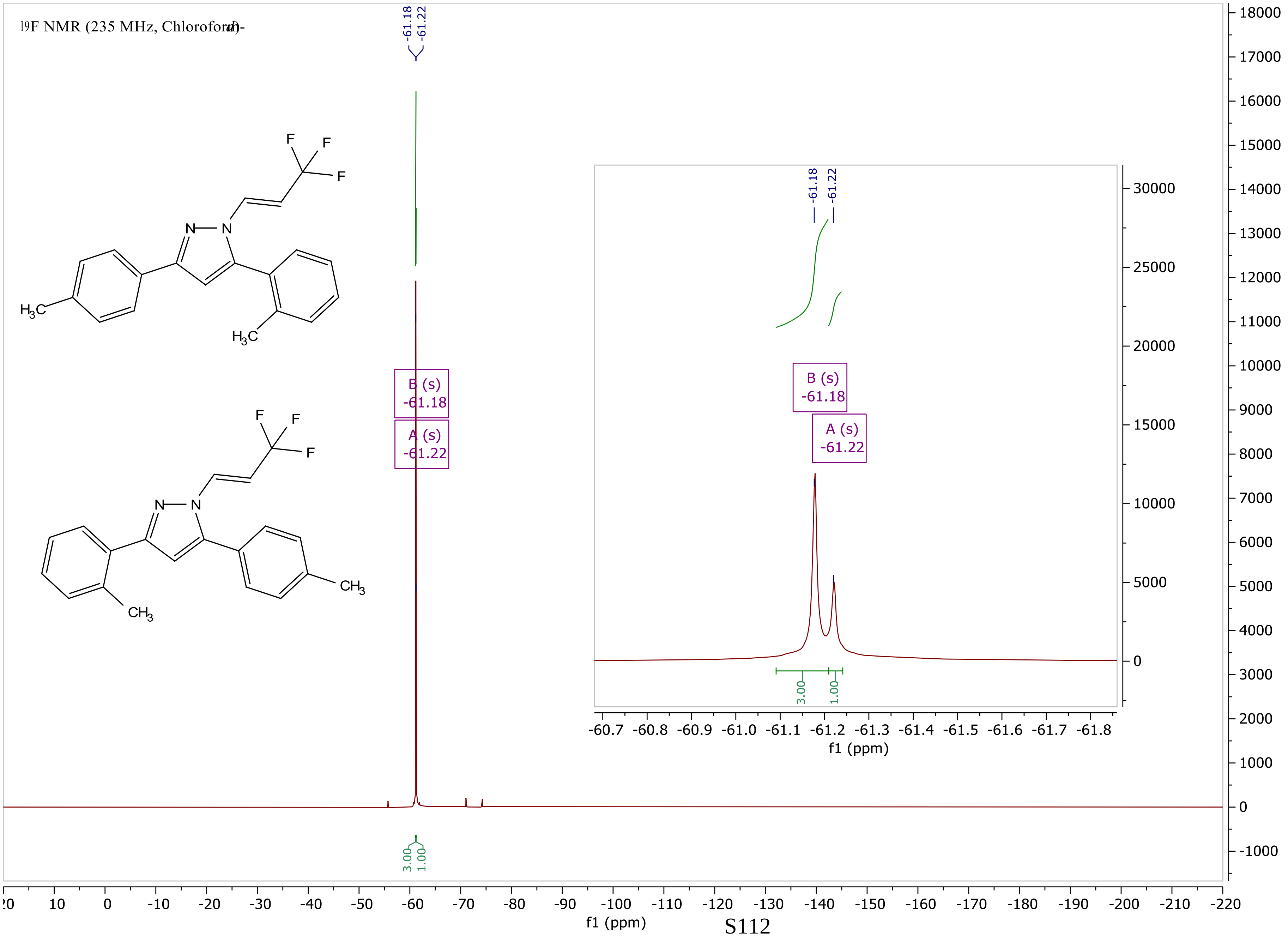
19F NMR (235 MHz, Chloroform-d)
-61.18
-61.22
B (s) -61.18
A (s) -61.22
3.00
1.00
f1 (ppm)
F
N
N
H3C
CH3

(*E*)-3-Phenyl-5-(trifluoromethyl)-1-(3,3,3-trifluoroprop-1-en-1-yl)-1*H*-pyrazole (20) isomer1

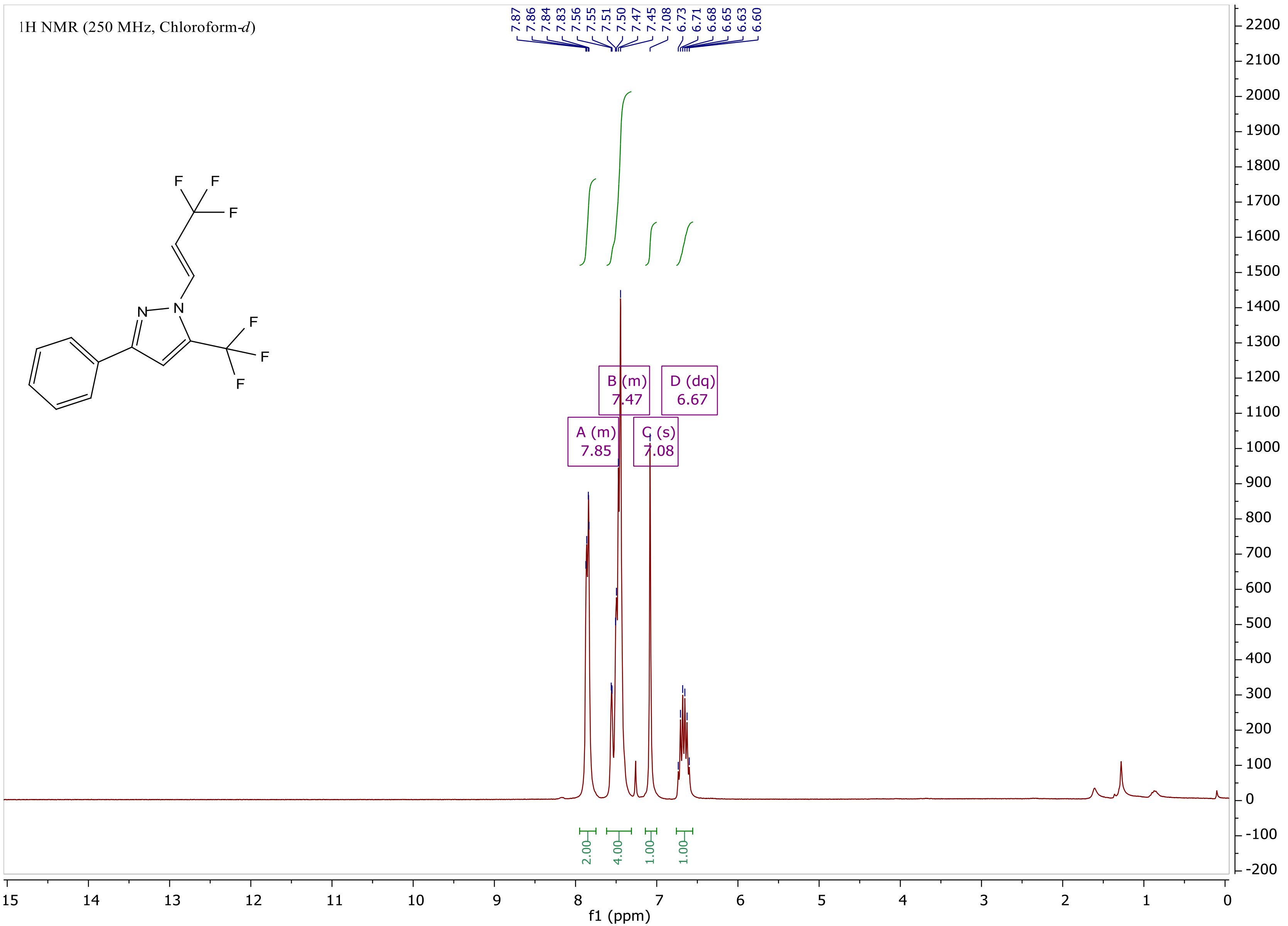

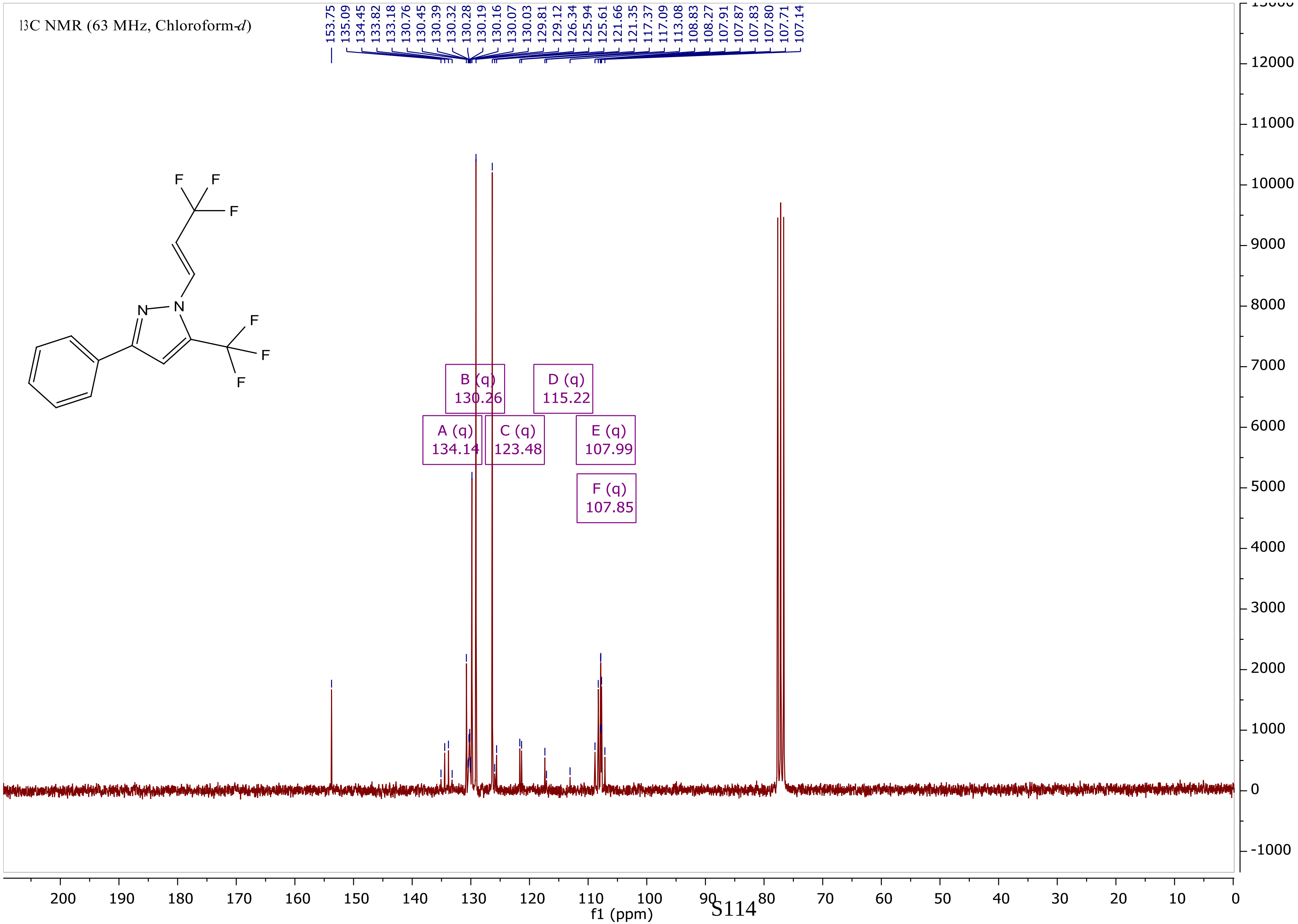
13C NMR (63 MHz, Chloroform-d)
153.75
135.09
134.45
133.82
133.18
130.76
130.45
130.39
130.32
130.28
130.19
130.16
130.07
130.03
129.81
129.12
126.34
125.94
125.61
121.66
121.35
117.37
117.09
113.08
108.83
108.27
107.91
107.87
107.83
107.80
107.71
107.14
A (q) 134.14
B (q) 130.26
C (q) 123.48
D (q) 115.22
E (q) 107.99
F (q) 107.85
f1 (ppm)

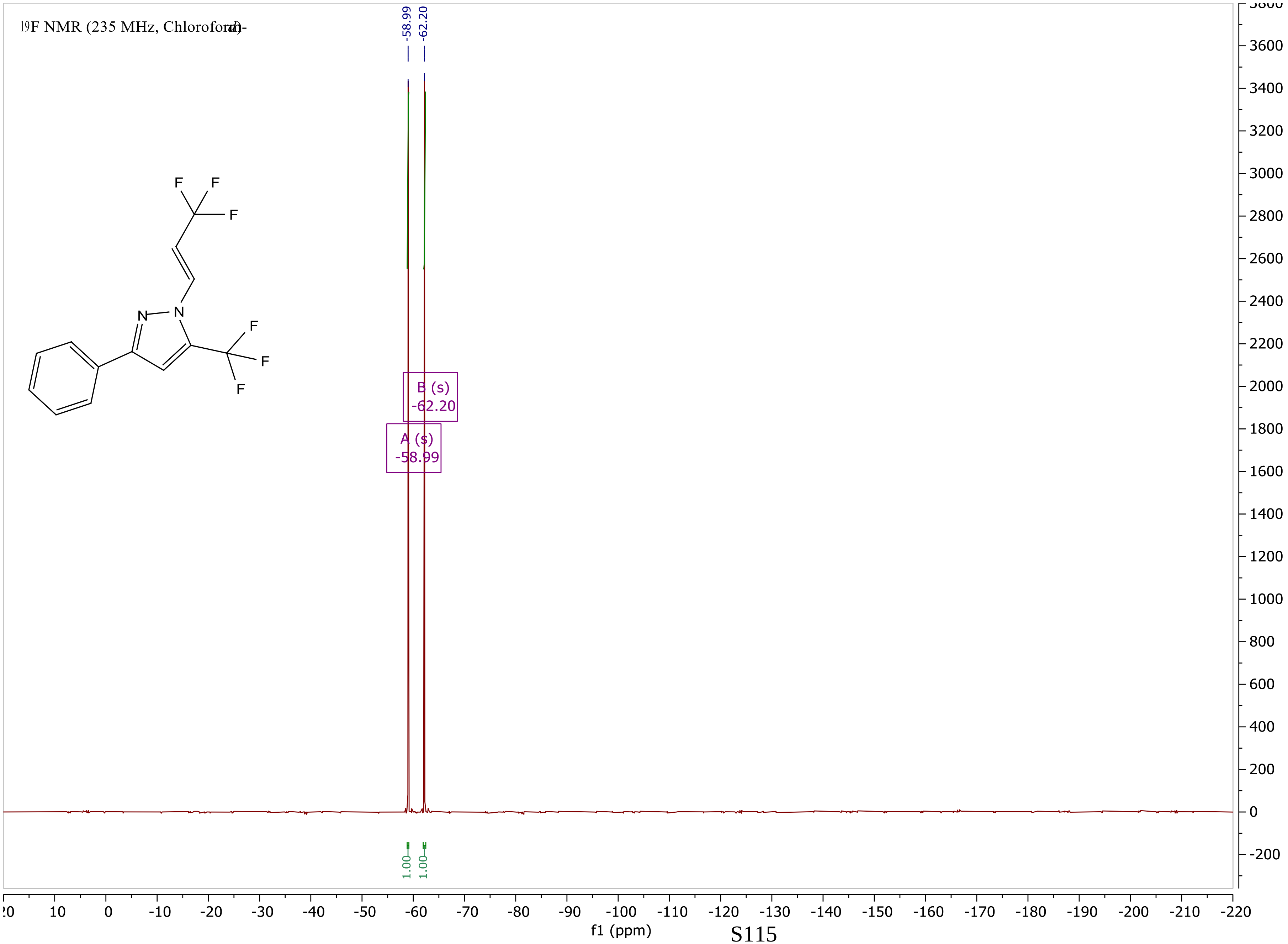
19F NMR (235 MHz, Chloroform-d)
-58.99
-62.20
A (s)
-58.99
B (s)
-62.20
1.00
1.00
f1 (ppm)

(*E*)-5-Phenyl-3-(trifluoromethyl)-1-(3,3,3-trifluoroprop-1-en-1-yl)-1*H*-pyrazole (21) isomer2

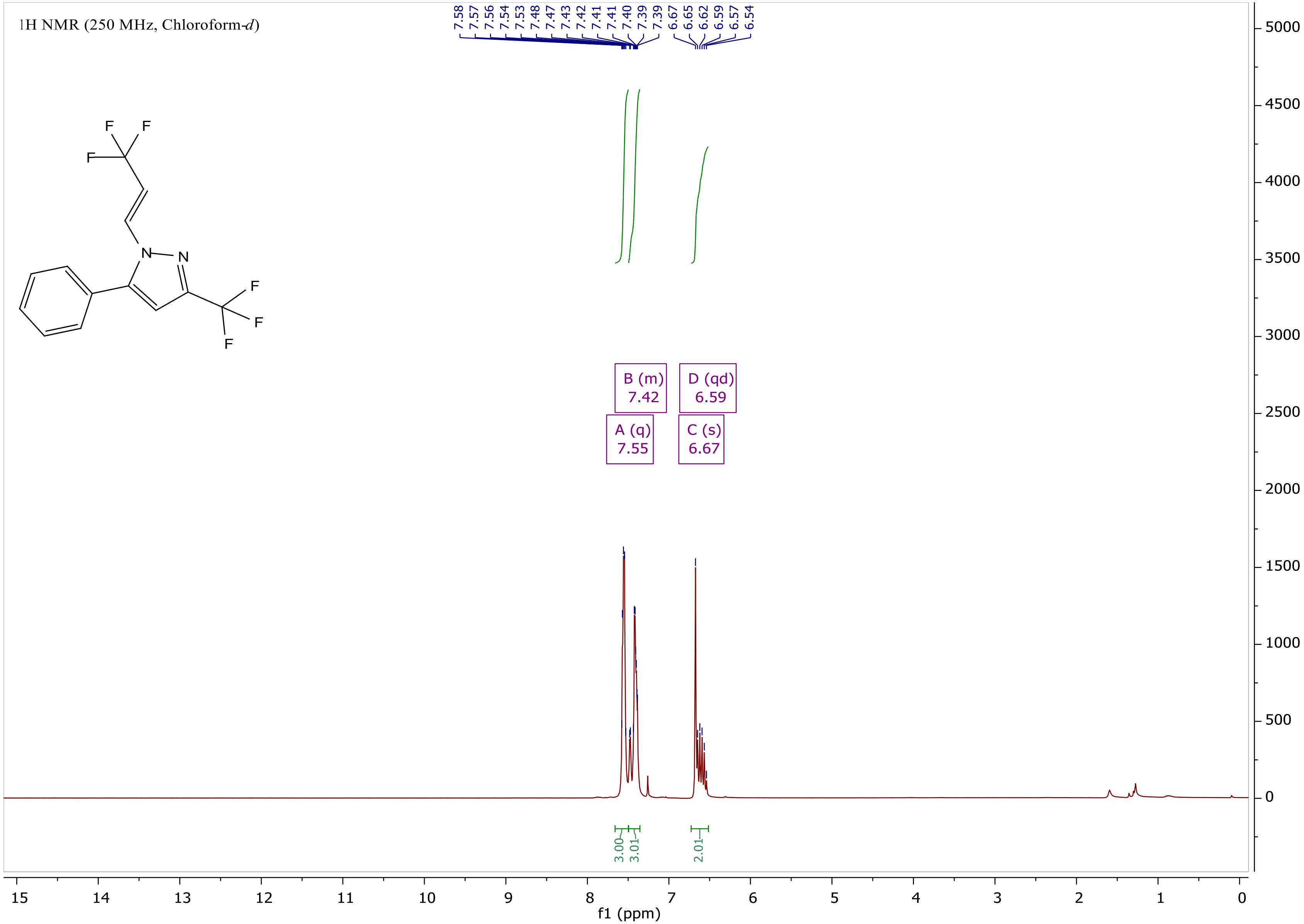

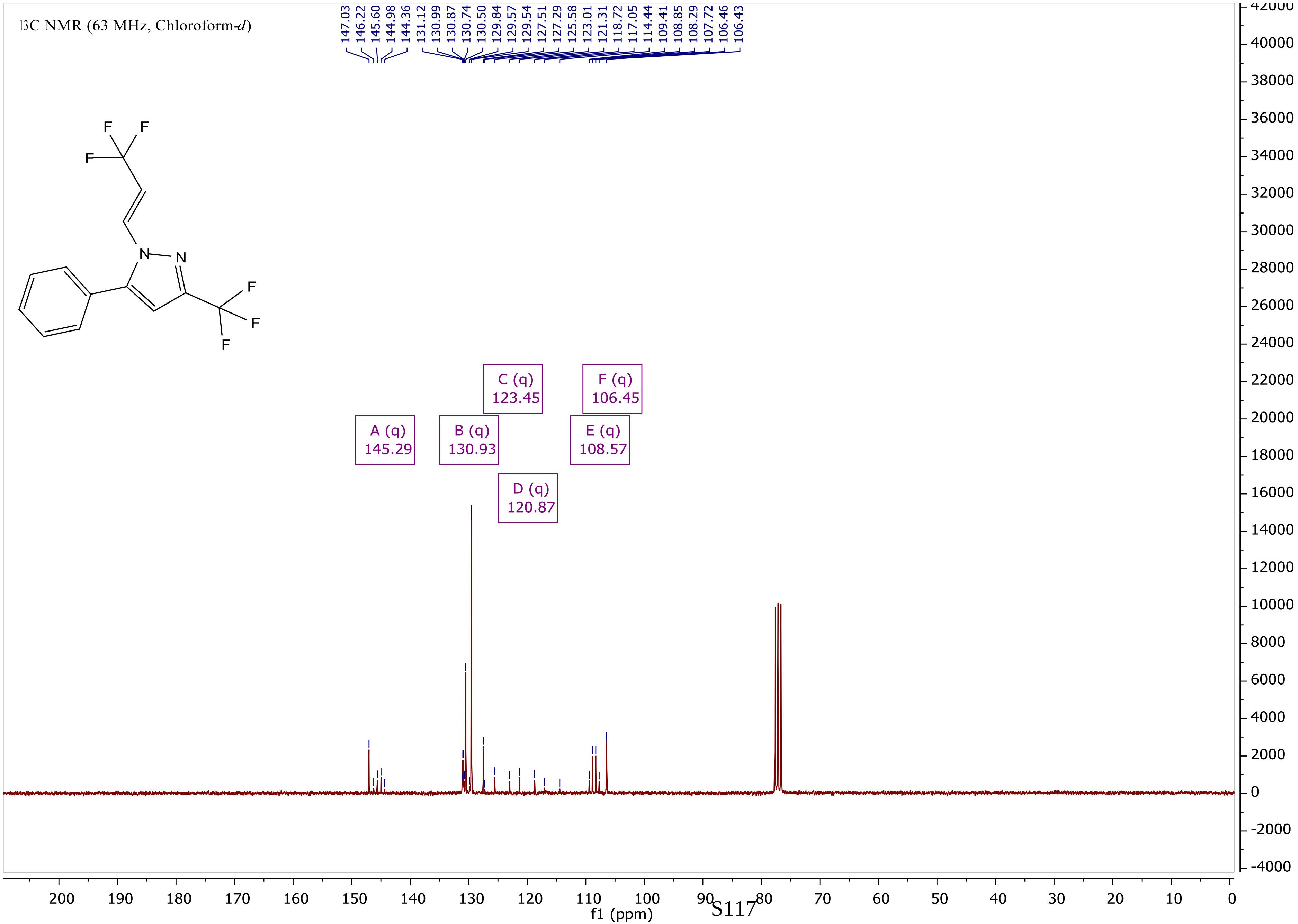
13C NMR (63 MHz, Chloroform-d)
147.03
146.22
145.60
144.98
144.36
131.12
130.99
130.87
130.74
130.50
129.84
129.57
129.54
127.51
127.29
125.58
123.01
121.31
118.72
117.05
114.44
109.41
108.85
108.29
107.72
106.46
106.43
A (q) 145.29
B (q) 130.93
C (q) 123.45
D (q) 120.87
E (q) 108.57
F (q) 106.45
f1 (ppm)

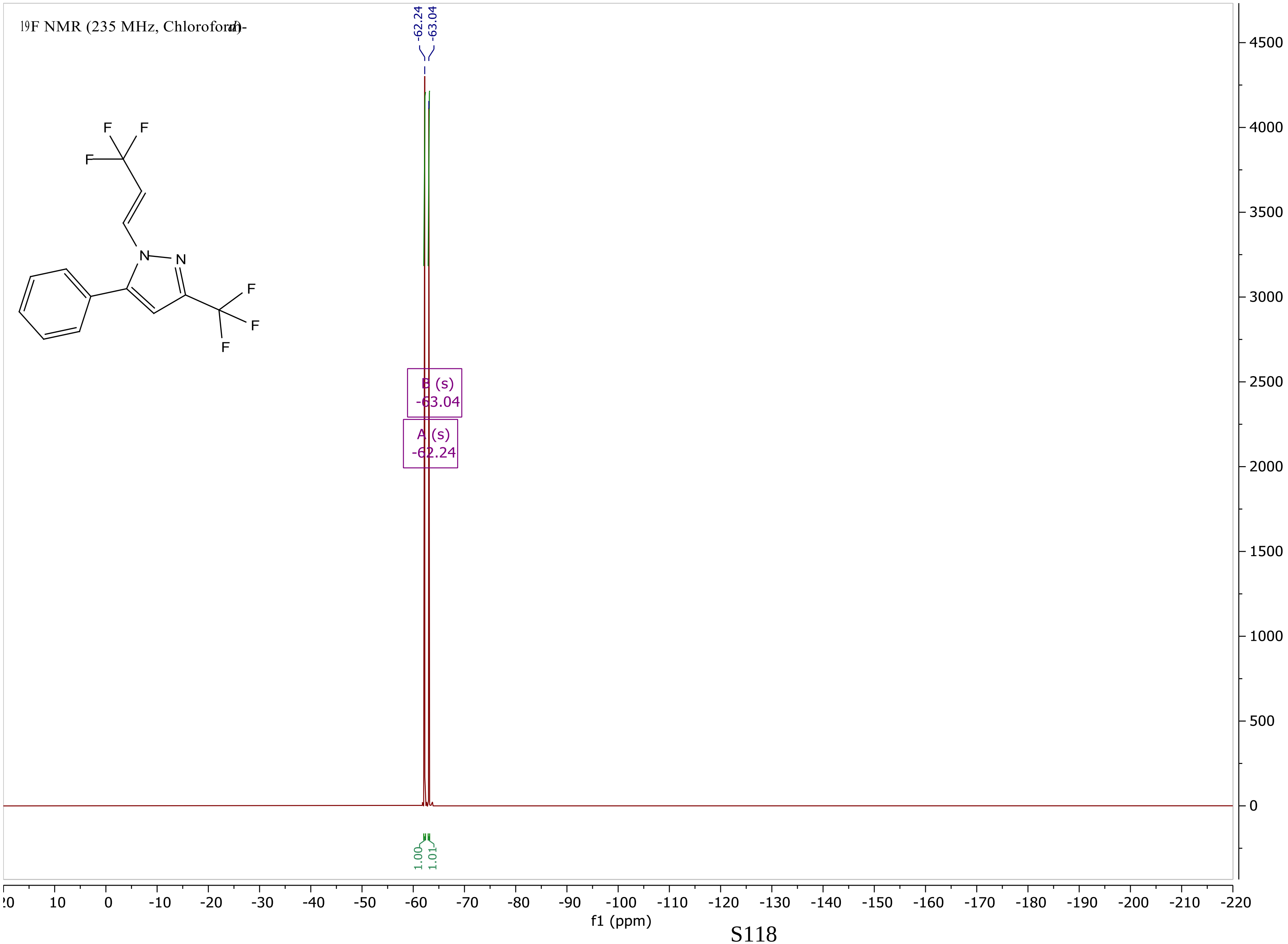
19F NMR (235 MHz, Chloroform-d)
-62.24
-63.04
F
F
F
N
N
F
F
F
B (s)
-63.04
A (s)
-62.24
4500
4000
3500
3000
2500
2000
1500
1000
500
0
1.00
1.01
20
10
0
-10
-20
-30
-40
-50
-60
-70
-80
-90
-100
-110
-120
-130
-140
-150
-160
-170
-180
-190
-200
-210
-220
f1 (ppm)

Ethyl (*Z*)-*N*-(4-cyano-1-((*E*)-3,3,3-trifluoroprop-1-en-1-yl)-1*H*-pyrazol-5-yl)acetimidate (22) isomer1,

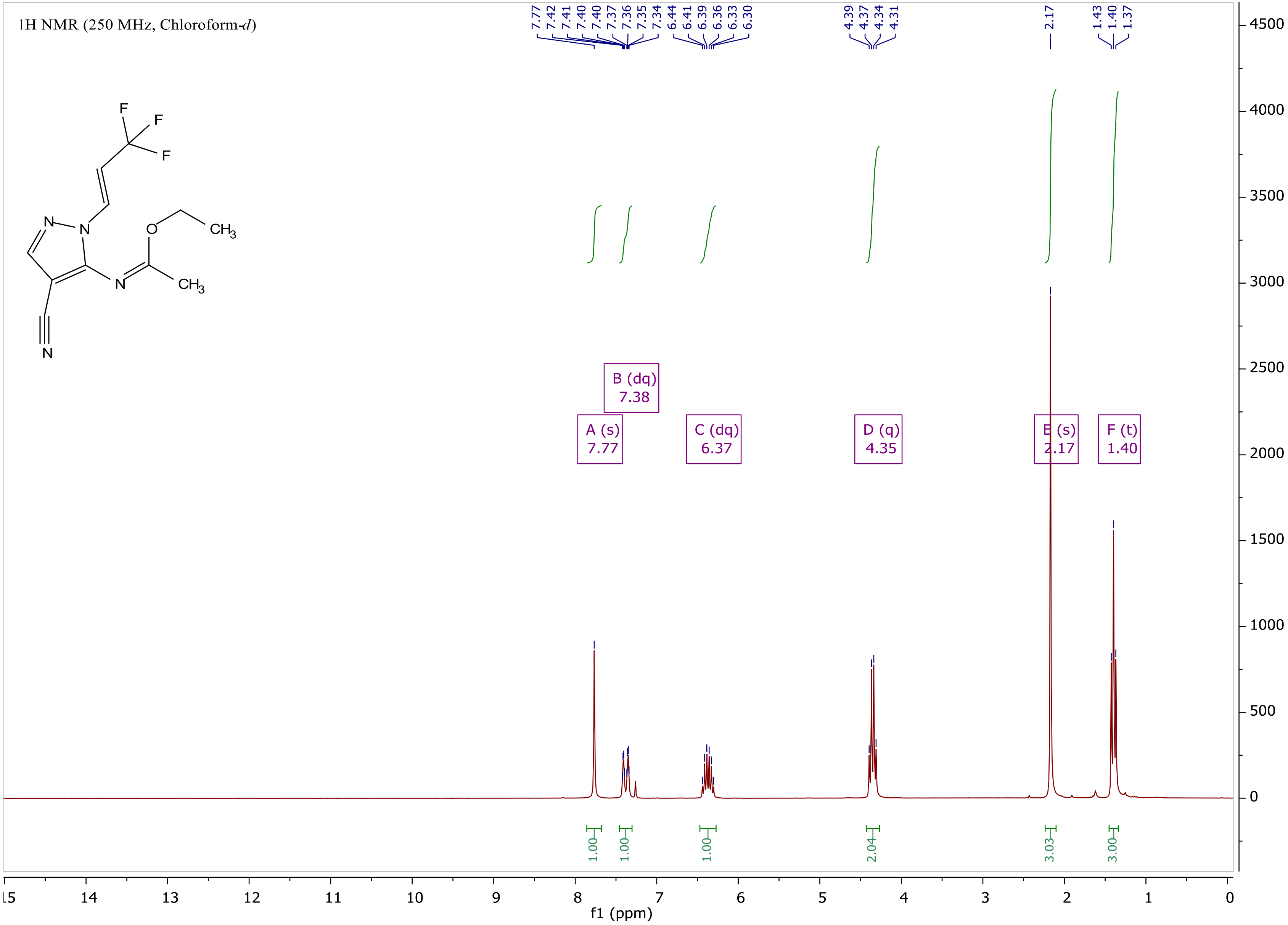

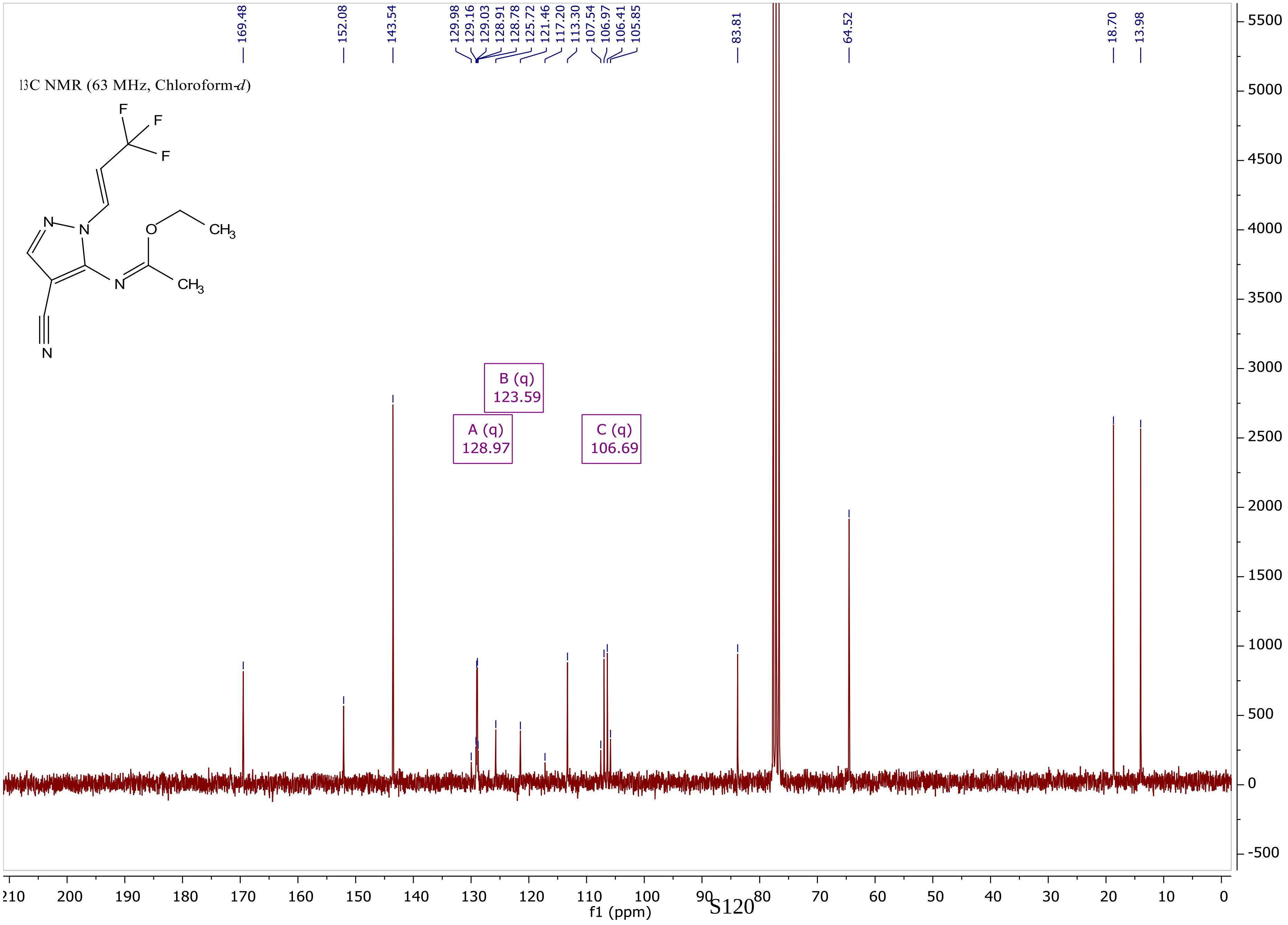
13C NMR (63 MHz, Chloroform-d)
169.48
152.08
143.54
129.98
129.16
129.03
128.91
128.78
125.72
121.46
117.20
113.30
107.54
106.97
106.41
105.85
83.81
64.52
18.70
13.98
B (q) 123.59
A (q) 128.97
C (q) 106.69
F
F
F
N
N
O
CH3
N
CH3
N
f1 (ppm)

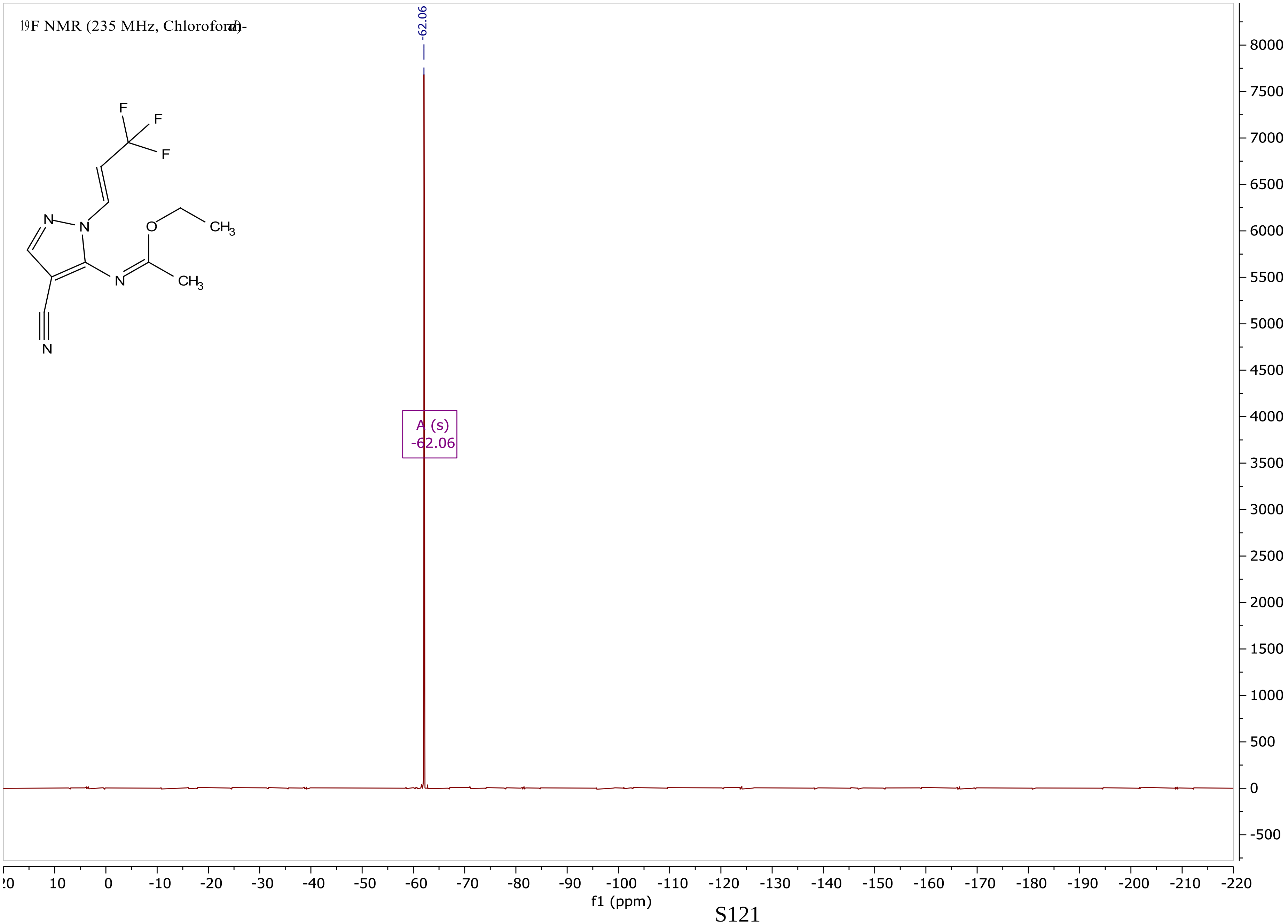
19F NMR (235 MHz, Chloroform-d)
-62.06
A (s)
-62.06
f1 (ppm)

Ethyl (*E*)-*N*-(4-cyano-1-((*E*)-3,3,3-trifluoroprop-1-en-1-yl)-1*H*-pyrazol-3-yl)acetimidate (23) isomer2

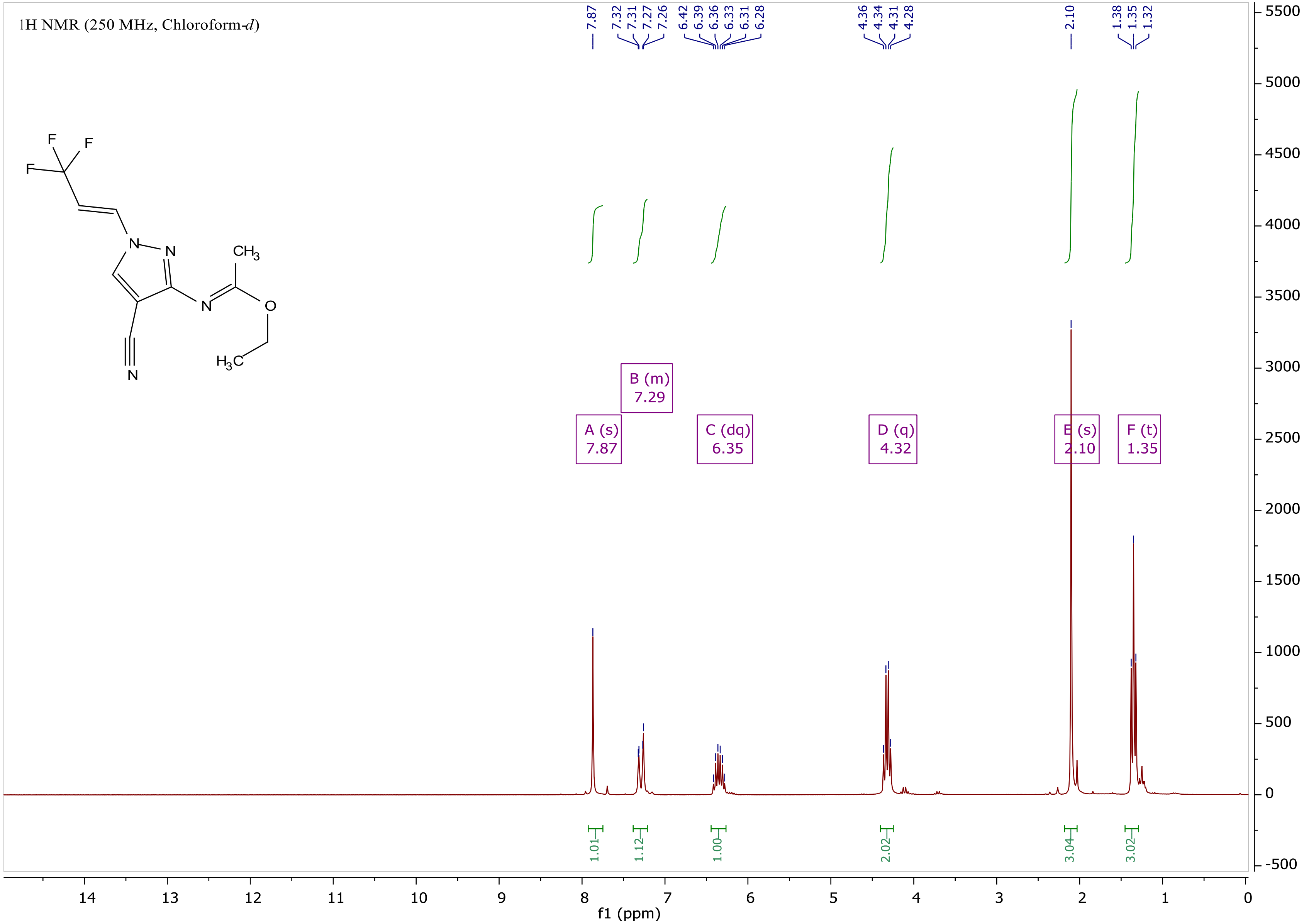

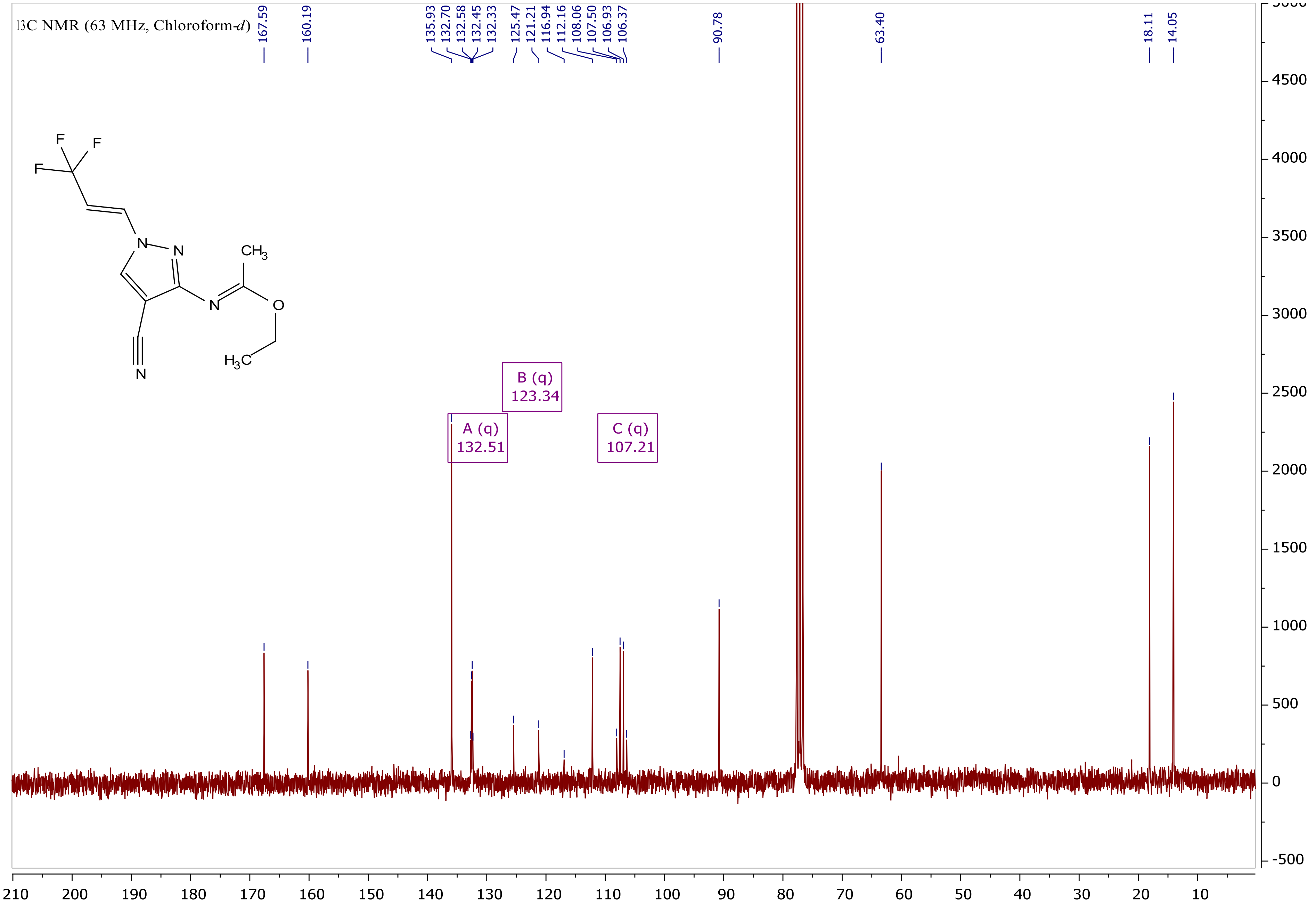

13C NMR (63 MHz, Chloroform-d)
167.59
160.19
135.93
132.70
132.58
132.45
132.33
125.47
121.21
116.94
112.16
108.06
107.50
106.93
106.37
90.78
63.40
18.11
14.05
A (q) 132.51
B (q) 123.34
C (q) 107.21
F
F
F
N
N
N
N
O
CH3
H3C
f1 (ppm)

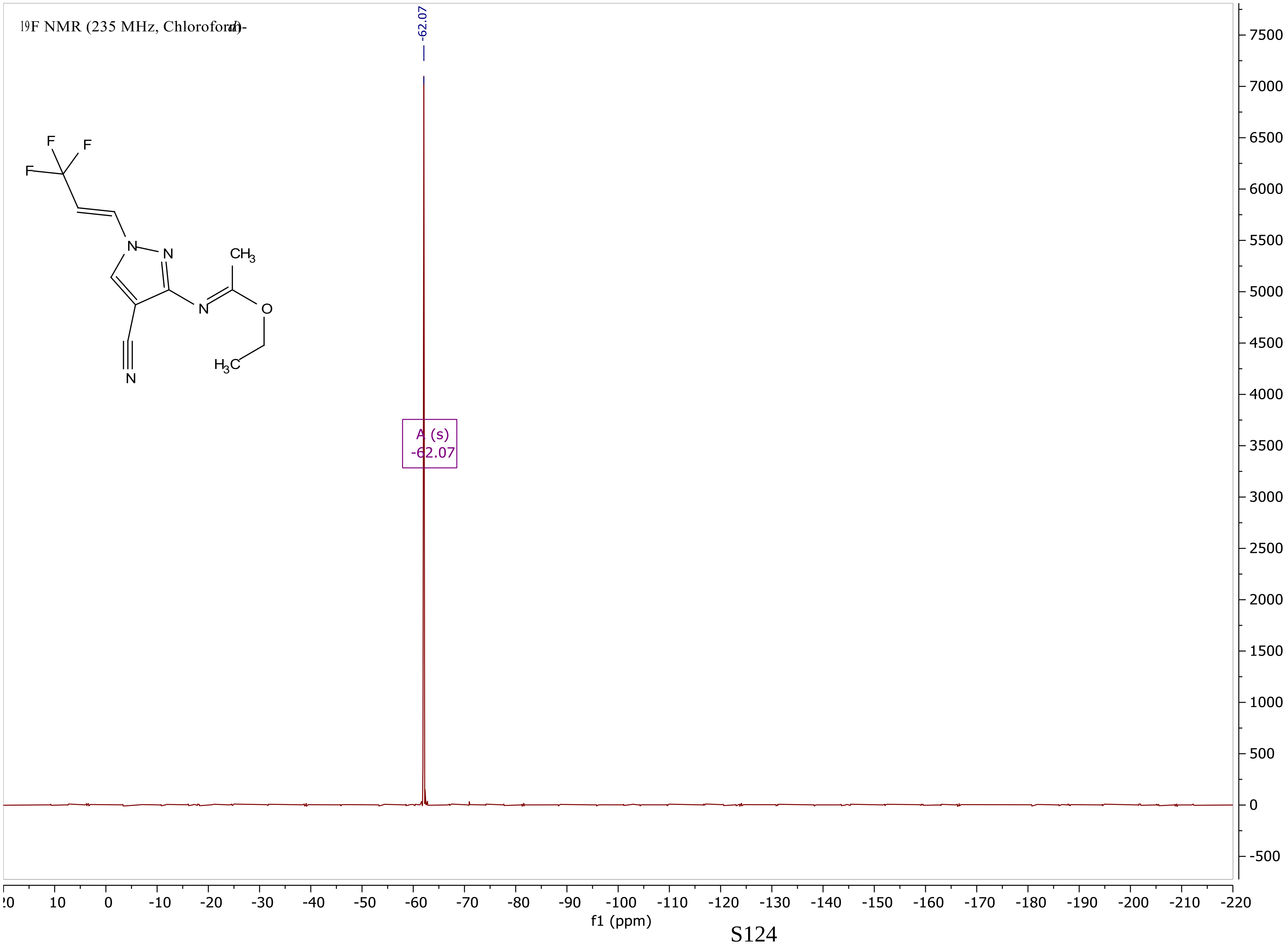
19F NMR (235 MHz, Chloroform-d)
-62.07
A (s)
-62.07
f1 (ppm)

(*E*)-4-Bromo-1-(3,3,3-trifluoroprop-1-en-1-yl)-1*H*-imidazole (24)

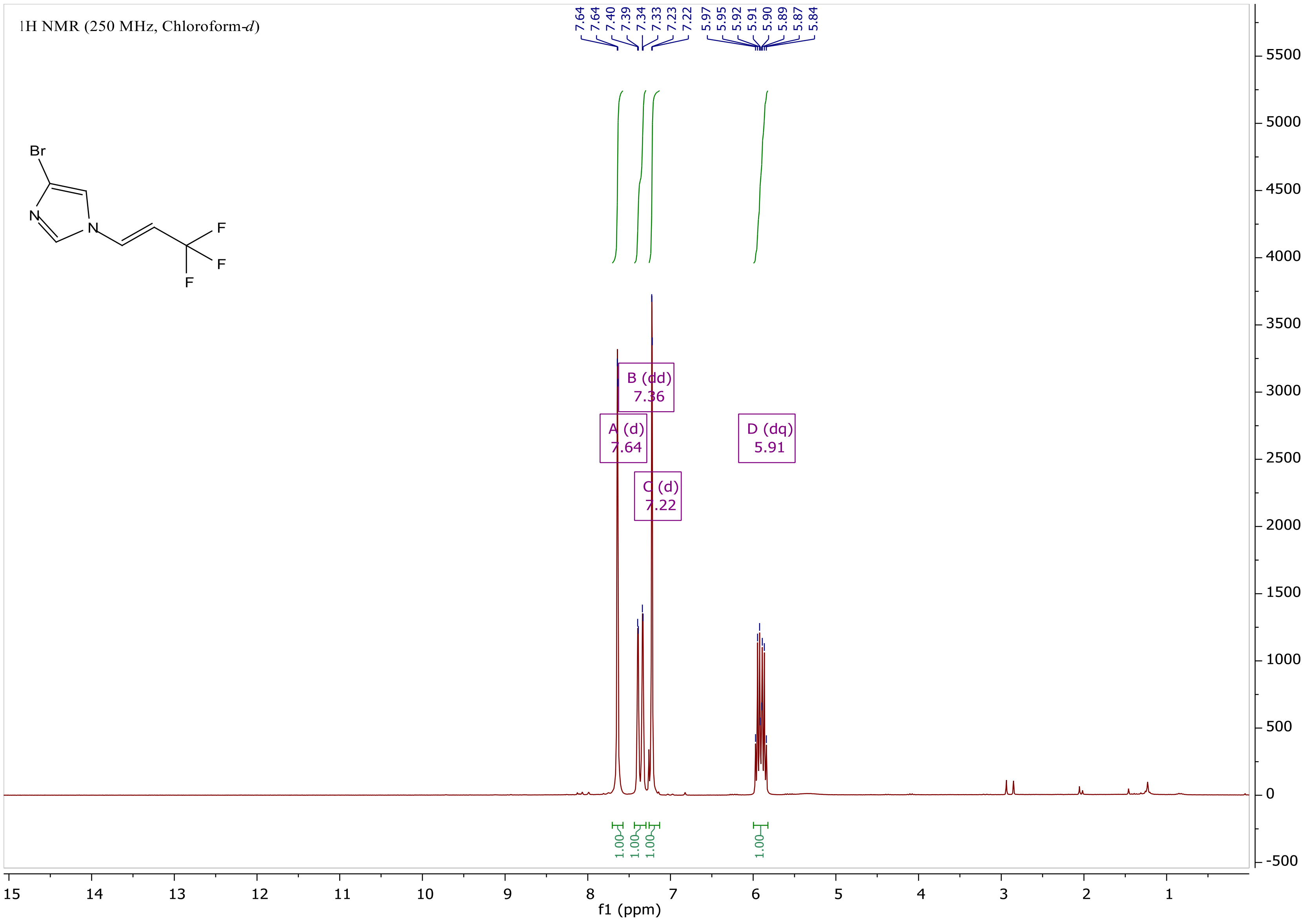

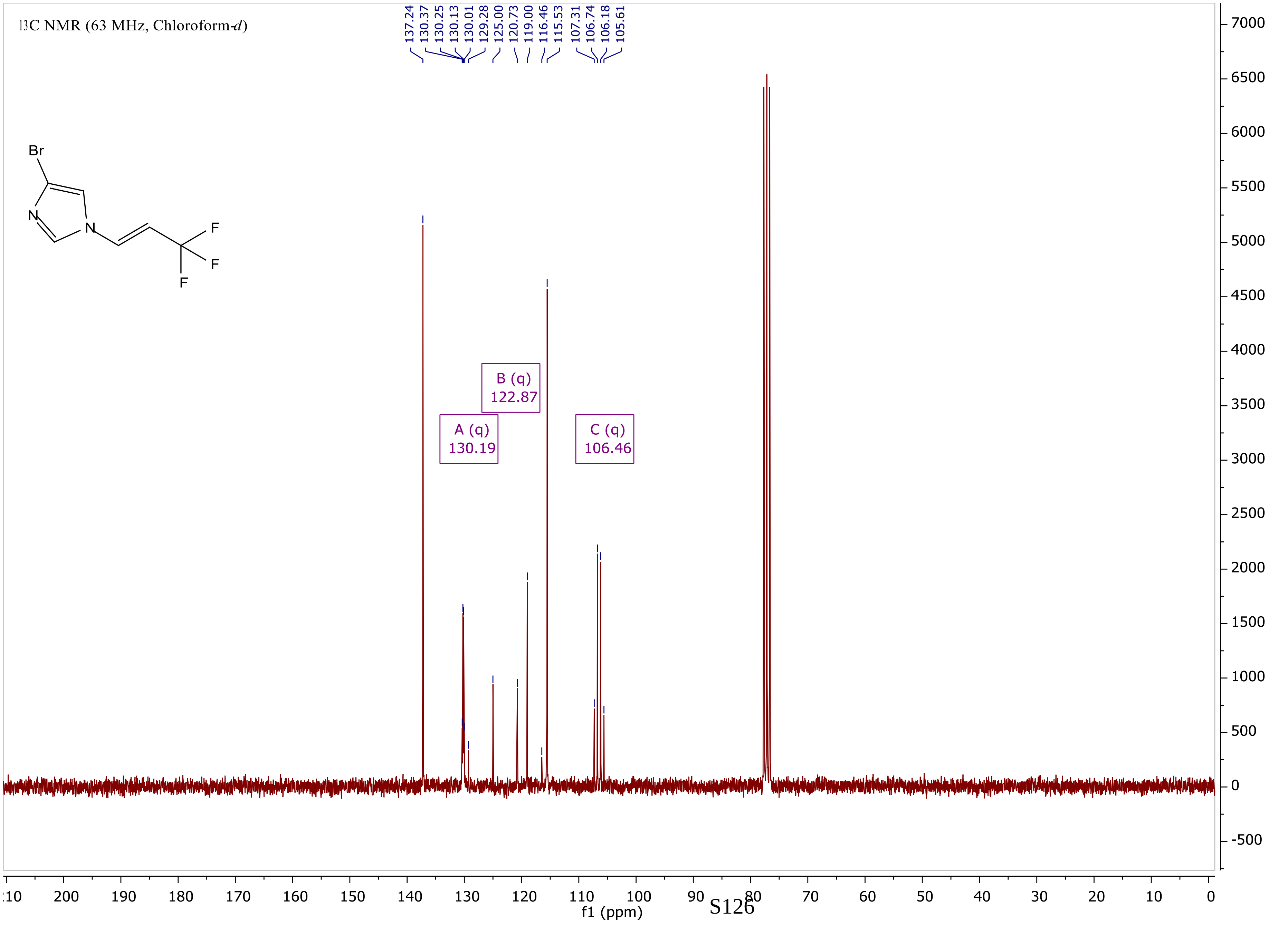
13C NMR (63 MHz, Chloroform-d)
137.24
130.37
130.25
130.13
130.01
129.28
125.00
120.73
119.00
116.46
115.53
107.31
106.74
106.18
105.61
Br
N
N
F
F
F
A (q)
130.19
B (q)
122.87
C (q)
106.46
f1 (ppm)

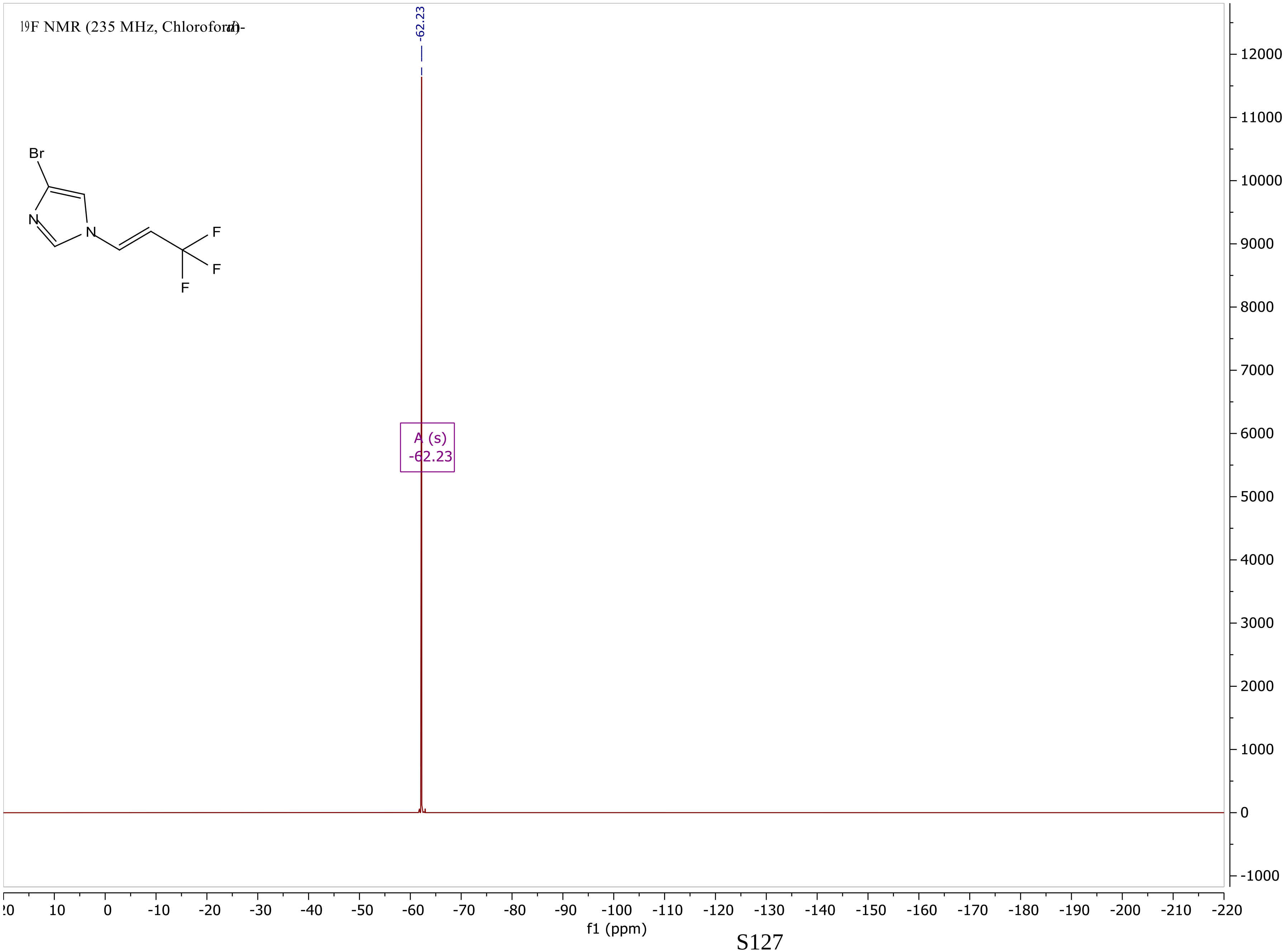
19F NMR (235 MHz, Chloroform-d)
-62.23
A (s)
-62.23
Br
N
N
F
F
F
f1 (ppm)

(*E*)-2-Ethyl-1-(3,3,3-trifluoroprop-1-en-1-yl)-1*H*-imidazole (25)

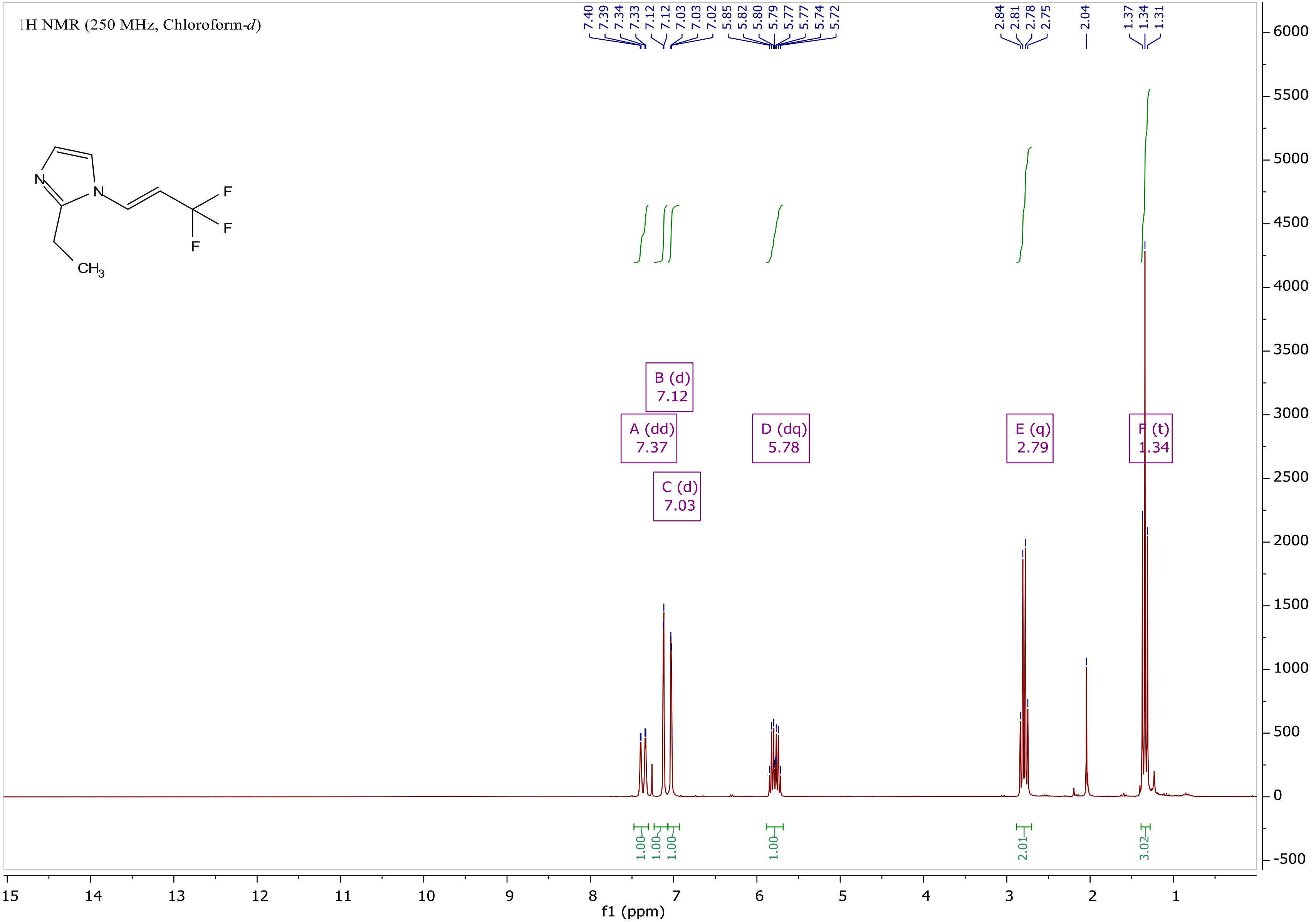

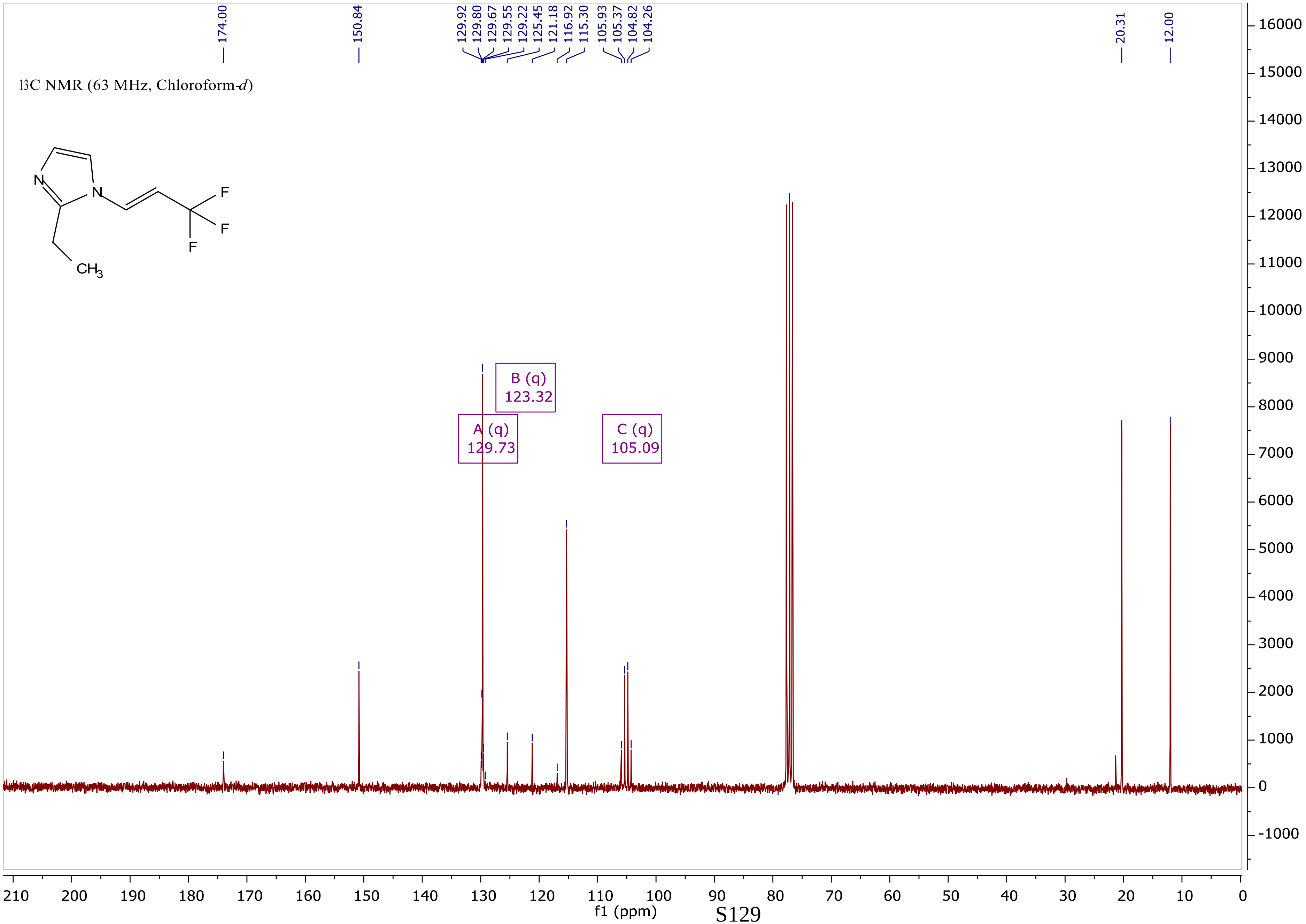
13C NMR (63 MHz, Chloroform-d)
174.00
150.84
129.92
129.80
129.67
129.55
129.22
125.45
121.18
116.92
115.30
105.93
105.37
104.82
104.26
20.31
12.00
A (q) 129.73
B (q) 123.32
C (q) 105.09
N
N
F
F
F
CH3
f1 (ppm)

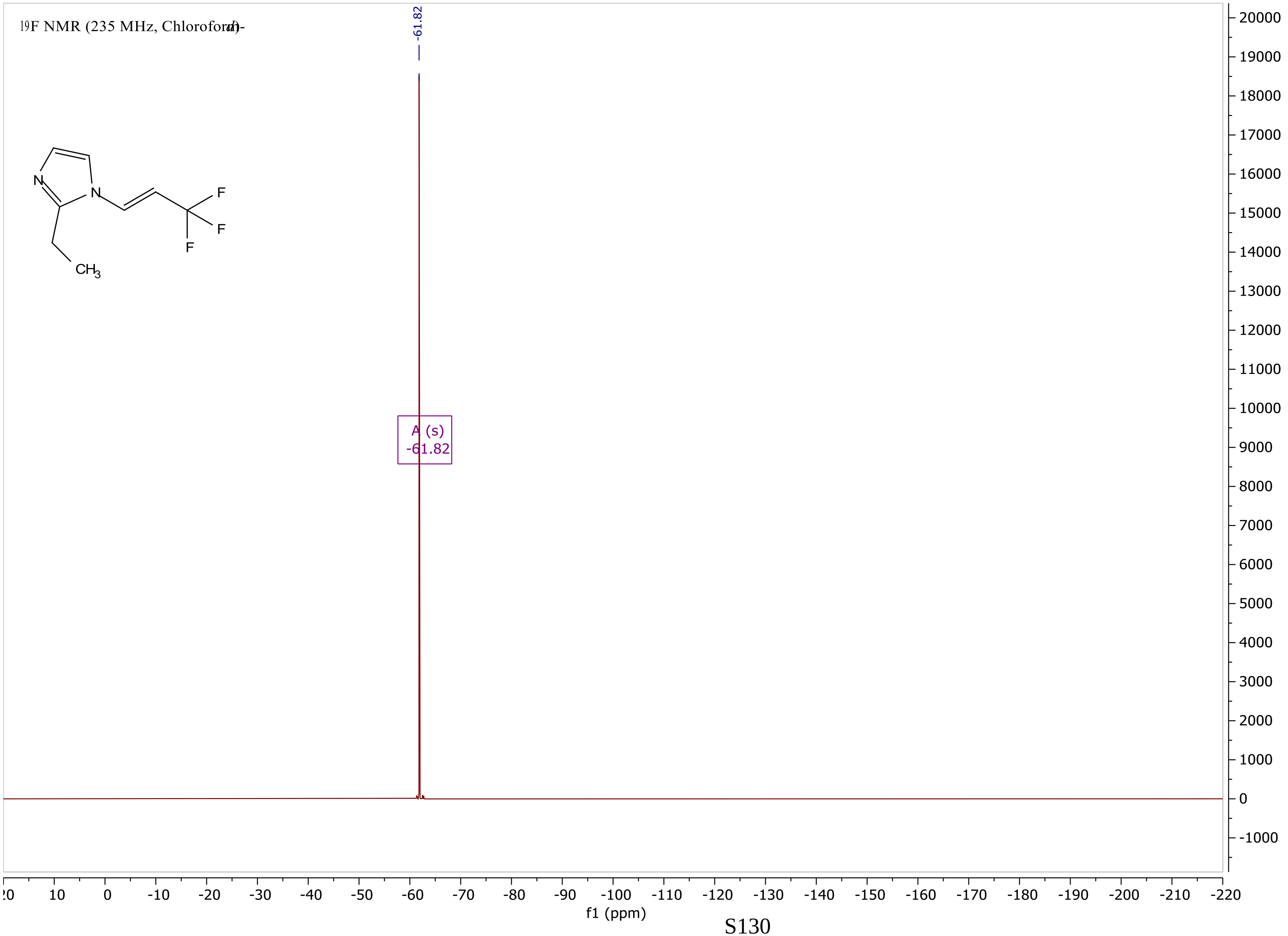

19F NMR (235 MHz, Chloroform-d)
-61.82
A (s)
-61.82
N
N
F
F
F
CH3
f1 (ppm)

(*E*)-4,5-Diphenyl-1-(3,3,3-trifluoroprop-1-en-1-yl)-1*H*-imidazole (26)

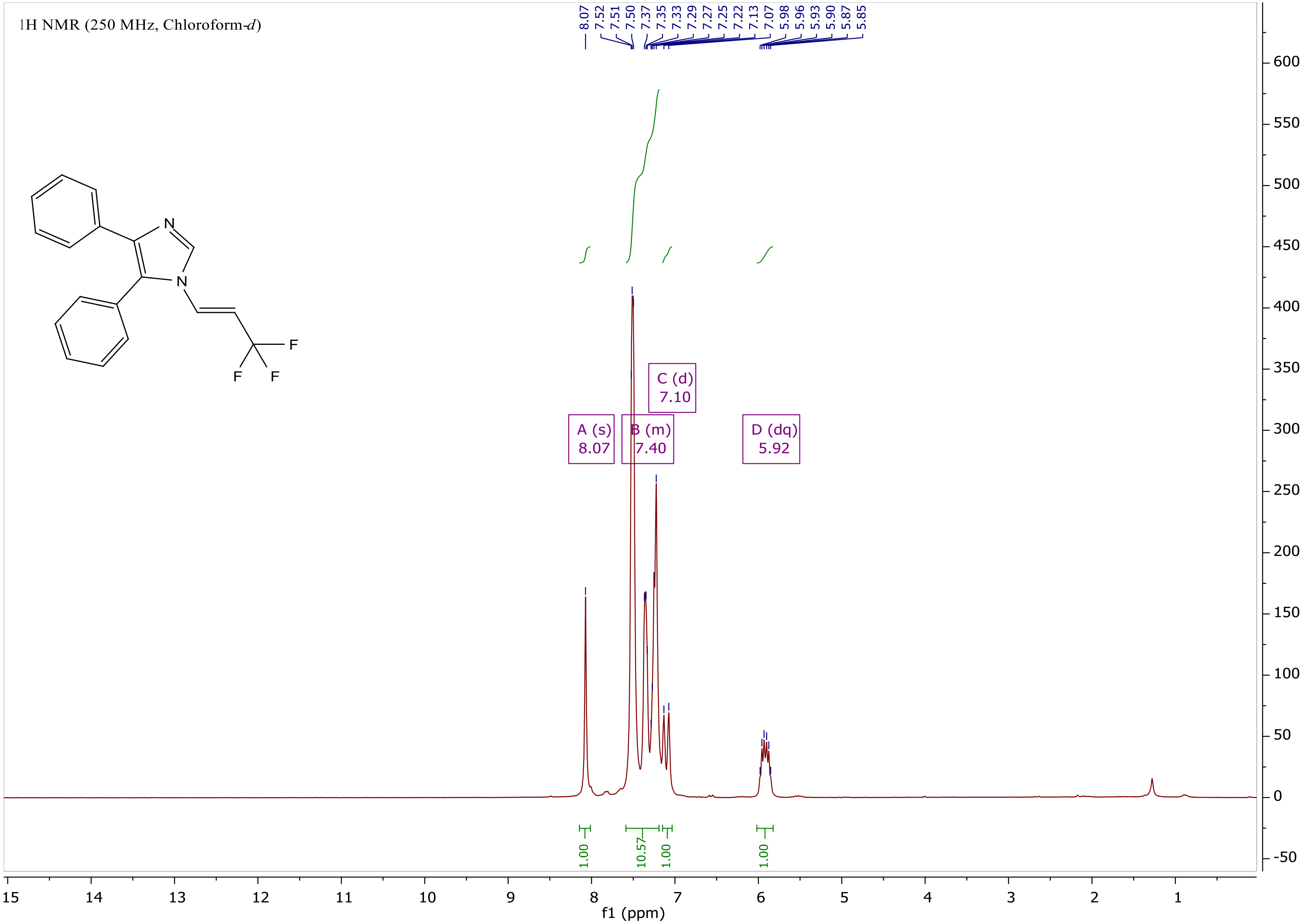

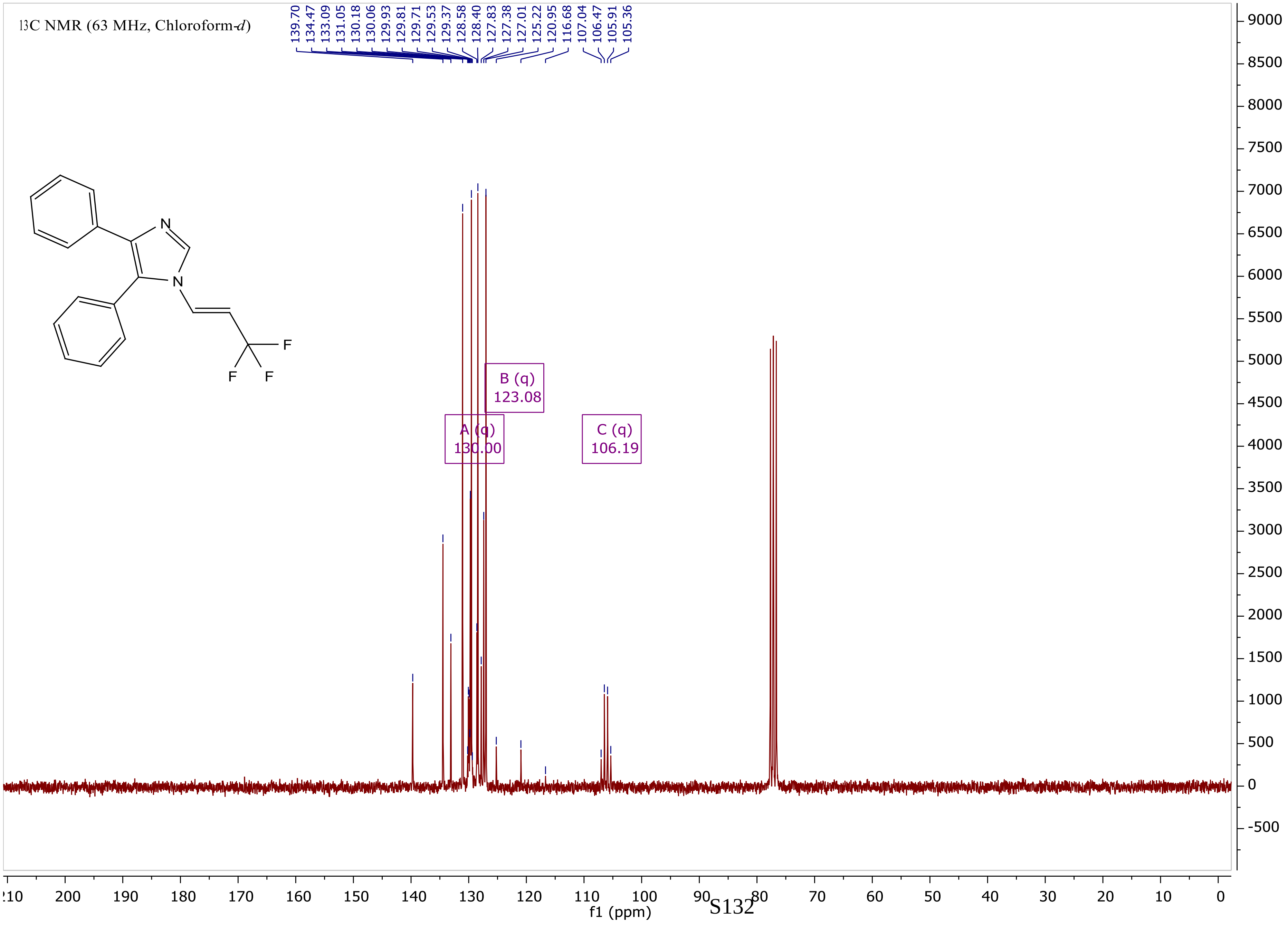
13C NMR (63 MHz, Chloroform-d)
139.70
134.47
133.09
131.05
130.18
130.06
129.93
129.81
129.71
129.53
129.37
128.58
128.40
127.83
127.38
127.01
125.22
120.95
116.68
107.04
106.47
105.91
105.36
B (q)
123.08
A (q)
130.00
C (q)
106.19
N
N
F
F
F
f1 (ppm)

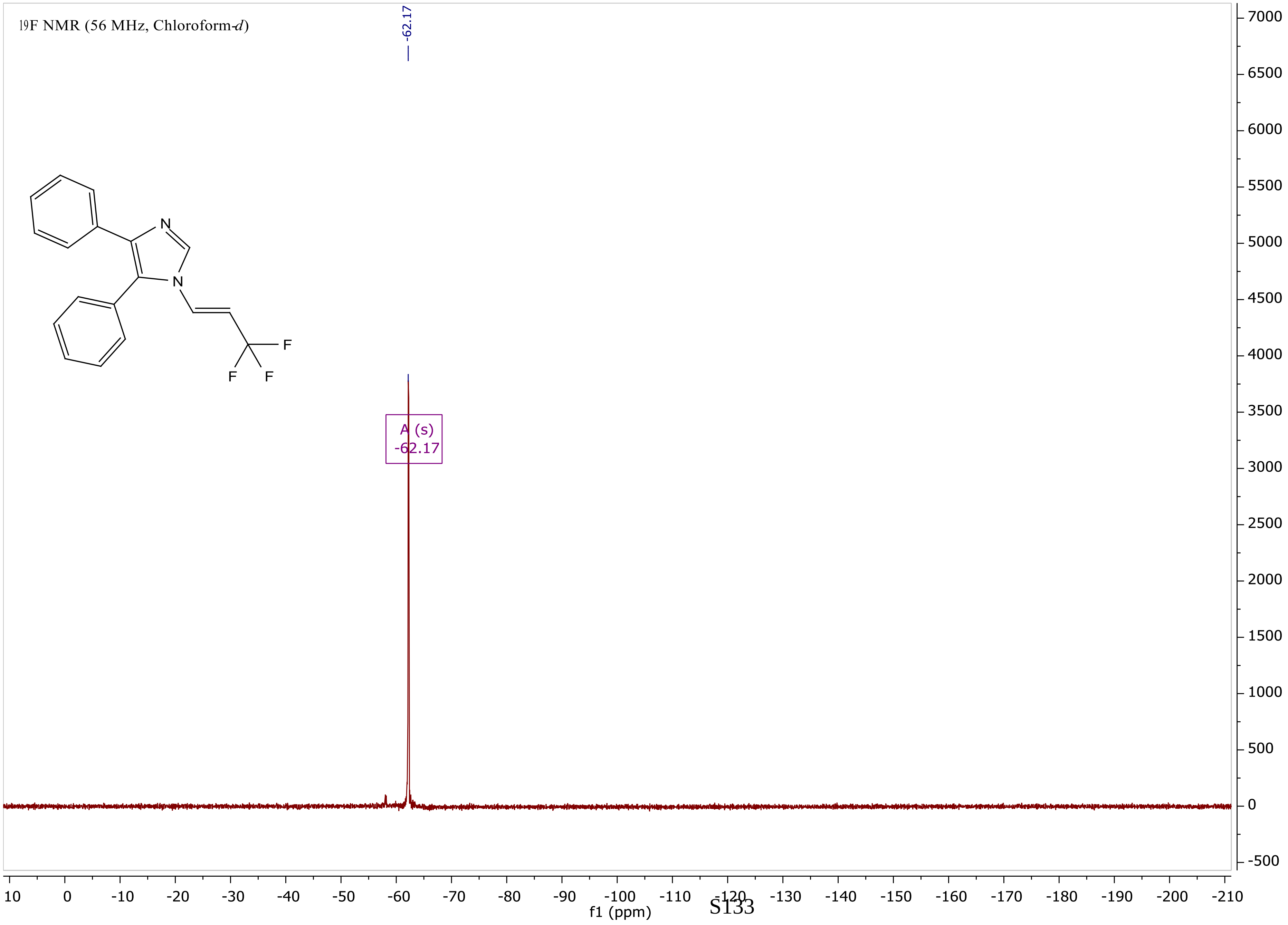
19F NMR (56 MHz, Chloroform-d)
-62.17
A (s)
-62.17
N
N
F
F
F
f1 (ppm)

Ethyl (*E*)-1-(3,3,3-trifluoroprop-1-en-1-yl)-1*H*-imidazole-4-carboxylate (27)

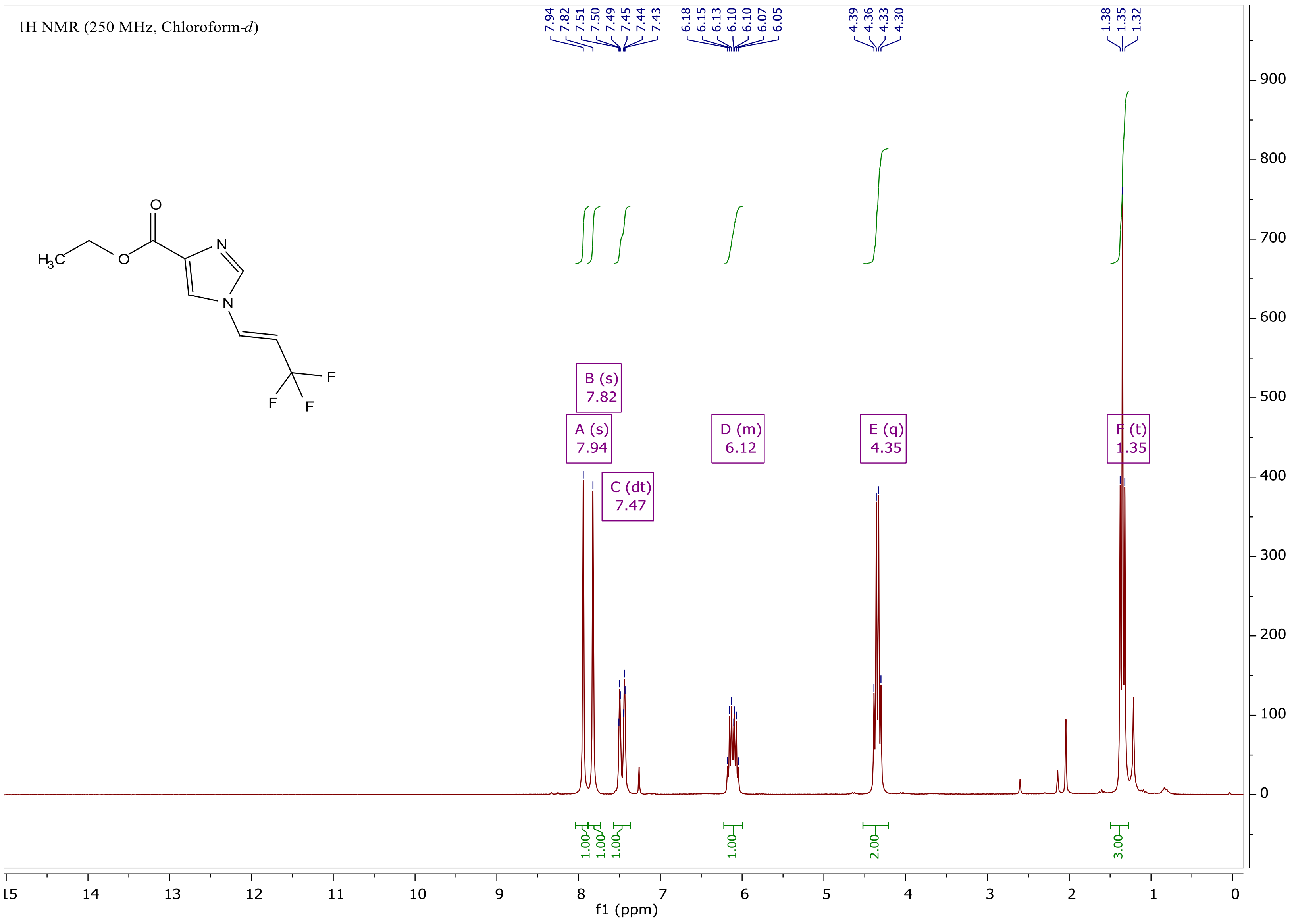

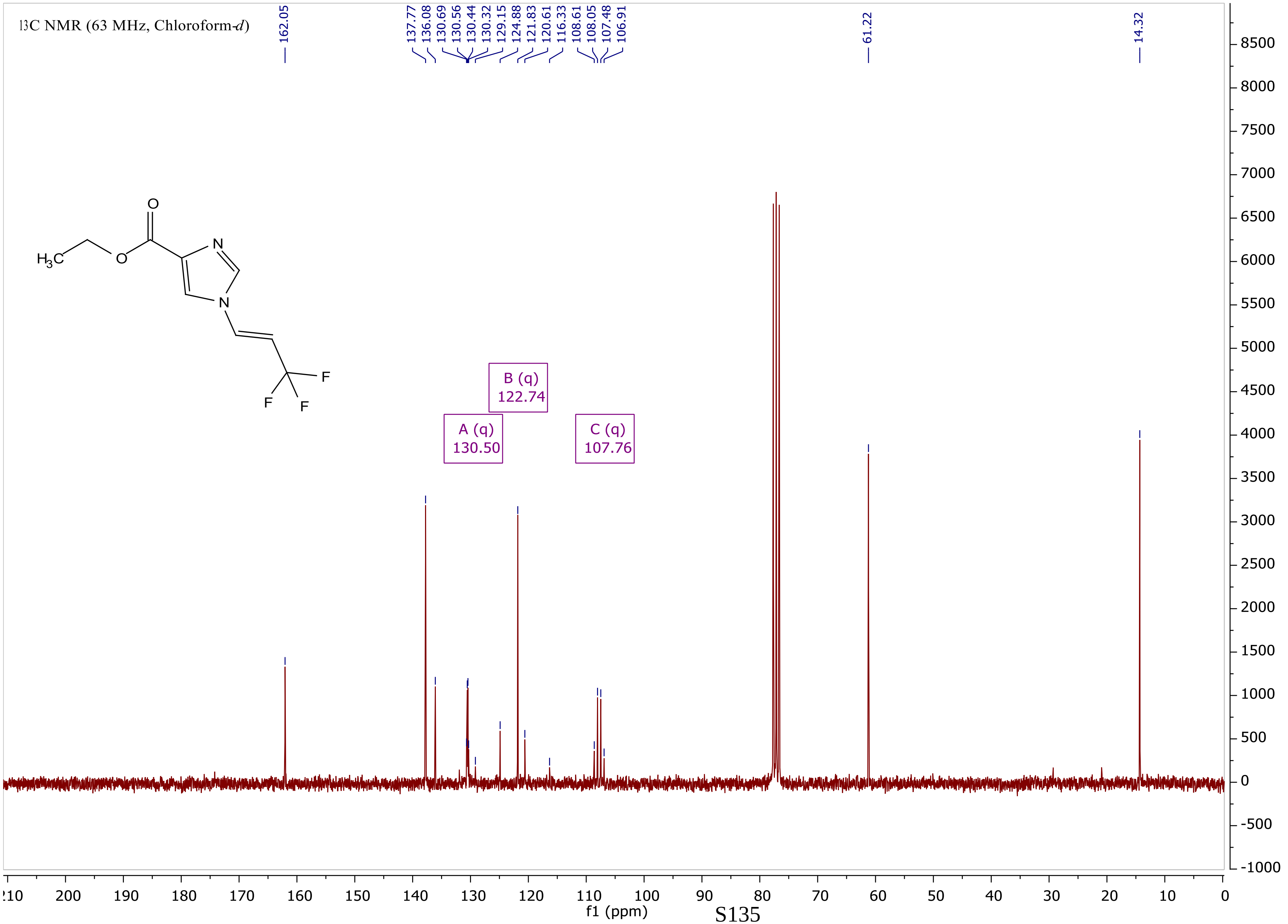
13C NMR (63 MHz, Chloroform-d)
162.05
137.77
136.08
130.69
130.56
130.44
130.32
129.15
124.88
121.83
120.61
116.33
108.61
108.05
107.48
106.91
61.22
14.32
B (q)
122.74
A (q)
130.50
C (q)
107.76
O
N
N
O
H3C
F
F
F
f1 (ppm)

19F NMR (56 MHz, Chloroform-*d*)

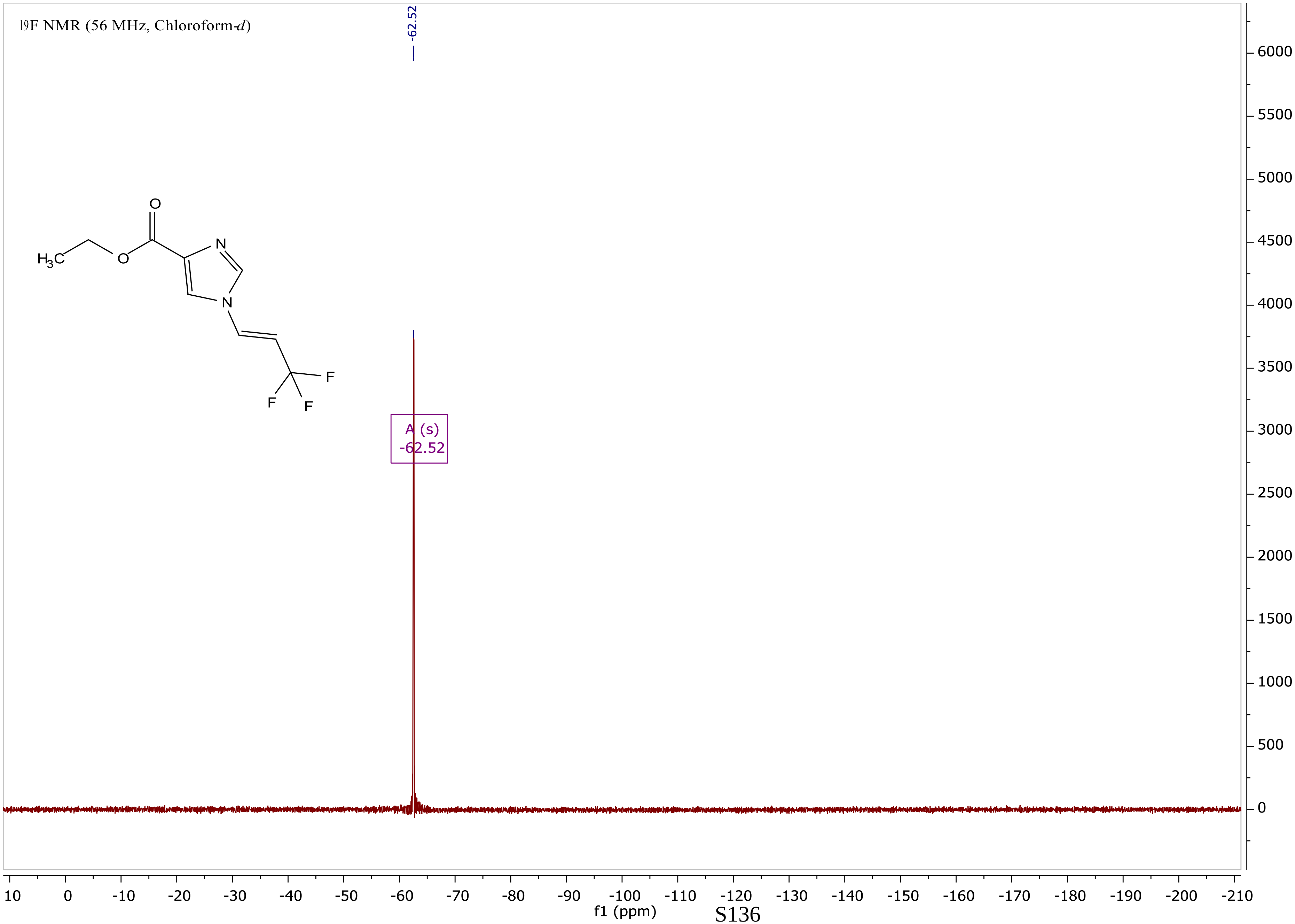

(*E*)-5-Phenyl-1-(3,3,3-trifluoroprop-1-en-1-yl)-1*H*-tetrazole (28)

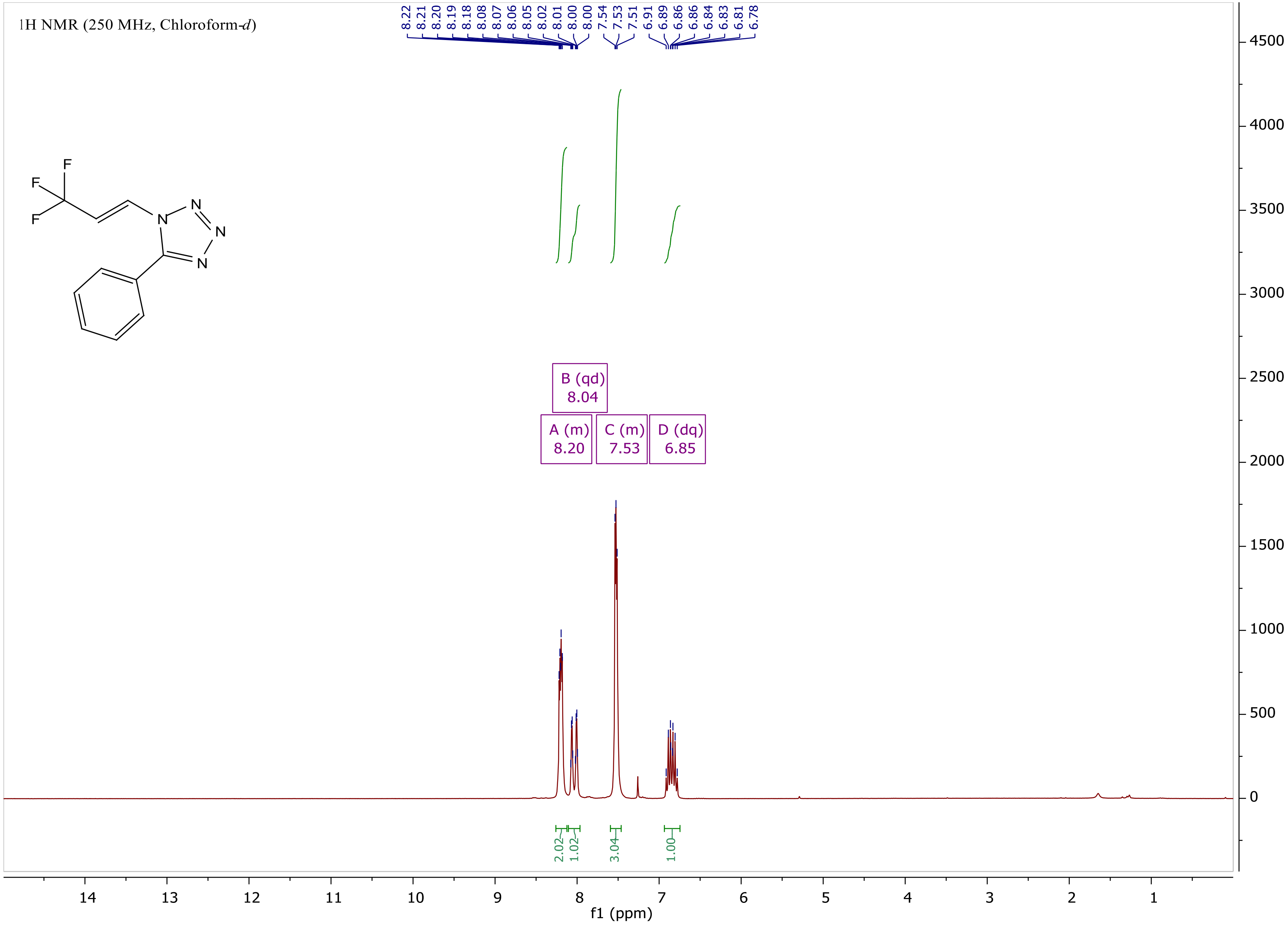

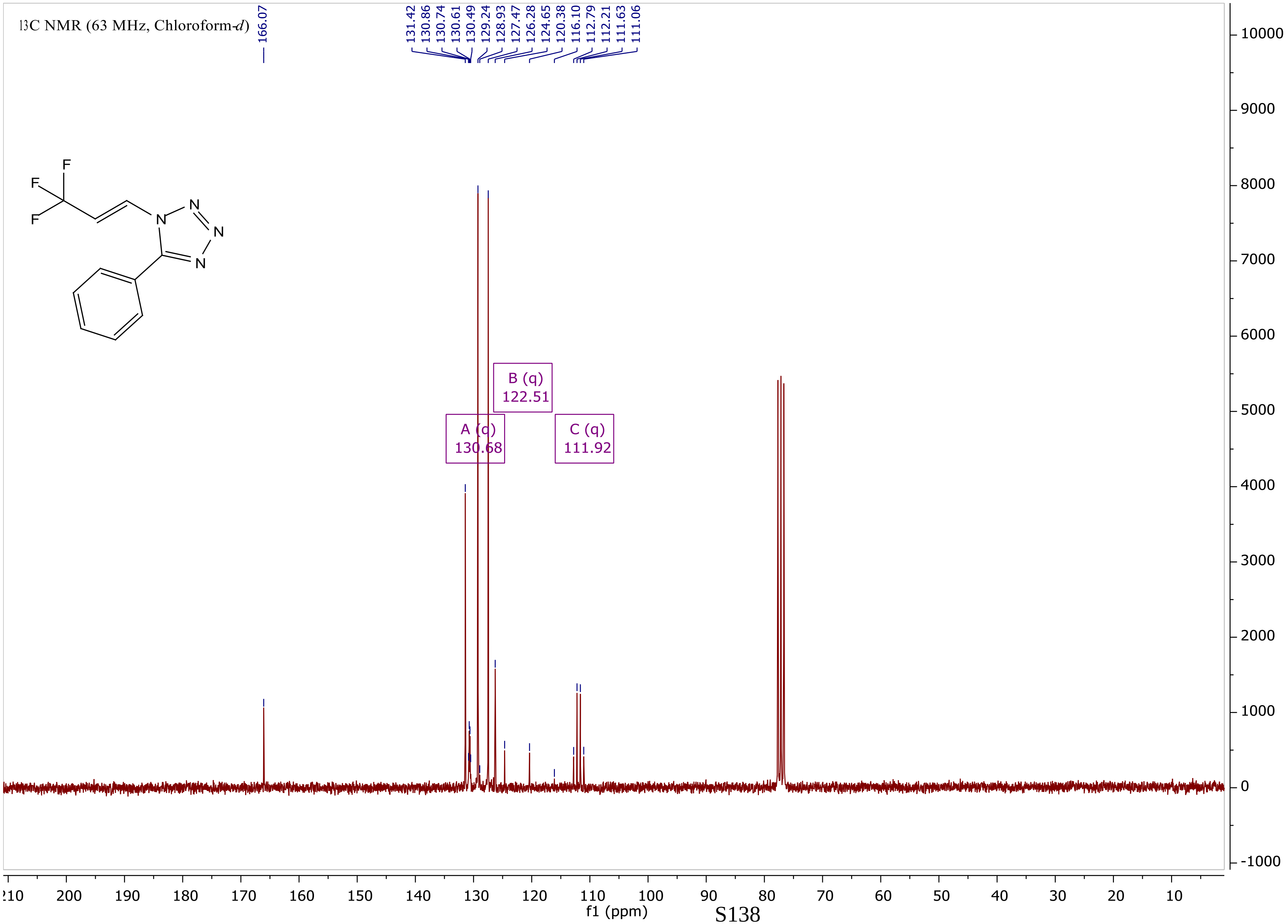
13C NMR (63 MHz, Chloroform-d)
166.07
131.42
130.86
130.74
130.61
130.49
129.24
128.93
127.47
126.28
124.65
120.38
116.10
112.79
112.21
111.63
111.06
A (q)
130.68
B (q)
122.51
C (q)
111.92
f1 (ppm)

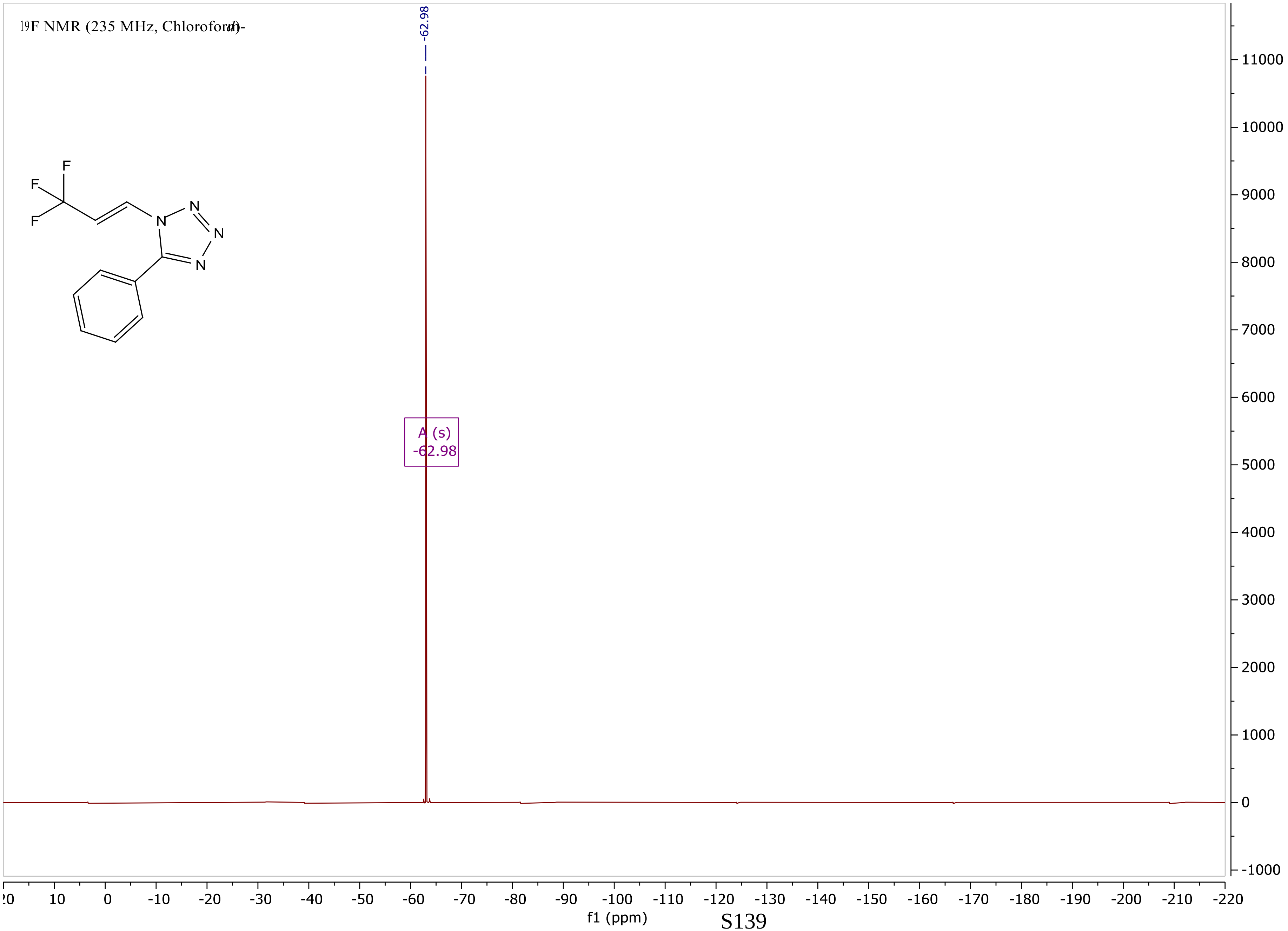
19F NMR (235 MHz, Chloroform-d)
-62.98
A (s)
-62.98
f1 (ppm)

(*E*)-2-(3,3,3-Trifluoroprop-1-en-1-yl)-2*H*-indazole (29)

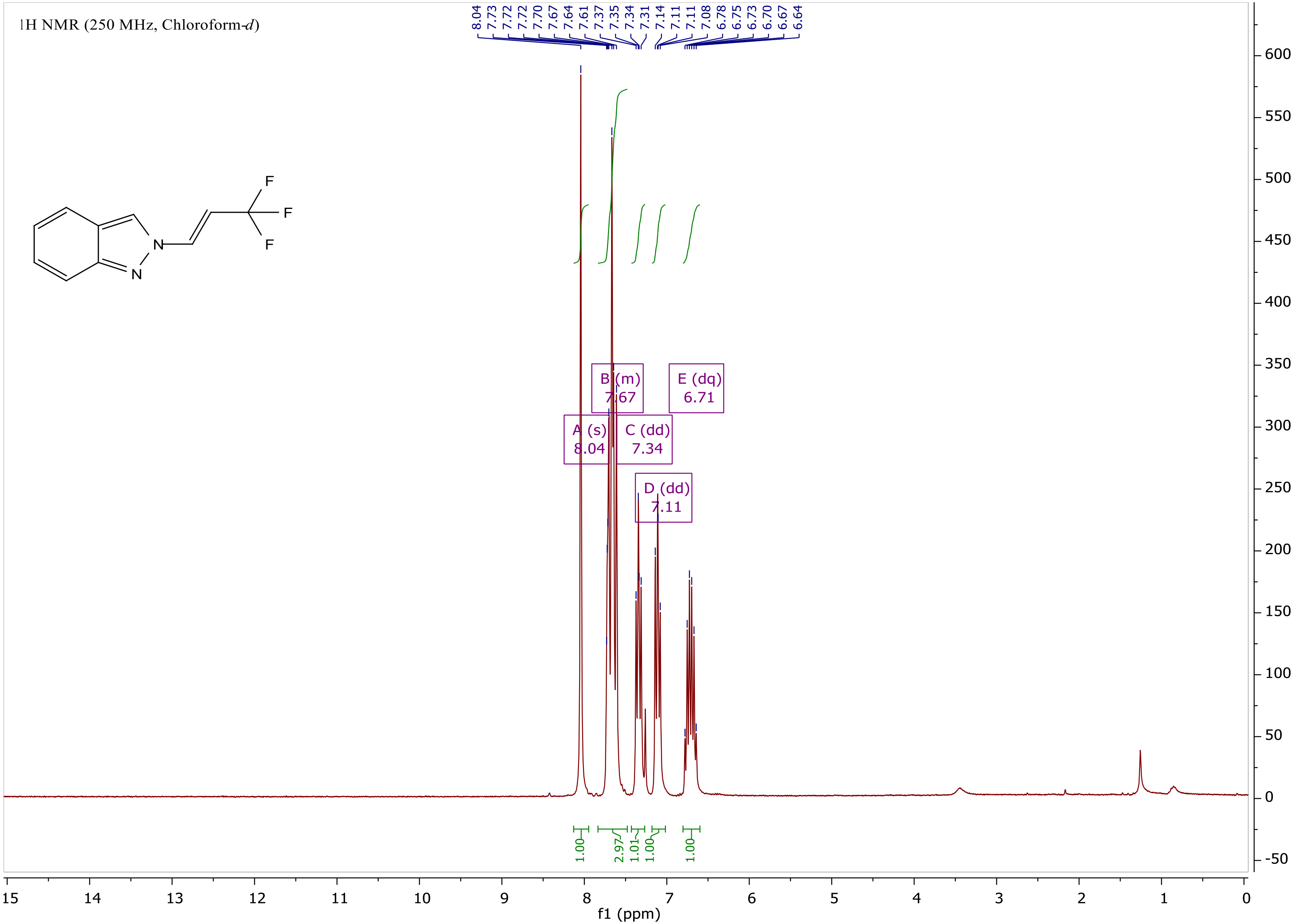

13C NMR (63 MHz, Chloroform-*d*)

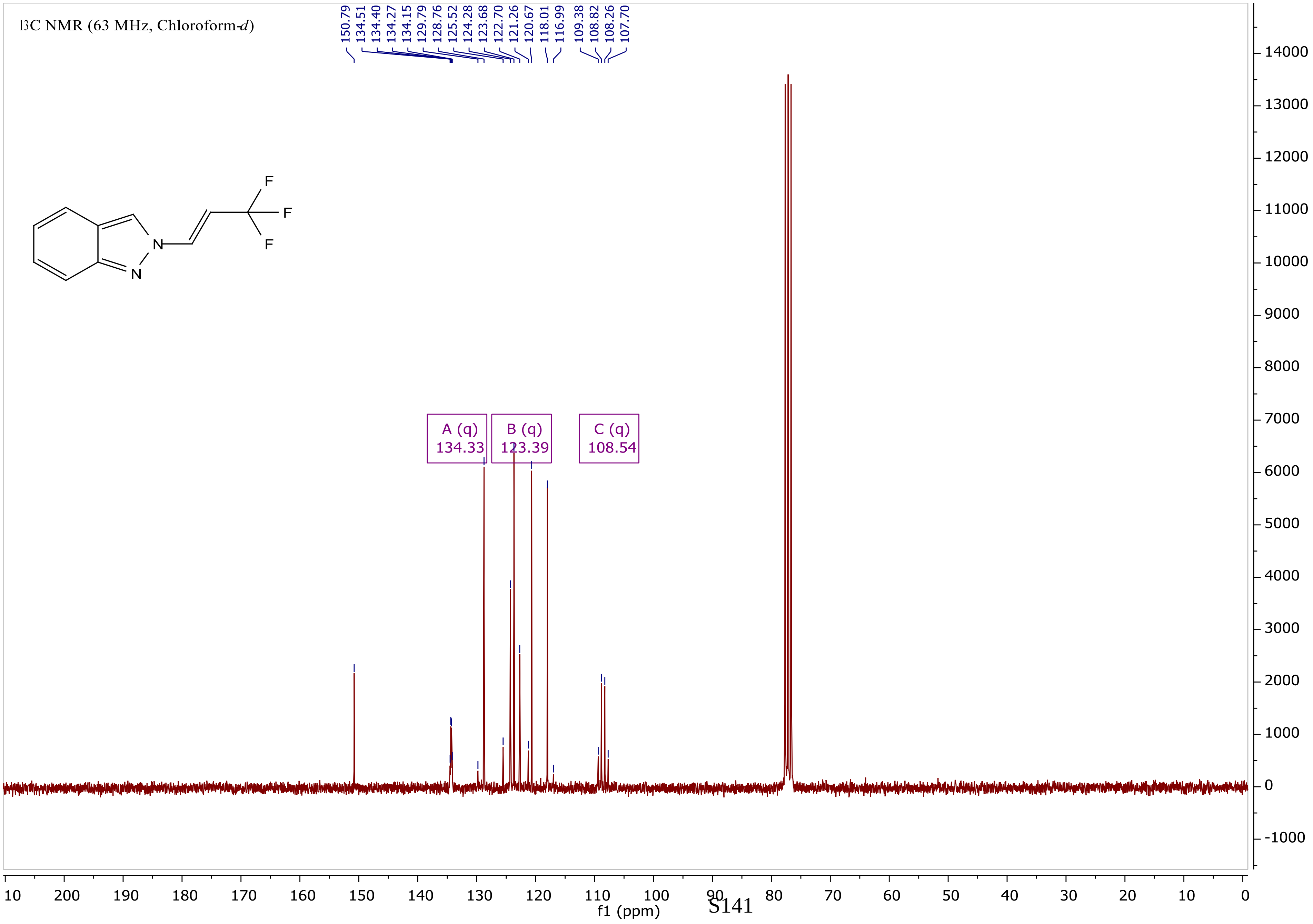

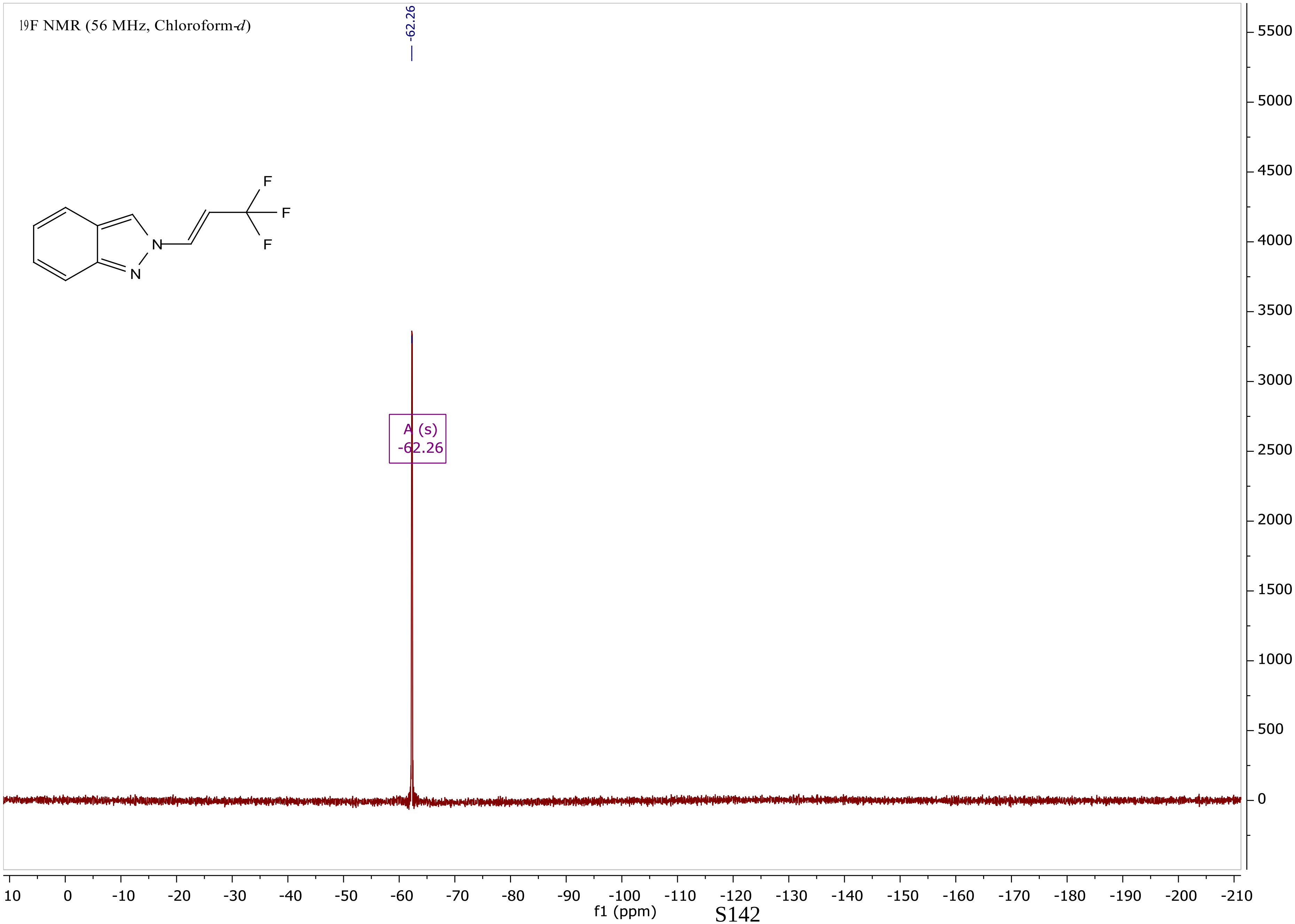
19F NMR (56 MHz, Chloroform-d)
-62.26
A (s)
-62.26
F
F
F
N
N
f1 (ppm)

Ethyl (*E*)-2-(3,3,3-trifluoroprop-1-en-1-yl)-2*H*-indazole-3-carboxylate (30) izomer1

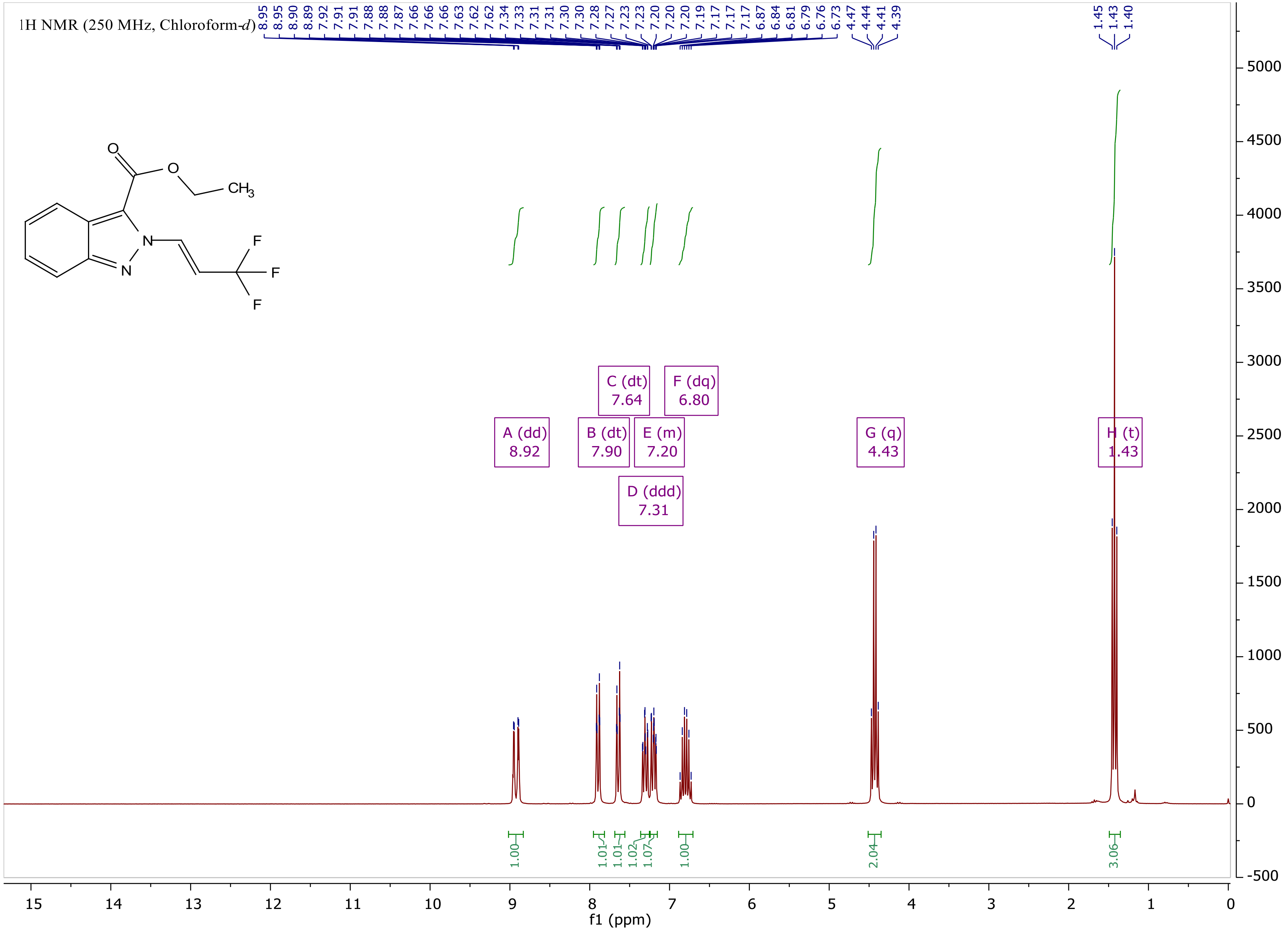

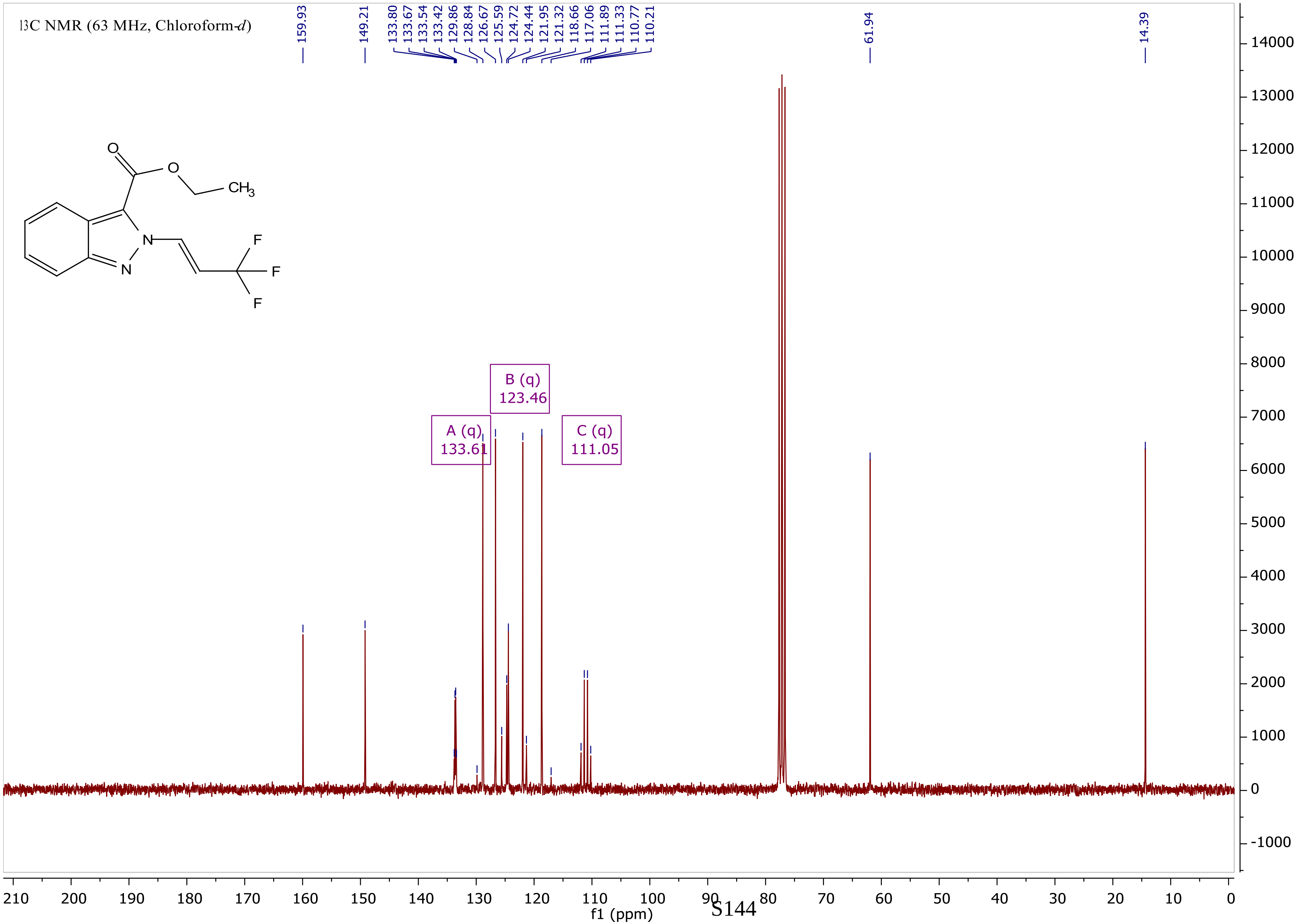
13C NMR (63 MHz, Chloroform-d)
159.93
149.21
133.80
133.67
133.54
133.42
129.86
128.84
126.67
125.59
124.72
124.44
121.95
121.32
118.66
117.06
111.89
111.33
110.77
110.21
61.94
14.39
A (q)
133.61
B (q)
123.46
C (q)
111.05
f1 (ppm)

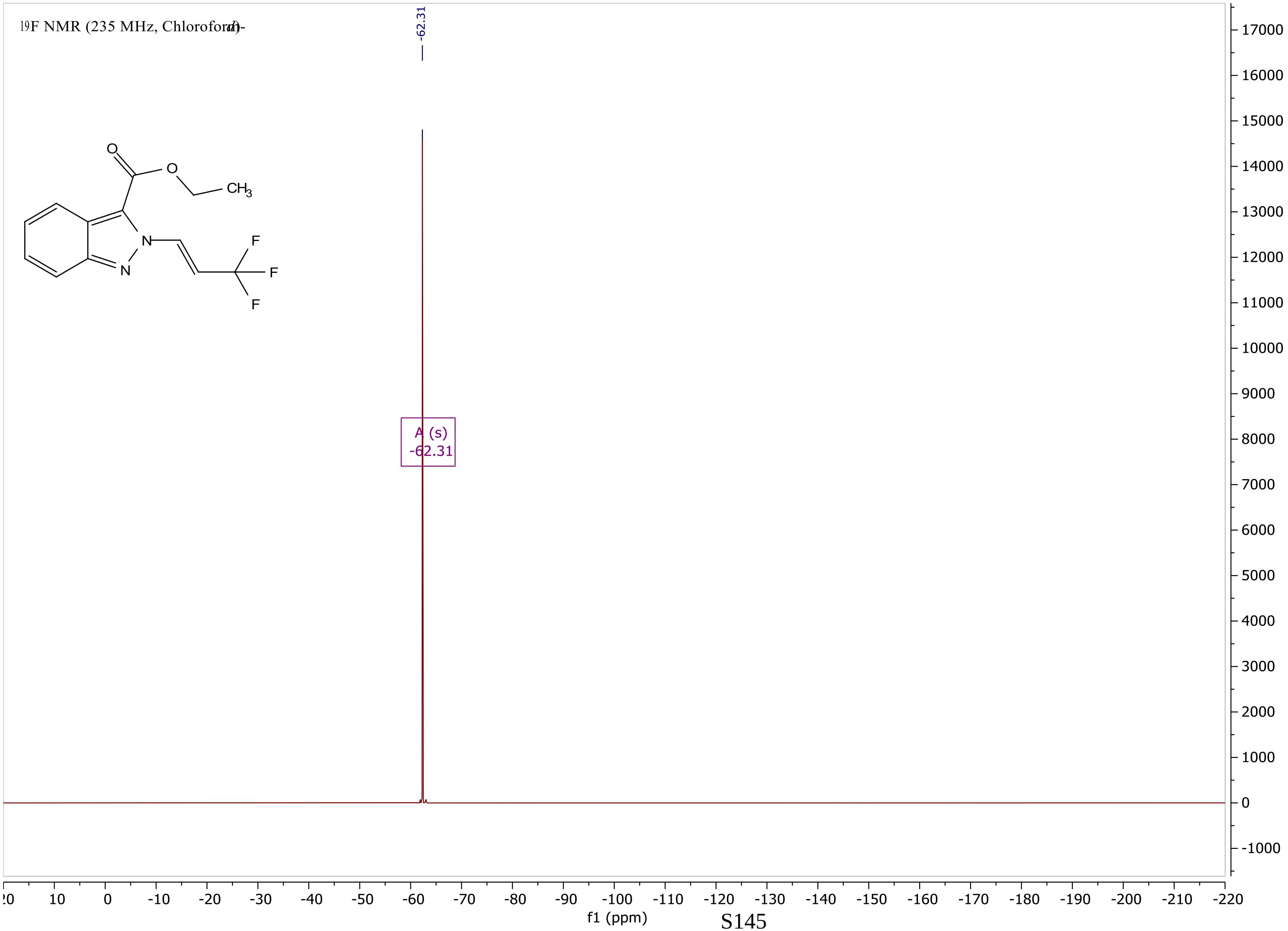
19F NMR (235 MHz, Chloroform-d)
-62.31
A (s)
-62.31
17000
16000
15000
14000
13000
12000
11000
10000
9000
8000
7000
6000
5000
4000
3000
2000
1000
0
-1000
20
10
0
-10
-20
-30
-40
-50
-60
-70
-80
-90
-100
-110
-120
-130
-140
-150
-160
-170
-180
-190
-200
-210
-220
f1 (ppm)

Ethyl (*E*)-1-(3,3,3-trifluoroprop-1-en-1-yl)-1*H*-indazole-3-carboxylate (31) izomer2

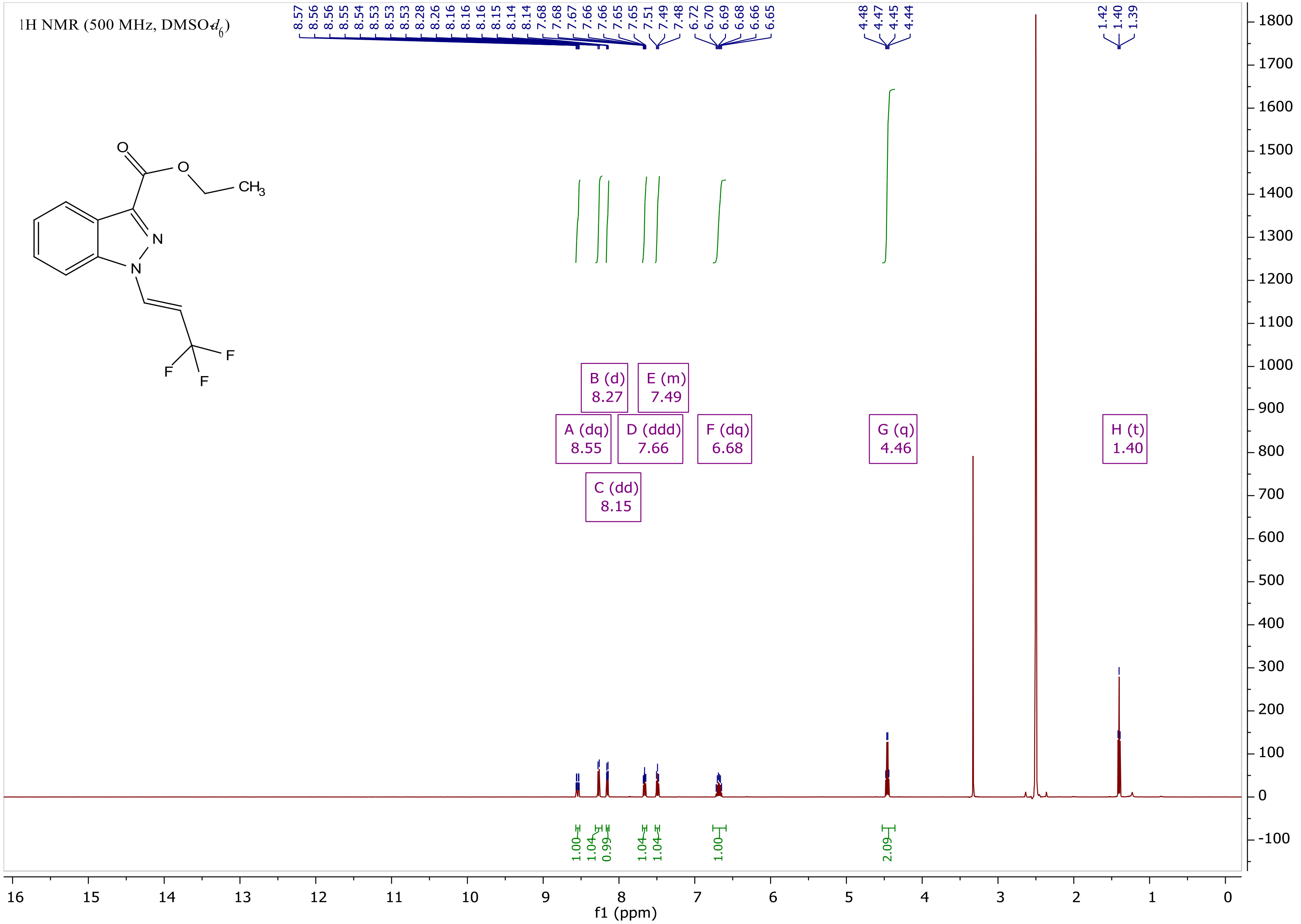

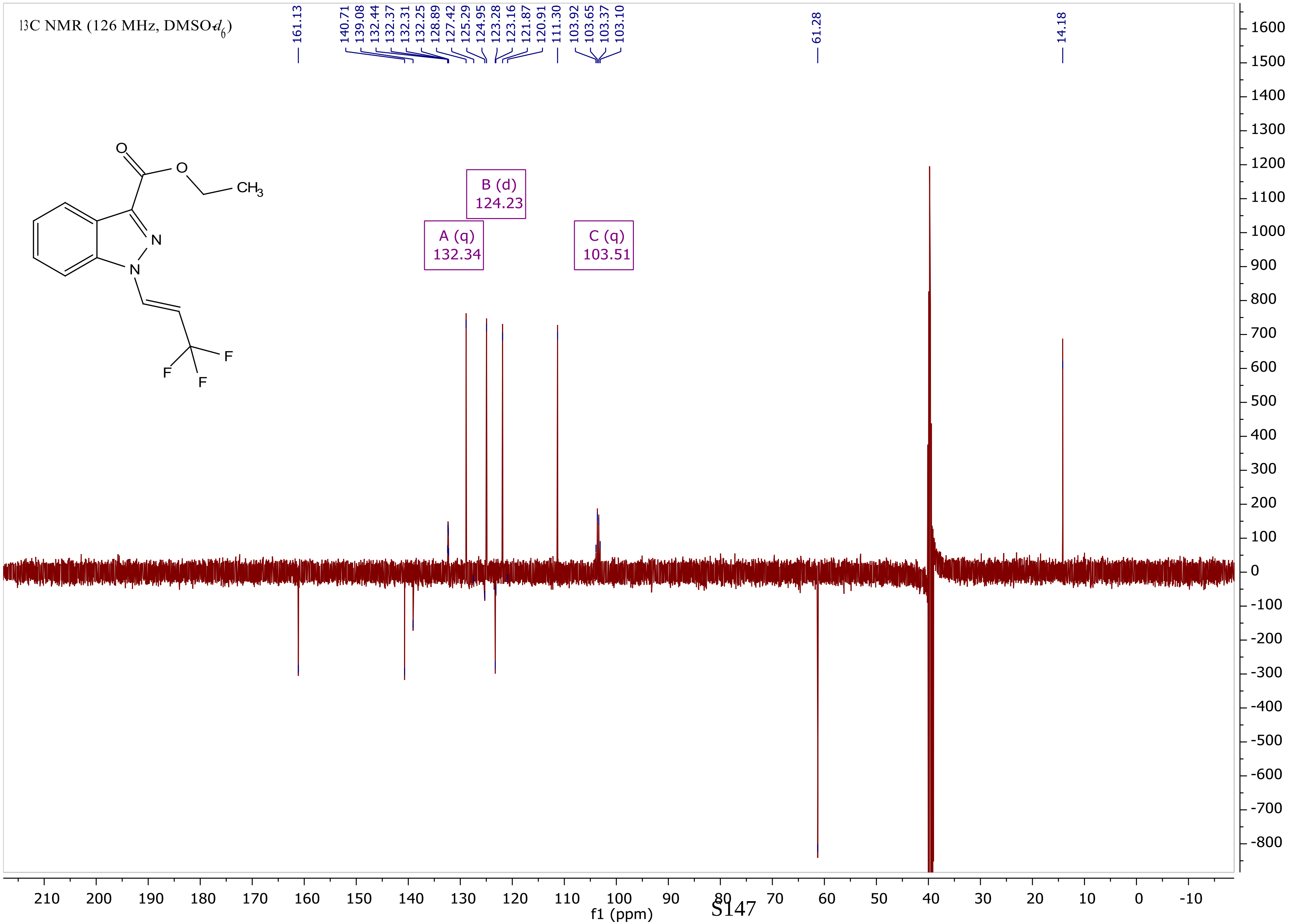
13C NMR (126 MHz, DMSO-d6)
161.13
140.71
139.08
132.44
132.37
132.31
132.25
128.89
127.42
125.29
124.95
123.28
123.16
121.87
120.91
111.30
103.92
103.65
103.37
103.10
61.28
14.18
A (q)
132.34
B (d)
124.23
C (q)
103.51
O
O
CH3
N
N
F
F
F
f1 (ppm)

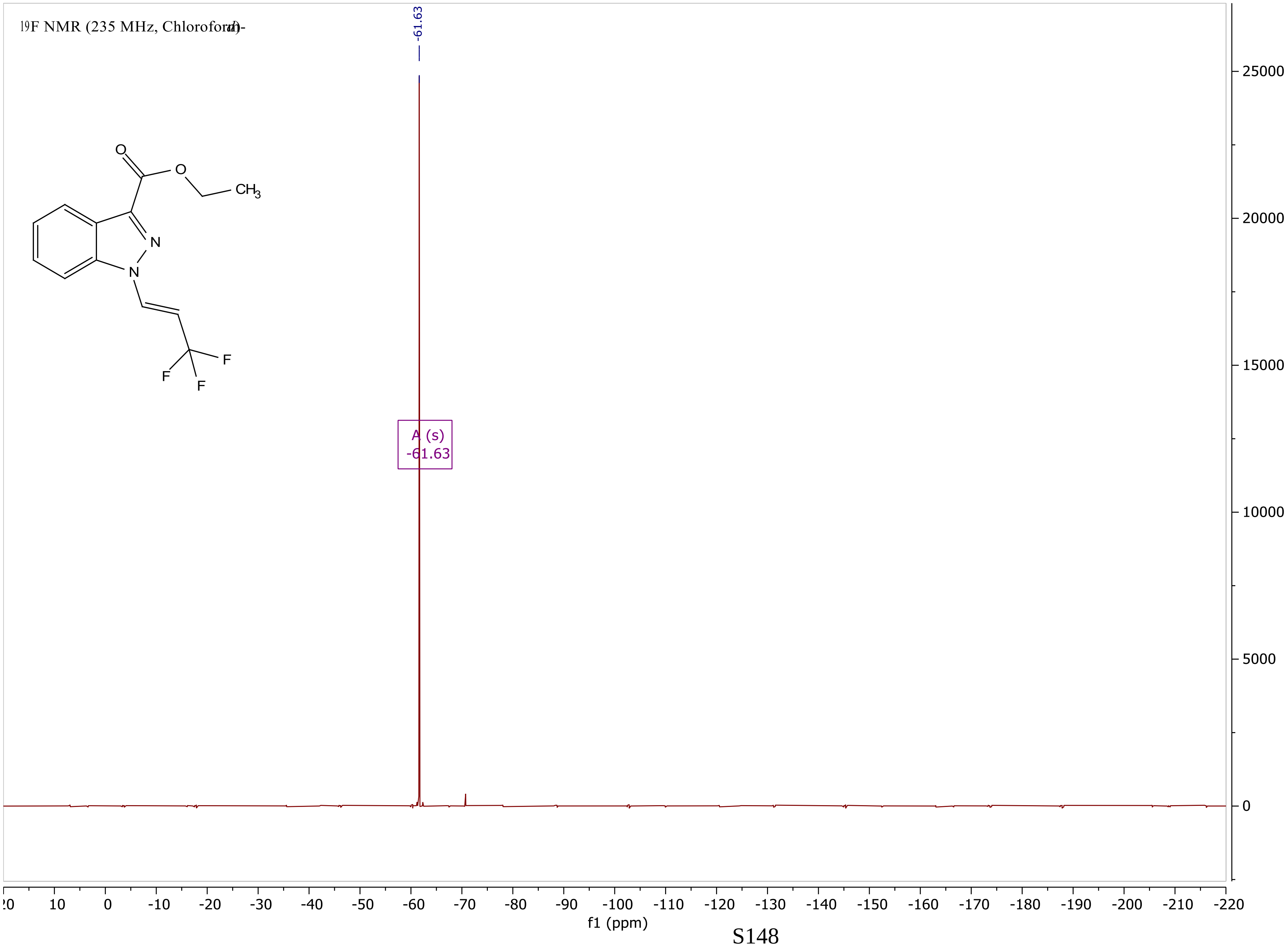
19F NMR (235 MHz, Chloroform-d)
-61.63
A (s)
-61.63
O
O
CH3
N
N
F
F
F
f1 (ppm)

(*E*)-6-Bromo-1-(3,3,3-trifluoroprop-1-en-1-yl)-1*H*-indazole (37)

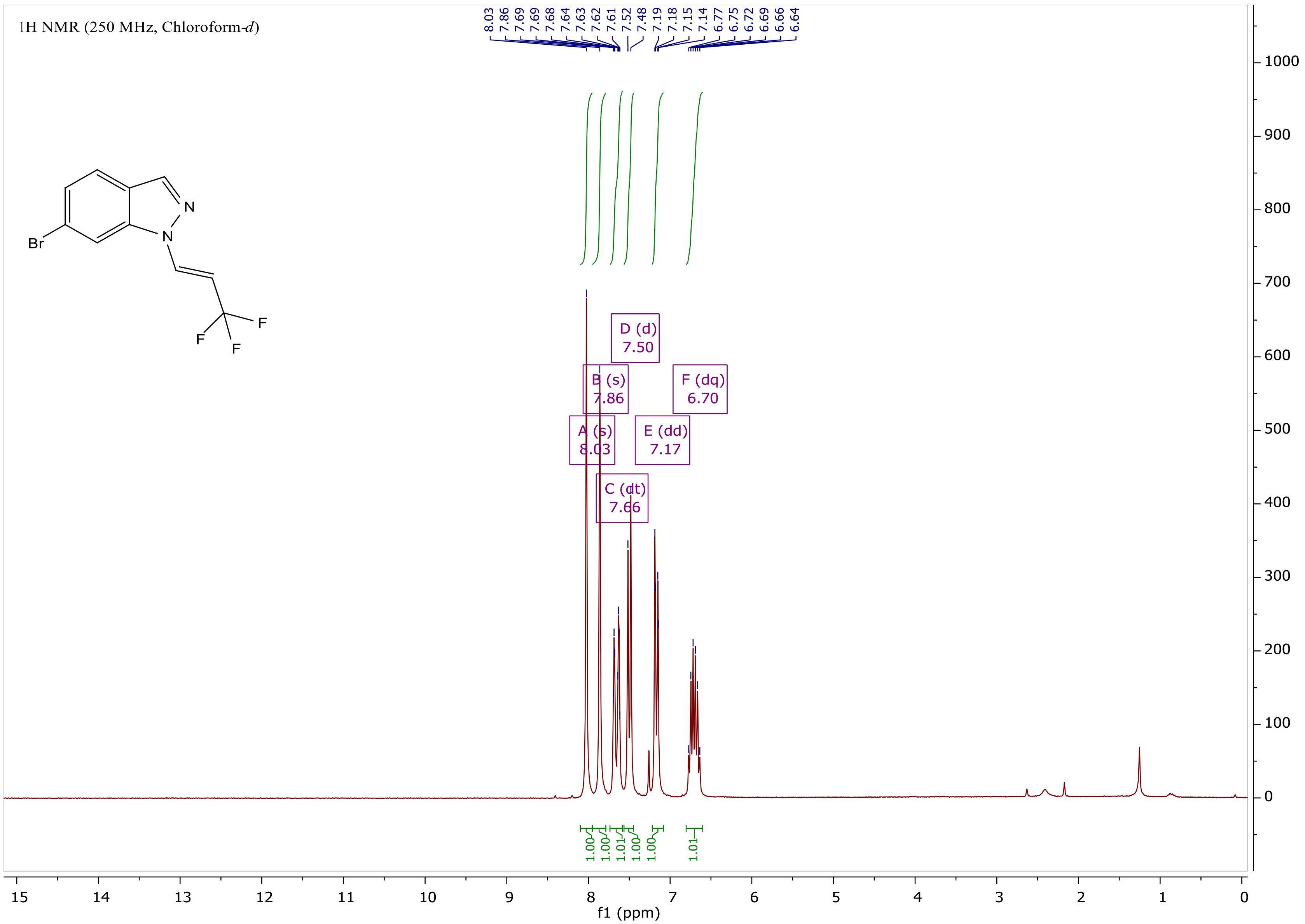

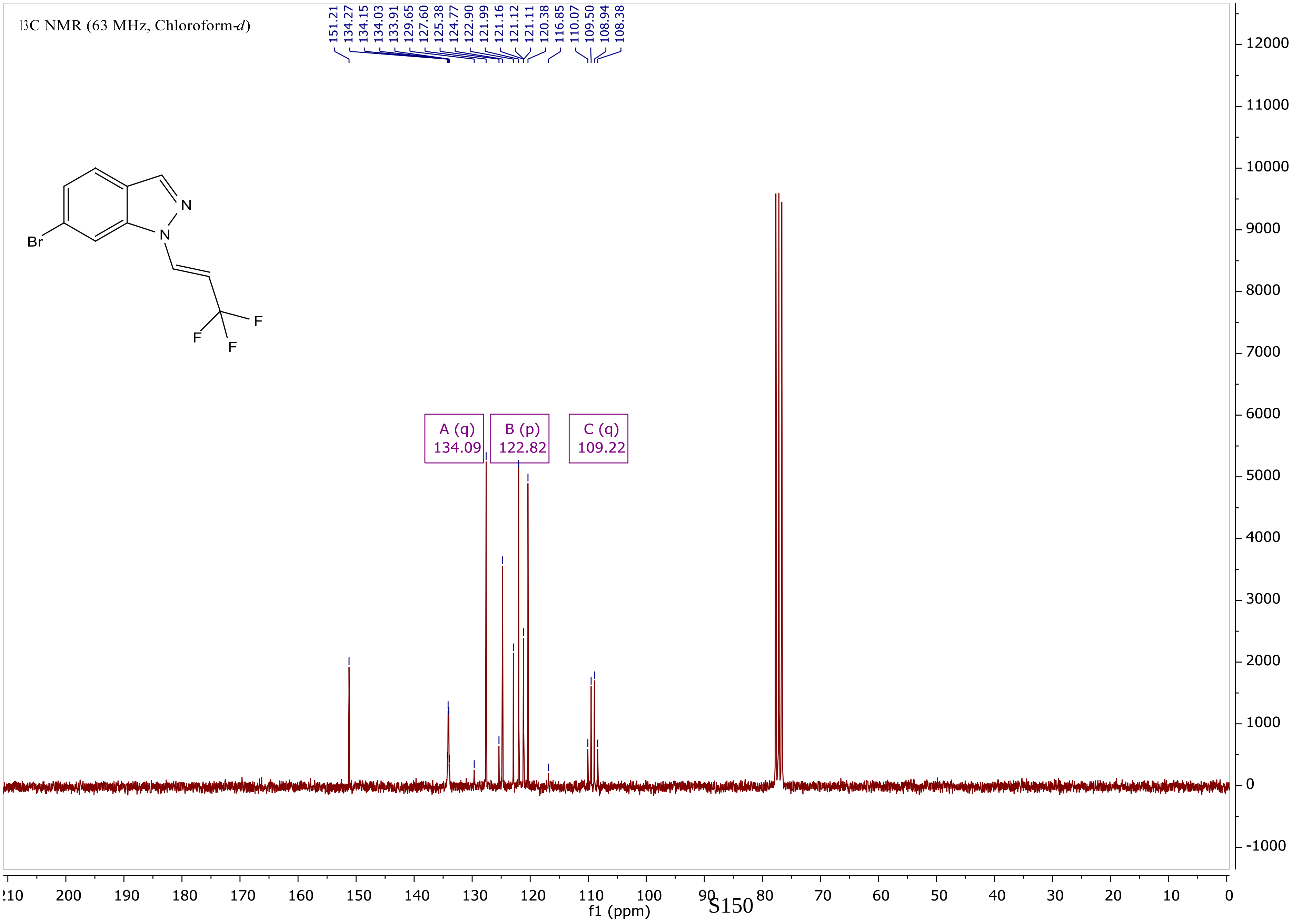
13C NMR (63 MHz, Chloroform-d)
151.21
134.27
134.15
134.03
133.91
129.65
127.60
125.38
124.77
122.90
121.99
121.16
121.12
121.11
120.38
116.85
110.07
109.50
108.94
108.38
Br
N
N
F
F
F
A (q)
134.09
B (p)
122.82
C (q)
109.22
f1 (ppm)

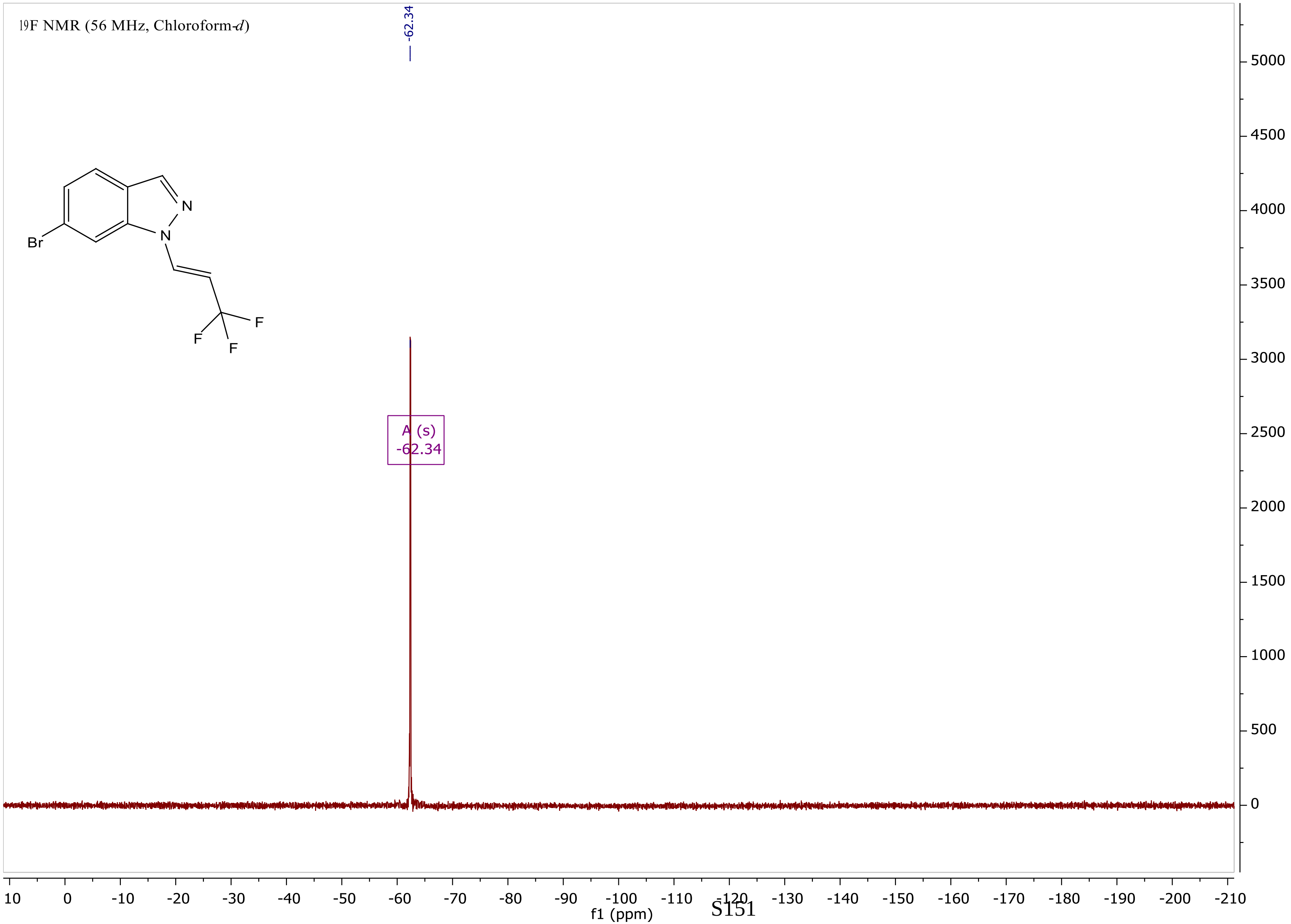
19F NMR (56 MHz, Chloroform-d)
-62.34
A (s)
-62.34
Br
N
N
F
F
F
5000
4500
4000
3500
3000
2500
2000
1500
1000
500
0
10 0 -10 -20 -30 -40 -50 -60 -70 -80 -90 -100 -110 -120 -130 -140 -150 -160 -170 -180 -190 -200 -210
f1 (ppm)

(*E*)-5-Nitro-1-(3,3,3-trifluoroprop-1-en-1-yl)-1*H*-indazole (33)

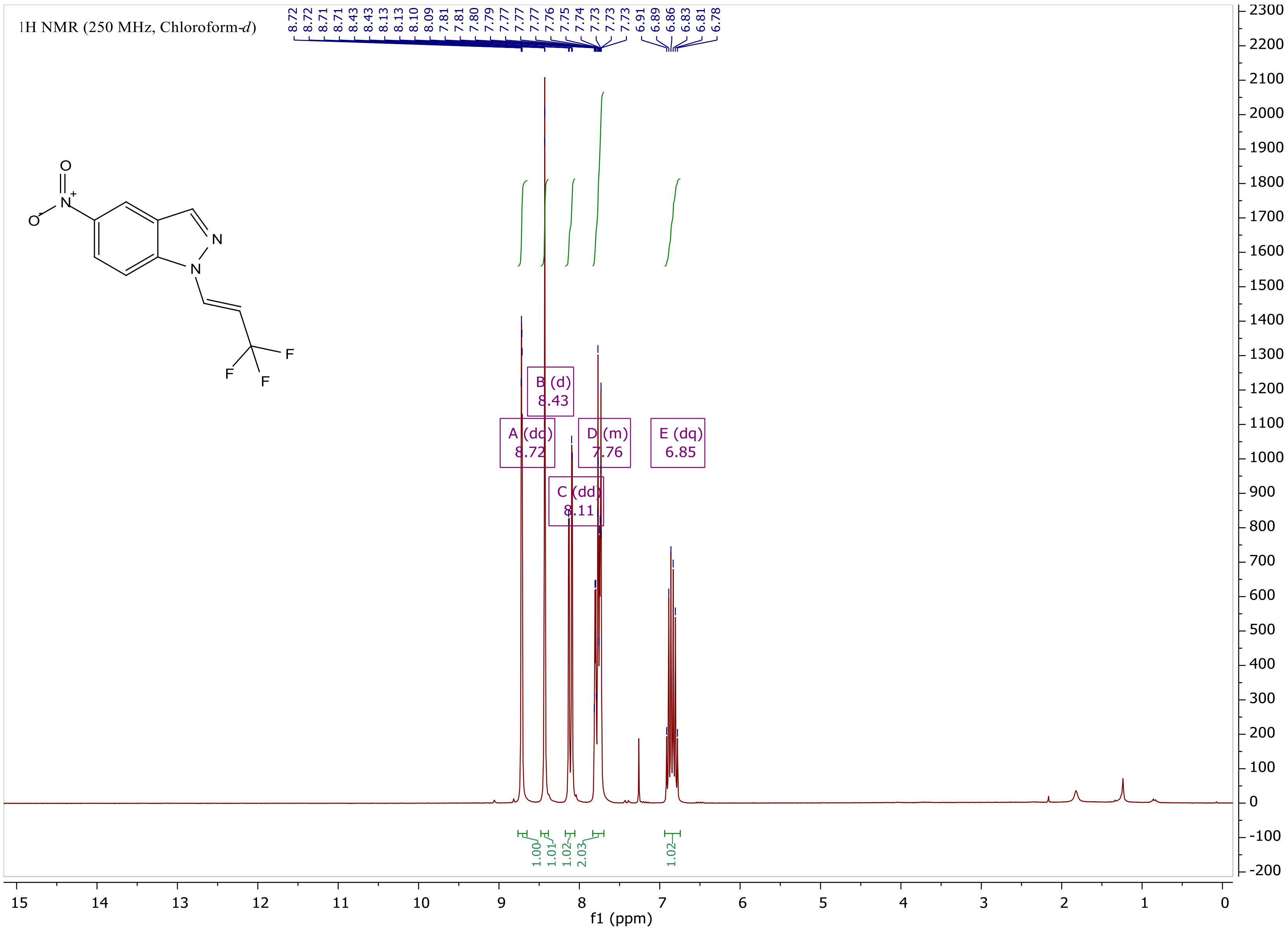

13C NMR (63 MHz, Chloroform-*d*)

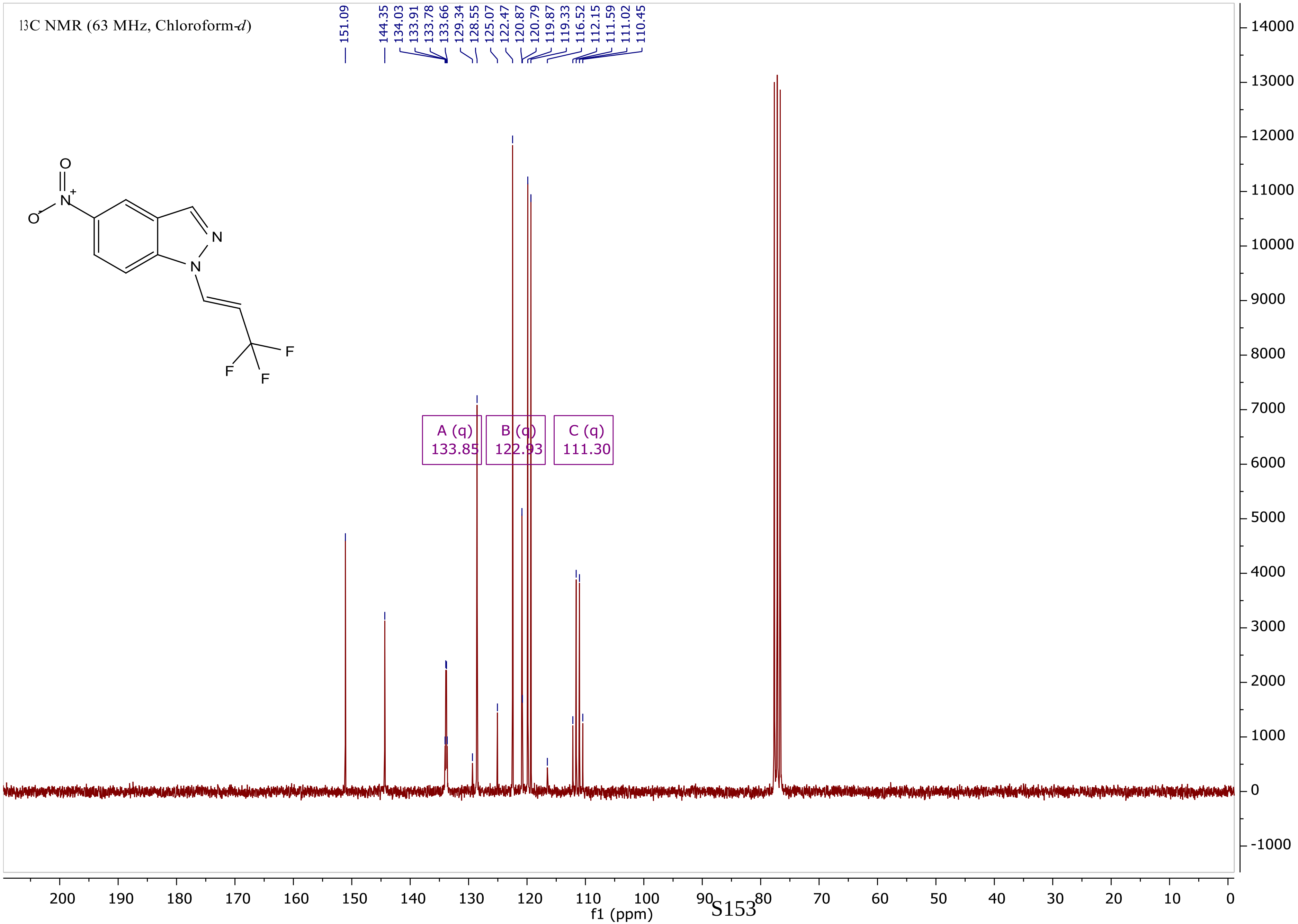

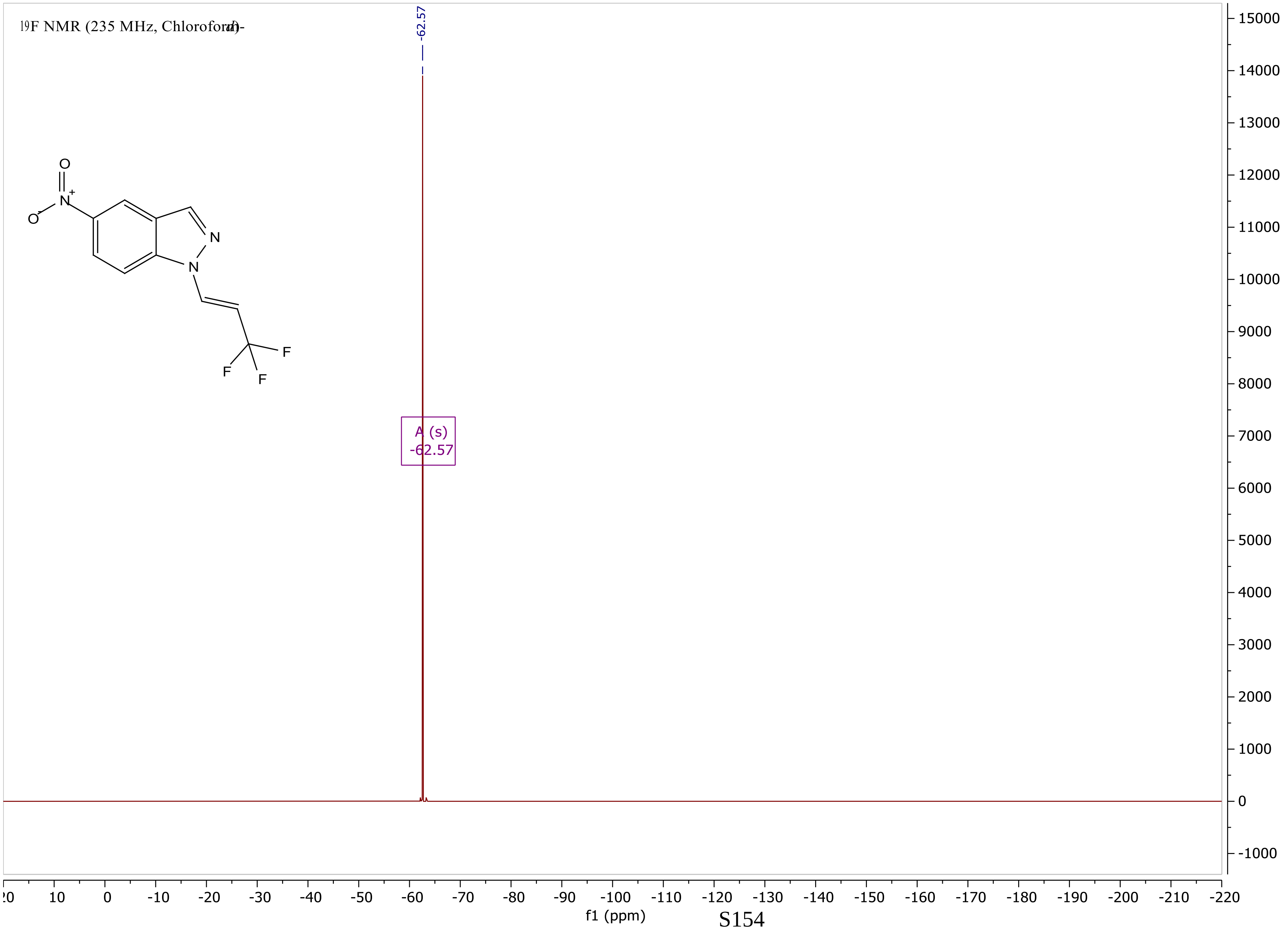
19F NMR (235 MHz, Chloroform-d)
-62.57
A (s)
-62.57
f1 (ppm)

(*E*)-5-((*tert*-Butyldimethylsilyl)oxy)-1-(3,3,3-trifluoroprop-1-en-1-yl)-1*H*-indazole (34)

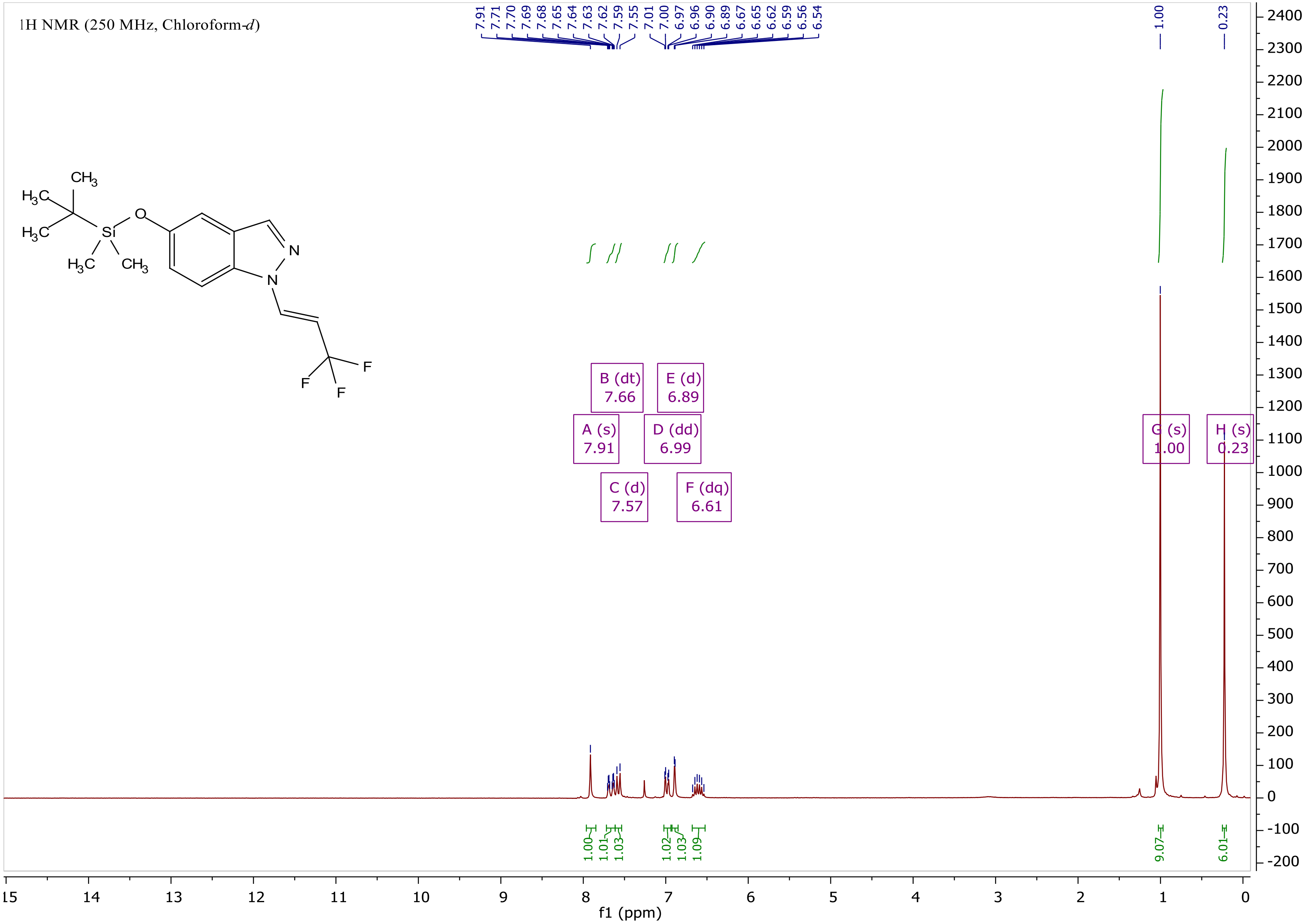

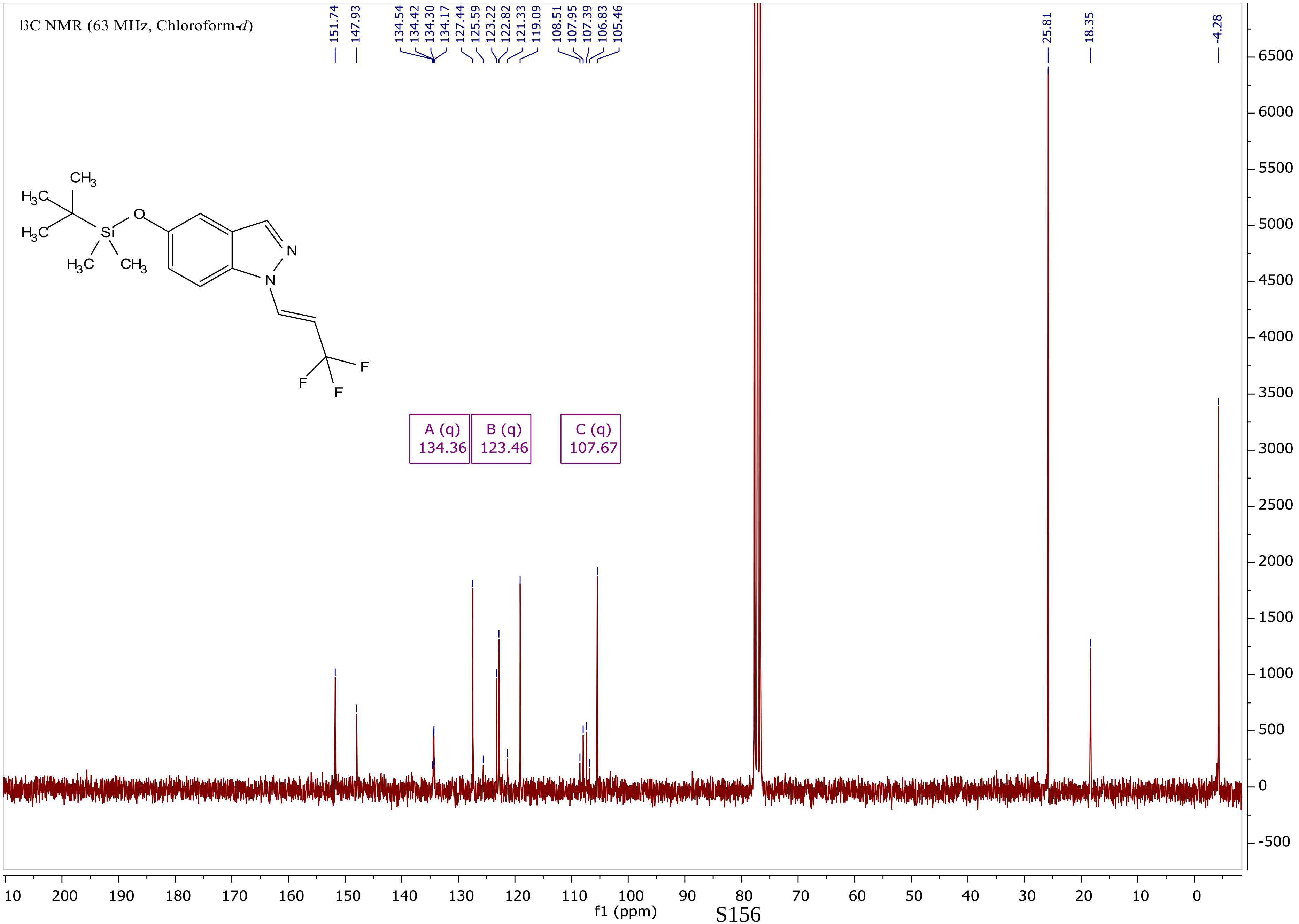
13C NMR (63 MHz, Chloroform-d)
151.74
147.93
134.54
134.42
134.30
134.17
127.44
125.59
123.22
122.82
121.33
119.09
108.51
107.95
107.39
106.83
105.46
25.81
18.35
-4.28
A (q) 134.36
B (q) 123.46
C (q) 107.67
f1 (ppm)

19F NMR (56 MHz, Chloroform-*d*)

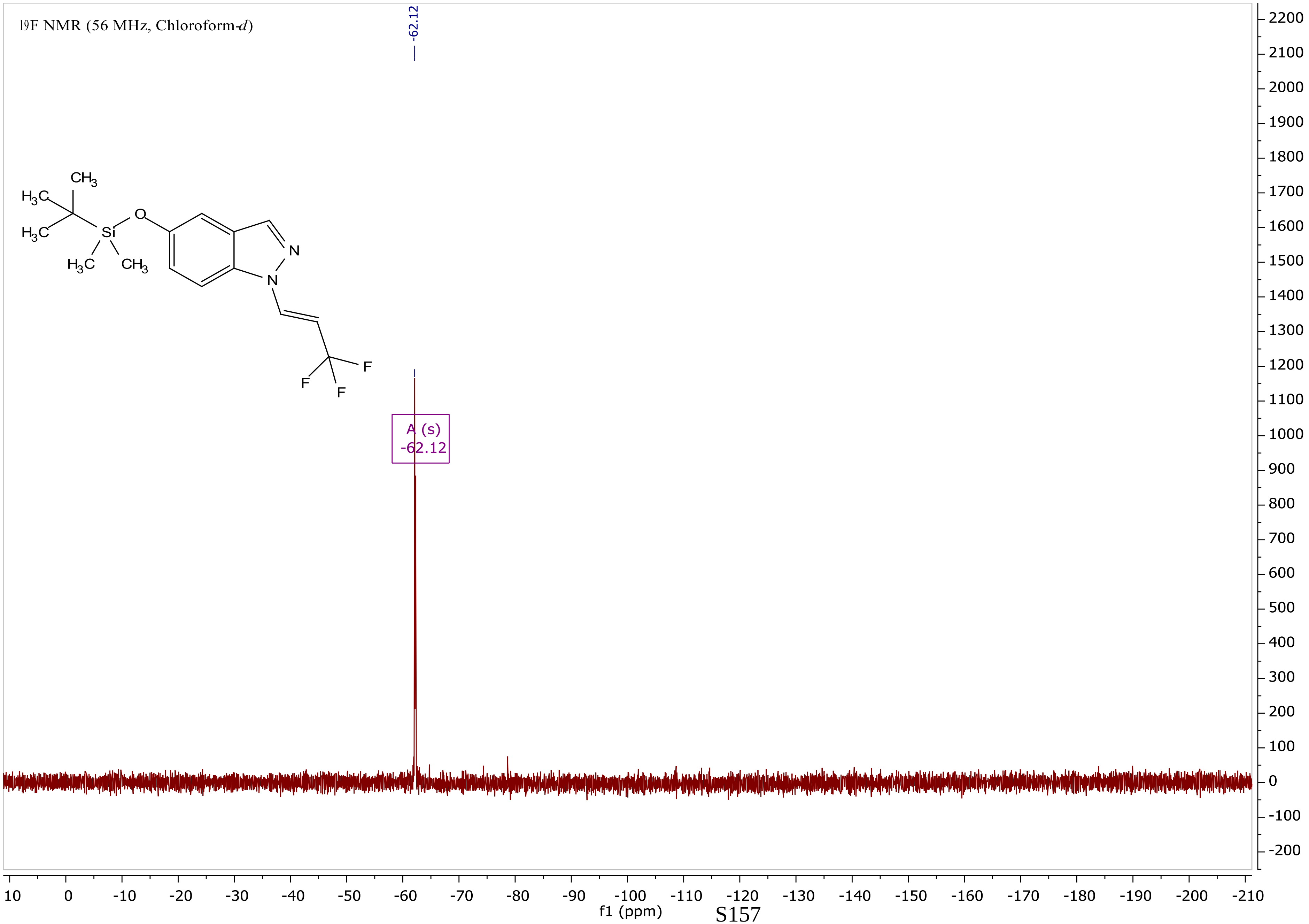

(*E*)-1-(3,3,3-Trifluoroprop-1-en-1-yl)-1*H*-benzo[d]imidazole (35)

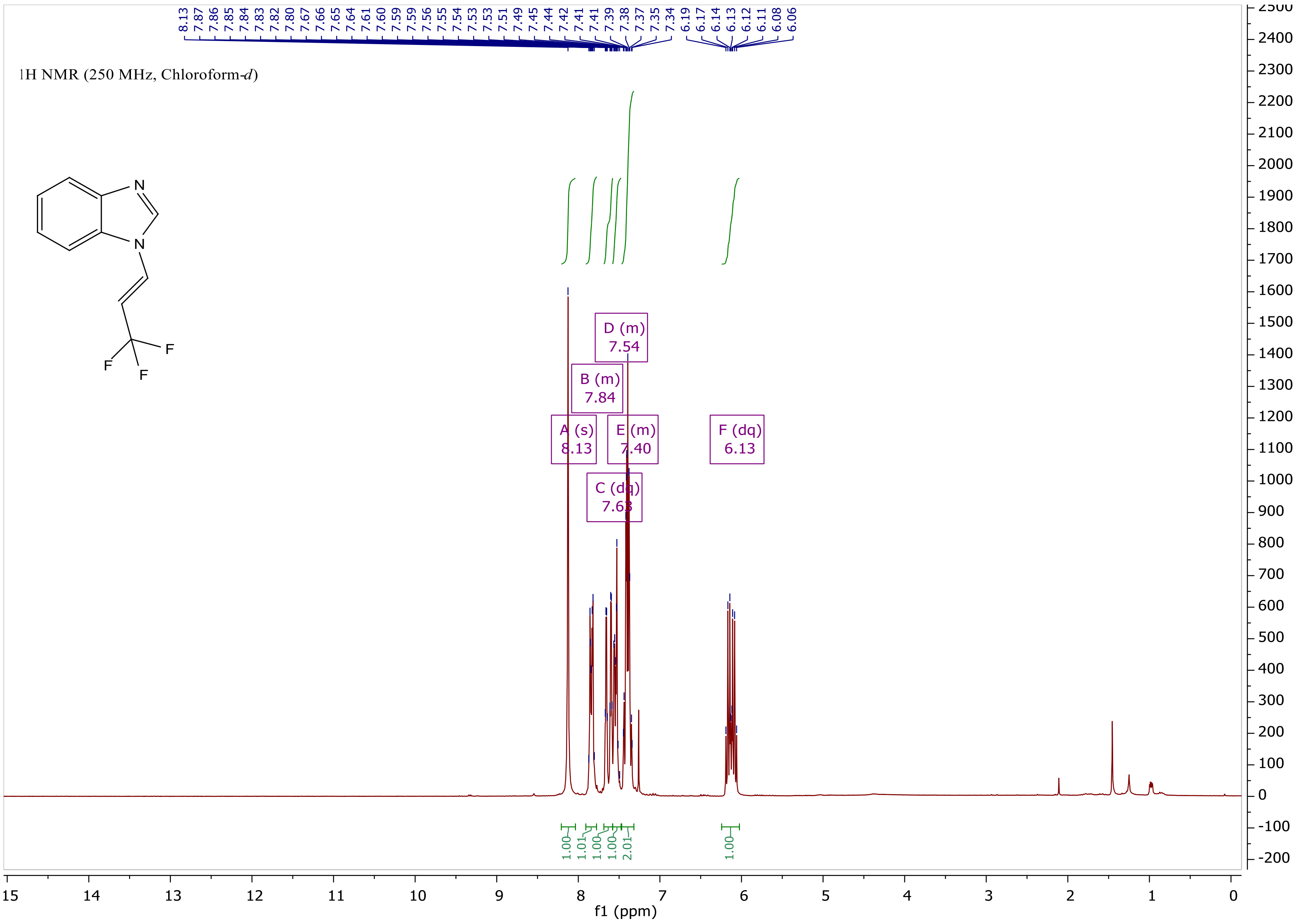

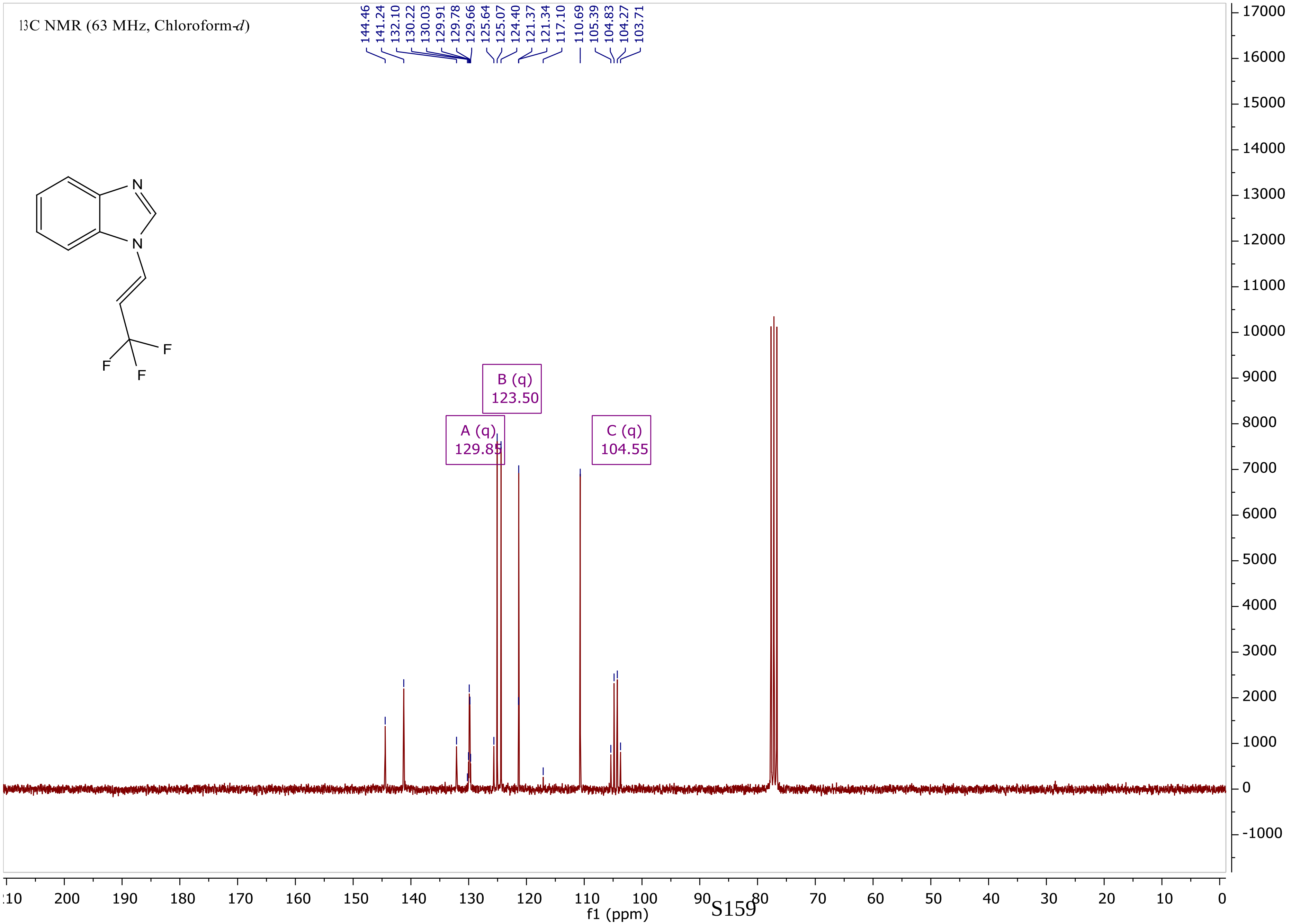
13C NMR (63 MHz, Chloroform-d)
144.46
141.24
132.10
130.22
130.03
129.91
129.78
129.66
125.64
125.07
124.40
121.37
121.34
117.10
110.69
105.39
104.83
104.27
103.71
N
N
F
F
F
A (q)
129.85
B (q)
123.50
C (q)
104.55
f1 (ppm)

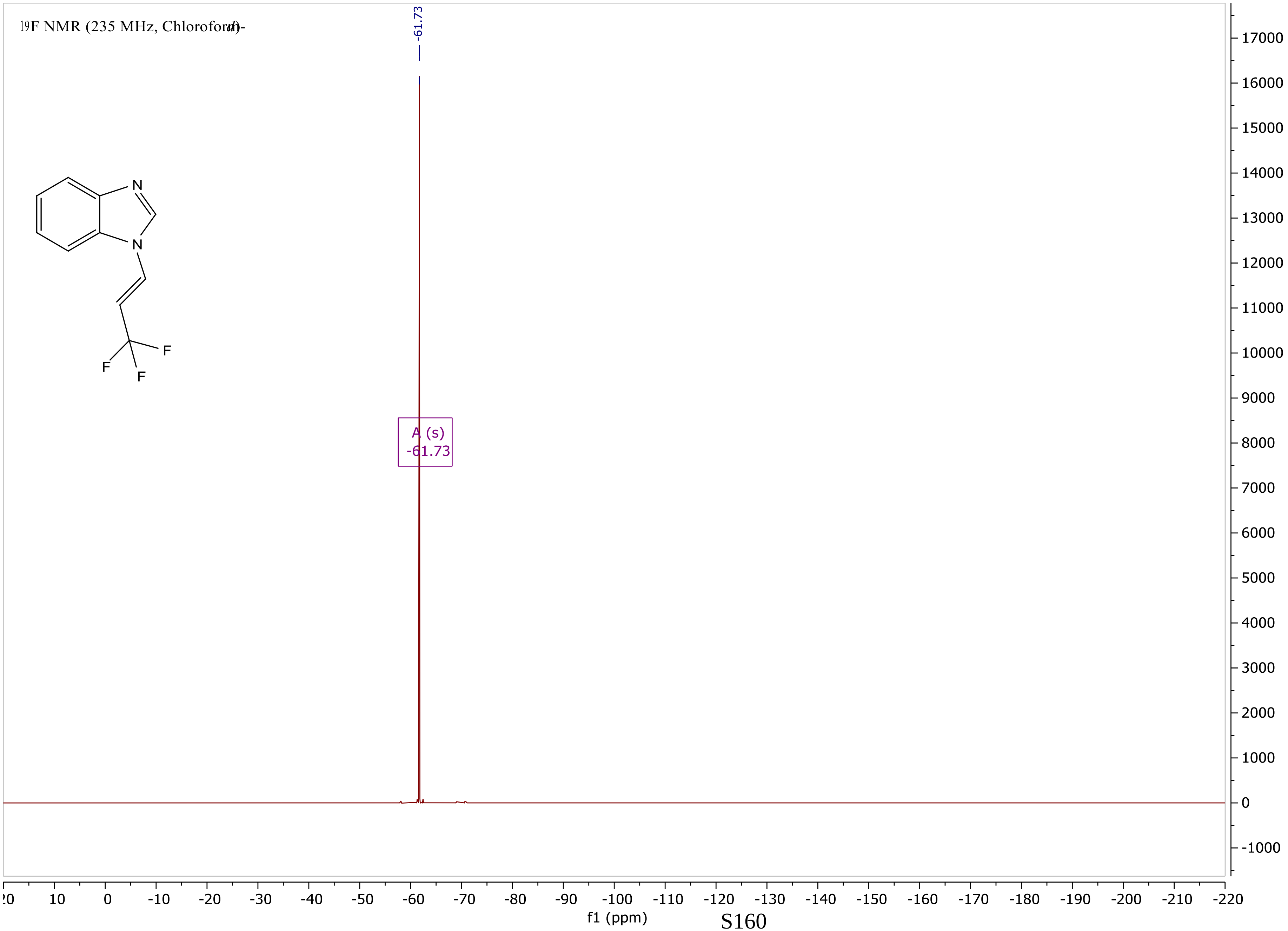
19F NMR (235 MHz, Chloroform-d)
-61.73
A (s)
-61.73
N
N
F
F
F
f1 (ppm)

(*E*)-5,6-Dimethyl-1-(3,3,3-trifluoroprop-1-en-1-yl)-1*H*-benzo[d]imidazole (36)

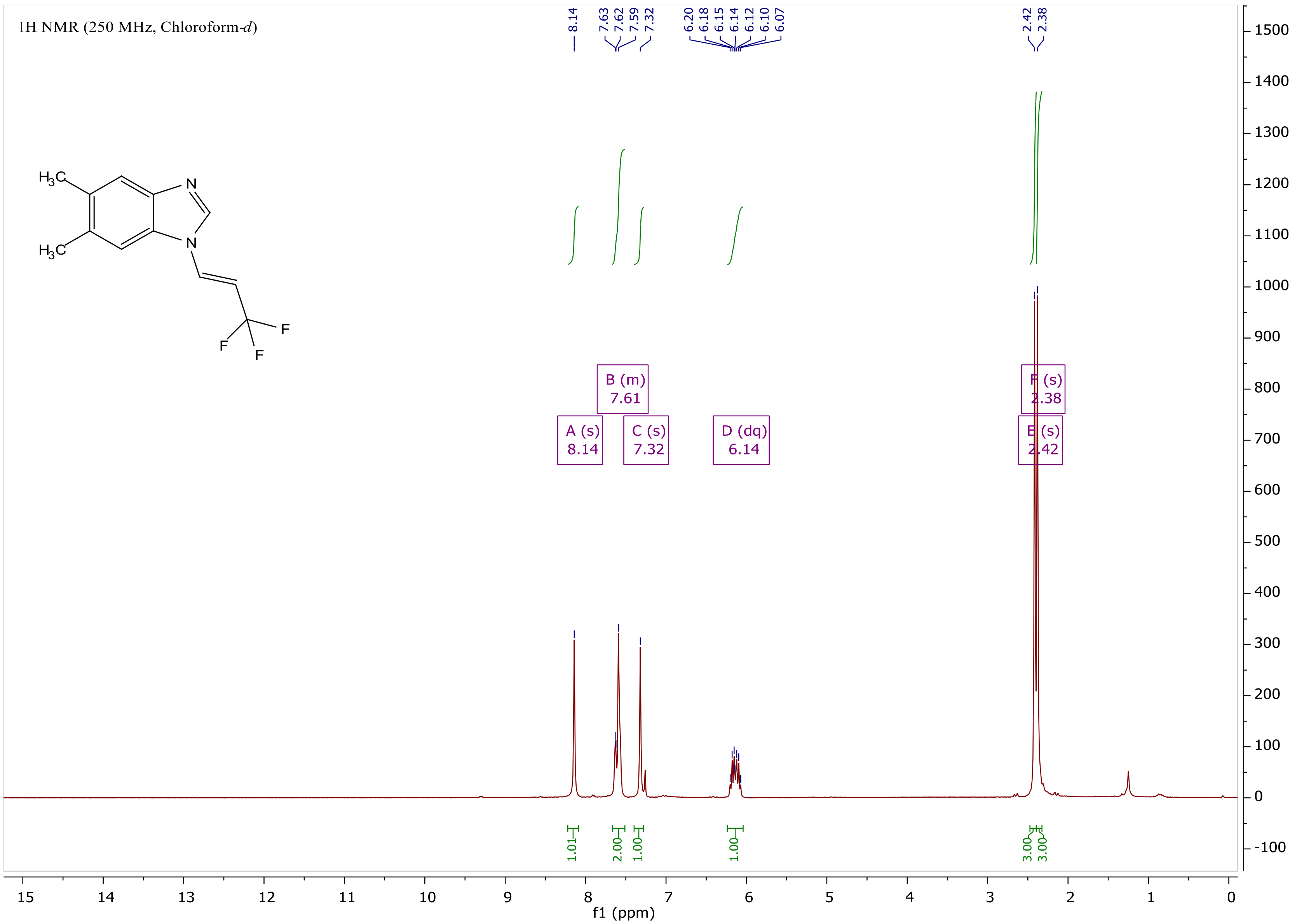

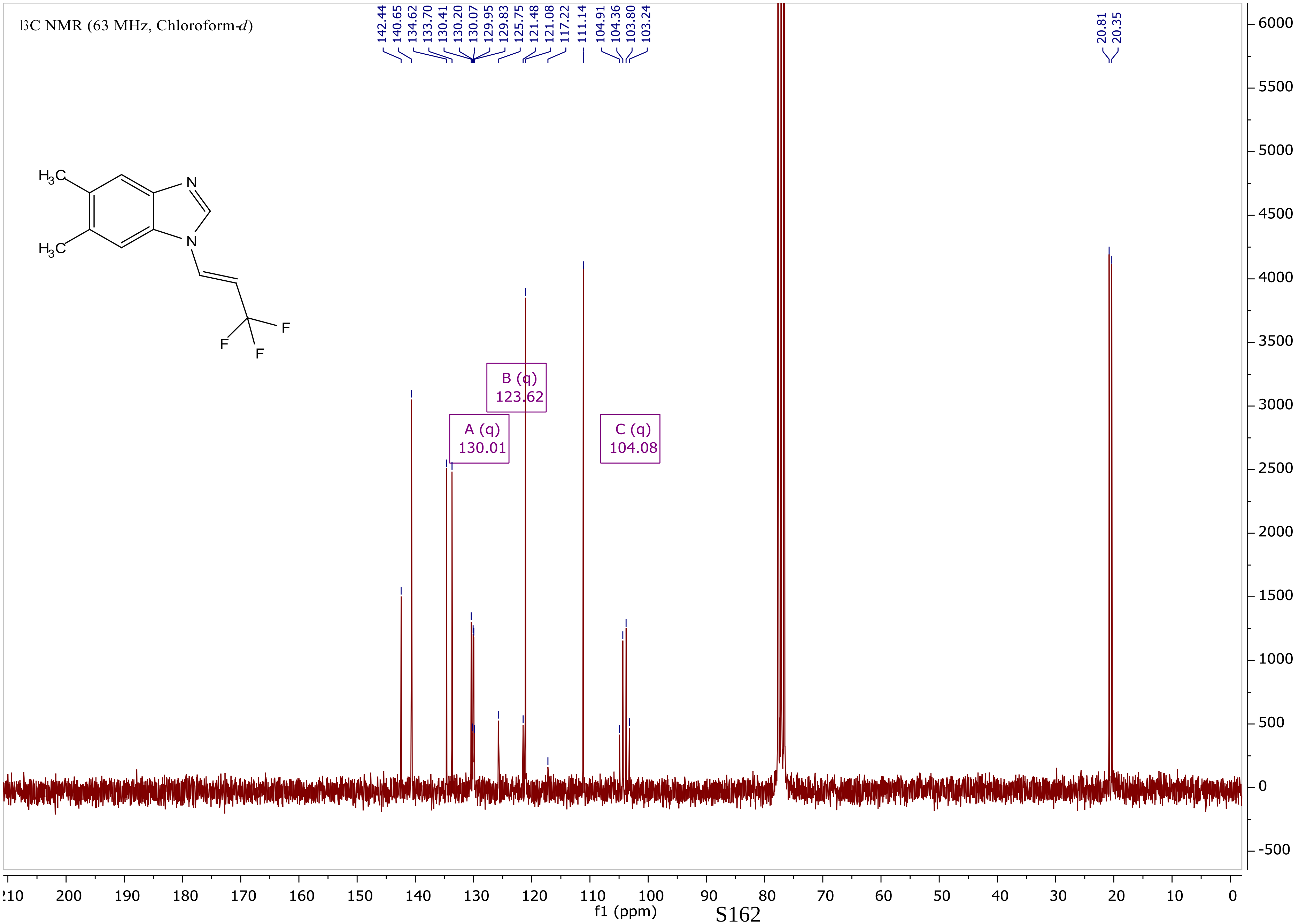
13C NMR (63 MHz, Chloroform-d)
142.44
140.65
134.62
133.70
130.41
130.20
130.07
129.95
129.83
125.75
121.48
121.08
117.22
111.14
104.91
104.36
103.80
103.24
20.81
20.35
A (q)
130.01
B (q)
123.62
C (q)
104.08
f1 (ppm)

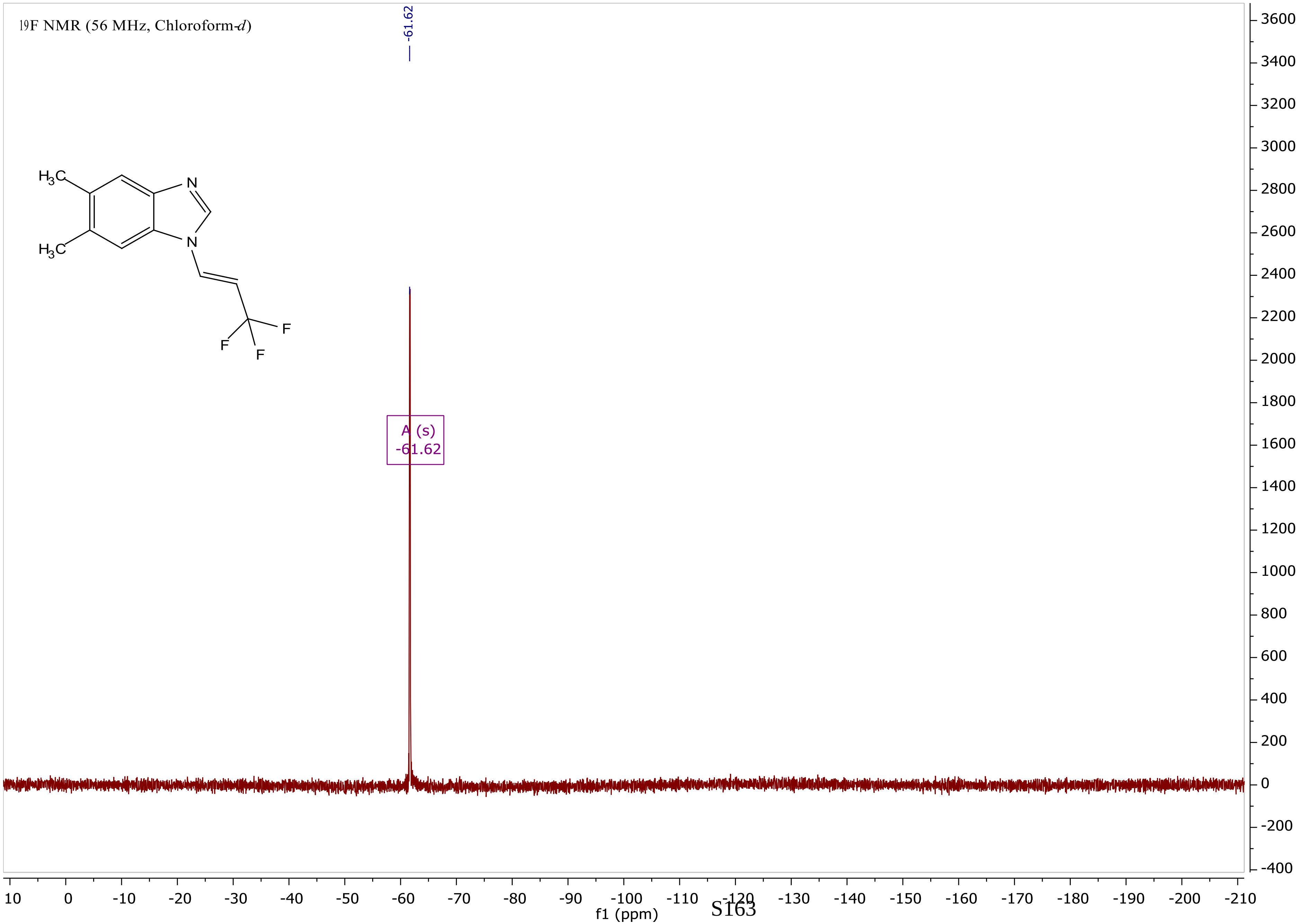
19F NMR (56 MHz, Chloroform-d)
-61.62
A (s)
-61.62
f1 (ppm)

(*E*)-5-Bromo-1-(3,3,3-trifluoroprop-1-en-1-yl)-1*H*-pyrrolo[2,3-b]pyridine (37)

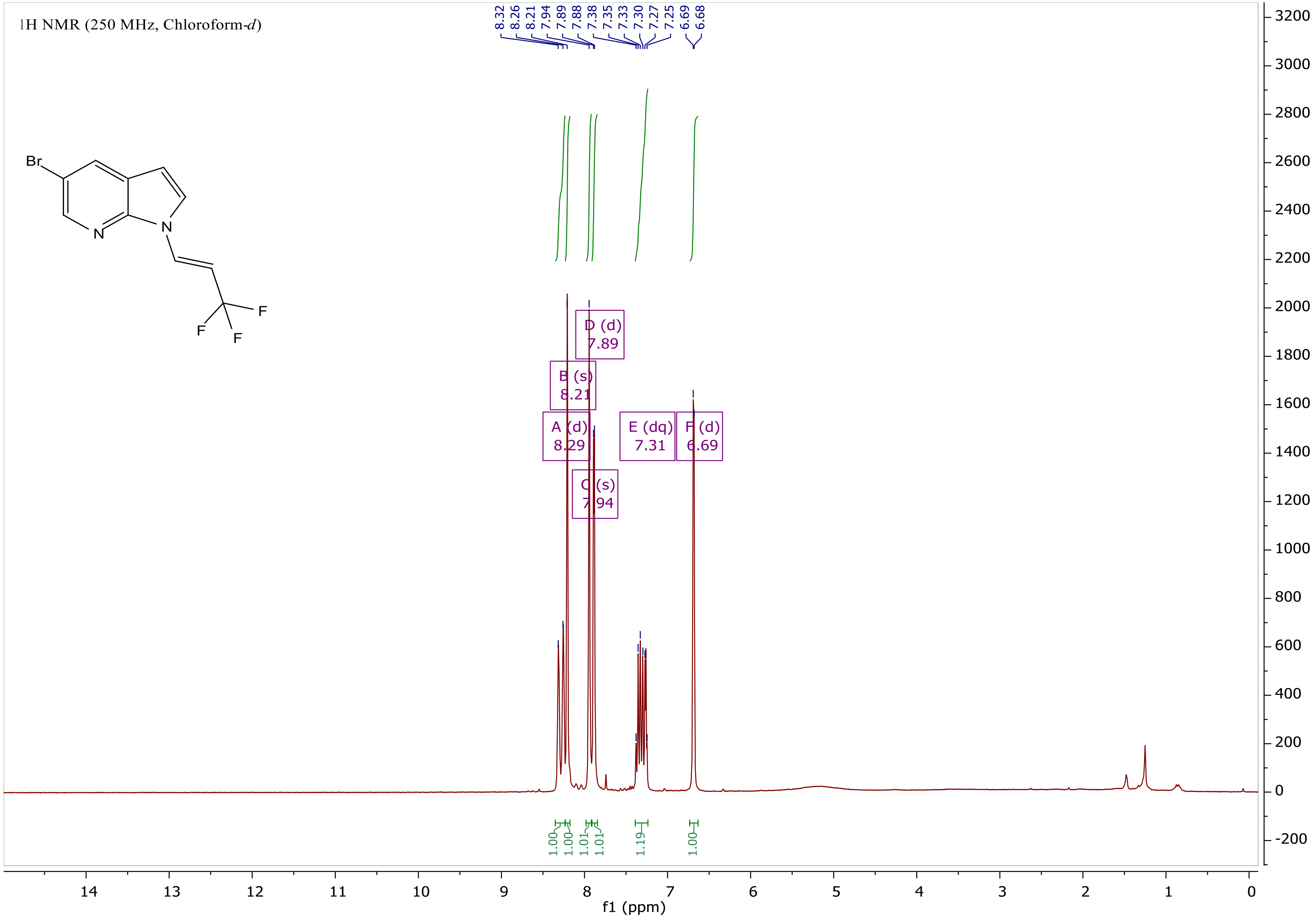

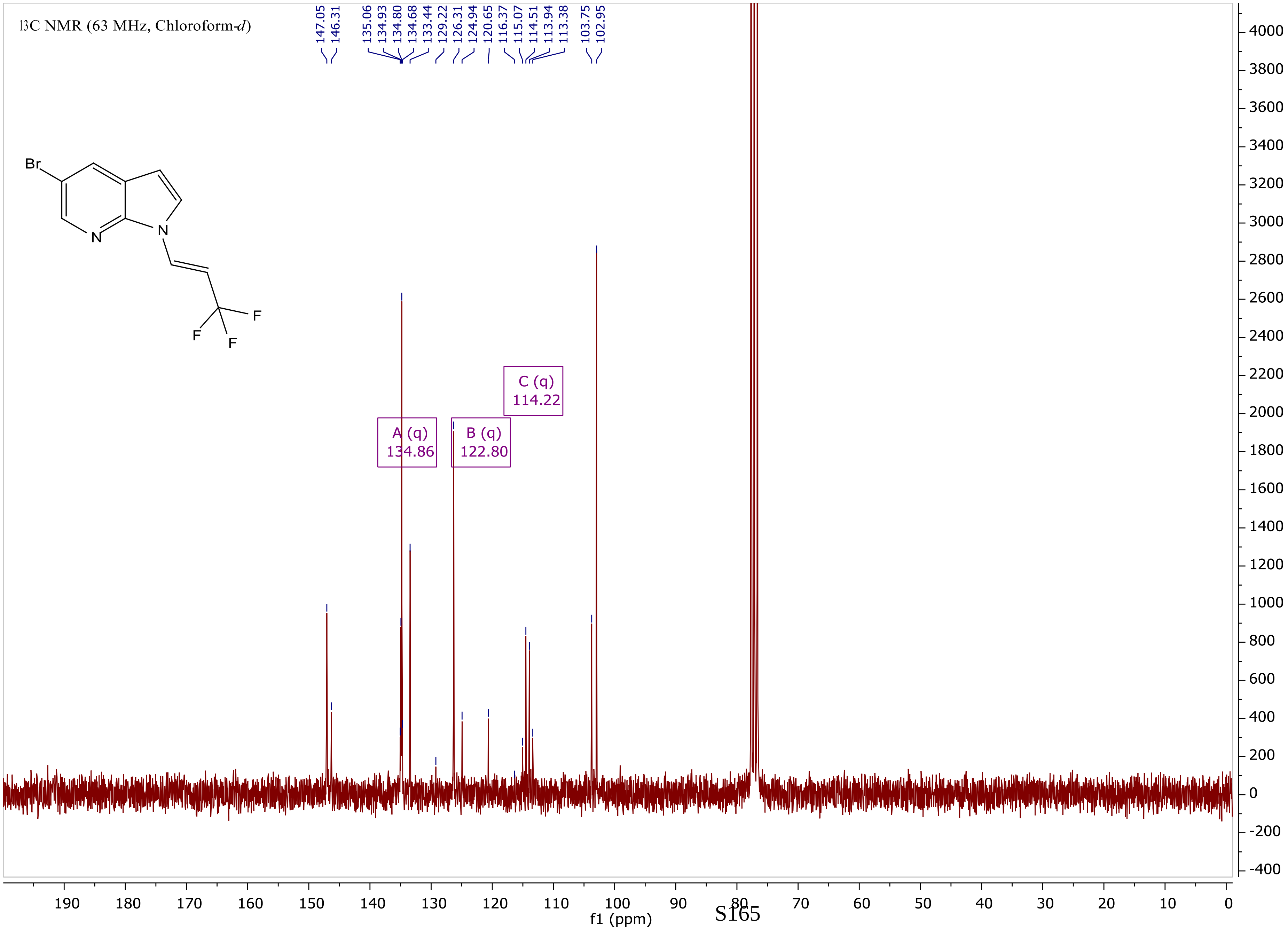
13C NMR (63 MHz, Chloroform-d)
147.05
146.31
135.06
134.93
134.80
134.68
133.44
129.22
126.31
124.94
120.65
116.37
115.07
114.51
113.94
113.38
103.75
102.95
Br
N
N
F
F
F
A (q)
134.86
B (q)
122.80
C (q)
114.22
f1 (ppm)

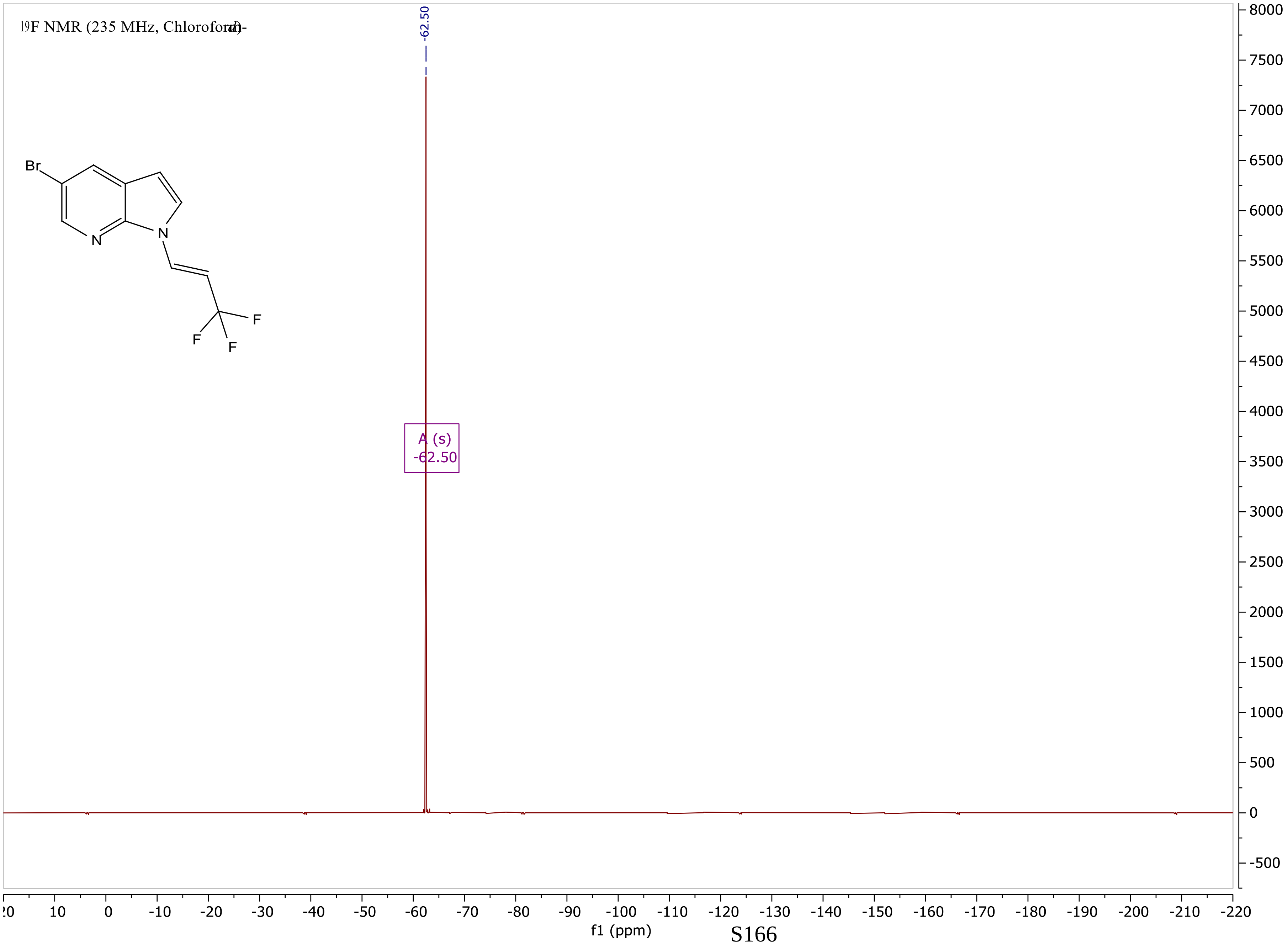
19F NMR (235 MHz, Chloroform-d)
-62.50
A (s)
-62.50
Br
N
N
F
F
F
f1 (ppm)

(*E*)-2-Chloro-3-methyl-1-(3,3,3-trifluoroprop-1-en-1-yl)-1*H*-pyrrolo[2,3-b]pyridine (38)

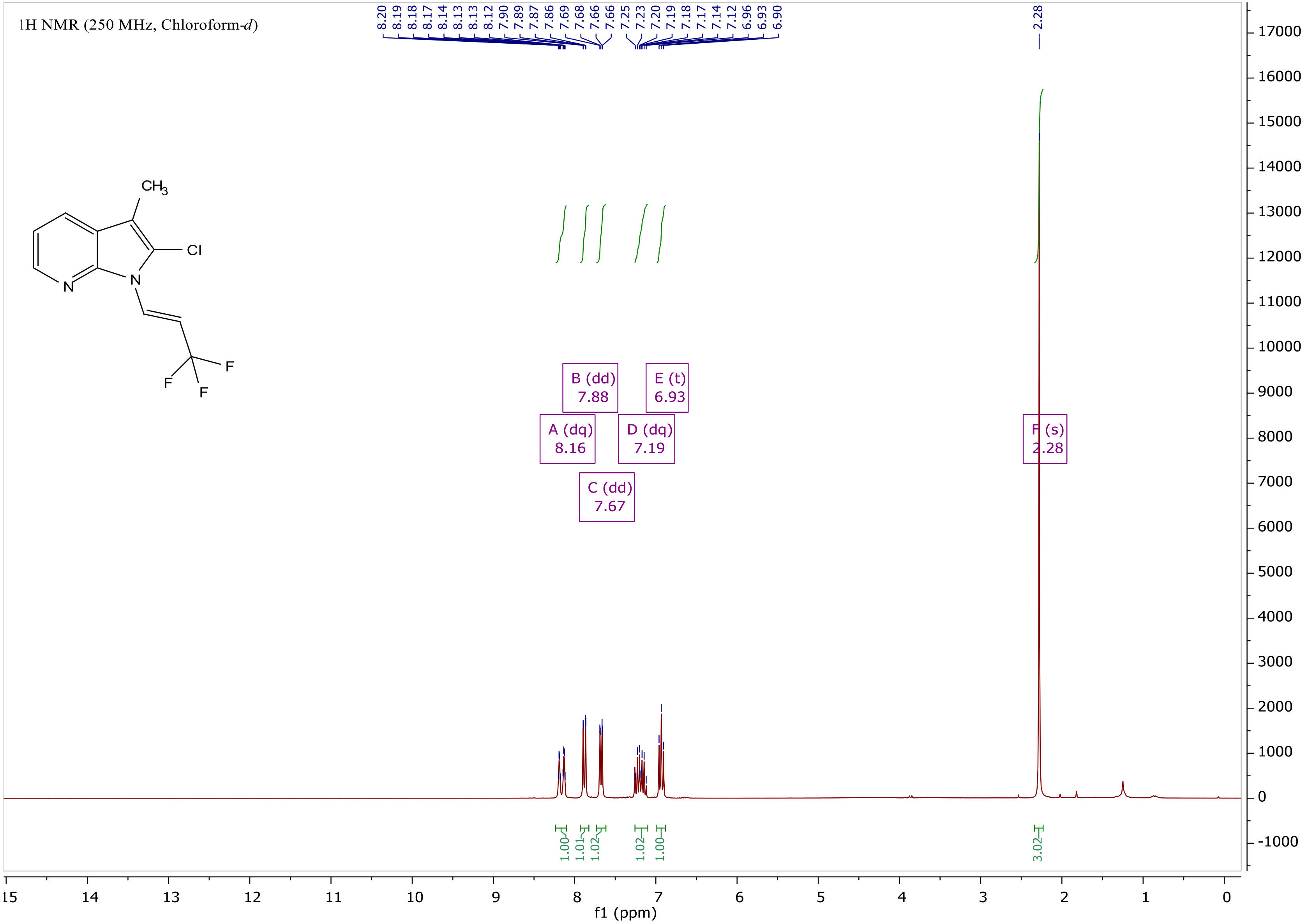

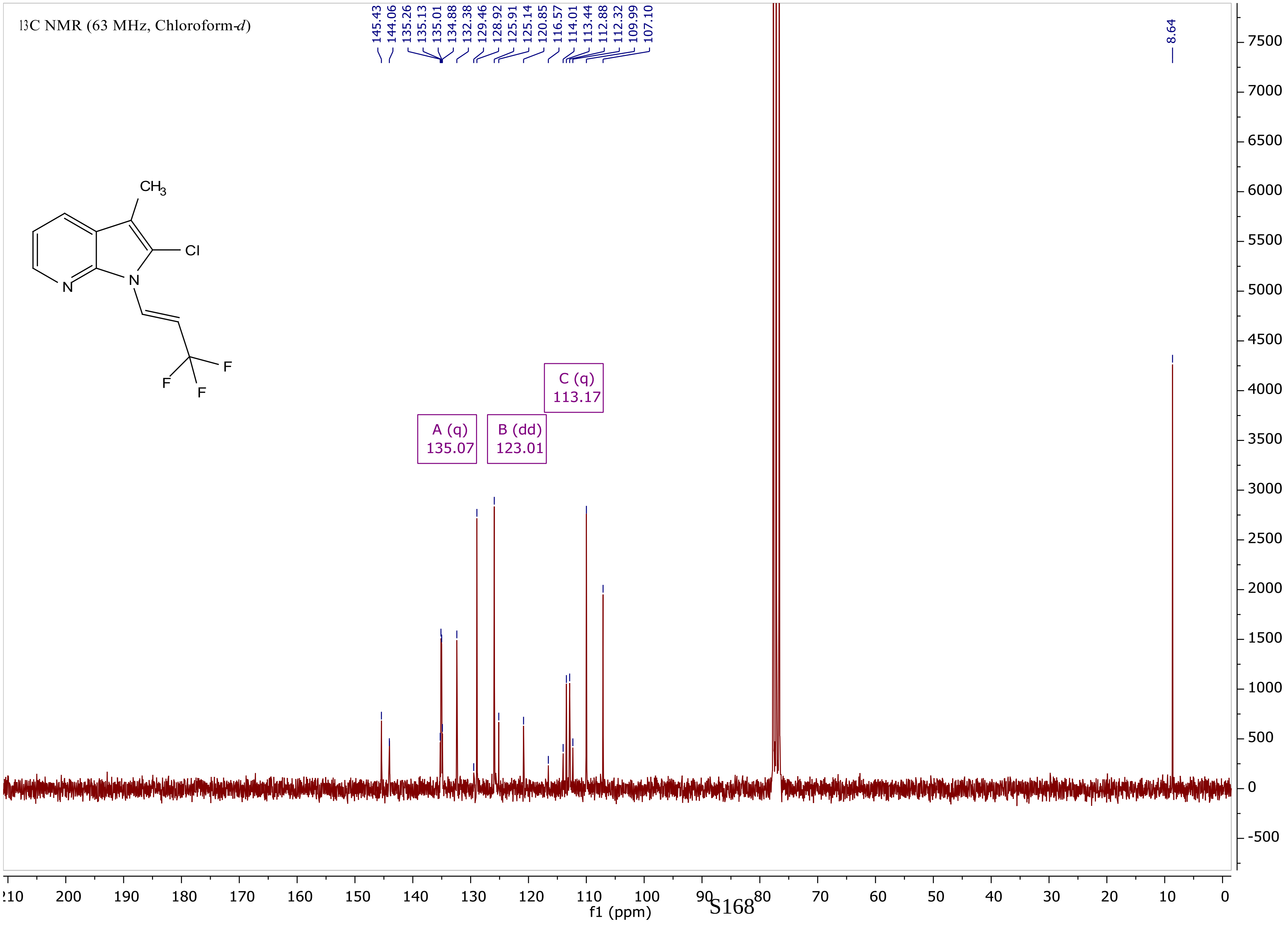
13C NMR (63 MHz, Chloroform-d)
145.43
144.06
135.26
135.13
135.01
134.88
132.38
129.46
128.92
125.91
125.14
120.85
116.57
114.01
113.44
112.88
112.32
109.99
107.10
8.64
CH3
Cl
N
N
F
F
F
A (q)
135.07
B (dd)
123.01
C (q)
113.17
f1 (ppm)

19F NMR (235 MHz, Chloroform-*d*)

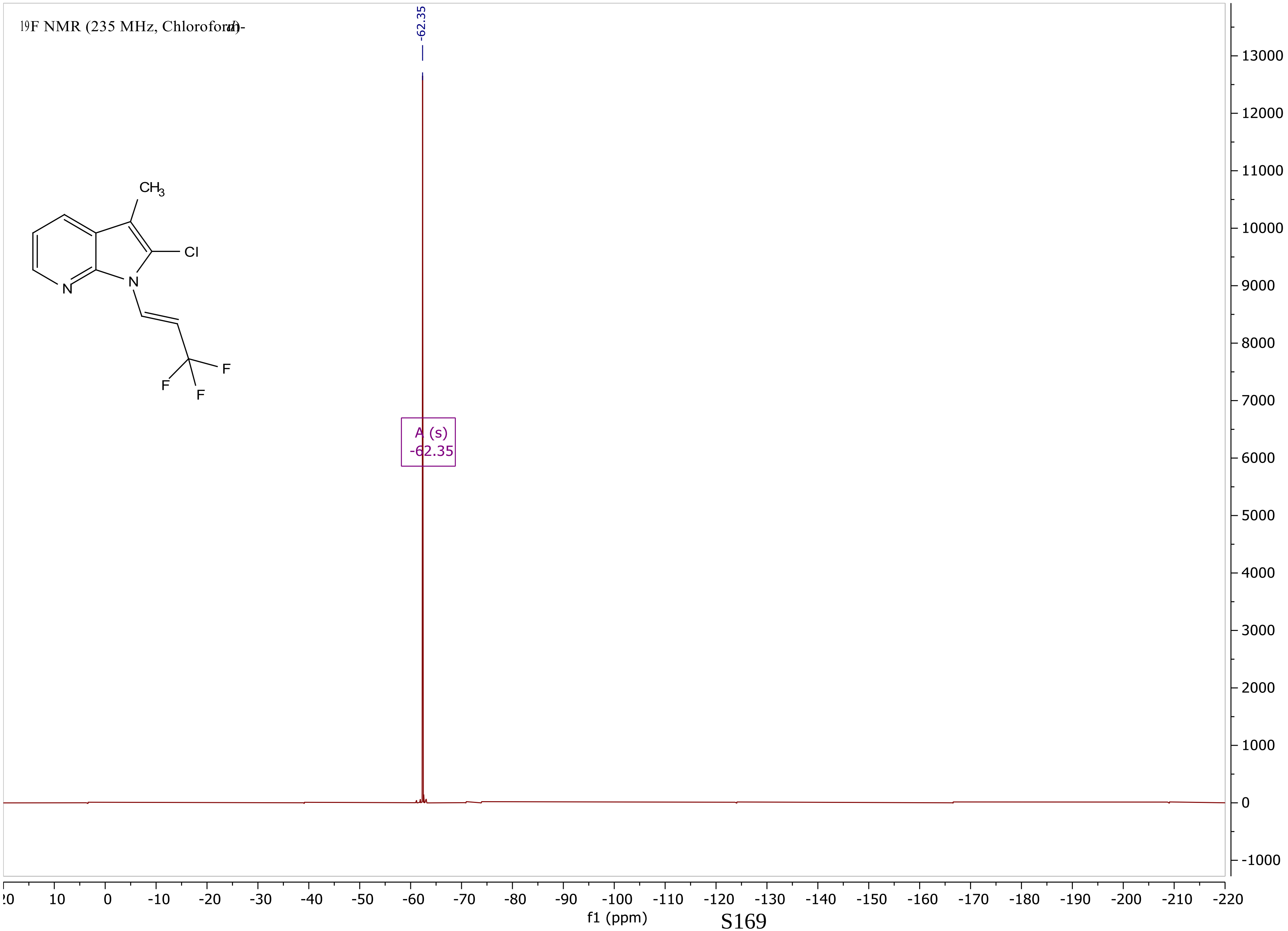

(*E*)-4-Nitro-1-(3,3,3-trifluoroprop-1-en-1-yl)-1*H*-benzo[d][1,2,3]triazole (39)

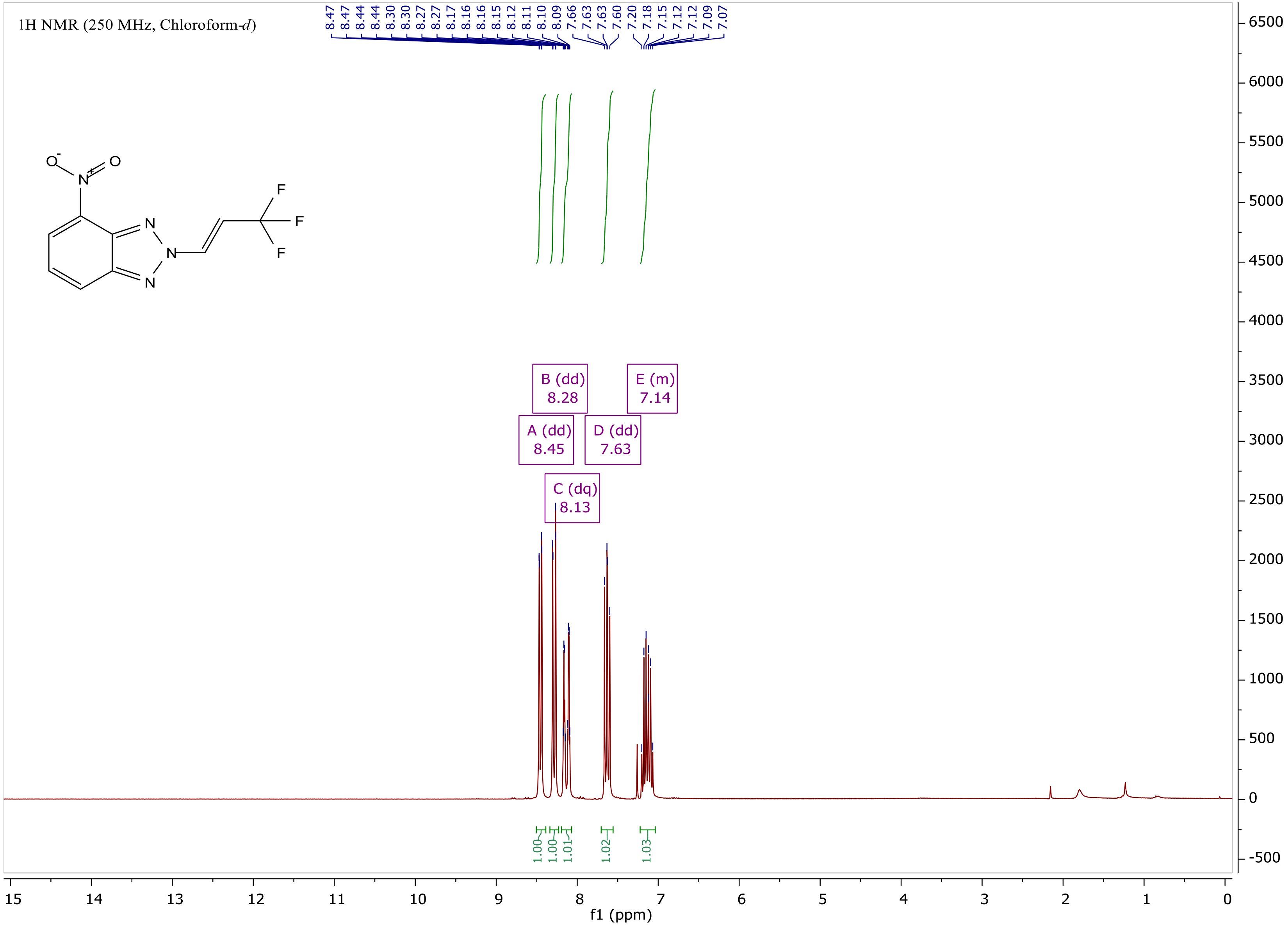

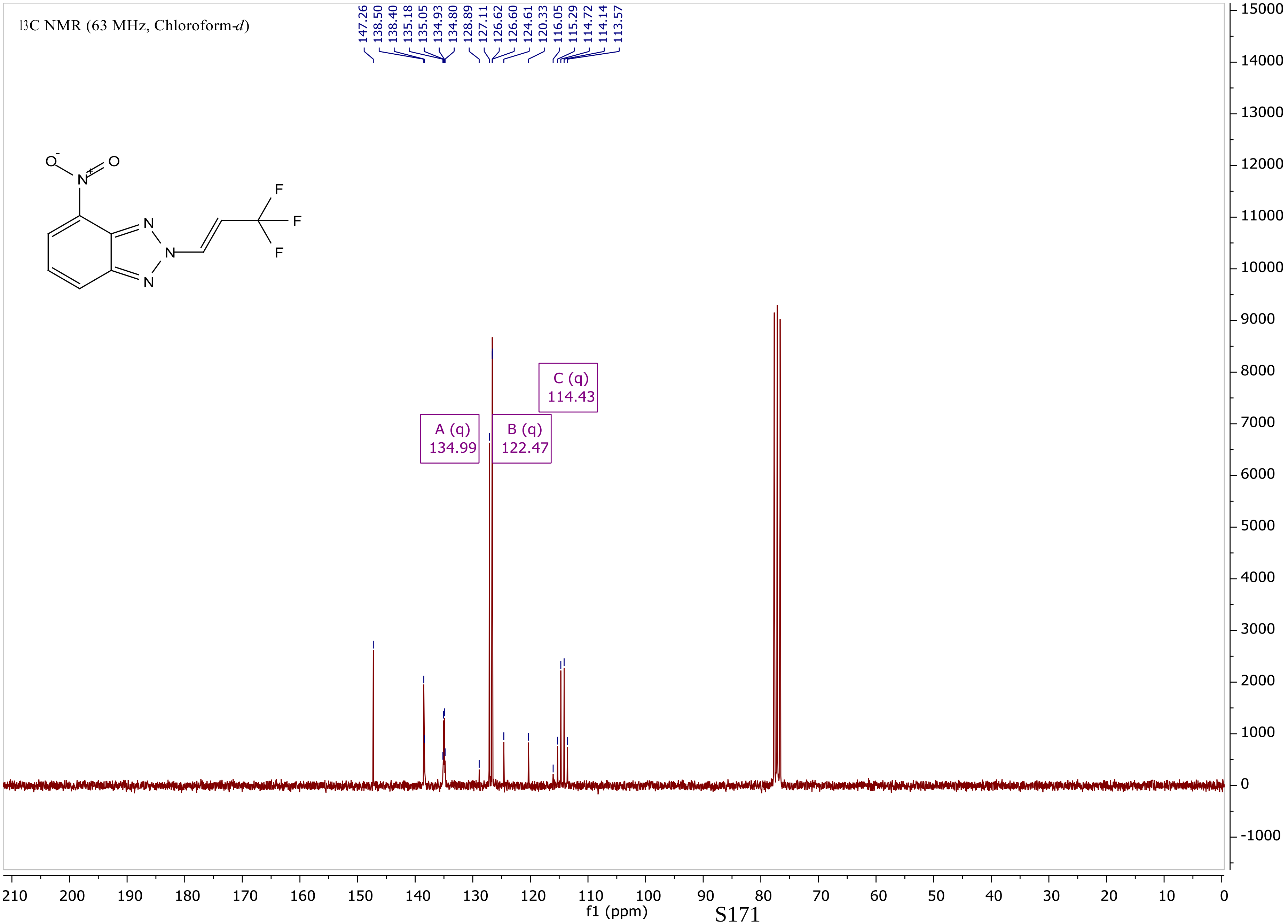

13C NMR (63 MHz, Chloroform-d)
147.26
138.50
138.40
135.18
135.05
134.93
134.80
128.89
127.11
126.62
126.60
124.61
120.33
116.05
115.29
114.72
114.14
113.57
A (q)
134.99
B (q)
122.47
C (q)
114.43
f1 (ppm)

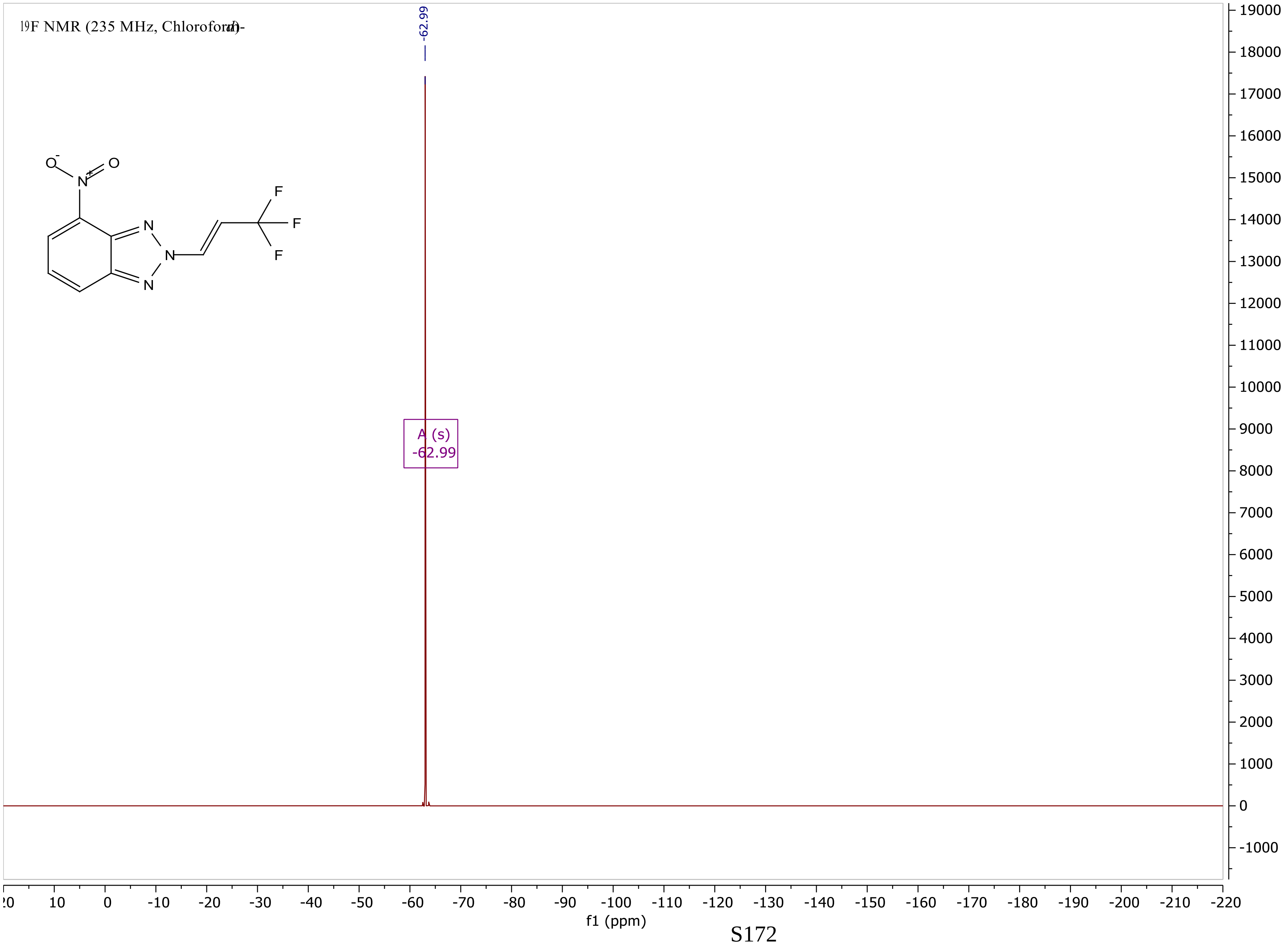

19F NMR (235 MHz, Chloroform-d)
-62.99
A (s)
-62.99
f1 (ppm)

(*E*)-4-Nitro-1-(3,3,3-trifluoroprop-1-en-1-yl)-1*H*-benzo[d][1,2,3]triazole (40)

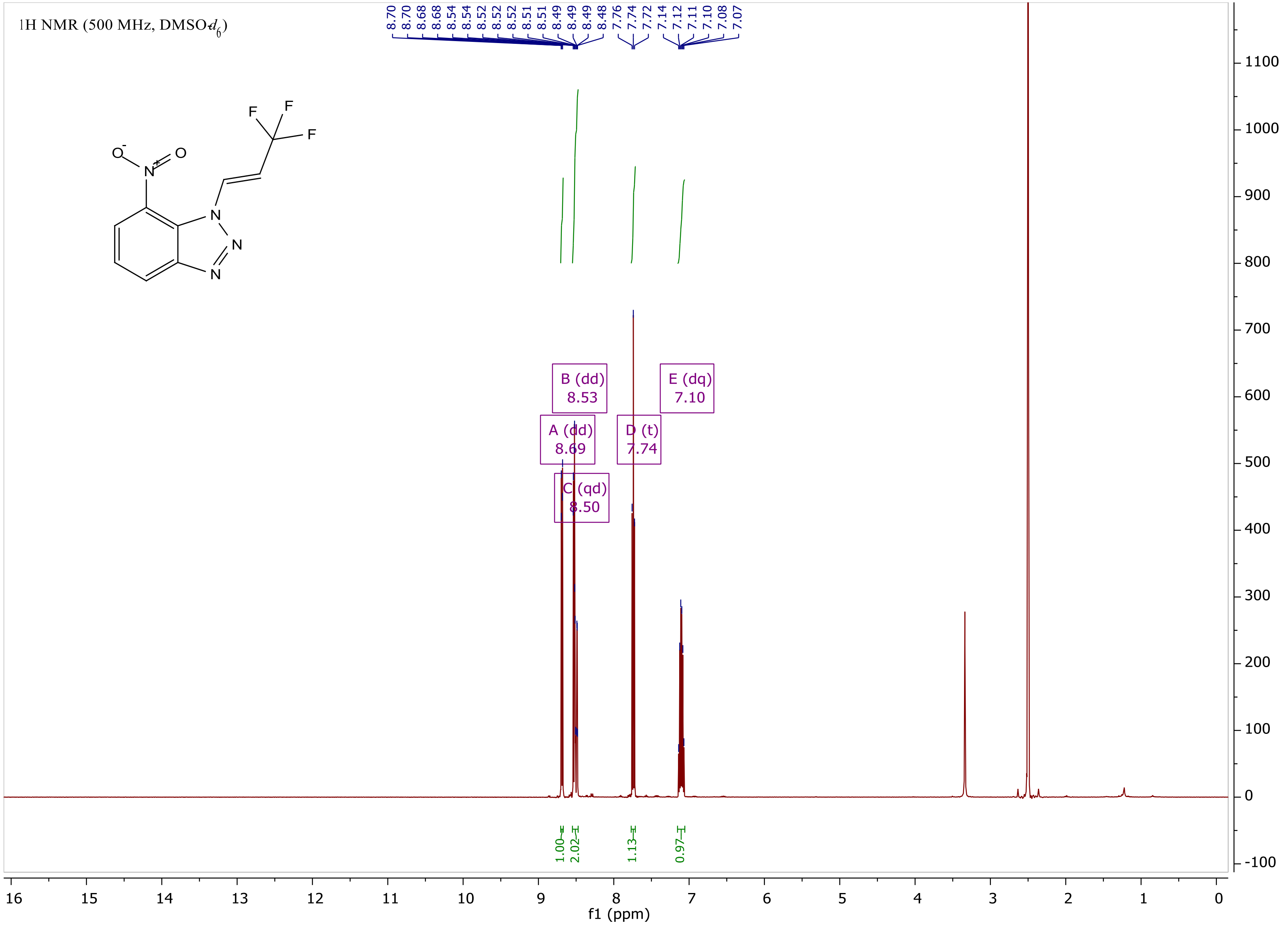

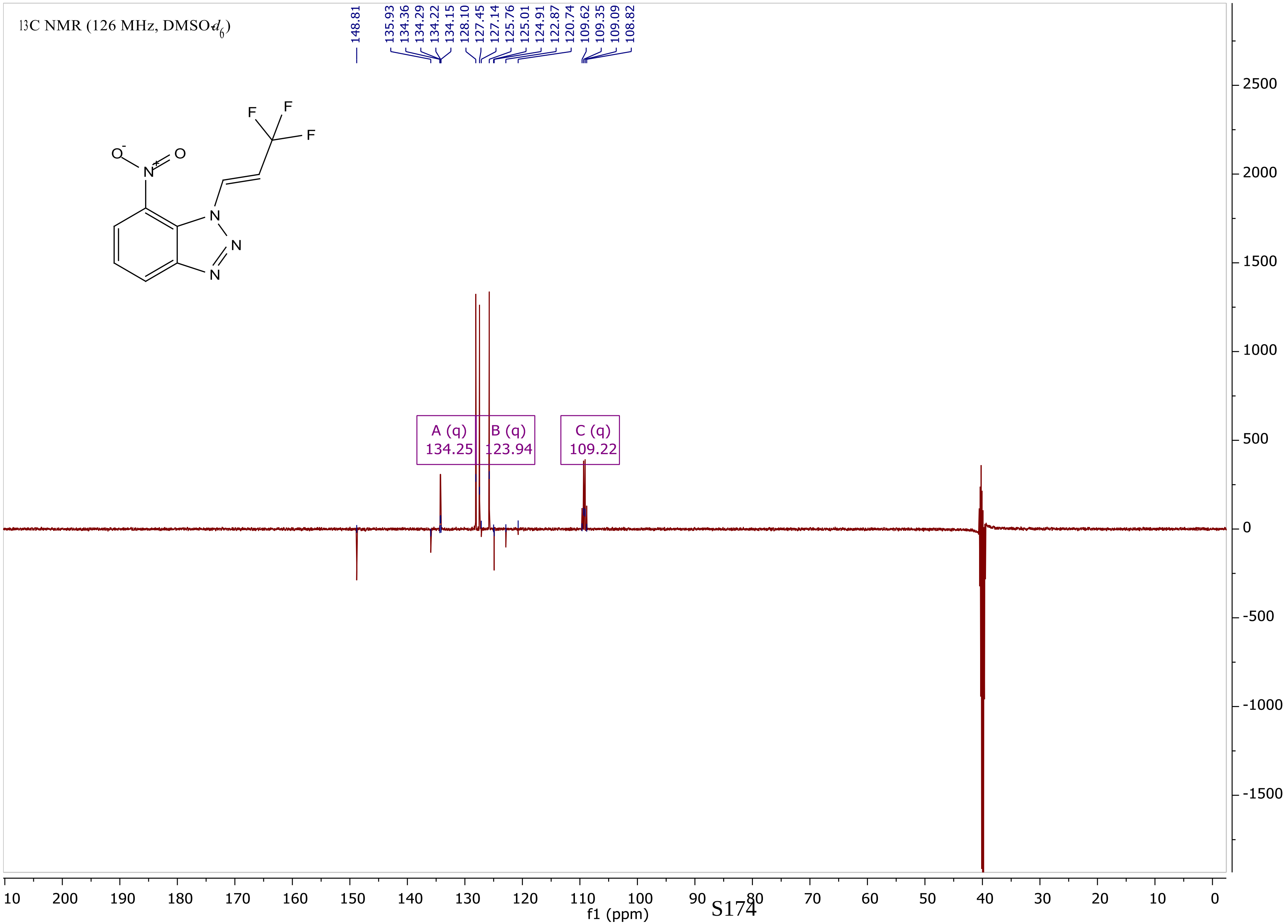
13C NMR (126 MHz, DMSO-d_6)
148.81
135.93
134.36
134.29
134.22
134.15
128.10
127.45
127.14
125.76
125.01
124.91
122.87
120.74
109.62
109.35
109.09
108.82
A (q)
134.25
B (q)
123.94
C (q)
109.22
f1 (ppm)

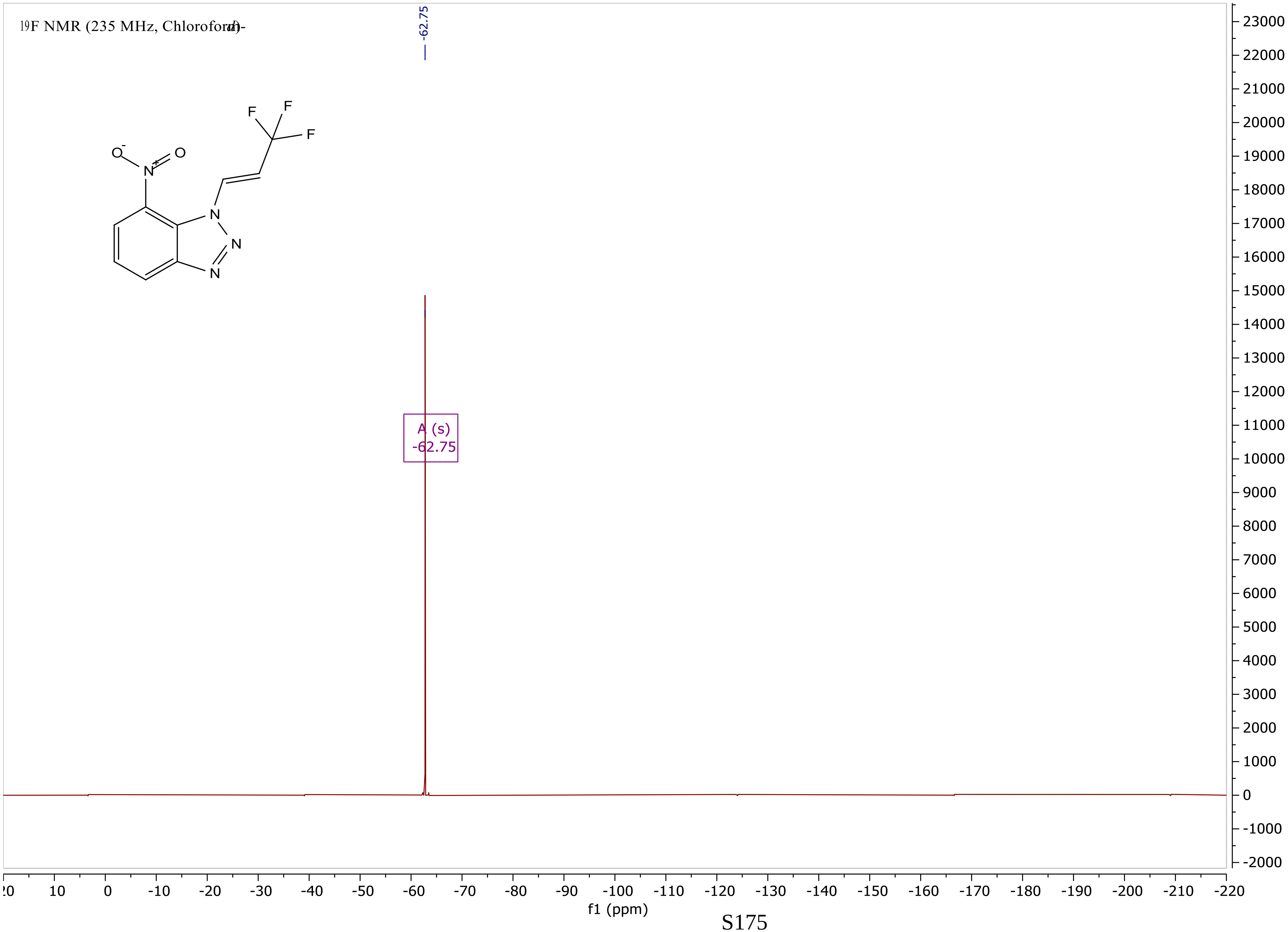
19F NMR (235 MHz, Chloroform-d)
-62.75
A (s)
-62.75
f1 (ppm)

(*E*)-4-Nitro-1-(3,3,3-trifluoroprop-1-en-1-yl)-1*H*-benzo[d][1,2,3]triazole (41)

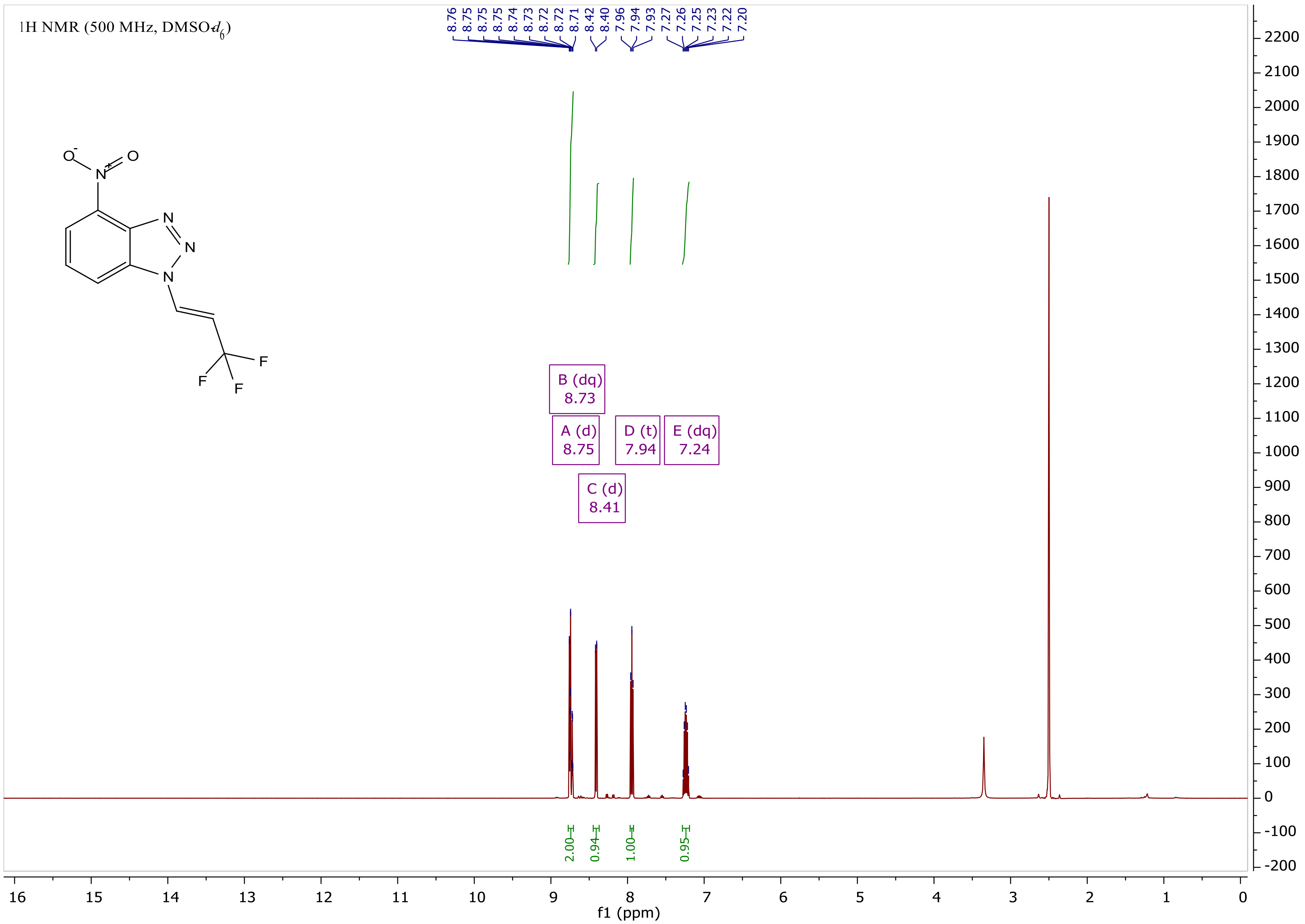

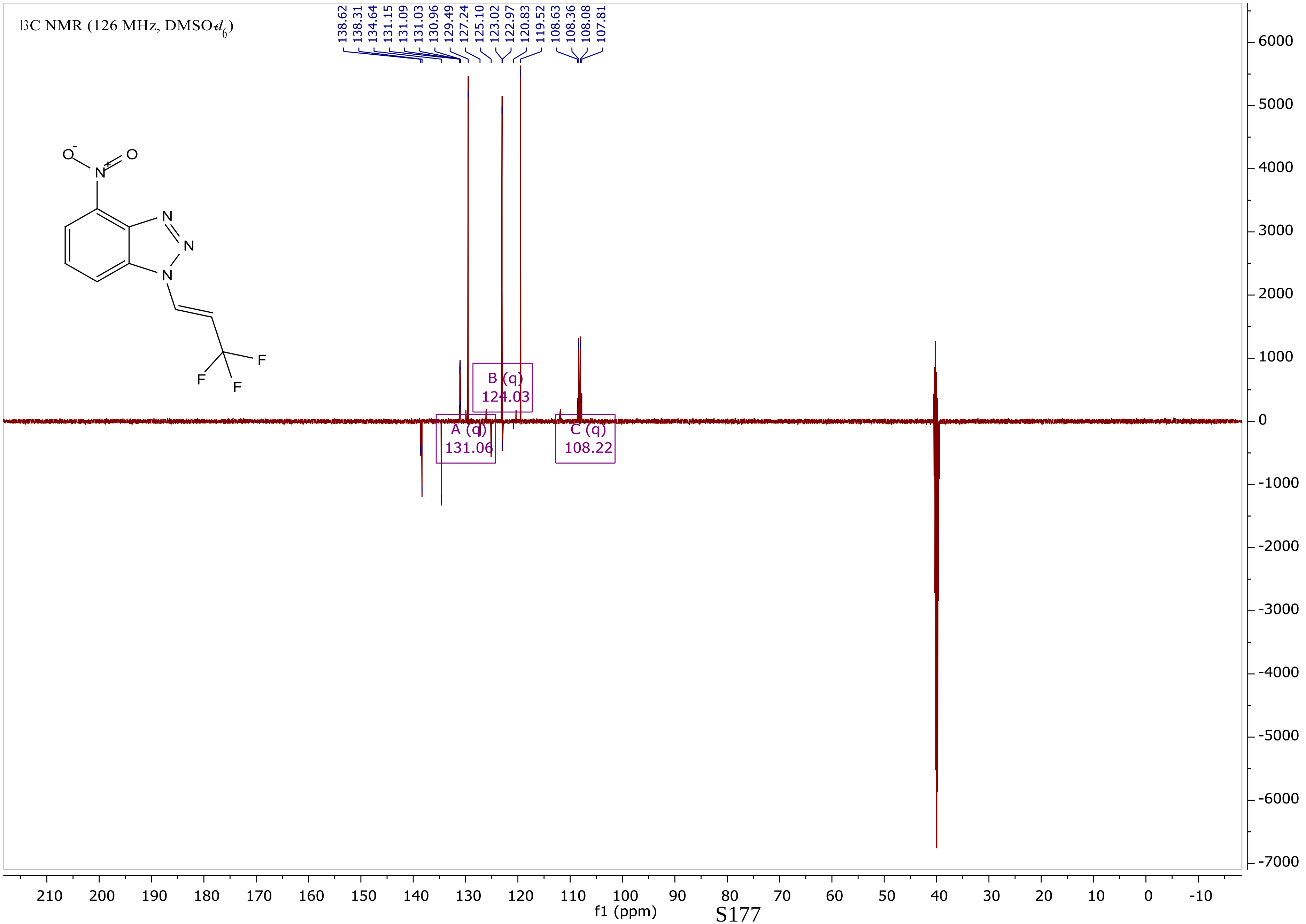
13C NMR (126 MHz, DMSO-d6)
138.62
138.31
134.64
131.15
131.09
131.03
130.96
129.49
127.24
125.10
123.02
122.97
120.83
119.52
108.63
108.36
108.08
107.81
A (q)
131.06
B (q)
124.03
C (q)
108.22
f1 (ppm)

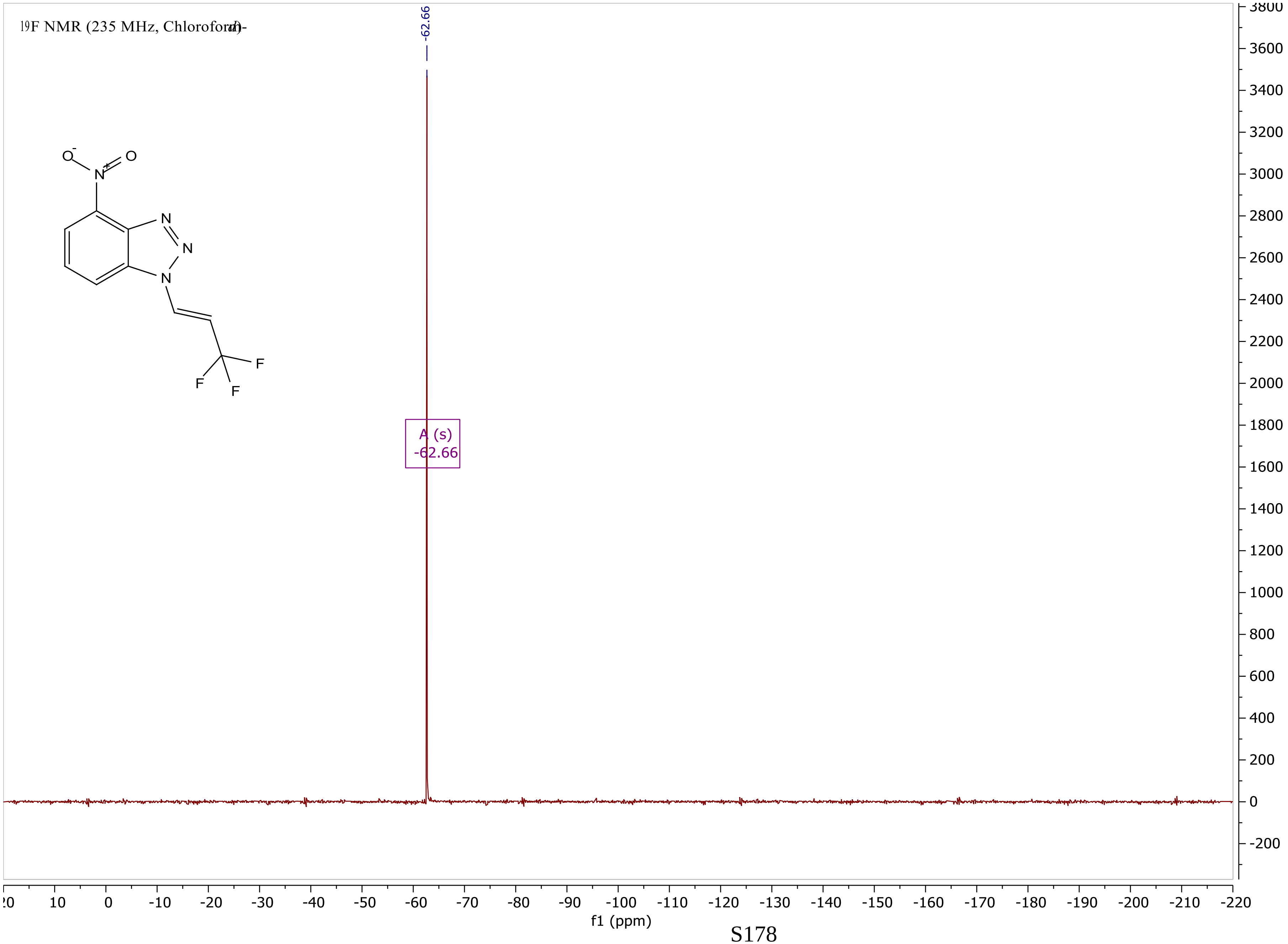
19F NMR (235 MHz, Chloroform-d)
-62.66
A (s)
-62.66
f1 (ppm)

(*E*)-6-Chloro-9-(3,3,3-trifluoroprop-1-en-1-yl)-9*H*-purine (42)

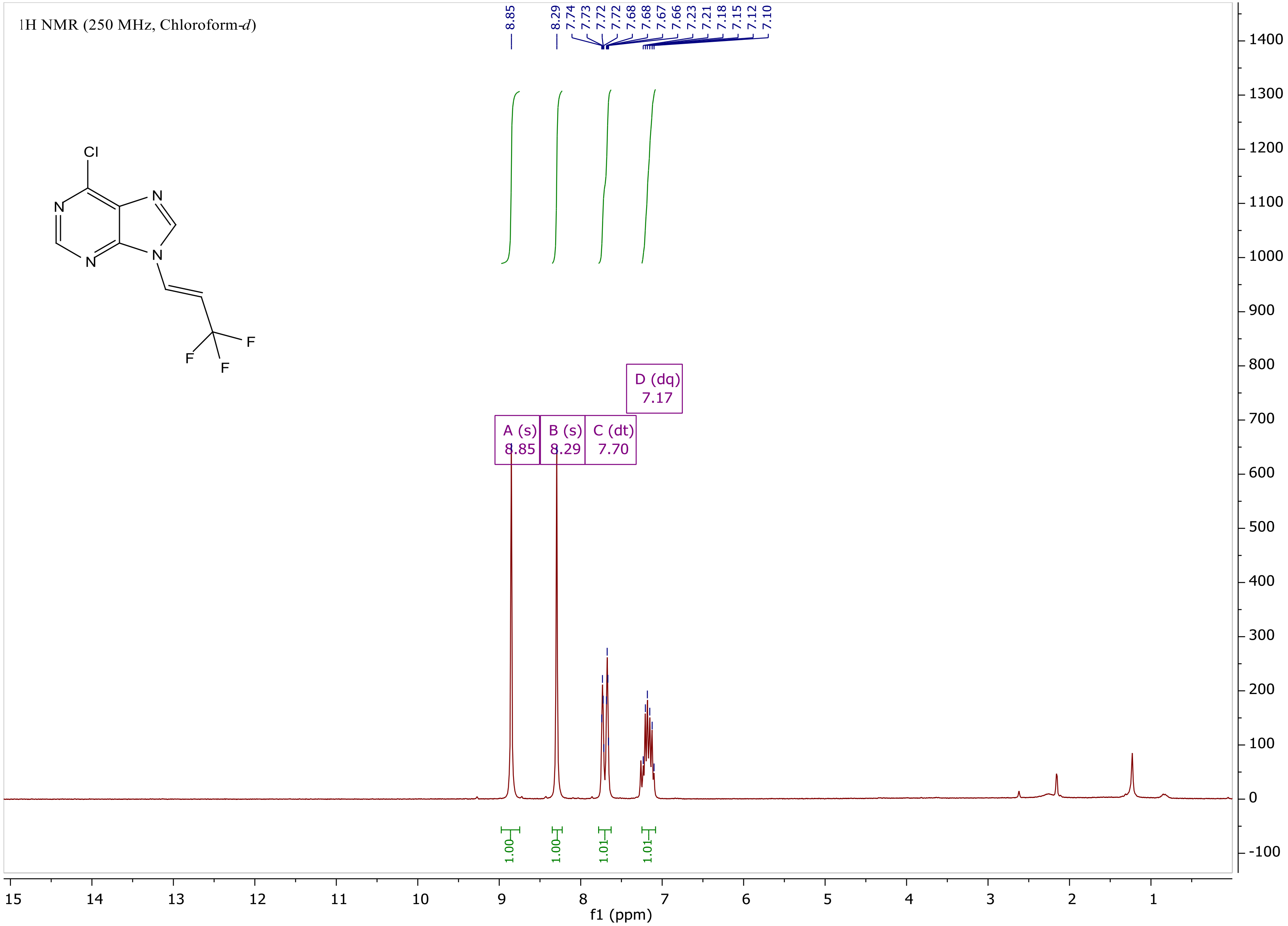

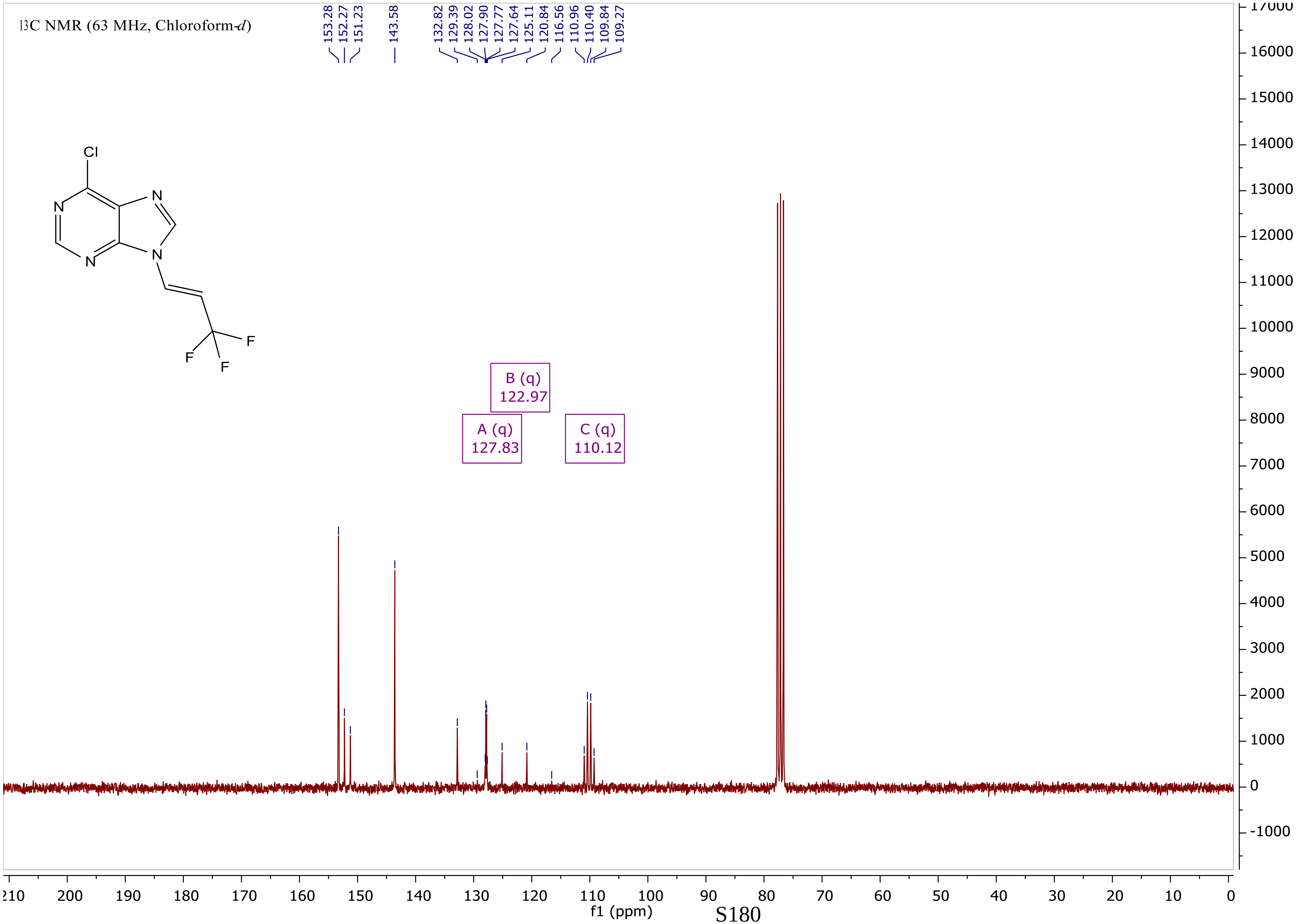
13C NMR (63 MHz, Chloroform-d)
153.28
152.27
151.23
143.58
132.82
129.39
128.02
127.90
127.77
127.64
125.11
120.84
116.56
110.96
110.40
109.84
109.27
Cl
N
N
N
N
F
F
F
B (q)
122.97
A (q)
127.83
C (q)
110.12
f1 (ppm)

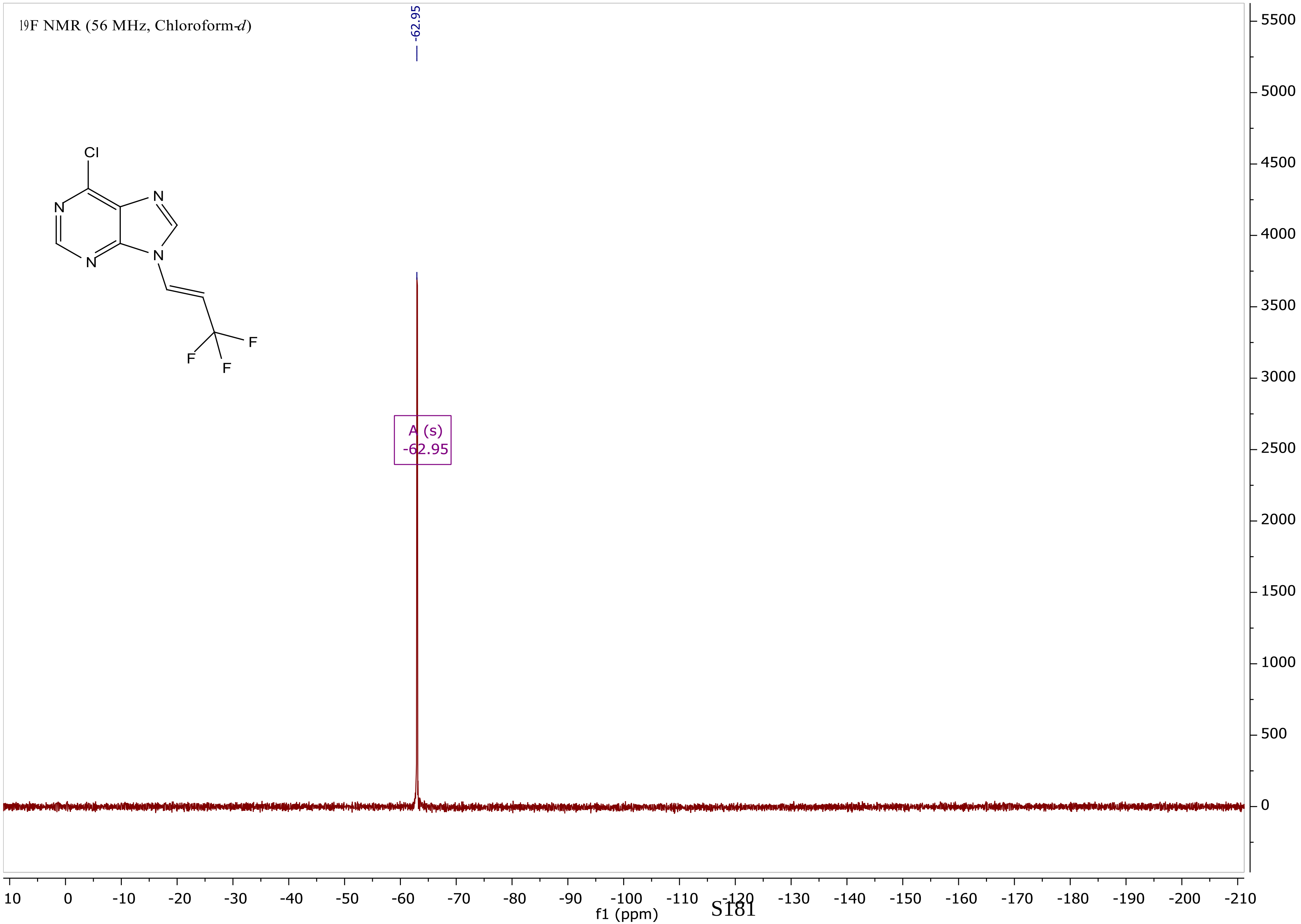

19F NMR (56 MHz, Chloroform-d)
-62.95
A (s)
-62.95
Cl
N
N
N
N
F
F
F
f1 (ppm)

(*E*)-6-Chloro-2-fluoro-9-(3,3,3-trifluoroprop-1-en-1-yl)-9*H*-purine (43)

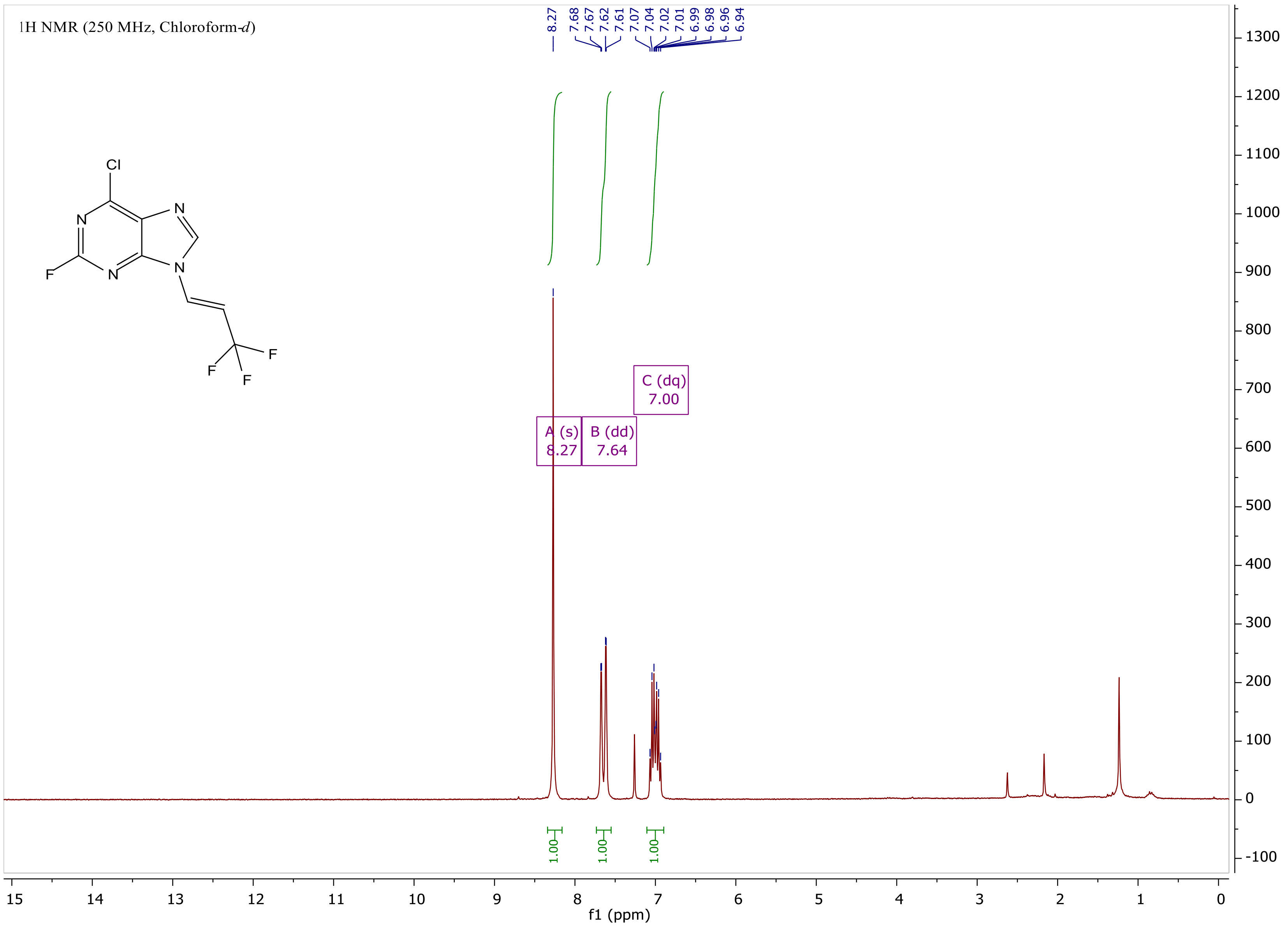

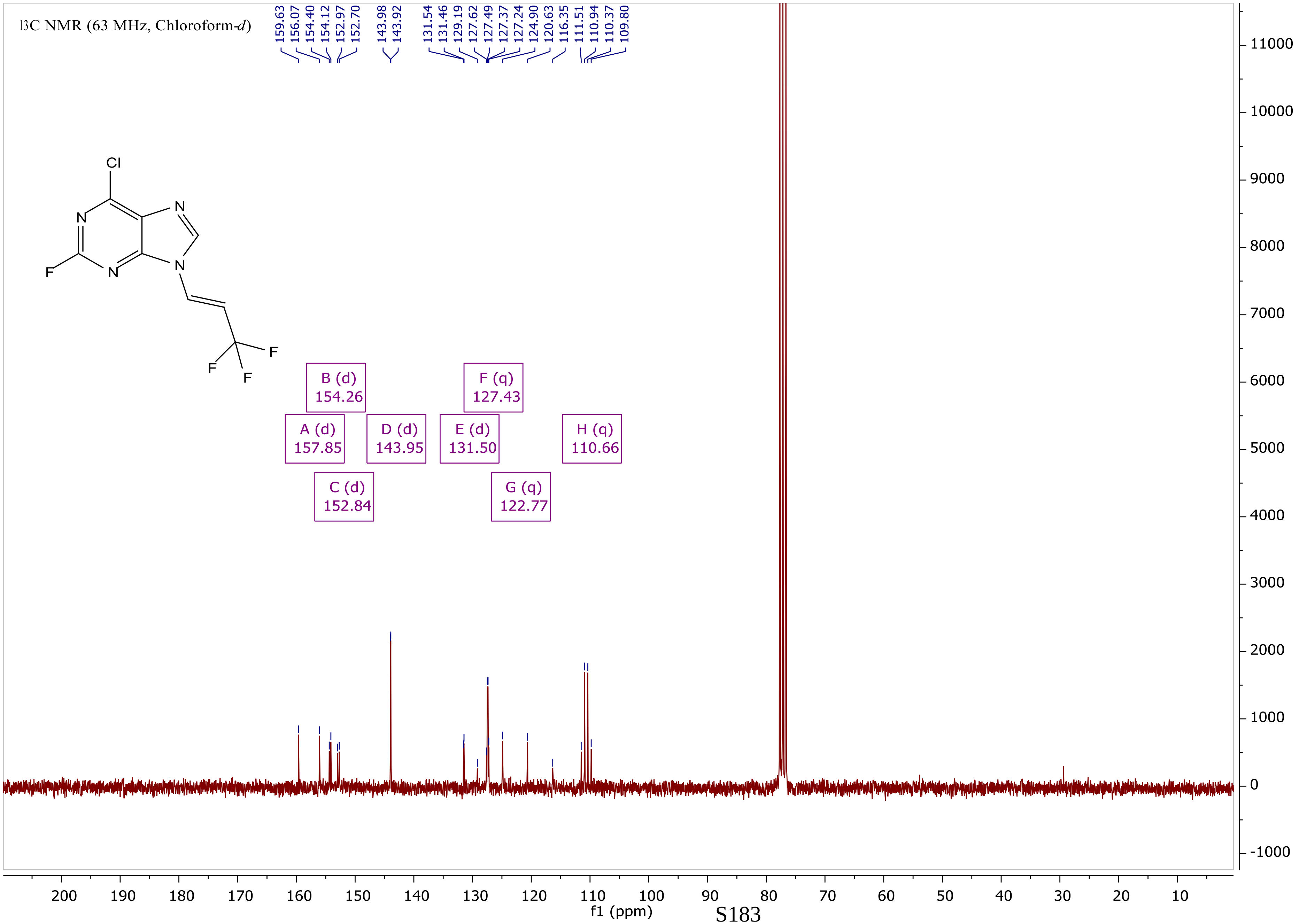
13C NMR (63 MHz, Chloroform-d)
159.63
156.07
154.40
154.12
152.97
152.70
143.98
143.92
131.54
131.46
129.19
127.62
127.49
127.37
127.24
124.90
120.63
116.35
111.51
110.94
110.37
109.80
Cl
N
N
N
N
F
F
F
F
A (d) 157.85
B (d) 154.26
C (d) 152.84
D (d) 143.95
E (d) 131.50
F (q) 127.43
G (q) 122.77
H (q) 110.66
f1 (ppm)

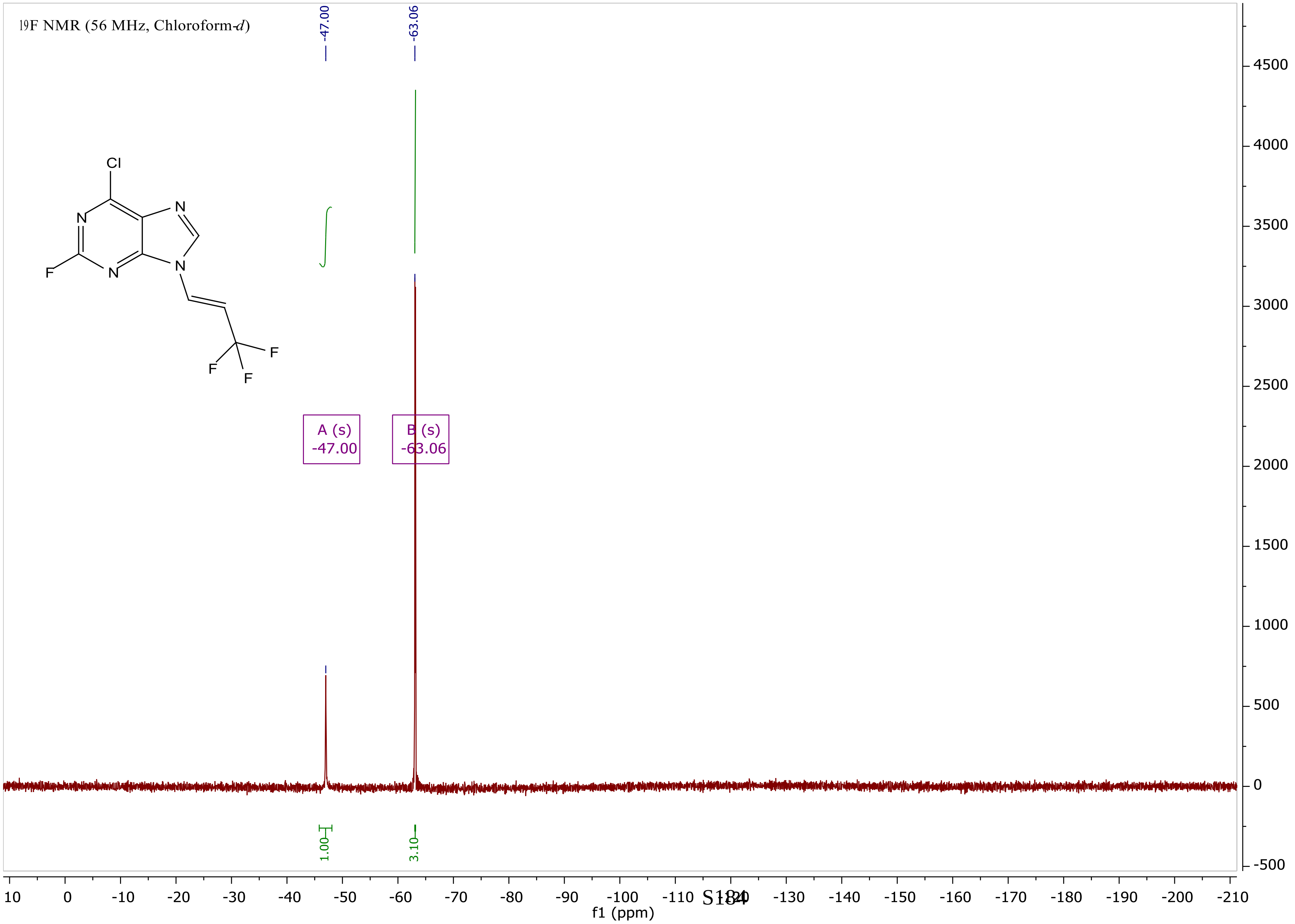

19F NMR (56 MHz, Chloroform-d)
-47.00
-63.06
Cl
N
N
N
N
F
F
F
F
A (s)
-47.00
B (s)
-63.06
1.00
3.10
f1 (ppm)

(*E*)-2,6-Dichloro-9-(3,3,3-trifluoroprop-1-en-1-yl)-9*H*-purine (44)

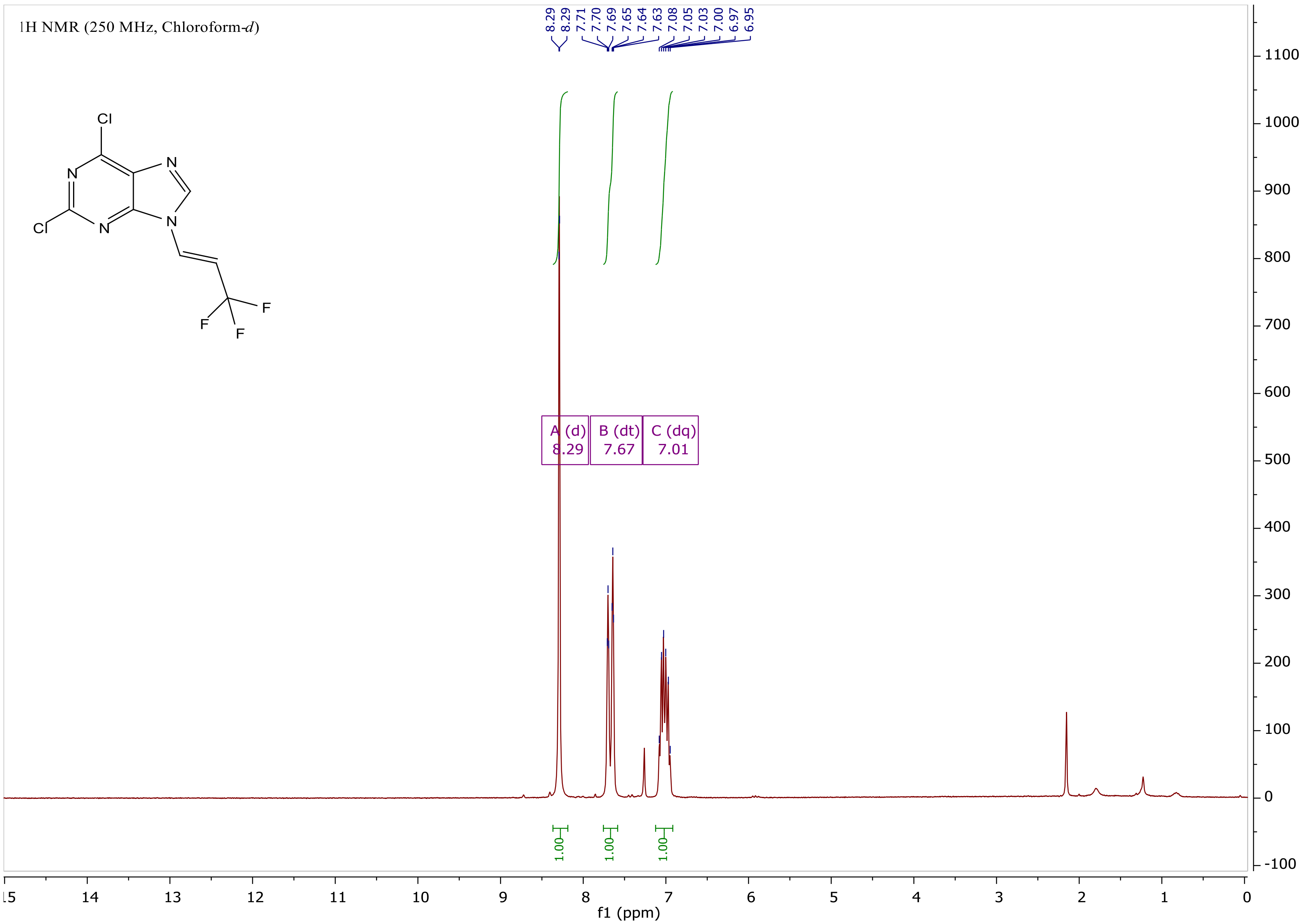

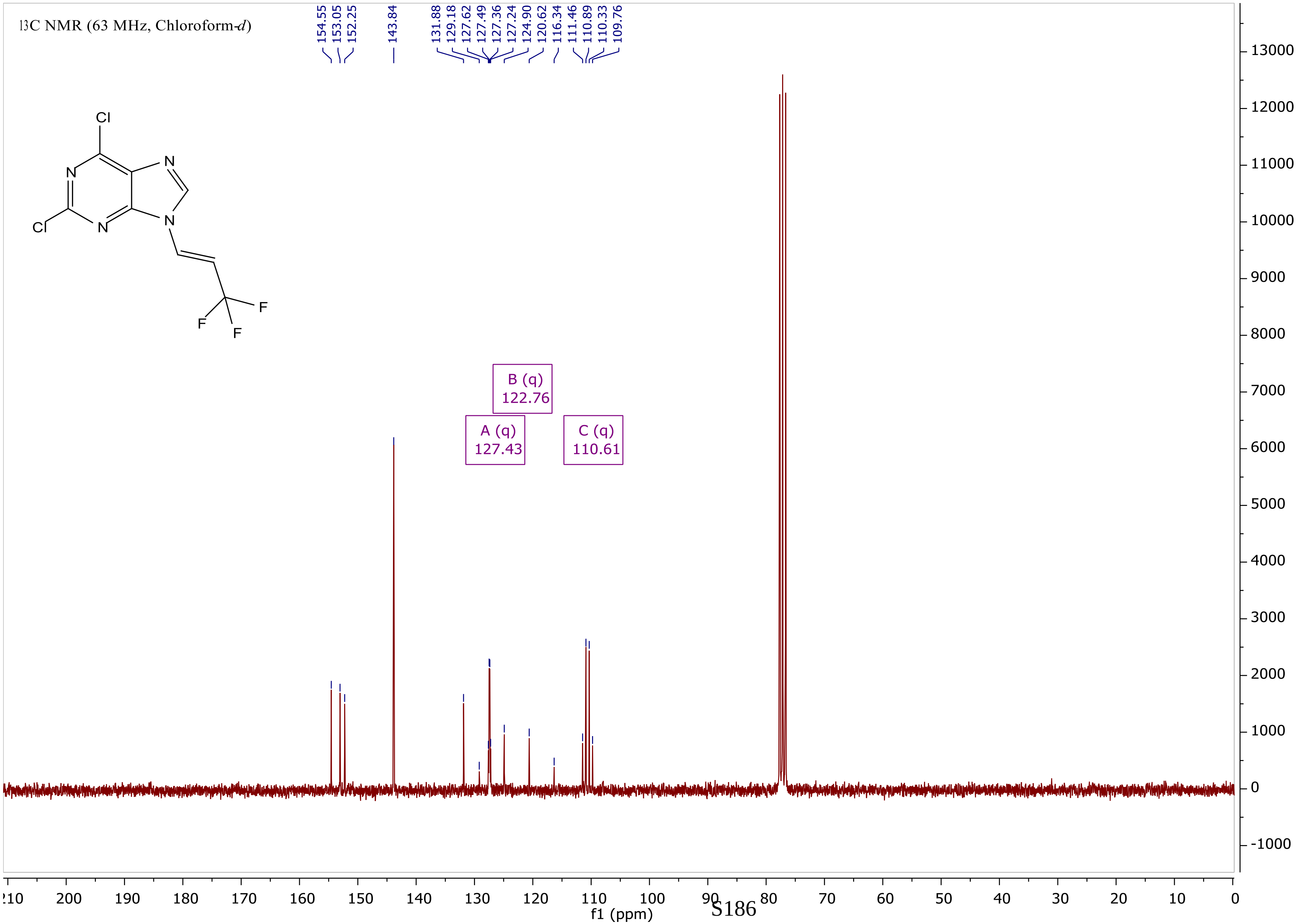
13C NMR (63 MHz, Chloroform-d)
154.55
153.05
152.25
143.84
131.88
129.18
127.62
127.49
127.36
127.24
124.90
120.62
116.34
111.46
110.89
110.33
109.76
Cl
N
N
N
N
Cl
F
F
F
B (q)
122.76
A (q)
127.43
C (q)
110.61
f1 (ppm)

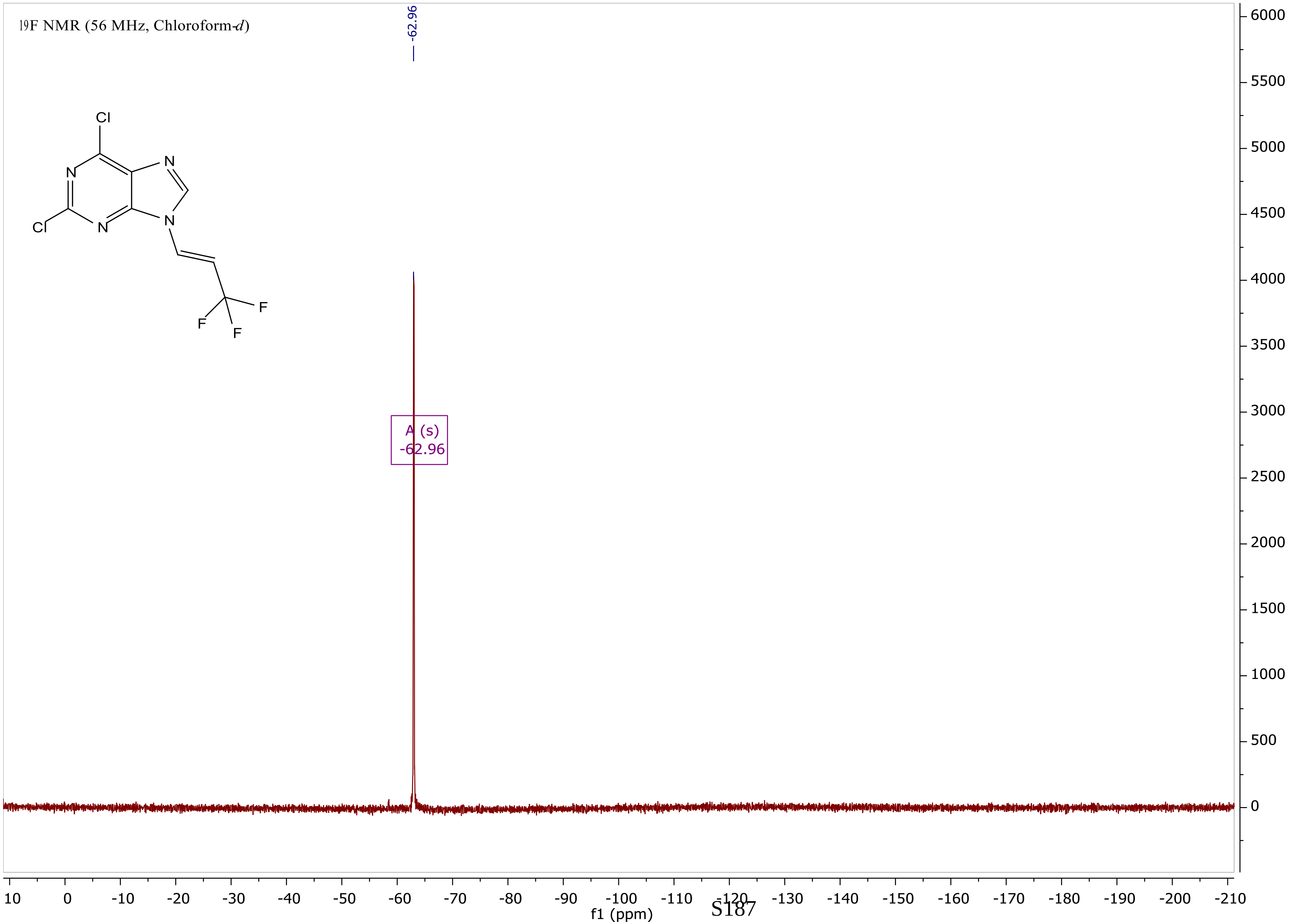
19F NMR (56 MHz, Chloroform-d)
-62.96
A (s)
-62.96
f1 (ppm)

(3,3,3-Trifluoroprop-1-en-1-yl)isoindoline-1,3-dione (45)

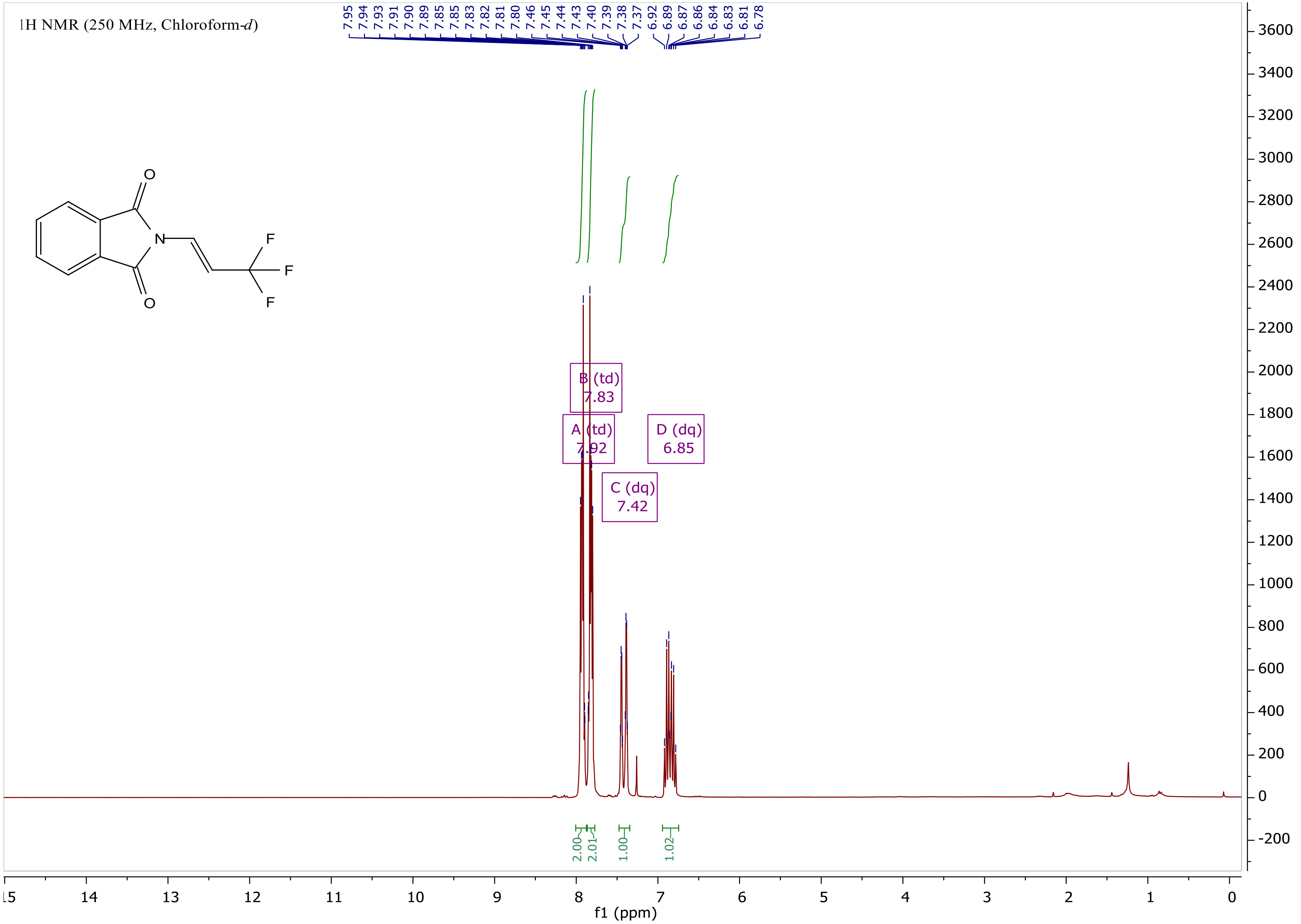

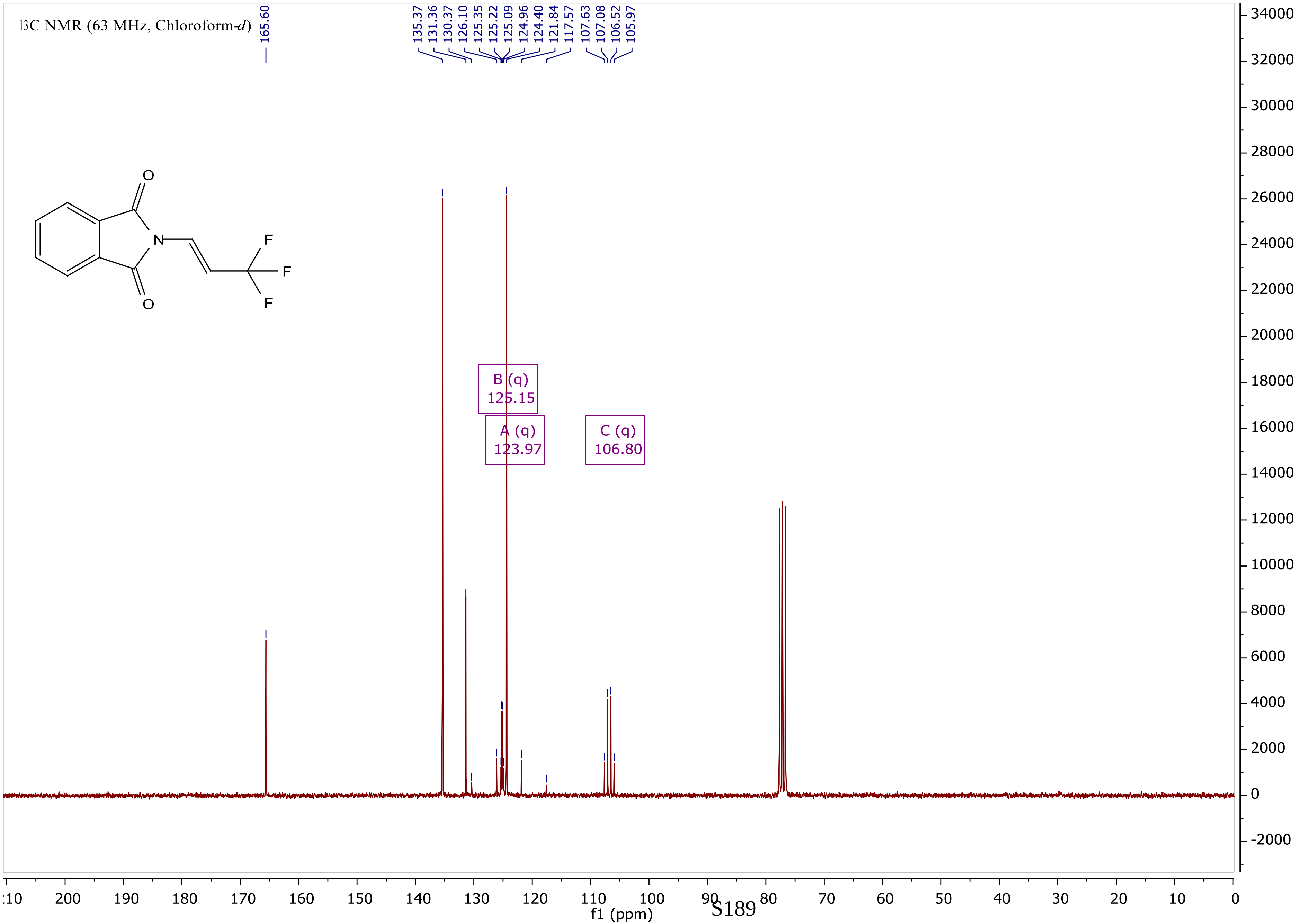
13C NMR (63 MHz, Chloroform-d)
165.60
135.37
131.36
130.37
126.10
125.35
125.22
125.09
124.96
124.40
121.84
117.57
107.63
107.08
106.52
105.97
B (q)
125.15
A (q)
123.97
C (q)
106.80
f1 (ppm)

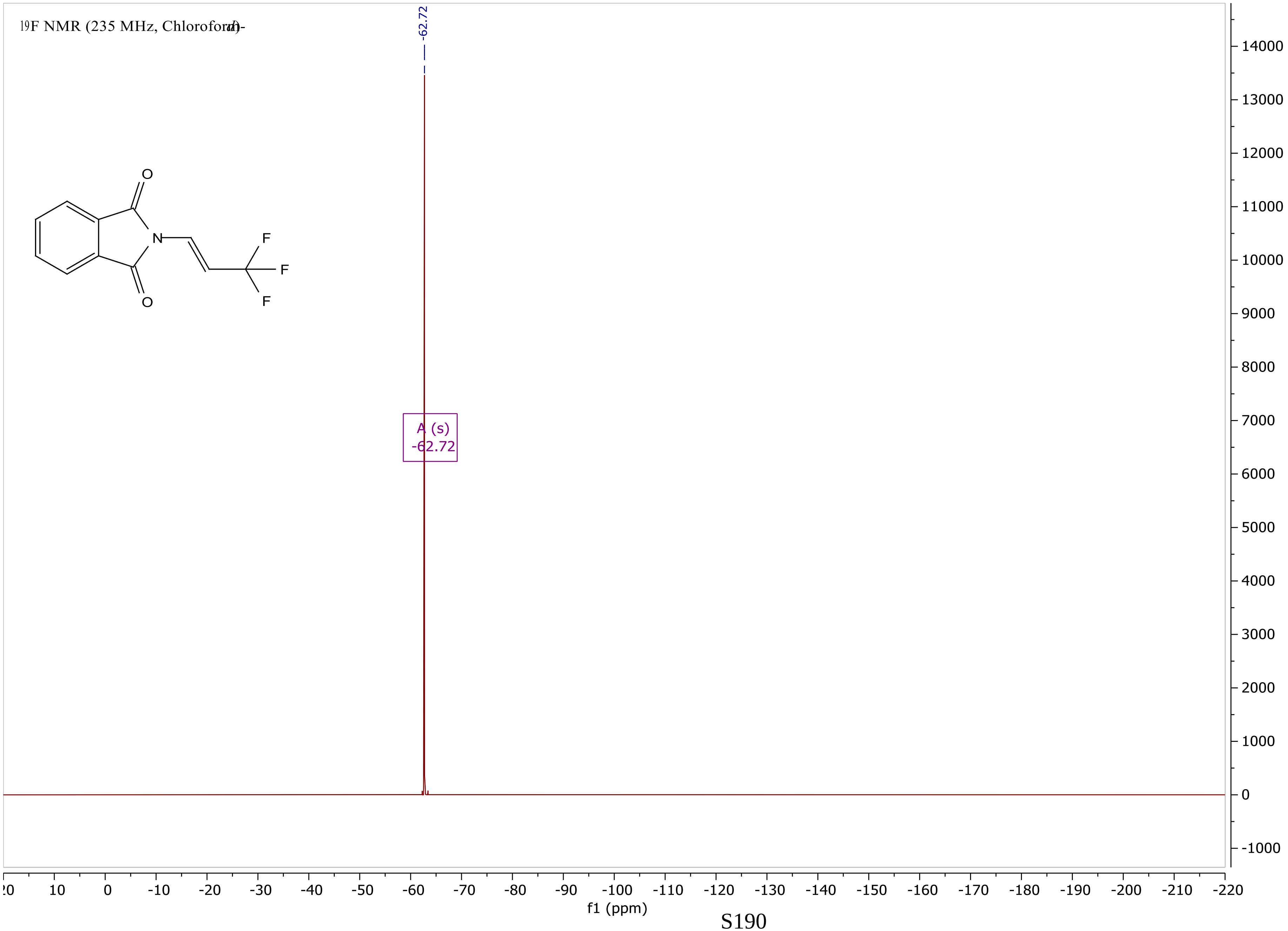
19F NMR (235 MHz, Chloroform-d)
-62.72
A (s)
-62.72
O
N
F
F
F
O
f1 (ppm)
20 10 0 -10 -20 -30 -40 -50 -60 -70 -80 -90 -100 -110 -120 -130 -140 -150 -160 -170 -180 -190 -200 -210 -220
14000 13000 12000 11000 10000 9000 8000 7000 6000 5000 4000 3000 2000 1000 0 -1000

(*E*)-5,5-Diphenyl-3-(3,3,3-trifluoroprop-1-en-1-yl)imidazolidine-2,4-dione (46)

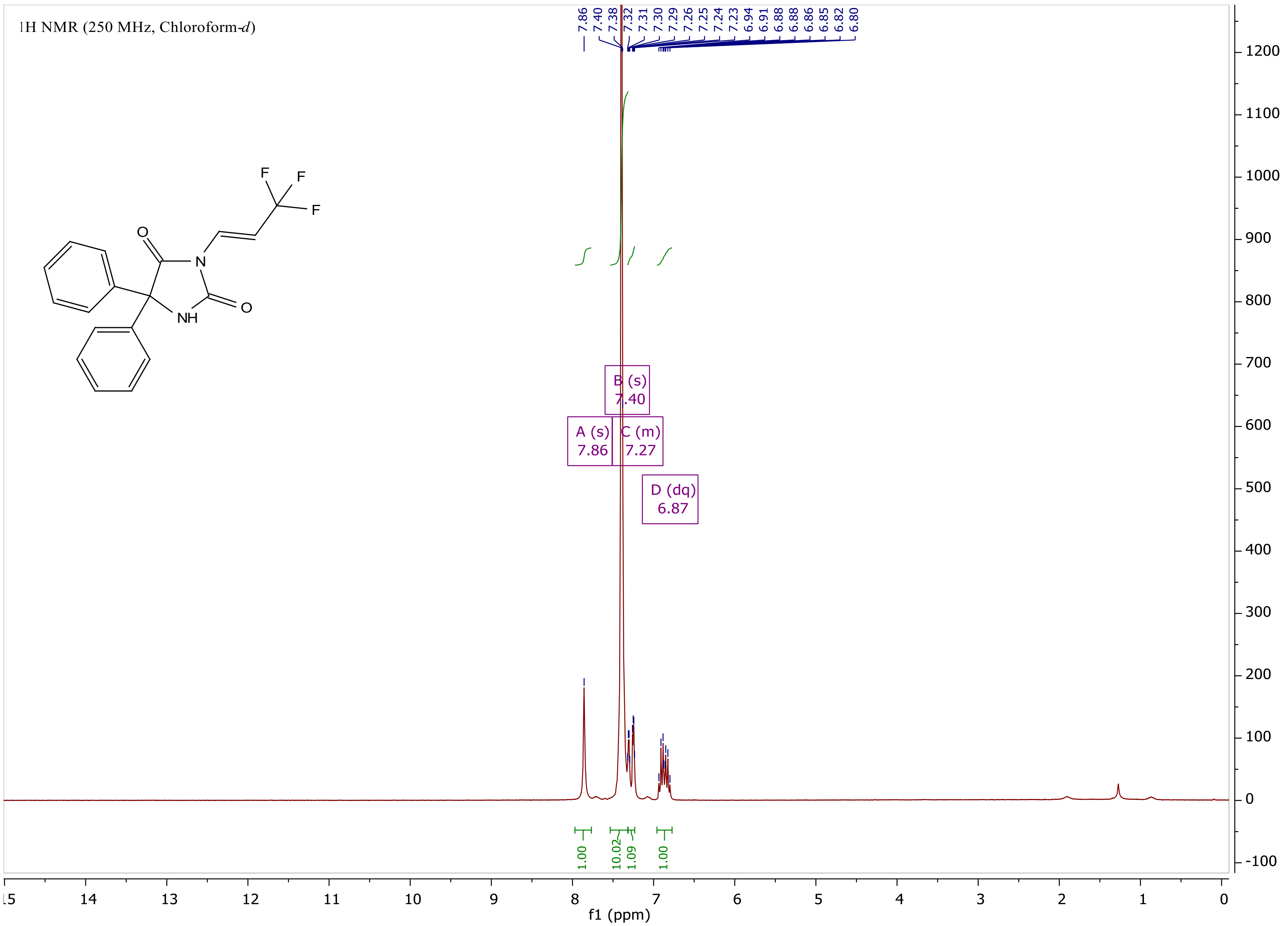

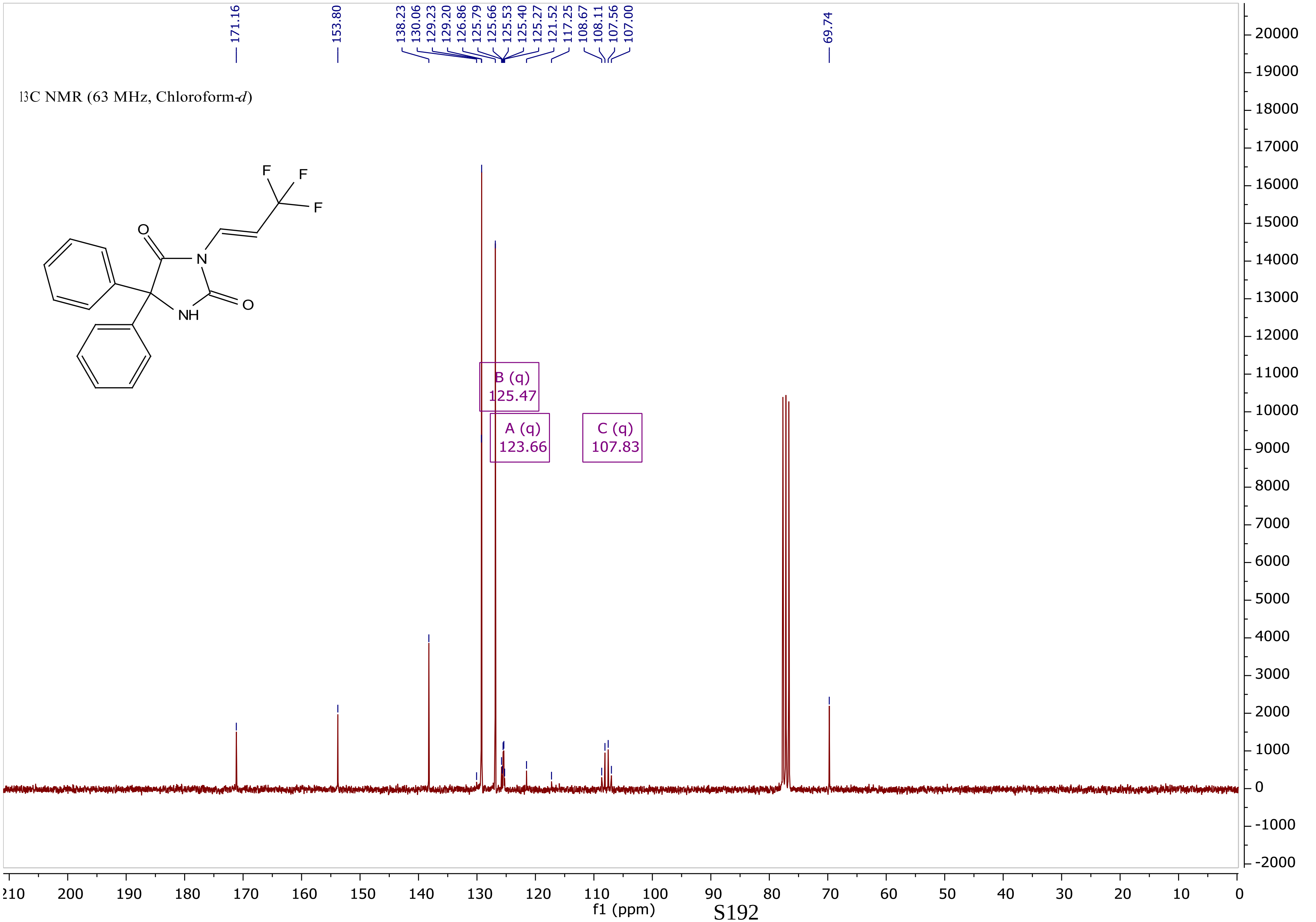
13C NMR (63 MHz, Chloroform-d)
171.16
153.80
138.23
130.06
129.23
129.20
126.86
125.79
125.66
125.53
125.40
125.27
121.52
117.25
108.67
108.11
107.56
107.00
69.74
B (q)
125.47
A (q)
123.66
C (q)
107.83
F
F
F
O
N
O
NH
f1 (ppm)

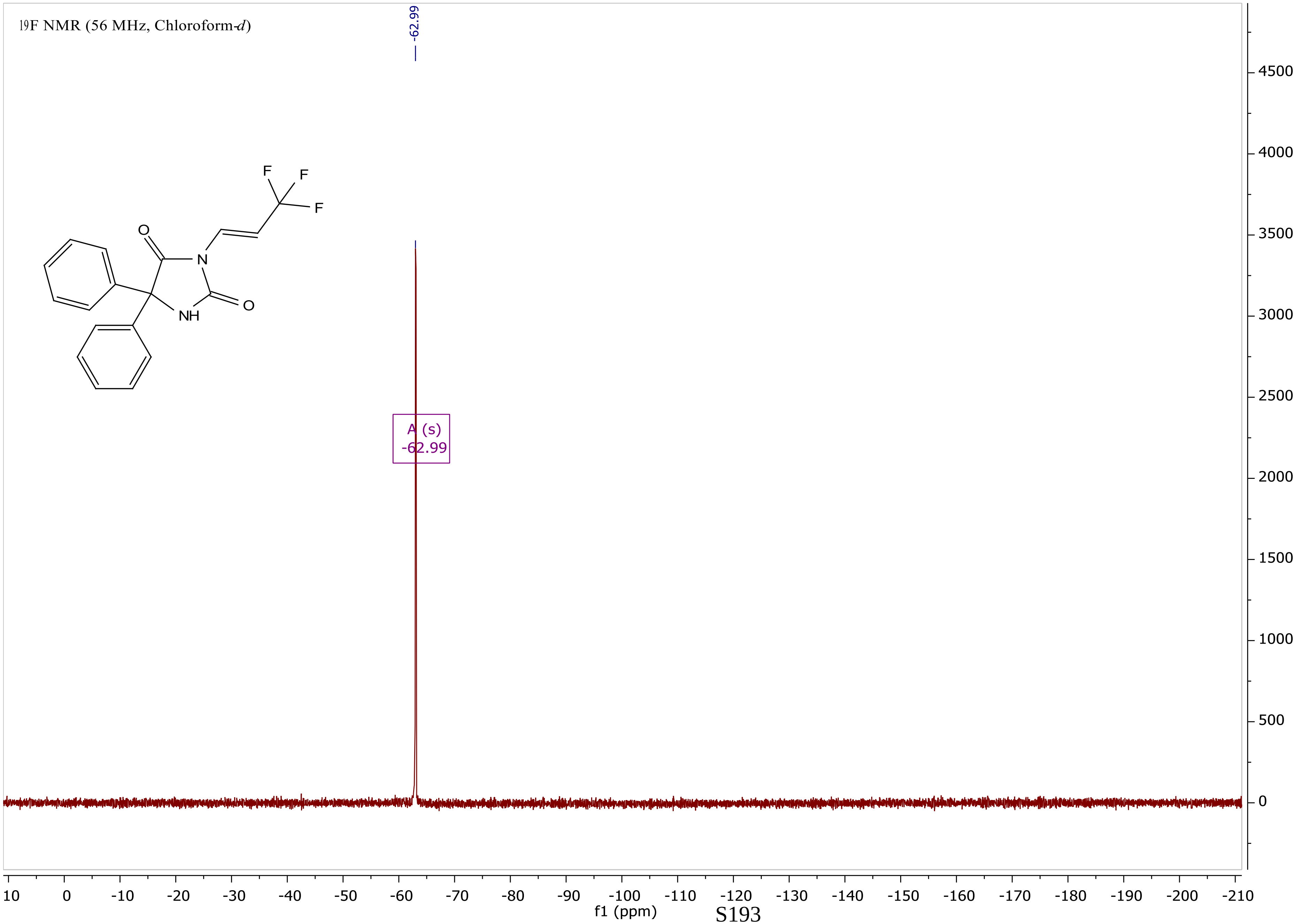
19F NMR (56 MHz, Chloroform-d)
-62.99
A (s)
-62.99
F
F
F
O
N
NH
O
4500
4000
3500
3000
2500
2000
1500
1000
500
0
10 0 -10 -20 -30 -40 -50 -60 -70 -80 -90 -100 -110 -120 -130 -140 -150 -160 -170 -180 -190 -200 -210
f1 (ppm)

[1] Á. Mészáros, A. Székely, A. Stirling, Z. Novák, *Angew. Chem. Int. Ed*. **2018**, *57*, 6643-6647.
[2] Z. Gonda, Z. Novák. *Chem. Eur. J.*, **2015**, *21*, 16801-16806.
[3] Li, F.; Nie, J.; Sun, L.; Zheng, Y.; Ma, J. *Angew. Chem. Int. Ed*. **2013**, *52*, 6255-6258.
[4] Albrecht, B. K. et. al. PCT Int. Appl. **2014**, WO/2014/139326
[5] Zhou, Yi et al. *Synth. Commun.* **2010**, *40*, (17), 2624-2632.
[6] Raut, Santosh et al. *J. Het. Chem.* **2020**, *57*, (3), 1291-1305.
[7] Miller, Michael et al. PCT Int. Appl. **2014**, WO/2014/137723
[8] Sakaizumi, Takeshi et a. *Bull. Chem. Soc. Jpn.*, **1987**, *60*(11), 3903-3909.
